# Water co-catalysis in aerobic olefin epoxidation mediated by ruthenium oxo complexes†‡

**DOI:** 10.1039/d3sc05516g

**Published:** 2024-01-09

**Authors:** Qun Cao, Martin Diefenbach, Calum Maguire, Vera Krewald, Mark J. Muldoon, Ulrich Hintermair

**Affiliations:** a School of Chemistry and Chemical Engineering, Queen's University Belfast Northern Ireland UK m.j.muldoon@qub.ac.uk; b Dynamic Reaction Monitoring Facility, Institute for Sustainability, University of Bath UK u.hintermair@bath.ac.uk; c Theoretical Chemistry, Department of Chemistry, Technische Universität Darmstadt Germany krewald@chemie.tu-darmstadt.de

## Abstract

We report the development of a versatile Ru-porphyrin catalyst system which performs the aerobic epoxidation of aromatic and aliphatic (internal) alkenes under mild conditions, with product yields of up to 95% and turnover numbers (TON) up to 300. Water is shown to play a crucial role in the reaction, significantly increasing catalyst efficiency and substrate scope. Detailed mechanistic investigations employing both computational studies and a range of experimental techniques revealed that water activates the Ru^VI^ di-oxo complex for alkene epoxidation *via* hydrogen bonding, stabilises the Ru^IV^ mono-oxo intermediate, and is involved in the regeneration of the Ru^VI^ di-oxo complex leading to oxygen atom exchange. Distinct kinetics are obtained in the presence of water, and side reactions involved in catalyst deactivation have been identified.

## Abstract 2

A versatile Ru-porphyrin catalyst system was developed for the mild and selective aerobic epoxidation of alkenes in which water was shown to play a key role as corroborated by detailed mechanistic studies.

## Graphical abstract

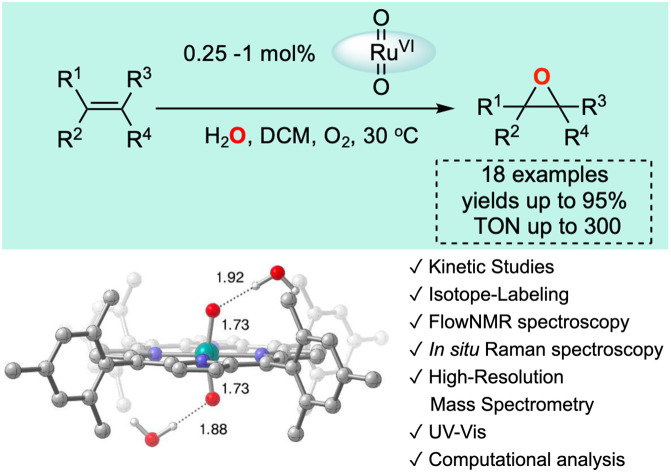

## Introduction

**Fig. 1:**
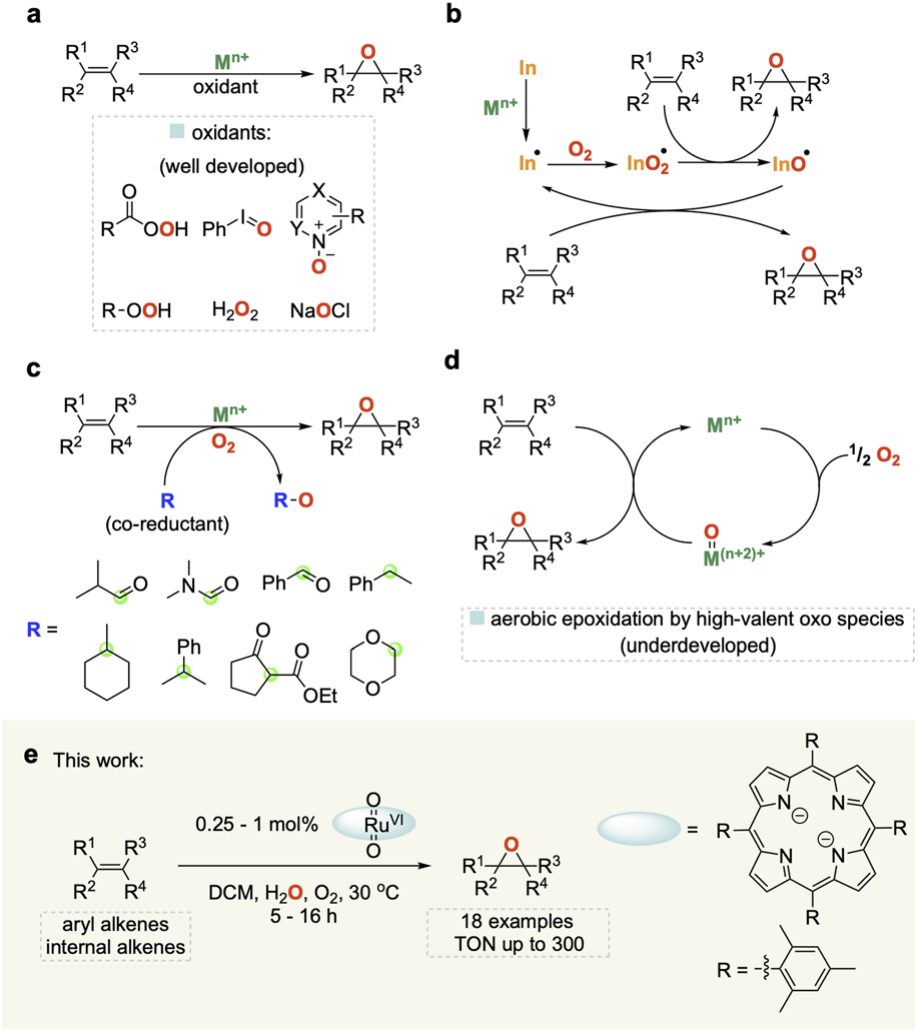
(a) Catalytic epoxidation of alkenes using stoichiometric oxidants. (b) Catalytic aerobic epoxidation *via* metal-initiated free radical oxidation (In = radical initiator). (c) Catalytic aerobic epoxidation with sacrificial co-reductants (R) (green circle = oxidation site). (d) Direct catalytic aerobic epoxidation by high-valent oxo species without co-reductants. (e) This work: water co-catalysed aerobic alkene epoxidation catalysed by ruthenium porphyrin complexes.

Epoxides are important building blocks in synthetic chemistry and are widely utilised in the production of polymers, resins, pharmaceuticals, agrochemicals and fragrances.^1–4^ Traditional methods for synthesizing epoxides use (super)stoichiometric amounts of organic peroxides on olefins or the dehydrochlorination of chlorohydrins with base.^5^ Much work has been carried out on developing more sustainable catalytic methods for the epoxidation of alkenes using a variety of terminal oxidants including peracids, *N*-oxides, hypervalent iodine, and H_2_O_2_ (Fig. 1a).^6–9^ Molecular oxygen (O_2_) represents the cleanest and most easily available oxidant, but harnessing it for selective oxidation catalysis remains a challenge due to its propensity to undergo radical reactions that are difficult to control and typically lead to low selectivity as well as limited substrate scope (Fig. 1b).^10^

A popular strategy to tame dioxygen for aerobic oxidation chemistry is to employ sacrificial co-reductants that increase selectivity and allow for a wider substrate scope, but in turn diminish the sustainability of the reaction (Fig. 1c).^11^ A few notable examples have shown that it is possible to generate molecularly defined high-valent oxo species from the reaction of O_2_ with metals such as Fe and Ru (Fig. 1d) that activate and transfer oxygen to organic substrates in a controlled manner without the use of sacrificial co-reductants.^12–14^ In 1985 Groves and co-workers first reported that [(TMP)Ru^VI^(O)_2_] (TMP = 5,10,15,20-tetramesitylporphyrin) could be used for the mild and selective epoxidation of alkenes, and related complexes for catalytic aerobic epoxidations have been studied since.^15–22^ However, all of these still require high catalyst loadings (2–10 mol%) affording low turnover numbers (TON < 50) with a narrow substrate scope to date. Further improvement of this promising but underdeveloped area of catalysis is hampered by a limited understanding of the reaction mechanism.

Here we report the development of an efficient and broadly applicable homogeneous catalytic system based on a ruthenium porphyrin complex along with important mechanistic insights derived from detailed investigations using orthogonal *operando* analytics, isotopic labelling, and quantum chemical calculations to elucidate the mechanistic origin of our finding that water significantly improves catalyst performance.

## Results and discussion

### Initial considerations

**Fig. 2:**
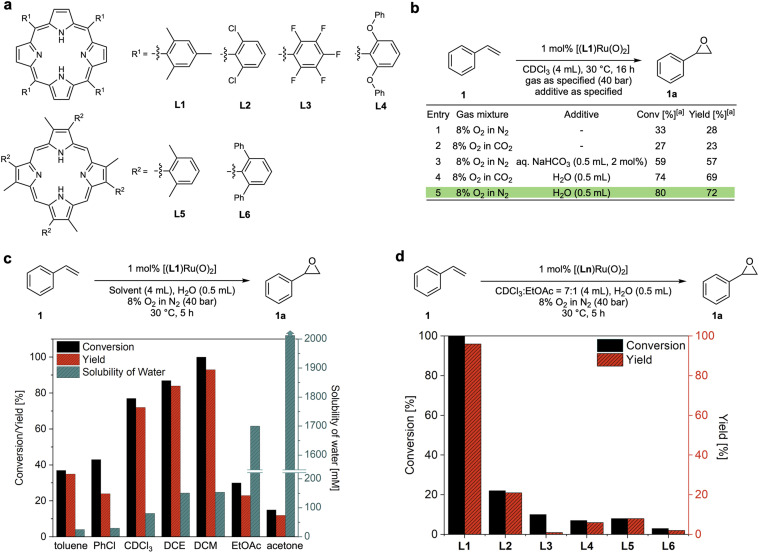
Reaction optimisation of dioxo-Ru catalysed aerobic epoxidation of styrene. (a) Porphyrin ligands tested. (b) Effects of water and CO_2_ (commercial grade solvents without drying). (c) Effects of solvents and water content for dioxo-Ru-porphyrin catalysed aerobic epoxidation of styrene at 30 °C^28,29^ (DCE = 1,2-dichloroethane, DCM = dichloromethane, EtOAc = ethyl acetate). (d) Comparison of conversions and yields for different dioxo-Ru-porphyrin complexes.

The reason why efficient aerobic oxidation catalysis without sacrificial co-reductants is difficult to achieve lies in the requirement for two challenging features to be combined in a single catalyst: (i) the generation of a highly oxidising yet selective catalyst intermediate from O_2_, and (ii) the effective utilisation of both oxygen atoms to close the catalytic cycle *via* a reduced state that can bind and activate O_2_ again. Groves' [(TMP)Ru^VI^(O)_2_] complexes showed promise in fulfilling both of these requirements, but with limited efficiency in terms of rate and stability.^15^ Building on this seminal work, Che and co-workers reported that the addition of aqueous NaHCO_3_ was found to improve the activity of a Ru-porphyrin catalyst in aerobic epoxidation–isomerisation reactions for converting aryl alkenes to their corresponding aldehydes.^23,24^ Following earlier reports that peroxocarbonate species can be effective oxidants for epoxidation reactions,^25–27^ we set out to investigate whether this could be a strategy to improve these catalytic systems. We thus prepared a range of porphyrin ligands (Fig. 2a) and their corresponding *trans*-dioxo ruthenium complexes for a systematic study of their catalytic behaviour in olefin epoxidation under various conditions. When [(L1)Ru^VI^(O)_2_] was tested for the aerobic epoxidation of styrene (1) in CDCl_3_ at 30 °C using 8% O_2_ in N_2_ (40 bar) only ∼30% of styrene oxide (1a) was obtained at 1 mol% catalyst loading (Fig. 2b, entry 1), a result which is consistent with previous reports.^15^ The use of 8% O_2_ in CO_2_ did not improve reactivity; in fact, it provided slightly lower product yields (Fig. 2b, entry 2). As reported by Che, the addition of aqueous NaHCO_3_ improved the catalytic activity of [(L1)Ru^VI^(O)_2_] to a 57% yield of 1a. Intriguingly, a control experiment revealed that the addition of water alone significantly improved the catalysis, providing conversions of 80% with a 72% yield of 1a in the absence of any CO_2_ (Fig. 2b, entry 5). Katsuki and co-workers previously found that water was important in the asymmetric sulfide oxidation and alkene epoxidation using [(salen)Ru(NO)] complexes.^18,19^ They proposed reaction pathways where water acted as proton transfer mediator, but limited mechanistic studies were carried out to support this hypothesis. We thus set out to investigate this intriguing finding and understand how water influences these aerobic oxidation reactions to further improve the catalysis.

### Reaction optimisation

Using 1 mol% [(L1)Ru^VI^(O)_2_], we examined the effect of water on the aerobic epoxidation of 1 with various solvents and additives under non-oxygen limiting conditions (Tables S1–S3†). The employment of moderately polar solvents (*e.g.* dichloromethane, chloroform, 1,2-dichloroethane, toluene, trifluorotoluene, chlorobenzene) led to higher product yields compared to more coordinating solvents such as acetone and acetonitrile, suggesting competition with substrate or O_2_ binding to the catalyst. No reactivity was observed in methanol and ethanol, and even small quantities of ethanol in chloroform were sufficient to shut down catalytic turnover (entry 12, Table S1†). This is likely due to alcohol oxidation, which is known for Ru oxo species,^30^ overriding alkene epoxidation.^31^ Within the range of weakly coordinating solvents, the catalytic activity increased with the solubility of water (Fig. 2c). Excellent catalytic performance was obtained with dichloromethane (DCM) as the solvent when an excess of water was added, affording full conversion of 1 and a 93% yield of 1a within 5 hours at 30 °C (Fig. 2c). Control experiments found that using an excess of water to ensure complete saturation (creating a biphasic system) gave the same results as using single-phase DCM saturated with water, showing the catalysis to be genuinely homogeneous (Fig. S3a†). Good performance could also be achieved using CDCl_3_ or PhCF_3_ with the addition of a small amount of ethyl acetate which seemed effective in affording good activity by increasing the water content of the solvent without deactivating the catalyst (Table S1†). To investigate ligand effects, dioxo-Ru-porphyrin complexes bearing different meso-substituted tetraarylporphyrins (L1–4) and sterically hindered β-pyrrole substituted porphyrins (L5 and L6) were tested in the aerobic epoxidation of 1 under these optimised reaction conditions (Fig. 2d). Both conversion and yield of 1a decreased noticeably when bulkier, more electron-withdrawing substituents were introduced to different positions in the porphyrin ligand of the catalyst, and Groves' electron-rich L1 (ref. 15) emerged as the best ligand to be taken forward in our investigation.

### Kinetic analysis

**Fig. 3:**
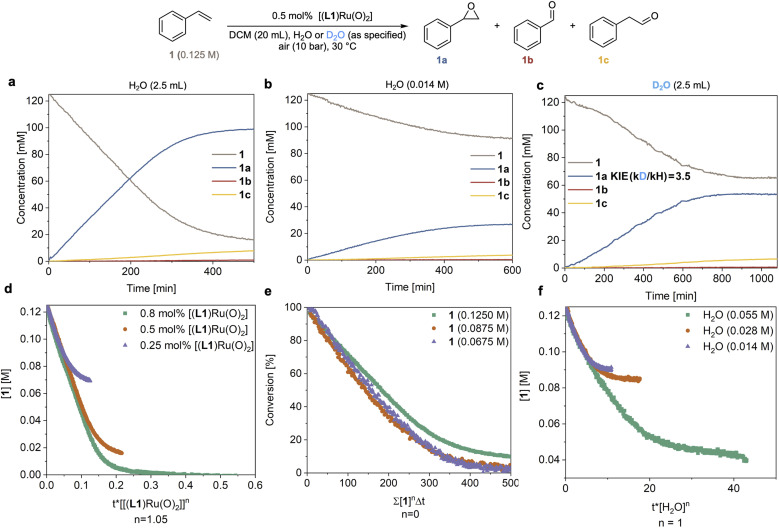
Kinetic analysis from *operando*^1^H FlowNMR spectroscopy. (a) Reaction progress in DCM saturated with an excess of H_2_O. (b) Reaction progress in laboratory grade DCM (residual H_2_O content 14 mM). (c) Reaction progress in DCM saturated with an excess of D_2_O (99.9 atom% D). (d) Determination of the order in catalyst using VTNA. (e) Determination of the order in 1 using VTNA. (f) Determination of the order in H_2_O using VTNA.

In order to understand the origin of the much improved performance [(L1)Ru^VI^(O)_2_] in the presence of water, we monitored the epoxidation of 1 with high-resolution FlowNMR spectroscopy in a recirculating batch setup (for details see method C in the ESI†).^32^ Reaction progress was monitored by ^1^H FlowNMR with a 2 min time interval which allowed us to track the starting material and products in real time, providing high-quality concentration profiles suitable for reaction progress kinetic analysis (RPKA) *via* variable time normalisation analysis (VTNA).^33,34^ Using 10 bar of air (21% O_2_) and 0.5 mol% catalyst the reaction proceeded well in water-saturated DCM at 30 °C, steadily consuming 1 with apparent zero order kinetics at a rate of 0.30 mM min^−1^ to reach 86% conversion and a yield of 78% 1a within 7 hours (Fig. 3a). We could also observe the slow, gradual isomerisation of the epoxide 1a into aldehyde 1c (up to 6%) and the formation of a small amount of benzaldehyde 1b (<1%). Control experiments found no signs for oxygen limitation under the conditions applied when different partial pressures of O_2_ were used (Table S3 and Fig. S8†). When the reaction was carried out using laboratory grade DCM without water added (with a residual concentration of 14 mM) the reaction proceeded with identical selectivity but approximately four times slower (*r* = 0.07 mM min^−1^) than the reaction with an excess of water (Fig. 3a*vs.*3b). When an excess of D_2_O was used instead of H_2_O a rate of 0.09 mM min^−1^ was observed (Fig. 3c), corresponding to a H/D kinetic isotope effect (KIE) of 3.5. This value clearly shows the involvement of water in the catalysis and indicates O–H/D bond breaking to be part of a kinetically significant step in the catalytic cycle (further discussed below). Evidence of O_2_ being the terminal oxidant in the catalysis was obtained from an experiment in which 1 mol% of [(L1)Ru^VI^(O)_2_] was tested with 1 in wet DCM under an N_2_ atmosphere and only 1% of 1a was obtained after 6 hours at 30 °C (Fig. S4a†).

Systematically varying catalyst, substrate and water concentration revealed a first order dependence on [(L1)Ru^VI^(O)_2_] (Fig. 3d), zero order in substrate 1 (Fig. 3e), and first order in water (Fig. 3f and S3b†) in the initial part of the reaction before deactivation led to a deviation of the kinetics from about 65% conversion (TON = 130) onwards (see below section discussing catalyst deactivation). The catalytic rate law thus obtained differs from that previously reported by others using the same and similar Ru-porphyrin catalysts. Che and co-workers studied catalysts with para-substituted tetraphenylporphyrins and observed a rate law for alkene epoxidation of rate = k[Ru^VI^][alkene] using dried organic solvents.^35^ It is worth noting that in this study, as well as most other previous studies, the amount of residual water is not reported to have been controlled or measured.^15–17,35^ Our results also differ from unpublished data in the PhD thesis of Ahn^36^ who studied [(L1)Ru^VI^(O)_2_] in detail, including testing the effect of added water but unfortunately under limiting oxygen conditions. When we carried out an experiment using dried DCM (20 ppm or 1.1 mM residual water content) with 0.5 mol% [(L1)Ru^VI^(O)_2_] the reaction afforded 1a in only 3% yield after 6 h (Fig. S4b†), consistent with previous reports that have found slow reaction rates.^15–17,35,36^^1^H NMR spectroscopic analysis of the dry DCM reaction showed that 47% of [(L1)Ru^VI^(O)_2_] remained in the post-reaction mixture, whereas all of the [(L1)Ru^VI^(O)_2_] had been consumed in the more efficient, water-saturated DCM reaction (Fig. S4b and c†) showing again the pronounced effect of water in facilitating catalytic turnover.

### Catalyst speciation and deactivation

**Fig. 4:**
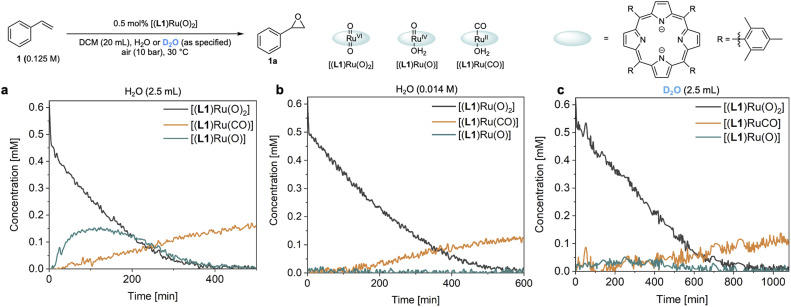
*Operando* catalyst speciation studies. (a) Distribution of ruthenium species observed by ^1^H FlowNMR spectroscopy in DCM saturated with an excess of H_2_O. (b) Distribution of ruthenium species observed by ^1^H FlowNMR spectroscopy in laboratory grade DCM (14 mM). (c) Distribution of ruthenium species observed by ^1^H FlowNMR spectroscopy in DCM saturated with an excess of D_2_O (99.9% atom% D).

The reaction progress data (Fig. 3) from the *operando* FlowNMR measurements showed a deviation from the initial zero order behaviour towards the end of the reaction (Fig. 3a–c), indicative of either inhibition or deactivation. Catalyst productivity and lifetime are important metrics for industrial application, but deactivation mechanisms are rarely investigated in academic studies.^37^ To gain insight into catalyst speciation and deactivation during turnover, ^1^H FlowNMR spectroscopy and orthogonal techniques (see below) were used to detect ruthenium intermediates during the reaction. By use of a high-sensitivity cryoprobe we were able to identify and monitor not only [(L1)Ru^VI^(O)_2_] (*δ* = 8.81 ppm) but also the formation of [(L1)Ru^II^(CO)(H_2_O)] (*δ* = 8.40 ppm) and the paramagnetic [(L1)Ru^IV^(O)(H_2_O)] (*δ* = −9.11 ppm) intermediates^38^ during the aerobic epoxidation of 1 under our optimised reaction conditions (Fig. S7†). As indicated by incomplete mass balances towards the end of the reaction not all Ru species were detectable by ^1^H NMR, but some important trends are reflected in the data recorded. As shown in Fig. 4, the amount of [(L1)Ru^VI^(O)_2_] decreased steadily over the course of the reaction, but about twice as quickly in the presence of H_2_O than with D_2_O or when anhydrous. The in-cycle mono-oxo complex [(L1)Ru^IV^(O)(H_2_O)] formed after oxygen atom transfer (OAT) to the substrate could only be detected transiently under the most efficient conditions of using water-saturated DCM. Both of these observations align with the faster catalytic rates observed under these conditions and point towards a shift in the turnover-limiting step of the catalytic cycle in the presence of water, as also shown by the rate law. The carbonyl complex [(L1)Ru^II^(CO)(H_2_O)] formed at a steady rate throughout the reaction to reach ∼20–25% of the total Ru loading after 8 hours as the only ruthenium species detectable by ^1^H NMR spectroscopy in the post-reaction mixture. As previously suggested^39^ and confirmed independently (Fig. S4d†), this carbonyl complex is inactive in the aerobic epoxidation and thus an irreversibly deactivated state of the catalyst.

**Fig. 5:**
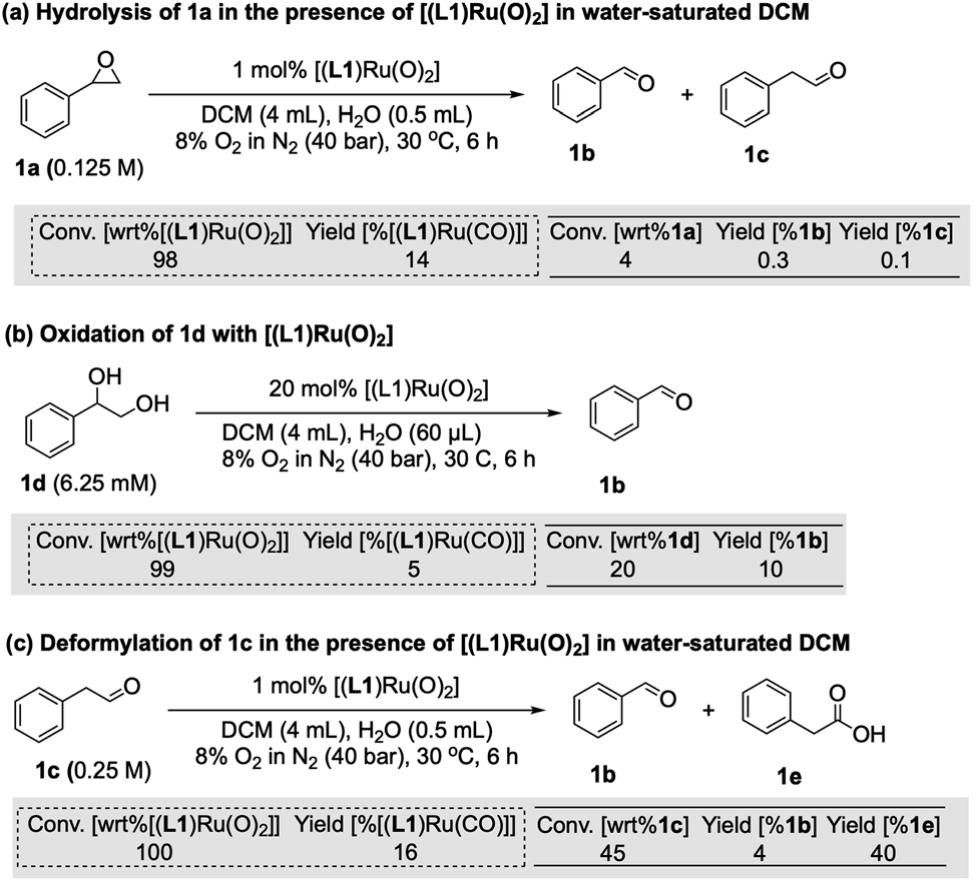
Investigation of the formation of [(L1)Ru(CO)] species during aerobic olefin epoxidation. Dashed boxes refer to conversion of starting Ru complex (wrt = “with regard to”) and yield of corresponding CO complex. Solid boxes refer to conversion of organic substrates and yields of corresponding products.

Since there are limited studies that explicitly consider deactivation pathways in this reaction, we carried out a series of experiments to better understand the formation of [(L1)Ru^II^(CO)] during catalysis. As shown in Fig. 3, in the oxidation of styrene (1) along with the desired epoxide (1a) benzaldehyde (1b) and phenylacetaldehyde (1c) were also formed. 1c may form *via* the isomerisation of 1a,^23^ whereas 1b contains one carbon atom less than the starting material. Although we have not detected any observable amounts of diols under our catalytic conditions it is known that epoxides may hydrolyse *in situ*, and control experiments confirmed that 1a could be slowly hydrolysed to 1-phenylethane-1,2-diol (1d, 8%) in water-saturated DCM at 30 °C (Fig. S17a†).^40^ When the diol 1d was added to [(L1)Ru^VI^(O)_2_] complete consumption of the complex and formation of [(L1)Ru^II^(CO)] was observed (Fig. 5a). Furthermore, using 20 mol% [(L1)Ru^VI^(O)_2_] to catalyse the aerobic oxidation of 1d led to the formation of 10% of 1b with 5% of [(L1)Ru^II^(CO)] (Fig. 5b).

It is also known that metal-peroxo or hydroperoxo complexes can catalyse the deformylation of aldehydes.^41^ A control experiment showed that 1c could react with [(L1)Ru^VI^(O)_2_] and lead to the formation of 1b (4%) and [(L1)Ru^II^(CO)] in 16% yield (Fig. 5c). When 2.5% of 1d was used as an additive in the aerobic epoxidation of 1 with [(L1)Ru^VI^(O)_2_], ^1^H FlowNMR data showed that product formation was greatly retarded in the first 100 min, but once all of 1d had been oxidised the rate of production of 1a increased (Fig. S17b†). This observation showed diols not to be a strong catalyst poison but to exhibit an inhibitory (or rather diverting) effect on the epoxidation. With 4% of 1c as an additive, the aerobic epoxidation of 1 led to a much faster formation of [(L1)Ru^II^(CO)] at the beginning of the reaction (Fig. S17c†). We thus conclude that the CO ligand in [(L1)Ru^II^(CO)] originates from both the deformylation of aldehyde 1c as well as oxidative C–C bond cleavage of small amounts of diol produced *in situ*, leading to the irreversible formation of inactive [(L1)Ru^II^(CO)] and benzaldehyde 1b.

**Fig. 6:**
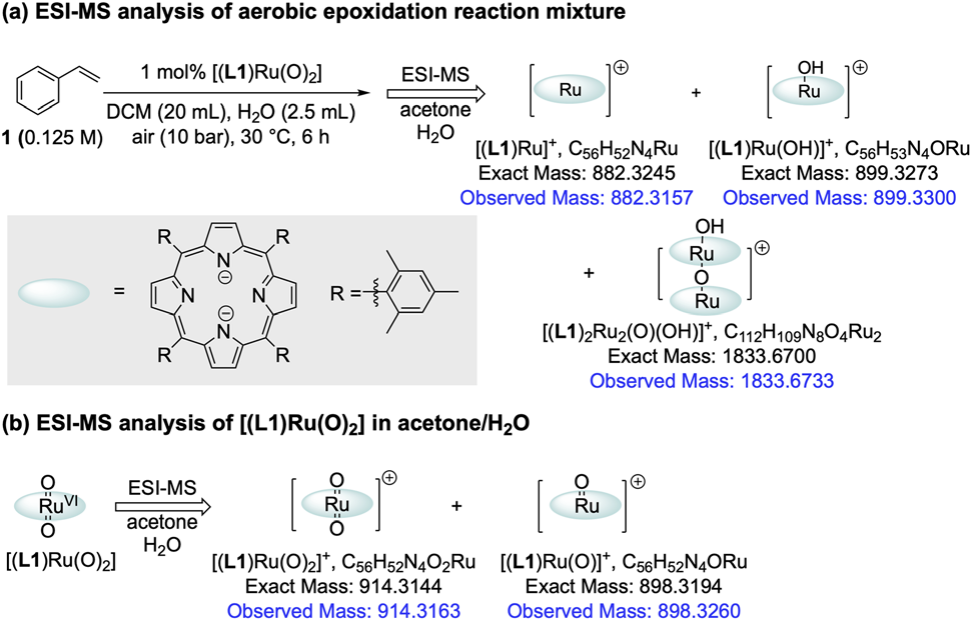
Investigation of catalyst deactivation during aerobic epoxidation using ESI-MS.

The amount of [(L1)Ru^II^(CO)] formed during the catalysis did not account for all of the activity loss, however, and a significant amount of the total Ru loading escaped the *operando*^1^H FlowNMR analyses towards the end of the reaction (Fig. 4a). We thus looked for other Ru species using UV-vis spectroscopy and high-resolution electrospray mass spectrometry (ESI-MS) during and after the aerobic epoxidation of 1 with [(L1)Ru^VI^(O)_2_]. The major species observed by ESI-MS was an ion at 899.3300 *m*/*z* corresponding to [(L1)Ru(OH)]^+^ and its solvent adducts as well as an envelope centred at 1833.6733 *m*/*z* corresponding to [(L1)_2_Ru_2_(O)(OH)]^+^ (Fig. 6a and S19†). Tandem MS/MS analysis showed this species to dissociate into [(L1)Ru(O)]^+^ and [(L1)Ru]^+^ upon targeted fragmentation (Fig. S20†). In contrast, only [(L1)Ru(O)_2_]^+^ and [(L1)Ru(O)]^+^ were observed when pure [(L1)Ru(O)_2_] was analysed by ESI.

MS under the same conditions (Fig. 6b and S21†), showing the reduced monomer and its dimer detected during the reaction to be generated by catalytic turnover. Several Ru-porphyrins have been reported to form μ-oxo dimers under oxidative conditions.^42^ Indeed, UV-Vis spectroscopic analysis of the post-reaction mixture showed weak but characteristic absorptions of such μ-oxo-Ru dimers around 600 nm (Fig. S23†),^42^ suggesting that even with the bulky tetramesityl-substituted porphyrin ligand L1 some association to inactive, NMR-silent dimers may occur during the catalysis in aqueous organic solvent.

### Isotopic labelling

**Fig. 7:**
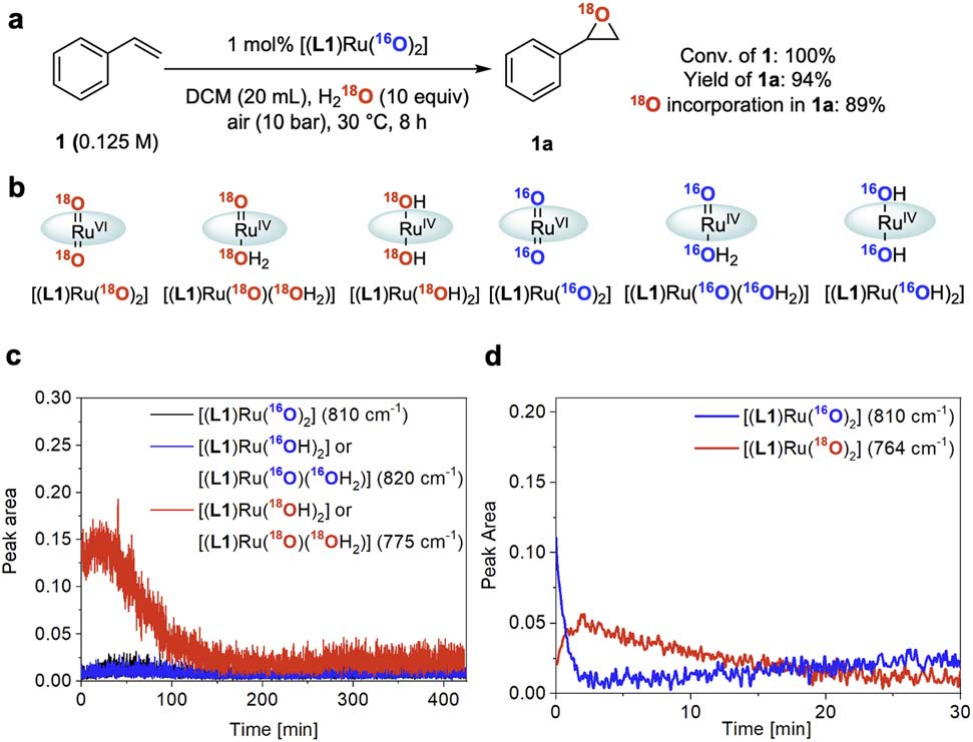
^18^O isotope labelling experiments. (a) ^18^O isotopic labelling experiment using ^18^OH_2_. Conversion and yield determined by quantitative ^1^H NMR spectroscopy, ^18^O incorporation in 1a determined by GC-MS. (b) ^18^O/^16^O isotope labelled ruthenium species observed by FlowRaman spectroscopy. (c) Profile of Ru species observed by FlowRaman spectroscopy (reaction conditions as in Fig. 7a). (d) Profile of Ru species observed by *in situ* Raman spectroscopy (see Fig. S14 in the ESI†).

To gain further insight into the role of water in the epoxidation catalysis we employed oxygen isotope labelling. When using 10 equiv. of ^18^OH_2_ relative to 1 under 10 bar of air containing ^16^O_2_, 1 was converted to 1a with 89 atom% ^18^O incorporation (Fig. 7a and S9†). In line with the kinetic relevance of water and the pronounced H/D KIE observed (see above), this finding further indicated that water is involved in a kinetically relevant step of the cycle to facilitate effective turnover. Similar data has previously been reported by Hirobe and co-workers^43^ and Ahn^36^ who studied the same catalyst. Katsuki and co-workers examined ^18^OH_2_ with their [(salen)Ru(NO)] catalyst and found lower levels of ^18^O incorporation, suggesting a different mechanism for their photo-assisted system.^19^*In situ* Raman spectroscopy of our reaction showed the doubly ^18^O-labelled mono-oxo complex [(L1)Ru^IV^(^18^O)(^18^OH_2_)] – or its *trans*-dihydroxy isomer [(L1)Ru^IV^(^18^OH)_2_] – forming from [(L1)Ru^VI^(^16^O)_2_] during turnover with ^18^OH_2_ and ^16^O_2_ (Fig. 7b and c; for details of the FlowRaman setup and band assignments see Supplementary Method D, Table S4 and Fig. S10–S15†). Thanks to a characteristic ^18/16^O isotope shift of 46 cm^−1^ in the ν(Ru=O) band we were able to follow the interconversion of [(L1)Ru^VI^(^16^O)_2_] into [(L1)Ru^VI^(^18^O)_2_] under these conditions (Fig. 7d), providing spectroscopic proof for dynamic oxygen exchange between high-valent ruthenium oxo complexes and water.

**Fig. 8:**
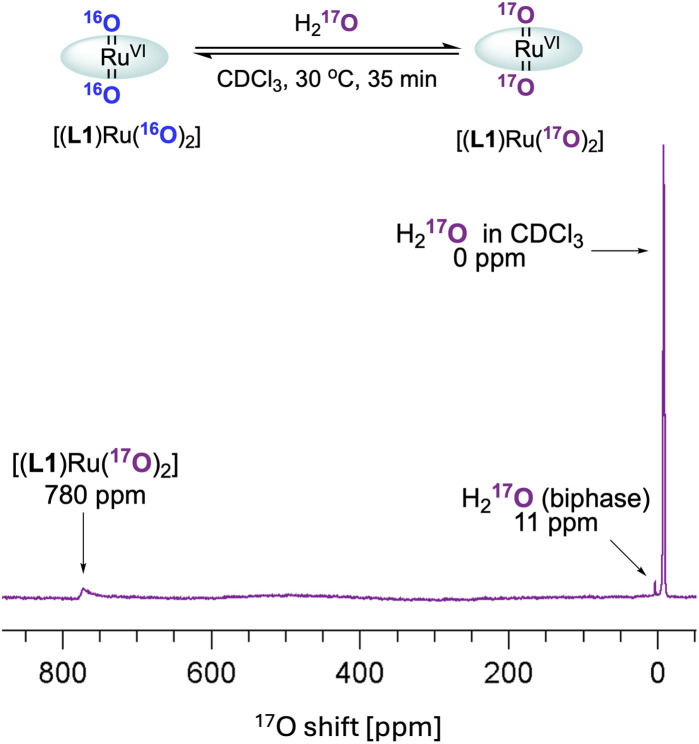
^17^O NMR spectrum of [(L1)Ru^VI^(^16^O)_2_] with ^17^OH_2_ in CDCl_3_.

These observations paired with superior catalytic activity in the presence of water made us wonder if the epoxidation may proceed *via* an active species other than [(L1)Ru^VI^(O)_2_], for example, a ruthenium hydroperoxide intermediate, with reactivity more typical of catalysis with H_2_O_2_.^9^ In water oxidation catalysis the interaction of high-valent metal oxo compounds with H_2_O is a key step in the formation of the O–O bond, and with high-valent Ru=O complexes in particular it is well recognised that water can form Ru–OOH intermediates *via* water nucleophilic attack (WNA) on an electrophilic oxo ligand.^44,45^ Such M–OOH intermediates are known to be powerful two-electron oxidants towards a range of substrates.^46–50^ In addition, the conversion of Fe^III^–OOH complexes to Fe^IV/V^ oxo species has been reported to occur *via* water assisted or Lewis acid activation pathways.^51^ It is also known that [(HOO)(HO)Mn^III^(por)] complexes can be generated from Mn^III^(por) with H_2_O_2_, which then act as precursors to [(O)_2_Mn^V^(por)] species under basic conditions.^52^ Looking for other ruthenium oxo species *in situ*, we thus exposed [(L1)Ru^VI^(^16^O)_2_] to ^17^OH_2_ (35–40 atom%) in dry CDCl_3_ and analysed the mixture by ^17^O NMR spectroscopy (supplementary method E†). After 30 min at 30 °C the formation of doubly exchanged [(L1)Ru^VI^(^17^O)_2_] was clearly observed in the ^17^O NMR spectrum at 780 ppm^53^ (Fig. 8) but no other ^17^O containing Ru species could be resolved. This result showed dynamic oxygen atom exchange between Ru^VI^=O and H_2_O to be possible even in the absence of catalytic turnover that generates reduced ruthenium complexes, without detecting any intermediates of this exchange reaction, however. Similar observations have previously been made by Ahn who also reported that exchange with ^18^OH_2_ was faster under catalytic conditions.^36^

### Computational studies

**Fig. 9:**
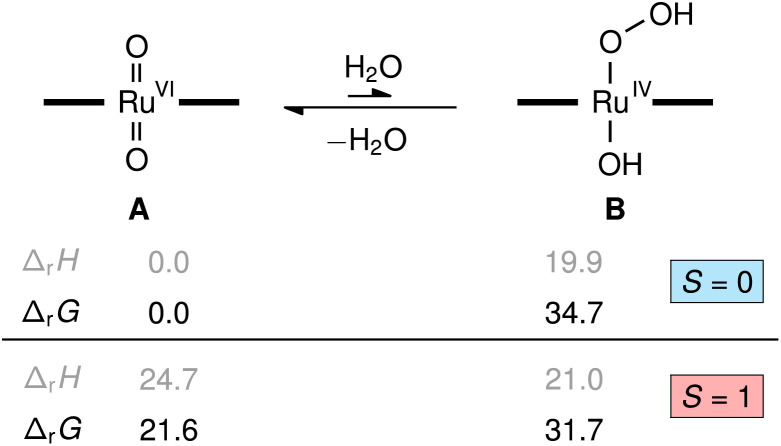
Relative enthalpies and Gibbs free energies in kcal mol^−1^ for the formation of [(L1)Ru^IV^(OH)(OOH)], B, from [(L1)Ru^VI^(O)_2_], A, and water computed at the M06L-D3(SMD)/def2-TZVP level (porphyrin ligand L1 illustrated with horizontal bars).

To understand the role of water in the reaction we investigated several possibilities with quantum chemical calculations at the density functional theory level (M06L/def2-TZVP). The reaction of [(L1)Ru^VI^(O)_2_] (A) with water to yield the transient hydroxo–hydroperoxo intermediate [(L1)Ru^IV^(OH)(OOH)] (B) shown in Fig. 9 was predicted to be energetically uphill (Δ_r_*H* = +19.9 kcal mol^−1^) and highly endergonic (Δ_r_*G* = +34.7 kcal mol^−1^). This thermochemical relation was further corroborated by coupled-cluster calculations (DLPNO-CCSD(T1)/CBS, see ESI† for details including the DFT-calculated reaction path from B to D, [(L1)Ru^IV^(O)]). Consistent with literature and our NMR spectroscopic findings, the dioxo complex A is a diamagnetic closed-shell singlet (*S* = 0) well-separated from its paramagnetic triplet electromer (*S* = 1) by Δ_S/T_*G* = +21.6 kcal mol^−1^, while the singlet and triplet states of the hydroxo-hydroperoxo derivatives are close in energy (Δ_S/T_*G* = −3.0 kcal mol^−1^). These results show the formation of a hydroperoxo complex to be too unfavourable for such an intermediate to play a significant role in olefin epoxidation catalysis with [(L1)Ru(O)_2_] at 30 °C.

**Fig. 10:**
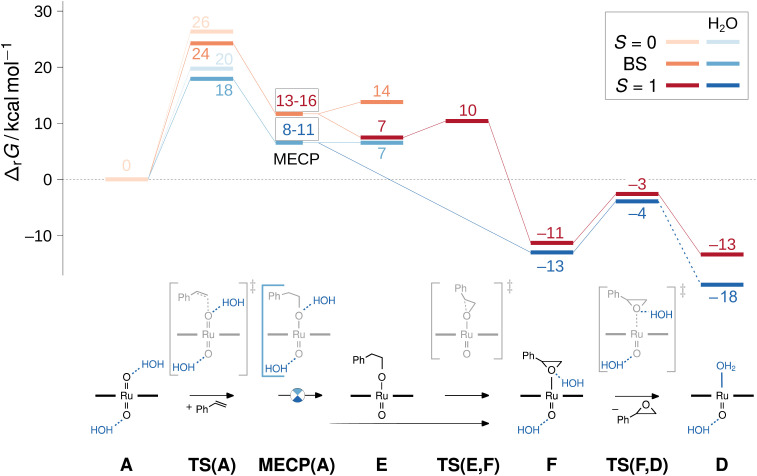
Gibbs free energy reaction profile for selected intermediates of the water-free and micro-solvated epoxidation path including two broken-symmetry singlet/triplet minimum energy cross points (MECP) and transition structures (TS) computed at the M06L-D3(SMD)/def2TZVP level. The lowest energy closed-shell singlets (*S* = 0) are shown in light orange and light blue, the broken-symmetry singlets (BS) in orange and blue, the triplet (*S* = 1) in dark red and dark blue. In the text, all species are labelled with capital letters with the multiplicity in superscript and the presence of two water molecules indicated with a ‘w2’ subscript.

However, as we and others have consistently found olefin epoxidation with [(L1)Ru^VI^(O)_2_] to be very slow in anhydrous organic solvents (even stoichiometrically),^15,36^ water must have an activating effect on the dioxo complex. If the closed-shell O=Ru^VI^=O is insufficiently electrophilic to react with an olefin (or indeed H_2_O) in non-polar media, we wondered if water perhaps activated the oxo through hydrogen bonding. Cenini and co-workers have reported NMR spectroscopic evidence for hydrogen bonding between water and some Ru(por)-dioxo complexes in organic solution,^54^ but this effect has not yet been considered in the context of OAT catalysis with such complexes. We therefore computationally examined whether such interactions could accelerate olefin epoxidation, an aspect that has not been considered in previous computational studies on Ru-porphyrin complexes for oxidation reactions.^55–58^ We thus compared the reaction coordinates from [(L1)Ru^VI^(O)_2_] and styrene to [(L1)Ru^IV^(O)] and epoxide in the absence and presence of specific water solvation in addition to the continuum solvent model applied for dichloromethane (Fig. 10).

Without H_2_O, the direct epoxidation of styrene by the singlet dioxo-Ru^VI^ complex ^1^A initially yields a triplet oxo-epoxide-Ru^IV^ complex ^3^F which is thermochemically accessible at Δ_r_*G* = −11 kcal mol^−1^ (red trace in Fig. 10). The net reaction thus involves spin crossing from the diamagnetic closed-shell ^1^A to the triplet spin surface,^59^ passing through a rate-limiting transition structure at 24 kcal mol^−1^ with an open-shell singlet electronic structure. In this broken-symmetry singlet TS, a π*(Ru=O) orbital is singly populated, effectively generating an oxyl radical for the initial C–O bond formation. A closed-shell singlet transition state as well as all subsequent intermediates are less stable as alternative electromers (see ESI†). Crossing from the broken-symmetry singlet TS onto the triplet product surface on the lowest free energy pathway involves a minimum energy crossing point (MECP)^60^ of 13–16 kcal mol^−1^ where the primary alkoxide is formed and one unpaired electron is fully transferred into the π system of the alkene. On the triplet energy surface, the resulting ruthenium–alkoxide complex ^3^E was found at 7 kcal mol^−1^. After passing a small barrier of 3 kcal mol^−1^ for the formation of the second C–O bond *en route* to the epoxide complex at −11 kcal mol^−1^, liberation of the epoxide product *via* a dissociation barrier of 8 kcal mol^−1^ leads to the coordinatively unsaturated triplet mono-oxo-Ru^IV^ complex [(L1)Ru(O)] (^3^D).

Commencing from a bis-aqua bound dioxo-Ru^VI^ complex ^1^A_w2_ lowers the broken-symmetry singlet transition state for the electrophilic attack of the olefin by 6 kcal mol^−1^ so that it is now located 18 kcal mol^−1^ above the reactants (blue trace in Fig. 10). The inclusion of two explicit water molecules as a model for micro-solvation^54^ is thus shown to significantly accelerate the reaction: whereas a barrier of 24 kcal mol^−1^ translates into a *t*_1/2_ ≈ 12 h, the reduced barrier of 18 kcal mol^−1^ in the bis-aqua ligated model corresponds to a half-life of less than two seconds. Spin states were not noticeably affected by the hydrogen bonding interactions and the respective closed-shell species were all higher in energy, as in the absence of water. A broken symmetry singlet-triplet ^BS/3^MECP_w2_ at 8–11 kcal mol^−1^ was found which was significantly more favourable than the 19–22 kcal mol^−1^ of the corresponding closed-shell singlet-triplet ^1/3^MECP_w2_ for the hydrated version of the reaction (see ESI†), and also lower than the ^BS/3^MECP at 13–16 kcal mol^−1^ found in the anhydrous path. This bis-aqua ligated ^BS/3^MECP_w2_ is a late structure on the potential energy surface (*i.e.* closer to C–O bond formation) and yields swift downhill access to the bis-aqua ligated oxo-epoxide-Ru^IV^ complex ^3^F_w2_ at −13 kcal mol^−1^ without passing through another intermediate. After liberation of the epoxide product and rebound of H_2_O, the water-bound triplet mono-oxo-Ru^IV^ complex ^3^D_w2_ is obtained with an overall Δ_r_*G* of −18 kcal mol^−1^. The presence of water thus not only accelerates the OAT to styrene but also stabilises the mono-oxo ruthenium intermediate, consistent with the experimentally observed catalyst speciation profiles (Fig. 4).

**Fig. 11:**
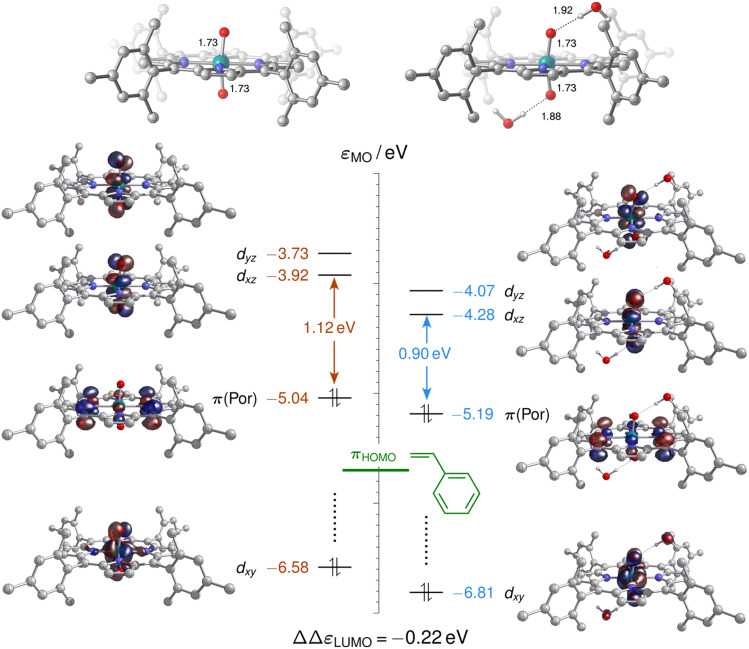
Molecular orbital energy diagram (eV) for [(L1)Ru^VI^(O)_2_], ^1^A (left), and [(L1)Ru^VI^(O)_2_]·(H_2_O)_2_, ^1^A_w2_ (right), showing selected frontier orbitals computed at the M06L-D3(SMD)/def2TZVP level. The relative stabilisation ΔΔ*ε*_LUMO_ of the lowest unoccupied molecular orbital (LUMO) is computed with reference to the corresponding highest occupied molecular orbital (HOMO), which is orthogonal to the O=Ru=O axis. ΔΔ*ε*_LUMO_ is equivalent to the difference of the HOMO/LUMO gaps, and the horizontal green bar at −5.7 eV indicates the HOMO energy level of styrene.

As neither the complex geometries nor their spin states were found to be affected by water hydrogen-bonding to the oxo groups of [(L1)Ru^VI^(O)_2_], the accelerating effect of micro-solvation on the OAT to styrene may be rationalised by the relative stabilisation of the LUMO which is involved in the electrophilic attack of the alkene. To compare relative LUMO energy changes, the HOMO is a suitable reference since it is fully localised on the porphyrin ligand and thus largely unperturbed by water ligation (Fig. 11). The difference in HOMO–LUMO energy gaps in the water-free and micro-solvated systems of ΔΔ*ε*_LUMO_ ≈ −0.2 eV can thus serve as a measure for the electronic influence of micro-solvation. In the micro-solvated case, the LUMO was found at −4.3 eV and thus closer in energy to the HOMO of styrene (−5.7 eV). As shown in the ESI,† the HOMO of the broken-symmetry singlet TS – result of the interaction of the ruthenium-dioxo LUMO and the HOMO of styrene – also benefits from water ligation by about 0.1 eV (Fig. S35†).

### Mechanistic interpretation and further optimisation

**Fig. 12:**
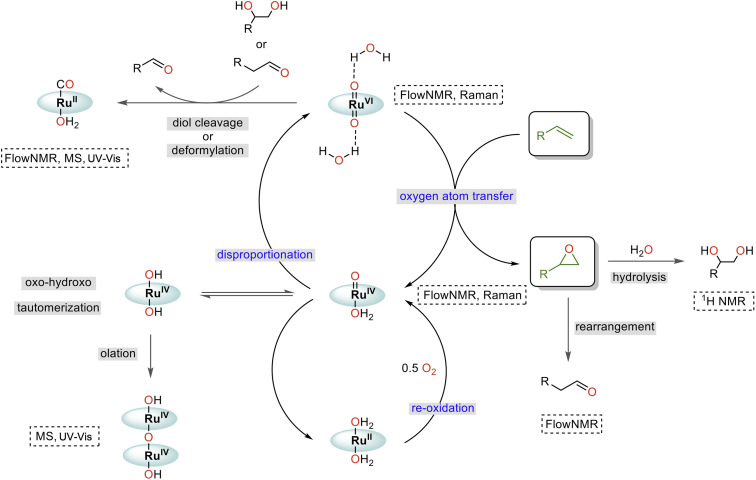
Proposed mechanism of the water-assisted, Ru-porphyrin catalysed aerobic epoxidation of alkenes.

Taking all our findings together with previous reports, we can suggest a mechanism for the water-promoted aerobic epoxidation of alkenes catalysed by oxo ruthenium porphyrin complexes (Fig. 12). The catalytic cycle consists of three main parts: oxygen atom transfer, disproportionation, and reoxidation.^57,61,62^ While we have been able to monitor the singlet Ru^VI^ di-oxo, the triplet Ru^IV^ mono-oxo and the singlet Ru^II^ carbonyl during turnover, the reduced catalyst state(s) reacting with O_2_ (presumably Ru^II^)^15,38,61,62^ have not been experimentally observed in *operando*. We also cannot fully rule out alternative pathways proceeding *via* Ru^III^ and Ru^V^ intermediates,^63^ noting that up to 2/3 of the [Ru] was in NMR-inactive states at the end of the catalysis. However, the high selectivity of the reaction and absence of autoxidation pathways (see substrate scope below) strongly indicate the catalytic flux to proceed *via* activated, closed-shell [(L1)Ru^VI^(O)_2_]·(H_2_O)_2_, and that NMR-silent species such as dimers or other paramagnetic complexes formed at later stages of the reaction to be deactivated states unrelated to the product-forming cycle (like the detected [(L1)Ru^II^(CO)]).

Comparing the reaction kinetics and catalyst speciation profiles in the absence and presence of water (Fig. 4) suggests the accelerating effect of water on substrate epoxidation by [(L1)Ru^VI^(O)_2_] to shift the turnover-limiting step of the cycle from oxygen-atom transfer under anhydrous conditions to catalyst reoxidation under the more efficient aqueous conditions. The first order reaction rate dependence in H_2_O, observation of extensive ^16/18^O scrambling, and the significant H/D kinetic isotope effect of 3.5 in water indicate rapid exchange of oxygen atoms with concomitant O–H bond breaking during the turnover-limiting disproportionation/reoxidation part of the water-assisted catalytic cycle.^36^ Although it is well established that O–H bond breaking is important in electron transfer mechanisms with Ru complexes,^30,64^ detailed studies have not been carried out for Ru-porphyrin systems. The closest precedent we are aware of is a study by Lau and co-workers who examined the oxidation of [(tmc)Ru^IV^(O)(solv)]^2+^ to [(tmc)Ru^VI^(O)_2_]^2+^ (where tmc = 1,4,8,11-tetramethyl-1,4,8,11-tetraazacyclotetradecane) by MnO_4_^−^ to find an H_2_O/D_2_O KIE of 3.5 which was ascribed to hydrogen atom transfer.^65^ Alternative pathways such as proton-coupled electron transfer are known to give rise to similar values,^64^ but the exact origin of the H-D KIE in our water-assisted aerobic epoxidation with Ru-porphyrins remains to be investigated.

Catalyst deactivation has been observed from both Ru^VI^ and Ru^IV^ intermediates, where [(L1)Ru^IV^(OH)_2_] may form from [(L1)Ru^IV^(O)(OH_2_)] through oxo-hydroxo tautomerisation^66–68^ and then dimerising by condensation (olation).^42^ Additional, thus far undetected deactivation mechanisms may also occur from the elusive reduced state that replenishes the Ru^IV^ mono-oxo intermediate by reaction with dioxygen. While the essentially irreversible formation of dimeric Ru^IV^–O–Ru^IV^ complexes merely drains active material from the cycle, the reaction of [(L1)Ru^VI^(O)_2_]·(H_2_O)_2_ with diols and aldehydes produces side products that diminish reaction selectivity and yield. The Lewis acid catalysed rearrangement of epoxides into aldehydes^69^ is thus particularly detrimental to this reaction, as is *in situ* epoxide hydrolysis.

We briefly investigated whether it is possible to suppress these deactivation pathways by further fine-tuning of the reaction conditions. Lowering the [Ru] concentration should slow down dimerisation, and pH control might suppress epoxide hydrolysis and facilitate proton shuttling^64^ in the reoxidation of the catalyst. Indeed, using 0.25 mol% [Ru] with a sodium acetate buffer (pH = 5.7) almost doubled the catalyst TON to 300 for the epoxidation of 1 (Fig. S24 and S25†). Further improvements are likely possible with more extensive reaction engineering.

### Substrate scope

**Fig. 13:**
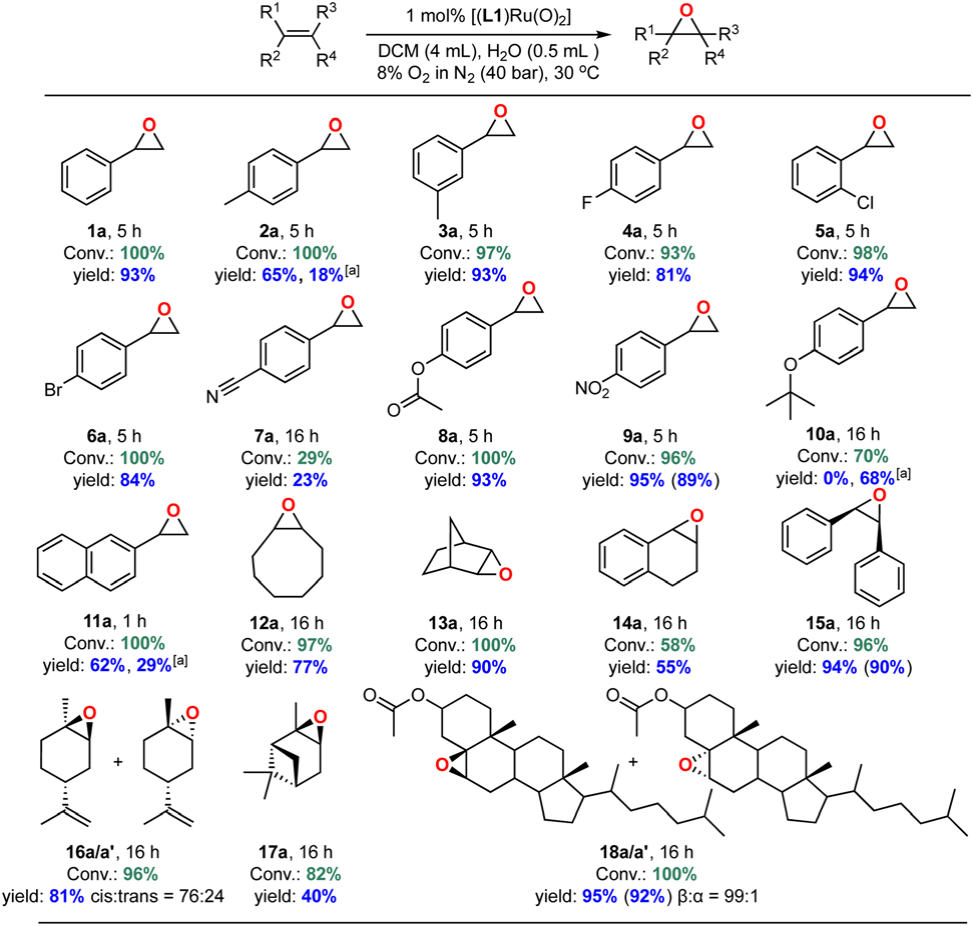
Substrate scope of [(L1)RuVI(O)_2_(H_2_O)_2_] catalysed aerobic alkene epoxidation. Conversions and yields determined by quantitative 1H NMR spectroscopy against mesitylene as internal standard; isolated yields provided in parentheses. [a] Yield of corresponding aldehyde from epoxide isomerisation.

With an efficient catalyst system for the mild and selective epoxidation of styrene in hand, we decided to explore its applicability to other unsaturated substrates, as limited substrate scopes have been reported in previous studies.^6^ Using 1 mol% [(L1)Ru^VI^(O)_2_] in either DCM or CDCl_3_/EtOAc mixtures with water added, we were pleased to find excellent functional group tolerance across a range of different substrates (Fig. 13 and S24†). Electron-withdrawing substituents such as halides, esters and NO_2_ were well tolerated (4a–6a, 8a, 9a), and even internal aliphatic alkenes were epoxidised in good to very good yields (12a–18a/a′). Strongly coordinating functionalities such as nitriles led to lower catalyst activity (an effect similar to that seen in the solvent screening), and the epoxide products of electron-rich systems such as 4-methyl-styrene, 4-*tert*-butoxystyrene, 2-vinylnaphthalene partially isomerised to the corresponding aldehydes *in situ* as also observed by others.^23^ Application to stereogenic terpene substrates such as d-limonene and α-pinene, whose epoxides are valuable building blocks for biorenewable polymers,^70,71^ afforded a 76 : 24 mixture of diastereomeric 1,2-limonene oxide (16a/a′) in 81% yield (with no 8,9-oxide formed) and up to 40% α-pinene oxide (17a) from a sterically challenging substrate. Oxidation of the large steroid cholesteryl acetate proceeded with full conversion to afford the corresponding epoxide (18a/a′) with a remarkable *β* : *α* stereoselectivity of 99 : 1, attributable to steric repulsion between the porphyrin ligand and the axial hydrogen atoms on C-3 and C-7 on the α-face of the substrate.^72^ Limitations were only met with terminal aliphatic alkenes such as 1-octene and highly sterically demanding substrates. For example, while the catalyst cleanly epoxidised *cis*-stilbene in high yield *trans*-stilbene was not a suitable substrate (Table S5†), as also found with other Ru-catalysed epoxidations using pyridine *N*-oxide as the oxidant.^73^ Importantly, the stereoselectivity found in products such as 18a showed that the oxidation is catalyst-controlled rather than proceeding *via* an autoxidation mechanism.^74^ This is further supported by the results of ^18^O labelling experiments (see above) and no ring-opening products formed during the oxidation of the radical clock substrate 1-cyclopropylvinyl-benzene (entry 6, Table S5†).^75,76^

## Conclusions

Water has been shown to co-catalyse the aerobic epoxidation of alkenes mediated by porphyrin ruthenium oxo complexes, a finding that gives rise to an order of magnitude improvement in activity over the previous five decades of research in homogeneous aerobic epoxidation catalysis. This protocol provides an efficient, clean and sustainable method for catalytic epoxide synthesis that does not operate *via* radical pathways such as autoxidation which typically governs aerobic systems. The reaction proceeds under mild conditions with gentle heating to 30 °C and operates within safe limiting oxygen concentrations (LOC) such as 8% O_2_ (ref. 77) in non-flammable solvent mixtures. From the different ligands investigated Groves' original TMP ligand emerged as the most efficient catalyst for this reaction, which under our optimised conditions reached a TON up to 300 with epoxide selectivity of >80%. A wide range of di- and tri-substituted aromatic and aliphatic alkenes were tolerated for the first time with our protocol, and high face-selectivity can be achieved with sterically demanding substrates due to the bulky TMP ligand. Mechanistically, the system elegantly fulfils the key criteria for an efficient aerobic oxidation catalyst, where the di-oxo complex selectively epoxidises the substrate and is effectively regenerated *via* disproportionation and reoxidation by O_2_ without the need for sacrificial co-reductants. The collective evidence from multiple *in situ* and *operando* analytics, isotope labelling experiments and computational analyses indicate that water activates [(L1)Ru^VI^(O)_2_] for OAT to the alkene by lowering its LUMO energy through hydrogen bonding such that catalyst reoxidation becomes turnover-limiting in the water-assisted system. We note that little is still known about this important step of the catalytic cycle where extensive oxygen atom scrambling and kinetically relevant O–H breaking occurs. We believe that a deeper understanding of the steps involved in the regeneration of the Ru^VI^ di-oxo complex would allow for further improvements. Our results represent another example of the importance of solvent effects and specific solvation in homogeneous oxidation catalysis,^78,79^ and we hope that future developments will be able to take advantage of our findings to improve catalyst activity further to apply them to challenges in asymmetric epoxidation, perhaps even the industrially important short-chain aliphatic α-olefins.

## Data availability

The datasets supporting this article have been uploaded as part of the ESI.†

## Author contributions

MJM and UH conceived the project, designed the methodology and jointly supervised the project. QC and CM performed synthetic and catalytic experiments, and QC carried out all spectroscopic analyses. MD carried out all the DFT calculations. MD and VK analysed the DFT data. QC, MD, VK, MJM and UH analysed the data and wrote the manuscript.

## Conflicts of interest

There are no conflicts to declare.

## Supplementary Material

SC-015-D3SC05516G-s001

## References

[cit1] Armanino N., Charpentier J., Flachsmann F., Goeke A., Liniger M., Kraft P. (2020). Angew. Chem., Int. Ed..

[cit2] Gorzynski Smith J. (1984). Synthesis.

[cit3] Glatt H., Jung R., Oesch F. (1983). Mutat. Res..

[cit4] Gomes A. R., Varela C. L., Tavares-da-Silva E. J., Roleira F. M. F. (2020). Eur. J. Med. Chem..

[cit5] Sienel G. , Rieth R. and Rowbottom K. T., in Ullmann's Encyclopedia of Industrial Chemistry, 10.1002/14356007.a09_531

[cit6] Petrosyan A., Hauptmann R., Pospech J. (2018). Eur. J. Org Chem..

[cit7] Batra M. S., Dwivedi R., Prasad R. (2019). ChemistrySelect.

[cit8] Hauser S. A., Cokoja M., Kühn F. E. (2013). Catal. Sci. Technol..

[cit9] Lane B. S., Burgess K. (2003). Chem. Rev..

[cit10] Punniyamurthy T., Velusamy S., Iqbal J. (2005). Chem. Rev..

[cit11] Costas M. , in Green Oxidation in Organic Synthesis, 2019, pp. 123–157, 10.1002/9781119304197.ch4

[cit12] Pereira M. M., Dias L. D., Calvete M. J. F. (2018). ACS Catal..

[cit13] Bryliakov K. P. (2017). Chem. Rev..

[cit14] Singh K. K., Sen Gupta S. (2017). Chem. Commun..

[cit15] Groves J. T., Quinn R. (1985). J. Am. Chem. Soc..

[cit16] Leung W.-H., Che C.-M., Yeung C.-H., Poo C.-K. (1993). Polyhedron.

[cit17] Lai T.-S., Zhang R., Cheung K.-K., Che C.-M., Lai T.-S., Kwong H.-L. (1998). Chem. Commun..

[cit18] Koya S., Nishioka Y., Mizoguchi H., Uchida T., Katsuki T. (2012). Angew. Chem., Int. Ed..

[cit19] Tanaka H., Nishikawa H., Uchida T., Katsuki T. (2010). J. Am. Chem. Soc..

[cit20] Goldstein A. S., Beer R. H., Drago R. S. (1994). J. Am. Chem. Soc..

[cit21] Shing K.-P., Wan Q., Chang X.-Y., Che C.-M. (2020). Chem. Commun..

[cit22] Vanover E., Huang Y., Xu L., Newcomb M., Zhang R. (2010). Org. Lett..

[cit23] Jiang G., Chen J., Thu H. Y., Huang J. S., Zhu N., Che C. M. (2008). Angew. Chem., Int. Ed..

[cit24] Zhang L. L., Wang X. Y., Jiang K. Y., Zhao B. Y., Yan H. M., Zhang X. Y., Zhang Z. X., Guo Z., Che C. M. (2018). Dalton Trans..

[cit25] Lane B. S., Burgess K. (2001). J. Am. Chem. Soc..

[cit26] Tsugawa T., Furutachi H., Marunaka M., Endo T., Hashimoto K., Fujinami S., Akine S., Sakata Y., Nagatomo S., Tosha T., Nomura T., Kitagawa T., Ogura T., Suzuki M. (2015). Chem. Lett..

[cit27] Ma W., Qiao Y., Theyssen N., Zhou Q., Li D., Ding B., Wang D., Hou Z. (2019). Catal. Sci. Technol..

[cit28] Merriman R. W. (1913). J. Chem. Soc., Trans..

[cit29] IUPAC-NIST Solubility Database, https://srdata.nist.gov/solubility/, accessed 23rd December, 2023

[cit30] Ishizuka T., Kotani H., Kojima T. (2016). Dalton Trans..

[cit31] Mallat T., Baiker A. (2011). Catal. Sci. Technol..

[cit32] Hall A. M. R., Chouler J. C., Codina A., Gierth P. T., Lowe J. P., Hintermair U. (2016). Catal. Sci. Technol..

[cit33] Blackmond D. G. (2005). Angew. Chem., Int. Ed..

[cit34] Burés J. (2016). Angew. Chem., Int. Ed..

[cit35] Ho C., Leung W.-H., Che C.-M. (1991). J. Chem. Soc., Dalton Trans..

[cit36] Ahn K.-H. , Ph.D thesis, Princeton University, 1988

[cit37] Crabtree R. H. (2015). Chem. Rev..

[cit38] Groves J. T., Ahn K. H. (1987). Inorg. Chem..

[cit39] Scharbert B., Zeisberger E., Paulus E. (1995). J. Organomet. Chem..

[cit40] Wang Z., Cui Y.-T., Xu Z.-B., Qu J. (2008). J. Org. Chem..

[cit41] Bagha U. K., Satpathy J. K., Mukherjee G., Sastri C. V., de Visser S. P. (2021). Org. Biomol. Chem..

[cit42] Leung W. H., Che C. M. (1989). J. Am. Chem. Soc..

[cit43] Ohtake H., Higuchi T., Hirobe M. (1992). Tetrahedron Lett..

[cit44] Shaffer D. W., Xie Y., Concepcion J. J. (2017). Chem. Soc. Rev..

[cit45] Matheu R., Garrido-Barros P., Gil-Sepulcre M., Ertem M. Z., Sala X., Gimbert-Suriñach C., Llobet A. (2019). Nat. Rev. Chem.

[cit46] Conley N. R., Labios L. A., Pearson D. M., McCrory C. C. L., Waymouth R. M. (2007). Organometallics.

[cit47] Cho J., Jeon S., Wilson S. A., Liu L. V., Kang E. A., Braymer J. J., Lim M. H., Hedman B., Hodgson K. O., Valentine J. S., Solomon E. I., Nam W. (2011). Nature.

[cit48] Shin B., Sutherlin K. D., Ohta T., Ogura T., Solomon E. I., Cho J. (2016). Inorg. Chem..

[cit49] Hölzl S. M., Altmann P. J., Kück J. W., Kühn F. E. (2017). Coord. Chem. Rev..

[cit50] Walker K. L., Dornan L. M., Zare R. N., Waymouth R. M., Muldoon M. J. (2017). J. Am. Chem. Soc..

[cit51] Kal S., Xu S., Que Jr L. (2020). Angew. Chem., Int. Ed..

[cit52] Jin N., Lahaye D. E., Groves J. T. (2010). Inorg. Chem..

[cit53] Fujii H., Kurahashi T., Tosha T., Yoshimura T., Kitagawa T. (2006). J. Inorg. Biochem..

[cit54] Gallo E., Caselli A., Ragaini F., Fantauzzi S., Masciocchi N., Sironi A., Cenini S. (2005). Inorg. Chem..

[cit55] Ishikawa A., Sakaki S. (2011). J. Phys. Chem. A.

[cit56] Shing K.-P., Cao B., Liu Y., Lee H. K., Li M.-D., Phillips D. L., Chang X.-Y., Che C.-M. (2018). J. Am. Chem. Soc..

[cit57] Zierkiewicz W., Privalov T. (2006). Dalton Trans..

[cit58] Funyu S., Kinai M., Masui D., Takagi S., Shimada T., Tachibana H., Inoue H. (2010). Photochem. Photobiol. Sci..

[cit59] This is analogous to the epoxidation reaction with the tetra-(pentafluorophenyl) substituted congener [(L3)Ru(O)_2_] reported in ref. 24

[cit60] Popp B. V., Stahl S. S. (2009). Chem.–Euro. J..

[cit61] Collman J. P., Barnes C. E., Collins T. J., Brothers P. J., Gallucci J., Ibers J. A. (1981). J. Am. Chem. Soc..

[cit62] Farrell N., Dolphin D. H., James B. R. (1978). J. Am. Chem. Soc..

[cit63] Groves J. T., Bonchio M., Carofiglio T., Shalyaev K. (1996). J. Am. Chem. Soc..

[cit64] Huynh M. H. V., Meyer T. J. (2007). Chem. Rev..

[cit65] Lam W. W. Y., Man W.-L., Leung C.-F., Wong C.-Y., Lau T.-C. (2007). J. Am. Chem. Soc..

[cit66] Lee K. A., Nam W. (1997). J. Am. Chem. Soc..

[cit67] Bernadou J., Meunier B. (1998). Chem. Commun..

[cit68] Seo M. S., In J.-H., Kim S. O., Oh N. Y., Hong J., Kim J., Que Jr L., Nam W. (2004). Angew. Chem., Int. Ed..

[cit69] Gouygou M. and Urrutigoïty M., in Comprehensive Organic Synthesis, ed. P. Knochel, Elsevier, Amsterdam, 2nd edn, 2014, pp. 757–794, 10.1016/B978-0-08-097742-3.00320-7

[cit70] Poland S. J., Darensbourg D. J. (2017). Green Chem..

[cit71] Maltby K. A., Hutchby M., Plucinski P., Davidson M. G., Hintermair U. (2020). Chem.–Euro. J..

[cit72] Tavarès M., Ramasseul R., Marchon J.-C., Bachet B., Brassy C., Mornon J.-P. (1992). J. Chem. Soc., Perkin Trans. 2 (1972-1999).

[cit73] Higuchi T., Ohtake H., Hirobe M. (1989). Tetrahedron Lett..

[cit74] Liu X., Ryabenkova Y., Conte M. (2015). Phys. Chem. Chem. Phys..

[cit75] Kristensen S. K., Laursen S. L. R., Taarning E., Skrydstrup T. (2018). Angew. Chem., Int. Ed..

[cit76] Triandafillidi I., Kokotou M. G., Lotter D., Sparr C., Kokotos C. G. (2021). Chem. Sci..

[cit77] Osterberg P. M., Niemeier J. K., Welch C. J., Hawkins J. M., Martinelli J. R., Johnson T. E., Root T. W., Stahl S. S. (2015). Org. Process Res. Dev..

[cit78] Fujisaki H., Ishizuka T., Kotani H., Shiota Y., Yoshizawa K., Kojima T. (2023). Nature.

[cit79] Goldsmith B. R., Hwang T., Seritan S., Peters B., Scott S. L. (2015). J. Am. Chem. Soc..

