# Supplementary material for: Water co-catalysis in aerobic olefin epoxidation mediated by ruthenium oxo complexes
Source: Chem Sci. 2024 Jan 9;15(9):3104–15. doi: 10.1039/d3sc05516g (PMC10901482; doi:10.1039/d3sc05516g)
Supplement: SC-015-D3SC05516G-s001 [file SC-015-D3SC05516G-s001.pdf]

Electronic Supplementary Material (ESI) for Chemical Science.
This journal is © The Royal Society of Chemistry 2024

Supporting Information

Water as a Co-catalyst in Aerobic Olefin Epoxidation Mediated by Ruthenium Oxo Complexes

Qun Cao,[a][b]† Martin Diefenbach,[c] Calum Maguire,[a] Vera Krewald,* [c] Mark J. Muldoon*[a] and Ulrich Hintermair*[b]

[a] School of Chemistry and Chemical Engineering, Queen's University Belfast, Northern Ireland, UK

[b] Dynamic Reaction Monitoring Facility and Institute for Sustainability, University of Bath, UK

[c] Theoretical Chemistry, Department of Chemistry, Technische Universität Darmstadt, 64287, Darmstadt, Germany

Email: krewald@chemie.tu-darmstadt.de; m.j.muldoon@qub,.ac.uk; u.hintermair@bath.ac.uk

† Qun Cao is now at the School of Chemistry, University of Leicester, UK

Table of Contents

1. **General Information**……………………………………………………………........……………………..S3

2. **Experimental Details**...……………………..S4

   2.1. **Supplementary Method A**: Procedure for the optimization of aerobic alkene epoxidation catalyzed by dioxo-Ru(porphyrin) catalyst……………………..……………………………..………………………....S4

   2.2. **Supplementary Method B**: General procedure for the synthesis of various epoxides with [(**L1**)Ru(O)$_2$]………....………………………………………………………………. ……………………..S5

   2.3. **Supplementary Method C**: Procedure for aerobic styrene epoxidation catalyzed by [(**L1**)Ru(O)$_2$] monitored by online FlowNMR spectroscopy……………………………………….…………………..S9

   2.4. **Supplementary Method D**: FlowRaman Spectroscopy and band assignment.………….…….…….S15

   2.5. **Supplementary Method E**: ^{17}O NMR analysis………………………..…......…………………….…S20

3. **Characterization data of (1-cyclopropylvinyl)benzene, 2-cyclopropyl-2-phenylacetaldehyde, porphyrins and Ru complexes**…………………………………………………………………………………….…..S30

4. **Products Characterization Data**.……………….…………………………..S48

5. **ESI-MS data of Ru complexes and their simulated spectra**…….……………….................................S50

6. **NMR Data**…...…………………..S54

7. **Quantum-Chemical Studies**……………………………………………………………………….….….S83

   7.1. **Computational Methods**……………………………………………………………………………….…S83

   7.2. **Formation of transient hydroxo-hydroperoxo intermediate [(L1)Ru(OH)(OOH)]**………….....……..S84

   7.3. **Direct epoxidation via oxygen atom transfer**………………………………………………………........S88

   7.4. **Cartesian Coordinates of optimized geometries**……………………………………………………….S96

      7.4.1. **Species pertinent to formation of hydroxo-hydroperoxo intermediate**…………………………S96

      7.4.2. **Further species related to anhydrous path**……………………..…………………………...S106

      7.4.3. **Species related to microsolvation path**…………………………………………………………..S137

8. **References**...……………………S166

1 General Information

Unless stated otherwise, all reagents were purchased from commercial sources and used without further purification. Thin layer chromatography (TLC) was carried out using Merck TLC silica gel 60 sheet and visualized with ultraviolet light (254/365 nm) or potassium permanganate stain. Flash column chromatography (FCC) was performed with Sigma Aldrich silica gel 40-60 Å as the stationary phase and solvents employed were analytical grade. The air cylinder and pre-mixed O_2/N_2 (8:92) (B standard) and O_2/CO_2 (8:92) (B standard) cylinders were supplied by BOC Specialty Gases.

Static ^{1}H NMR spectra were recorded on a Bruker AVX400 (400 MHz) and Bruker AVX500 (500 MHz) spectrometer at 25 ^{o}C. Static ^{13}C NMR spectra were recorded on a Bruker AVX400 (101 MHz) and Bruker AVX500 (126 MHz) spectrometer at 25 ^{o}C. Static ^{19}F NMR spectra were recorded on a Bruker AVX500 (470 MHz) spectrometer at 25 ^{o}C. Online FlowNMR and ^{17}O NMR spectroscopy were recorded on Bruker 500 MHz Advance II+ Ultrashield equipped with a nitrogen-cooled BBO Prodigy CryoProbe, for more details see Supplementary Method C and D, respectively.

High resolution mass spectroscopy (HRMS) data was obtained on a Bruker Daltonics-micrOTOF-Q using positive electrospray (ES^+). UV-vis spectra were recorded on a Cary 60 UV-Vis and an AvaSpec-ULS2048CL-EVO with Avalight-DH-S-BAL light source. For more details of FlowRaman spectroscopy see Supplementary Method D. Water concentration was determined by Mettler-Toledo DL32 Karl Fischer titrator.

Gas chromatography analysis was carried out using Shimadzu GC-MS QP2010 ultra with SGE BP20(Wax) GC capillary column (66.7 m × 250 μm × 0.50 μm) was employed for all the separations using the following conditions: initial column temperature 40 °C; initial hold time 1 min; next temperature 100 °C; hold time 2 min; temperature ramp 3 °C/min, final temperature 250 °C; hold time 5 min; temperature ramp 10 °C/min; injection temperature 250 °C; injection volume 1 μL; split ratio 10; column pressure 280 kPa; column flow rate 2.73 mL/min; The effluent was combusted in an H_2/air flame and detected using FID (flame ionization detector) in an Argon shield, or sent to an electron ionization MS detector (ion source temperature: 200 °C, interface temperature: 250 °C).

2 Experimental Details

Supplementary Method A: Procedure for the optimization of aerobic alkene epoxidation catalyzed by dioxo-Ru(porphyrin) catalyst.

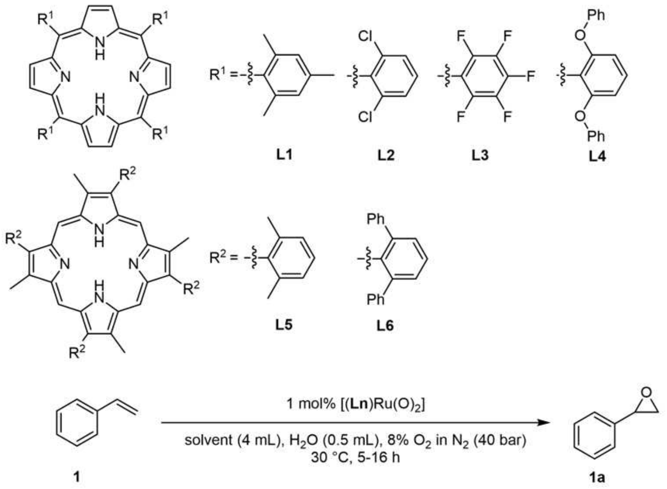

Figure S1. Optimization of aerobic alkene epoxidation catalyzed by dioxo-Ru(porphyrin) catalysts.

Reactions were carried out in 20 mL high-pressure reactors made of stainless steel or Hastelloy C276 and the reaction mixture was placed in a glass liner along with a teflon-coated magnetic stirrer bar. Into a glass liner, (porphyrin)Ru-dioxo complex ([(**Ln**)Ru(O)$_2$], ~0.0050 g, 0.005 mmol, 1 mol%), and internal standard mesitylene (0.0500 g), a magnetic stir bar in 4 mL of organic solvent and H_2O (0.5 mL) were added. After the styrene (**1**, 0.0520 g, 0.5 mmol) was added, the glass liner was then placed inside the reactor, which was then sealed and pressurized to 40 bar with 8% O_2 in N_2 gas. The reactor was then stirred in a heating block (on a hotplate stirrer) at 30 °C for 5 -16 hours. Reactions were sampled after the reactor body was cooled in an ice bath and slowly depressurized. The collected sample was analyzed by quantitative ^{1}H NMR spectroscopy.

Note: Styrene should only be used after passing through short basic Al_2O_3 plug in order to remove any stabilizers.

Supplementary Method B: General procedure for the synthesis of various epoxides with [(L1)Ru(O)$_2$]

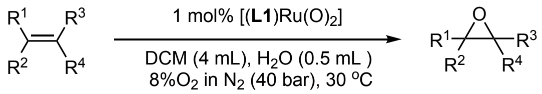

Figure S2. Synthesis of various epoxides by [(**L1**)Ru(O)$_2$] catalyzed aerobic epoxidation.

Reaction was carried out following Supplementary Method A. To obtain isolated yield of the desired epoxide, the organic phase of the reaction was dried over $MgSO_4$ and purified using flash chromatography with the noted solvent systems. Then the product containing fractions were combined and concentrated under reduced pressure.

Note: All alkene substrates should only be used after passing through short basic Al_2O_3 (in order to remove stabilizers).

Table S1. Effect of solvent on [(L1)Ru(O)$_2$] catalyzed aerobic epoxidation

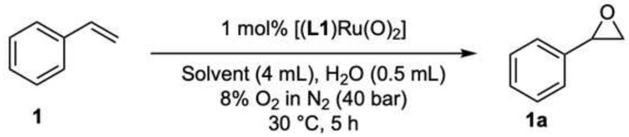

Entry	Solvent	Conv. [%][a][b]	Yield [%][a][b]
1	$CDCl_3$	77 (80)	72 (72)
2	1,2-dichloroethane	87 (89)	84 (83)
3	toluene	37 (41)	35 (36)
4	PhCl	43 (45)	24 (23)
5	$PhCF_3$	37 (40)	36 (39)
6	DCM	100	93
7	EtOAc	23 (61)	30 (52)
8	acetone	15	12
9	EtOH	0	0
10	MeOH	0	0
11	MeCN	0	0
12[c]	$CHCl_3$	0	0
13[d]	$CDCl_3$:EtOAc = 15:1	53 (58)	46 (42)
14[d]	$CDCl_3$:EtOAc = 7:1	63 (80)	61 (70)
15[d]	$CDCl_3$:EtOAc = 3:1	40 (83)	37 (64)
16	$CDCl_3$:EtOAc = 7:1	100	96
17	$PhCF_3$:EtOAc = 7:1	89	73

[a] Conversion and yield were determined by ^{1}H NMR using mesitylene as internal standard.
[b] Conversion and yield of reaction after 16 h were recorded in parathesis.
[c] Contain 0.5-1% EtOH as stabilizer
[d] 0.5 mol% [(**L1**)Ru(O)$_2$].

Table S2. Effect of additive on [(L1)Ru(O)₂] catalyzed aerobic epoxidation

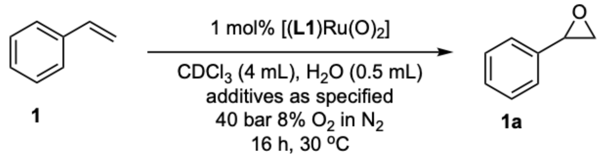

Entry	Additives	Conv. [%][a]	Yield [%][a]
1[b]	N/A	23	16
2	N/A	80	72
3	NaCl	75	70
4	10 mol% TBA^+F^-	49	37
5	10 mol% TBA^+Cl^-	44	32
6	10 mol% TBA^+Br^-	37	35
7	10 mol% TBA^+I^-	53	45
8	10 mol% $TBA^+(OH)^-$	7	1
9	10 mol% TBA^+ $(OAc)^-$	70	61
10	10 mol% DMA	97	71
11	10 mol% NMI	83	65
12	4 mol% HOAc	51	41

[a] Yield and conversion were calculated using ^1H NMR with mesitylene as internal standard.
[b] Without the addition of H_2O, dry $CDCl_3$ with 60 ppm of water.
TBA^+: tetrabutylammonium cation, DMA: dimethylacetamide, NMI: 1-methylimidazole

Table S3. Effect of gas pressure and composition on [(L1)Ru(O)₂] catalyzed aerobic epoxidation

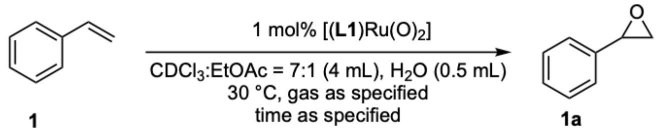

Entry	Gas mixture	Pressure [bar]	Time [h]	Conv [%][a]	Yield [%][a]
1	air	ambient	1.5	12	12
			3	24	23
2	8% O_2 in N_2	10	1.5	34	34
			3	72	70
3	air	10	1.5	40	38
			3	84	81
4	8% O_2 in N_2	40	1.5	41	39
			3	85	81
5	air	40	1.5	41	39
			3	83	80

[a] Conversion and yield were determined by ^1H NMR using mesitylene as internal standard.

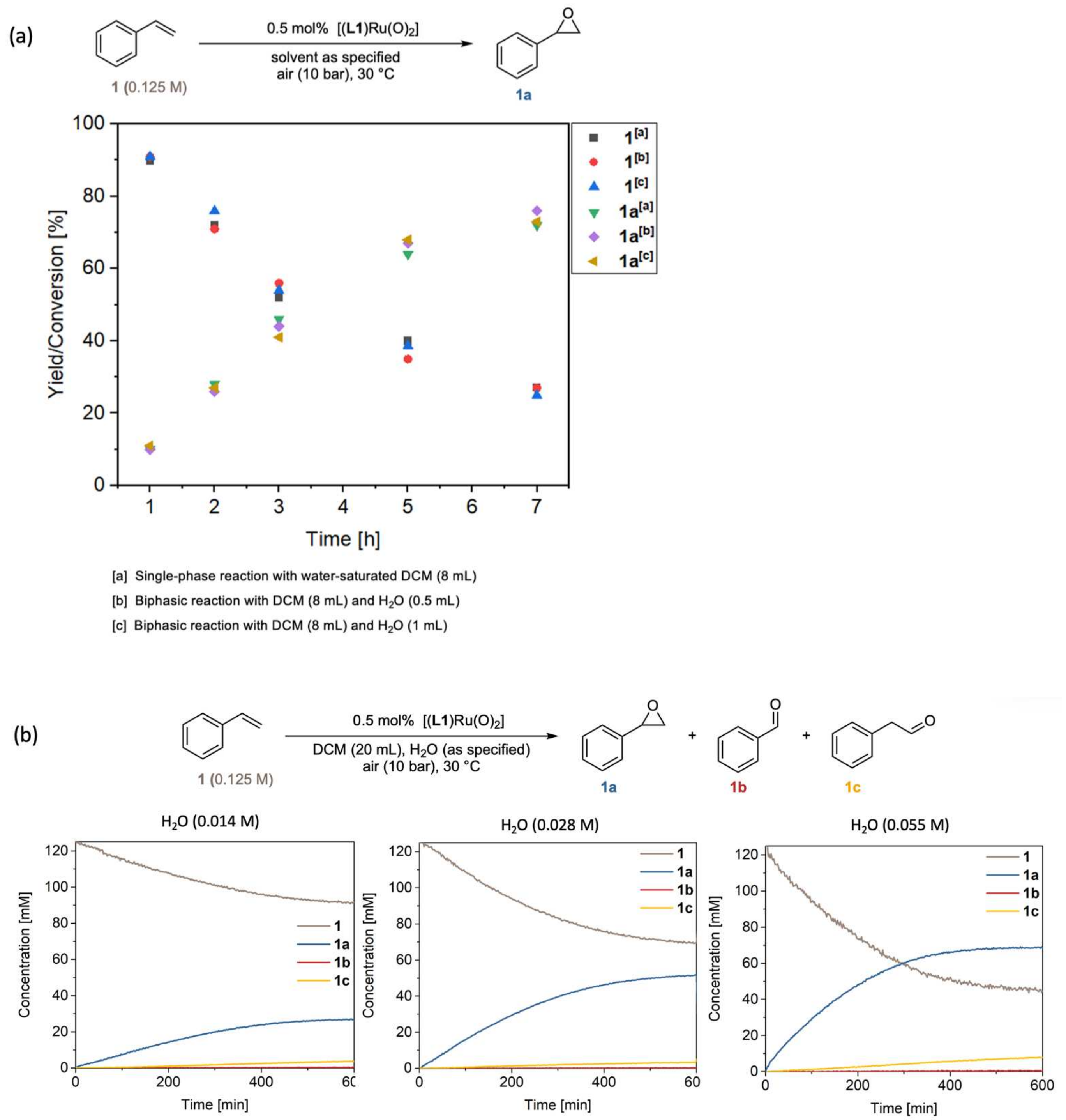

Figure S3. (a) Reaction profile of biphasic and single-phase [(**L1**)Ru(O)$_2$] catalyzed aerobic epoxidation of **1**. Conversion and yield were determined by ^{1}H NMR with mesitylene as internal standard. (b) Reaction progress in DCM with various amount of water.

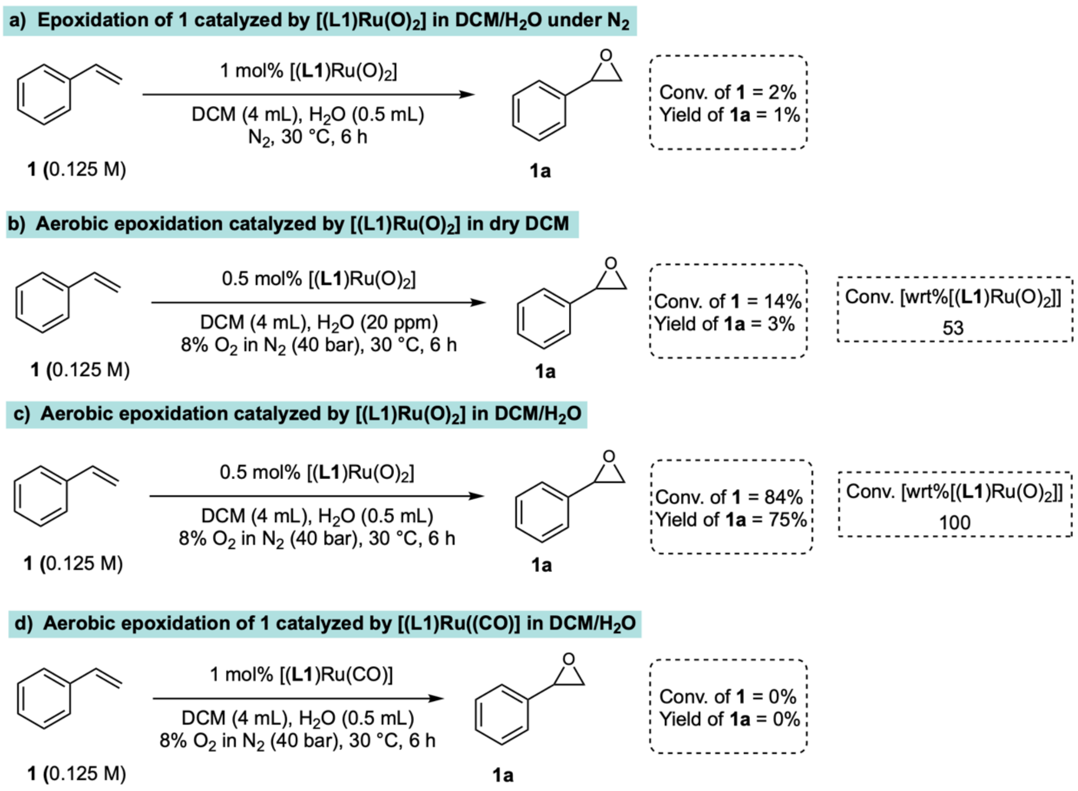

Figure S4. Control experiments for the aerobic epoxidation of styrene. Conversion and yield were measured by ^1H NMR with mesitylene as internal standard.

Note: As it is difficult to ensure strictly anhydrous conditions for the aerobic epoxidation in a flow setup that has previously been used with aqueous mixtures,[1] a control experiment with dry DCM (20 ppm of water after being dried with 3Å molecular sieves) was carried out under batch conditions (Figure S9b).

Supplementary Method C: Procedure for aerobic styrene epoxidation catalyzed by [(L1)Ru(O)$_2$] monitored by online FlowNMR spectroscopy

The aerobic epoxidation of styrene was caried out in a 100 mL thick-walled glass Büchi Miniclave pressure reactor made of glass with a stainless-steel lid connected to the flow NMR apparatus *via* 1/16” Swagelok connections. A micro-annular gear pump (mzr-6355 from HNP Mikrosysteme GmbH) was used to circulate the reaction mixture through the polytetrafluoroethylene tubing (PTFE, O.D. 1/16”, I.D. 0.75 mm, Upchurch Scientific) connected to a Teflon InsightMR flow tube (O.D. 1/32’’, I.D. 0.51 mm, Bruker) with 5 mm glass tip placed in the probe of the spectrometer (Figure S5). The inner volume the flow system was approximately 6 mL. Under reaction conditions, the flowrate was set to 4 mL/min with a sample delay time of 45 seconds. NMR spectra were recorded on a Bruker 500 MHz Advance II$^+$ Ultrashield equipped with a nitrogen-cooled BBO Prodigy CryoProbe. ^{1}H NMR chemical shifts are referenced against DCM solvent (δ 5.30). The reaction monitoring software used was InsightMR, and data processing was performed with TopSpin 4.0.6.

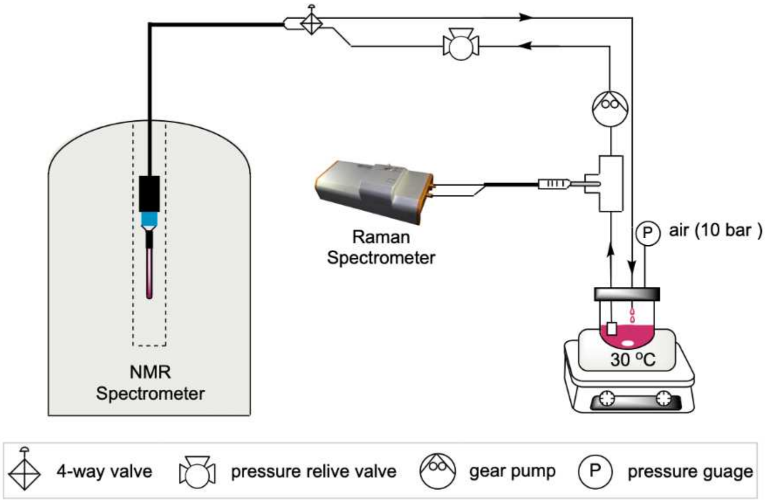

Figure S5. Experimental setup for on-line FlowNMR and FlowRaman monitoring of Ru catalyzed aerobic epoxidation reactions under high-pressure.

The FlowNMR apparatus was flushed with laboratory grade acetone followed by dichloromethane, then dried with air before use. To the glass reactor vessel, styrene (0.2600 g,1.25 mmol), mesitylene (0.1250 g, 1.04 mmol), dichloromethane (20 mL), water (2.5 mL) and a Teflon-coated stir bar were added in air, followed by sealing the

autoclave with all tubing attached (Figure S6). On the end of the inlet tubing, a hydrophobic filter (Figure S7, F) was used to prevent the aqueous phase from entering the flow system which could affect the flow rate and spectral quality obtained. After the reaction mixture was circulated through the system at 4 mL/min for 5 minutes without any remaining air bubbles or water droplets in the tip, the NMR flow tube was then inserted into the spectrometer. Both the reactor and NMR probe were heated to 30 °C, and once the temperature had stabilized throughout the system the NMR spectrometer lock was turned off with non-deuterated DCM as solvent, tuned to proton and shimmed on ^{1}H peaks. Acquisition parameters for ^{1}H NMR experiment were entered (see below for details), spectra of the starting material were recorded both statically (no flow) and at 4 mL/min to derive flow correction factors of all relevant integrals. Then 4 mL stock solution of DCM containing [(**L1**)Ru(O)$_2$] (0.5 mol%, 0.0120 g, 0.0125 mmol) was added to the reaction vessel. The autoclave was sealed and quickly pressurized with air (10 bar) to start the reaction and the sequence commenced to start the FlowNMR reaction monitoring. At the end of the reaction, additional calibration spectra with and without flow were recorded before all heating was switched off, the flow stopped, and the reactor carefully vented into the fume hood. An aliquot of the reaction mixture was taken by syringe and further analyzed by GC-FID/MS, ESI, UV-vis.

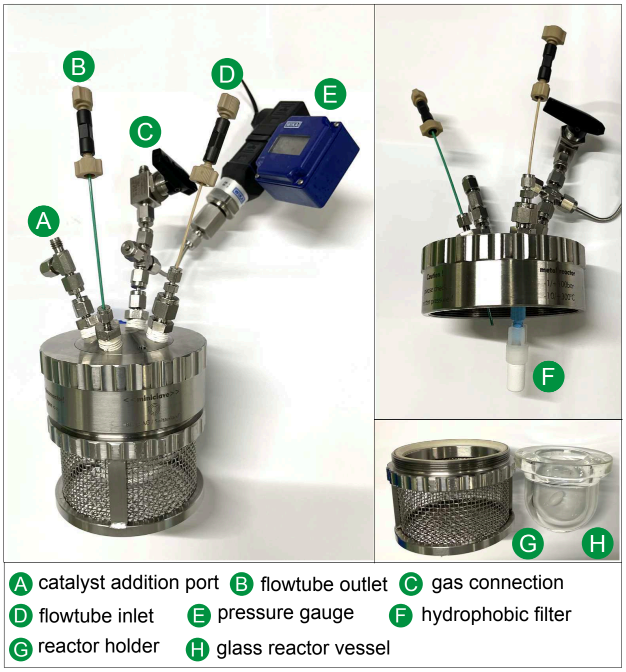

Figure S6. Büchi Miniclave Reactor set up for biphasic FlowNMR and FlowRaman monitoring under pressure.

FlowNMR acquisition parameters:

^{1}H NMR experiments were continuously executed every 2 minutes until the end of the reaction using zg30 with the following settings:

- NS = 16
- D1 = 3s
- RG = 6.35
- O1P = 6.175 ppm
- SW = 40 ppm
- Expt = 76s

Static calibration spectra were recorded with the same parameters but with a D1 of 60 seconds.

Quantitative NMR acquisition Correction factors:

Flow effects resulting from different degrees of pre-magnetisation across different nuclei may be corrected for by comparing integral values from flow spectra with static reference measurements.[2] A correction factor is then calculated for each peak comparing the integral at static and flow conditions to accurately quantify flow data using the following equation:

$$\mathrm{CF} = \frac{\left(\frac{Static\ Peak\ Area_{analyte}}{Static\ Peak\ Area_{internal\ standard}}\right)}{\left(\frac{Flow\ Peak\ Area_{analyte}}{Flow\ Peak\ Area_{internal\ standard}}\right)}$$

$$C_{analyte} = \mathrm{CF} \times \frac{Flow\ Peak\ Area_{analyte}}{Flow\ Peak\ Area_{internal\ standard}} \times \frac{N_{internal\ standard}}{N_{analyte}} \times C_{internal\ standard}$$

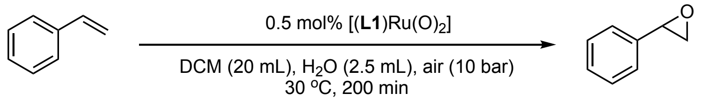

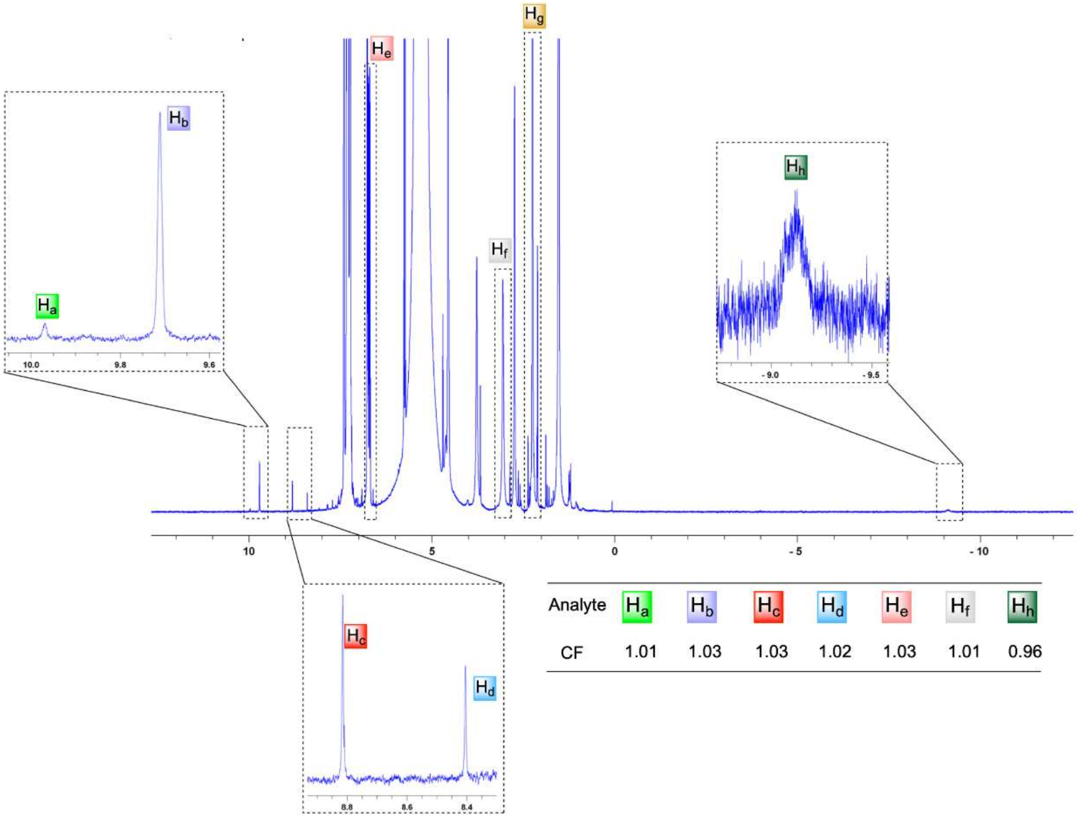

Analyte	H_a	H_b	H_c	H_d	H_e	H_f	H_h
CF	1.01	1.03	1.03	1.02	1.03	1.01	0.96

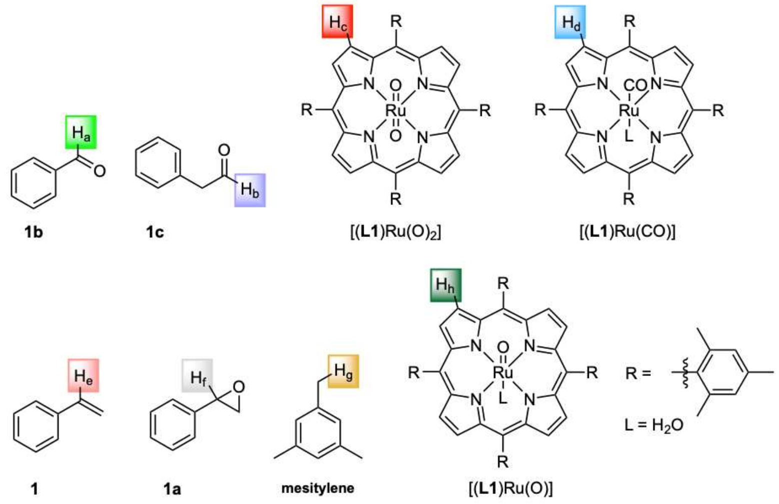

Figure S7. ^{1}H FlowNMR spectrum of reaction mixture at 200 min, with relevant peaks highlighted and their corresponding correction factors (CF) used for quantitative online monitoring.

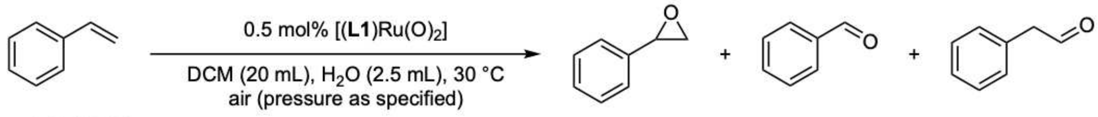

1 (0.125 M) **1a** **1b** **1c**

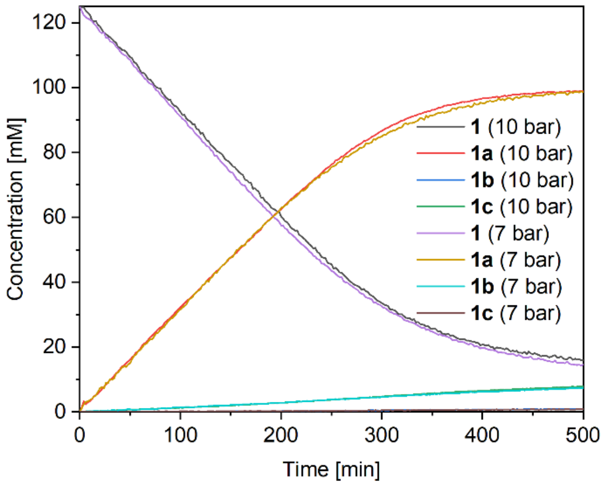

Figure S8. Online monitoring of [(L1)Ru(O)$_2$] catalyzed aerobic epoxidation of styrene in DCM/H_2O under air (7 bar and 10 bar).

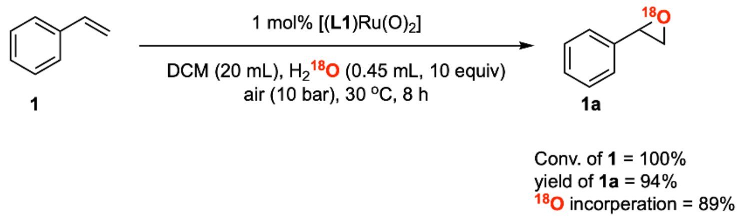

(a)

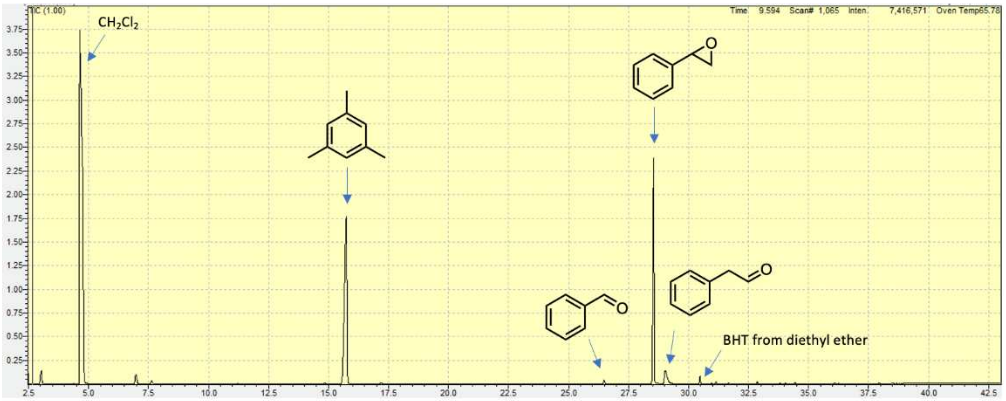

(b)

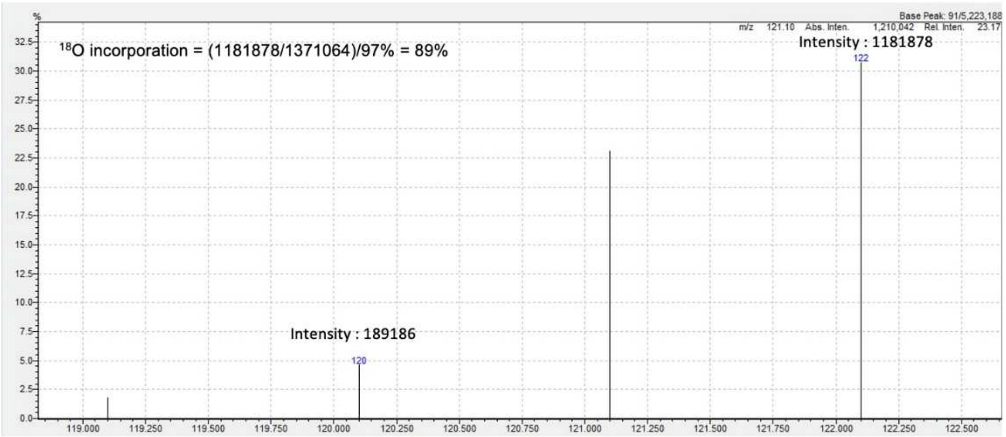

Figure S9. Determination of ^{18}O incorporation into styrene oxide. (a) GC-FID trace from the aerobic oxidation of styrene in DCM with $H_2{}^{18}O$ in $^{16}O_2$; (b) mass spectrometry of styrene oxide peak from the aerobic oxidation of styrene in DCM with $H_2{}^{18}O$ in $^{16}O_2$.

Supplementary Method D: FlowRaman Spectroscopy and band assignment

Raman spectroscopy data was recorded with a Tornado HyperFlux™ Pro Plus Raman Analyser (495 mW @ 785 nm) with a stainless steel BallProbe® (1/2'', 12.7 mm, sapphire windows sealed with gold). The BallProbe® was directly added into the flow path of the MarqMetrix Raman FlowCell, which was coupled to the fluid flow path between the inlet tubing of the reactor and Teflon InsightMR flow tube (Figure S5). The reaction was monitored with SpectralSoft (Tornado spectral) using the following acquisition parameters:

Laser wavelength: 785 nm

Spectral range: 200 cm^{-1} to 3300 cm^{-1}

Expose time: 34 ms

Laser power: 285 mW

Interval time: 3s

The Raman spectral data were processed with Panorama Pro (LabCongition).

Table S4. Observed Raman stretching frequencies of Ru complexes including their isotope band shift[3]

Compound	$^{16}O{=}Ru^{VI}{=}^{16}O$	$^{18}O{=}Ru^{VI}{=}^{18}O$	$^{16}OH{-}Ru^{IV}{-}^{16}OH$ or $^{16}O{=}Ru^{IV}{-}^{16}OH_2$	$^{18}OH{-}Ru^{IV}{-}^{18}OH$ or $^{18}O{=}Ru^{IV}{-}^{18}OH_2$
$\nu_{obs.}$	810 cm^{-1}	764 cm^{-1}	820 cm^{-1}	775 cm^{-1}
Obs. Isotope shift	46 cm^{-1}		45 cm^{-1}	
Calc. Isotope shift	46.4 cm^{-1}		46.9 cm^{-1}	
Ref.	3		This work	

For the (H)O-Ru-O(H) complexes, the Raman-active symmetric stretching frequency ν in a linear triatomic model was calculated using equation below[4]:

$$\nu = \frac{1}{2\pi c}\sqrt{\frac{F+k}{m_o}}$$

Where the c is the velocity of light (3×10^{10} cm/s), m is the mass of O atoms, F and k are Ru-O(H) bond force constant and interaction force constants.

In addition, the reaction profile of the ruthenium species observed at 820 cm^{-1} and 775 cm^{-1} (Figure S11a & S13a) mirrored the reaction profile of the [(**L1**)Ru(O)(H_2O)] species observed using ^1H FlowNMR (Figures S11b & S13b), suggesting that [(**L1**)Ru$(OH)_2$] and [(**L1**)Ru(O)(H_2O)] interconvert through oxo-hydroxo tautomerization as described in the literature.[5] Thus, the bands at 820 cm^{-1} and 775 cm^{-1} have been assigned to [(**L1**)Ru($^{16}OH)_2$]/[(**L1**)Ru(^{16}O)($^{16}OH_2$)] and [(**L1**)(Ru$^{18}OH)_2$]/[(**L1**)Ru(^{18}O)($^{18}OH_2$)], respectively.

Note: A Raman band of [(**L1**)Ru(^{16}O)(^{18}O)] has previously been reported at 774 cm^{-1},[3] thus we could not exclude the possibility that a small amount of [(**L1**)Ru(^{16}O)(^{18}O)] was also formed during the reaction with $^{18}OH_2$ as additive.

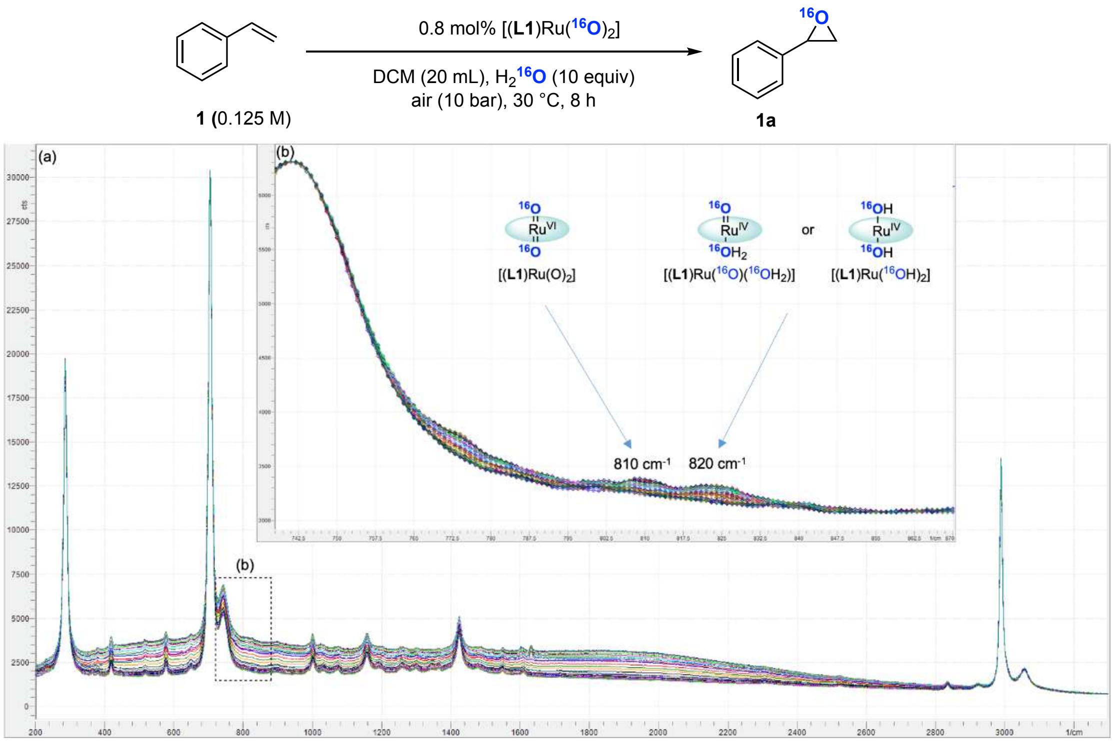

Figure S10. FlowRaman spectral of the aerobic epoxidation of styrene in $^{16}OH_2$ saturated DCM under $^{16}O_2$. (a) Full spectral window acquired; (b) Ru-oxo region with DCM (band at 741 cm^{-1}) as Y scale reference.

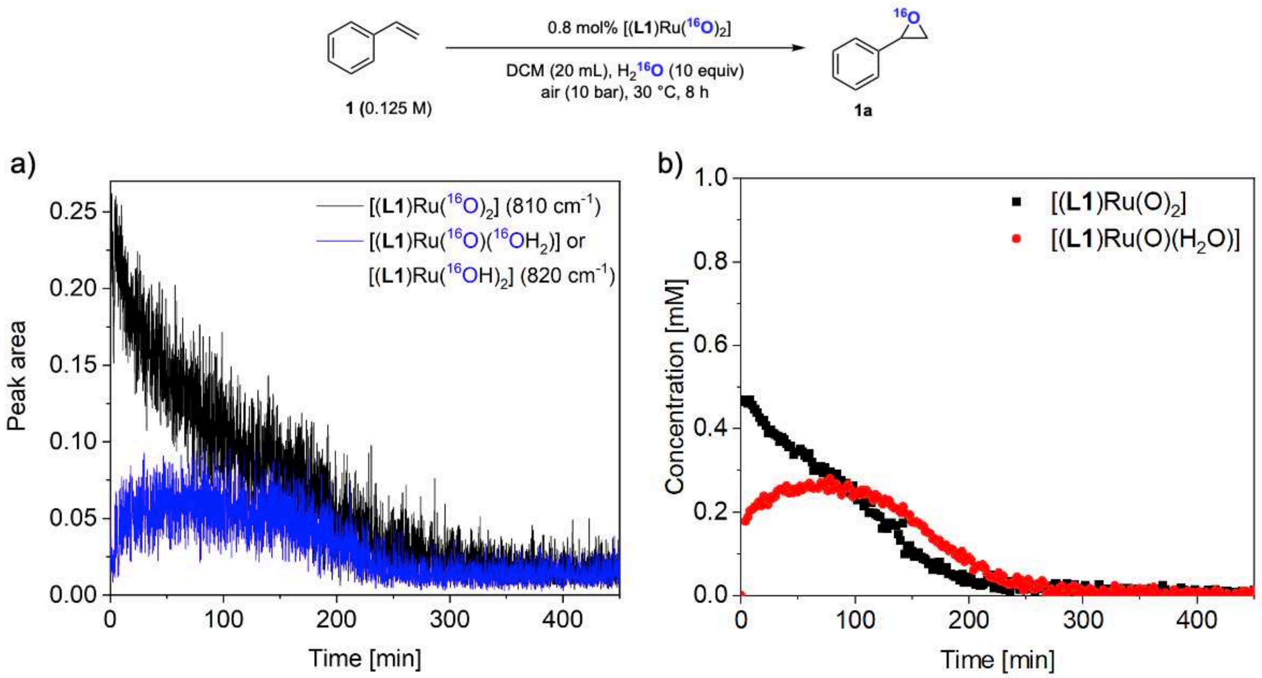

Figure S11. Reaction profile of ruthenium species observed by FlowRaman and ^{1}H FlowNMR in $^{16}OH_2$ saturated DCM under $^{16}O_2$. (a) Peak area plot of ruthenium species observed by FlowRaman. (b) Ruthenium species observed by ^{1}H FlowNMR.

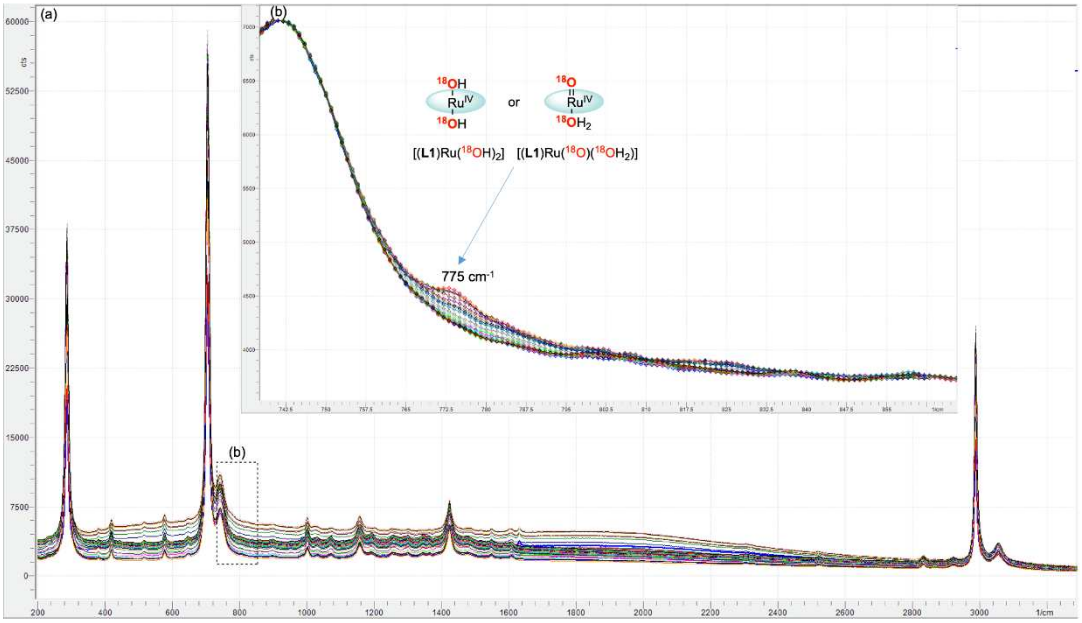

Figure S12. FlowRaman spectral of the aerobic epoxidation of styrene in $^{18}OH_2$ saturated DCM under $^{16}O_2$. (a) Full spectral window acquired; (b) Ru-oxo region with DCM (band at 741 cm^{-1}) as Y scale reference.

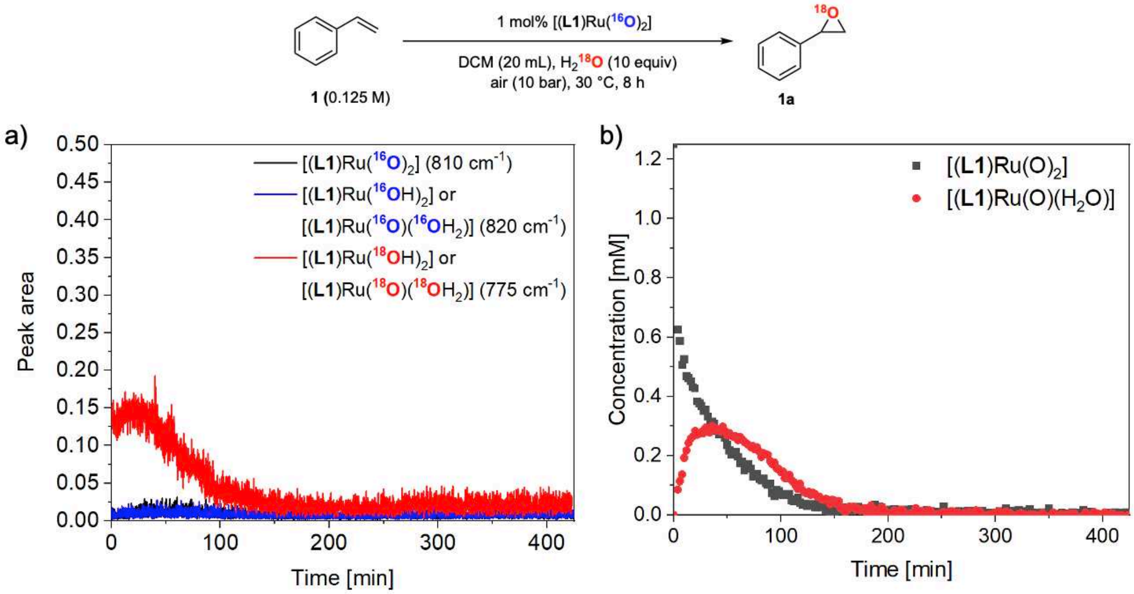

Figure S13. Reaction profile of ruthenium species observed by FlowRaman and ^{1}H FlowNMR in $^{18}OH_2$ saturated DCM under $^{16}O_2$. (a) Peak area plot of ruthenium species observed by FlowRaman. (b) Ruthenium species observed by ^{1}H FlowNMR.

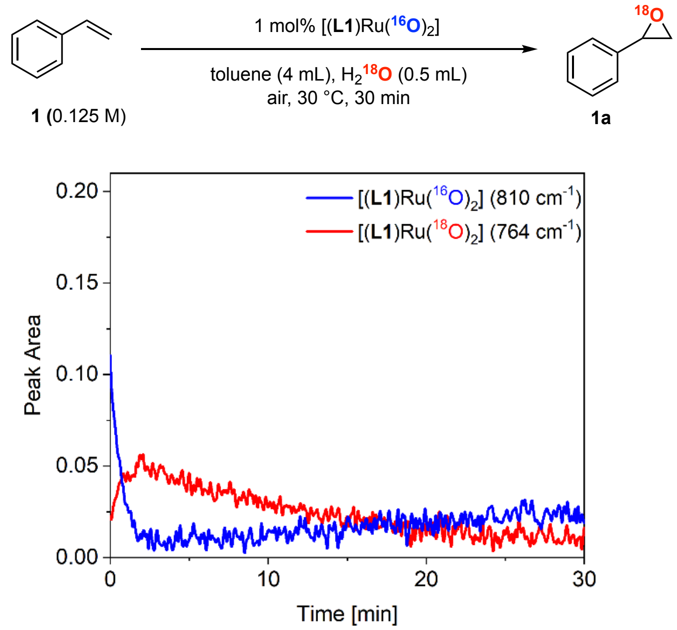

Figure S14. Peak area plot of *in situ* Raman spectra of aerobic epoxidation of styrene in $^{18}OH_2$ saturated toluene under $^{16}O_2$.

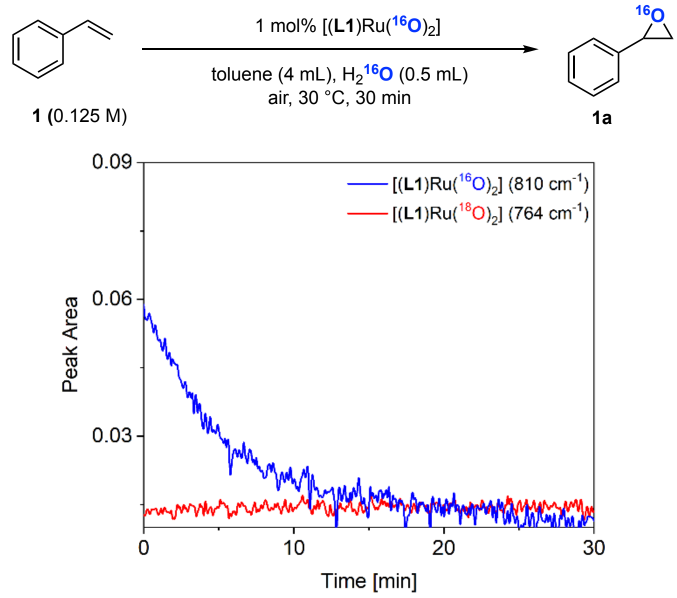

Figure S15. Peak area plot of *in situ* Raman data of aerobic epoxidation of styrene in $^{16}OH_2$ saturated toluene under $^{16}O_2$.

Supplementary Method E: ^{17}O NMR analysis

[(**L1**)Ru(O)$_2$] (0.0010 g, 0.0011 mmol) was dissolved in 0.5 mL dry $CDCl_3$ (dried with 3Å molecular sieves) and transferred to an NMR tube. To this mixture $^{17}OH_2$ (35-40%, Cambridge Isotope Laboratories, 100 mL, 0.0022 mmol, 2.0 equiv.) was added by micro syringe. After shaking for one minute at room temperature the sample was analyzed by ^{17}O NMR spectroscopy on a Bruker 500 MHz Advance II$^+$ Ultrashield equipped with a nitrogen-cooled BBO Prodigy CryoProbe operating at 67.8 MHz controlled through TopSpin (4.0.8). Spectra were manually phased, baseline corrected with apodization to 20 Hz line broadening, and the free water line centered to 0 ppm. Measurements were performed with sample spinning but without lock and sweep using the following parameters: p1 = 11.5 μs, d1 = 100 ms, aq = 48 ms, sw = 5000 ppm, ns = 10.000, time = 35 min.

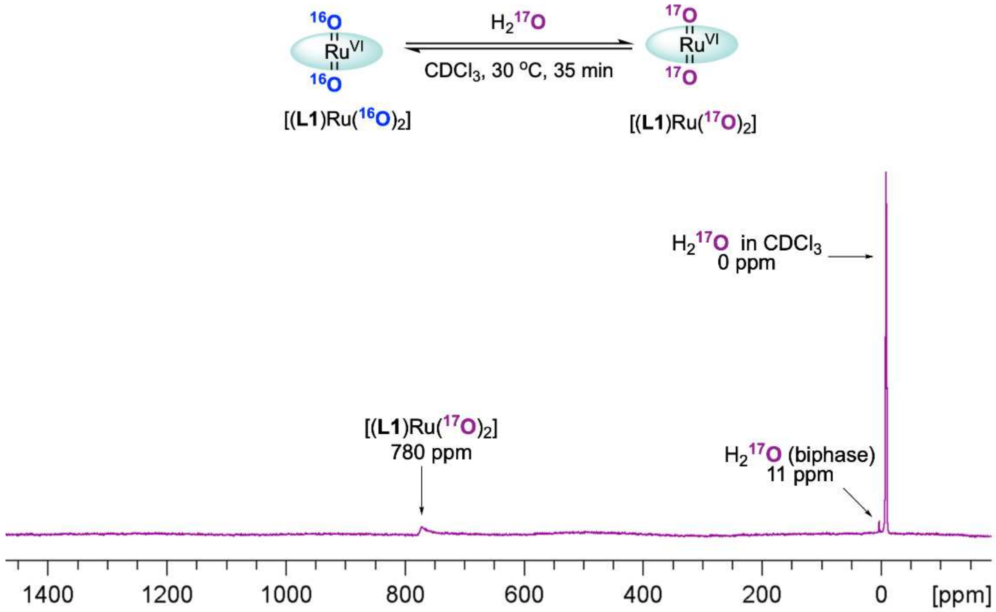

Figure S16. ^{17}**O NMR spectrum of** [(L1)Ru^{VI}(^{16}O)$_2$] **with** ^{17}OH$_2$ **in** CDCl$_3$

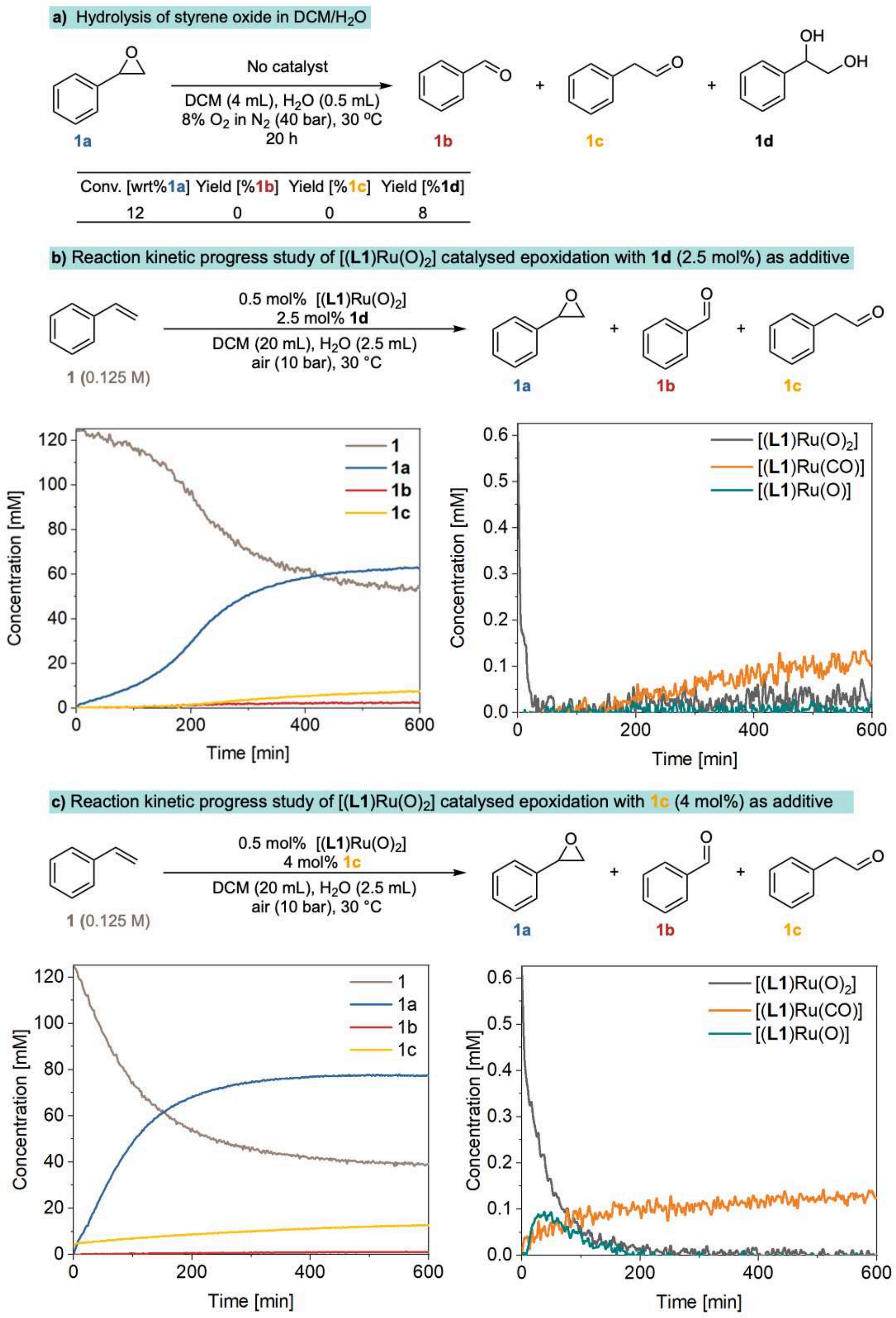

Figure S17. Investigation of catalyst inhibition and deactivation. (a) hydrolysis of **1a** in water-saturated DCM. (b) Reaction progress and catalyst speciation of [(**L1**)Ru(O)$_2$] catalyzed epoxidation of **1** with the addition of 1d (2.5 mol%). (c) Reaction progress and catalyst speciation of [(**L1**)Ru(O)$_2$] catalyzed epoxidation of **1** with the addition of 1c (4 mol%).

ESI-MS of reaction mixture

When the reaction was finished, a sample from the reaction mixture (200 mL) was taken and diluted with acetone (4 mL) and water (0.2 mL) before ESI-MS analysis. In cases where an additive was needed, 100 mL of an aqueous solution of formic acid (1 M) was added to the mixture and stirred for 1 min. The sample mixture was injected into a Bruker Daltonics-micrOTOF-Q mass spectrometer using a syringe pump with an injection speed of 0.1 mL/min. Data was recorded with the following parameters unless otherwise specified: -500 V end plate offset voltage, 4500 V capillary voltage, 1800-2300 V TOF detector, 220 °C dry temperature. The MS/MS collision energy range used was 0-200 eV. For details of porphyrin-Ru complexes observed by ESI-MS and their simulated spectra generated in Bruker DataAnalysis 4.3 software see the following Figures S18-22 and Supplementary Information Section 5: ESI-MS of Ru complexes and their simulated spectra.

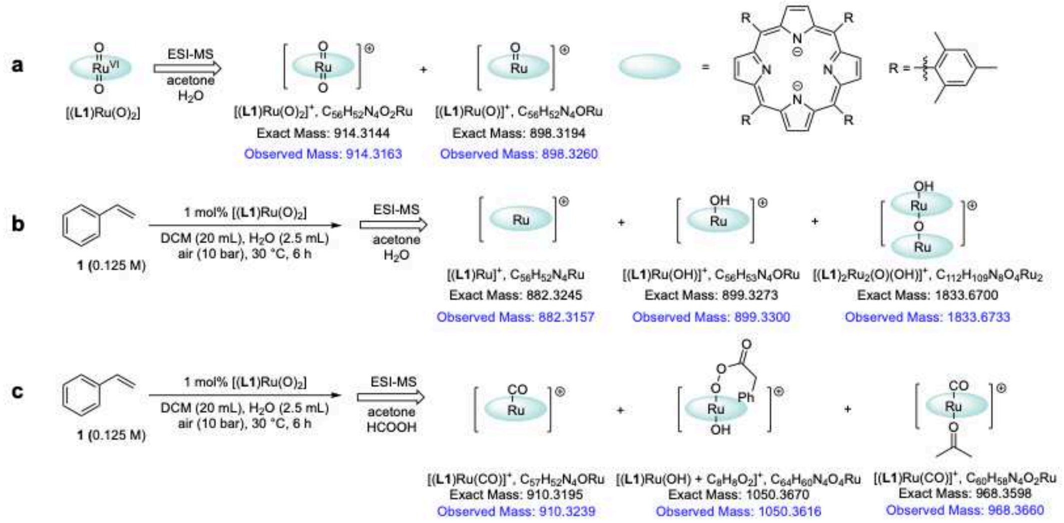

Figure S18. ESI-MS analysis. **a**, ESI-MS of [(**L1**)Ru(O)$_2$] in acetone/H_2O. **b**, ESI-MS of reaction mixture in acetone/H_2O. **c**, ESI-MS of reaction mixture in acetone with aq. HCOOH as additive.

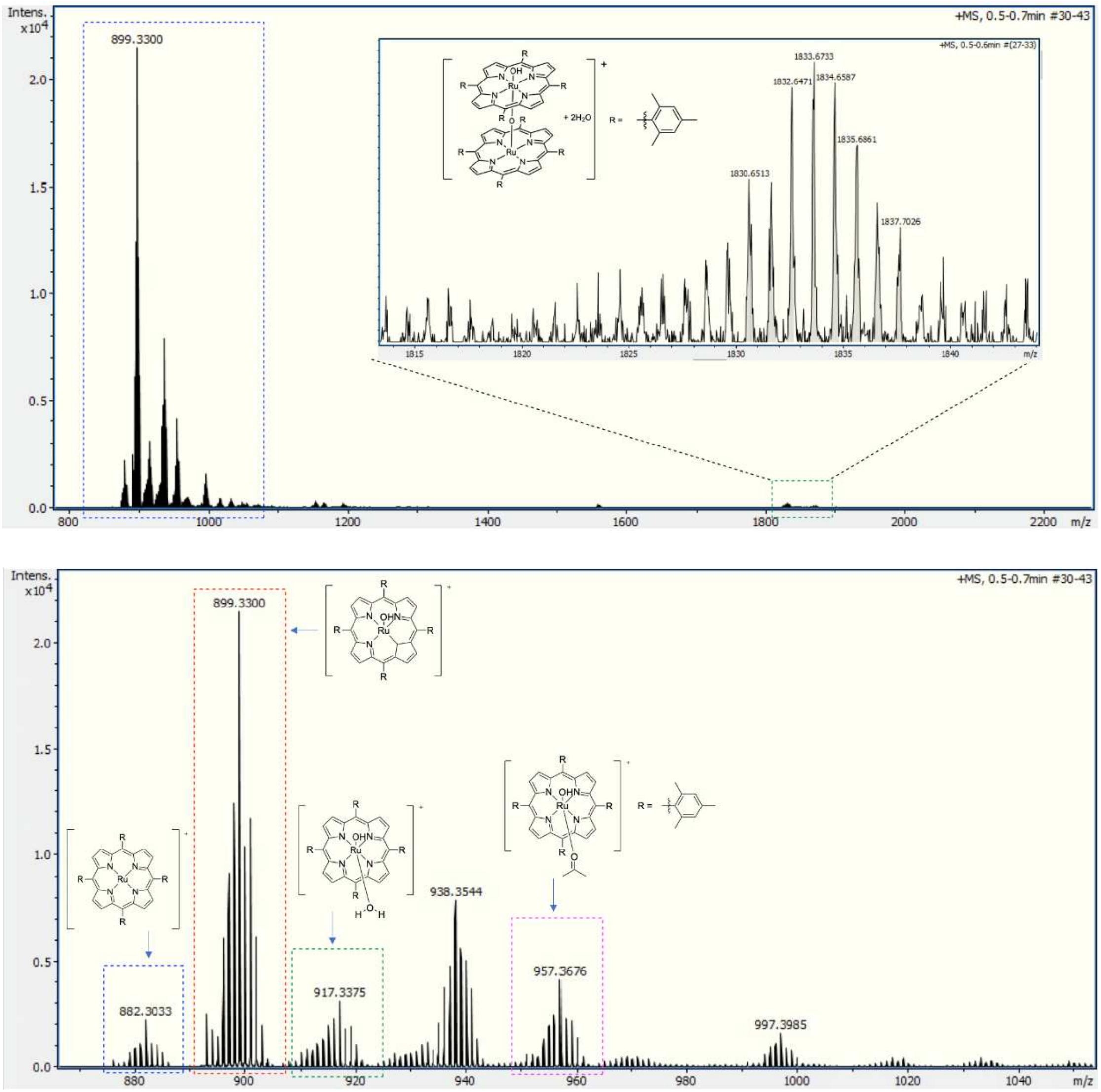

Figure S19. ESI-MS of reaction mixture (conditions see Figure S19b) in acetone/H_2O

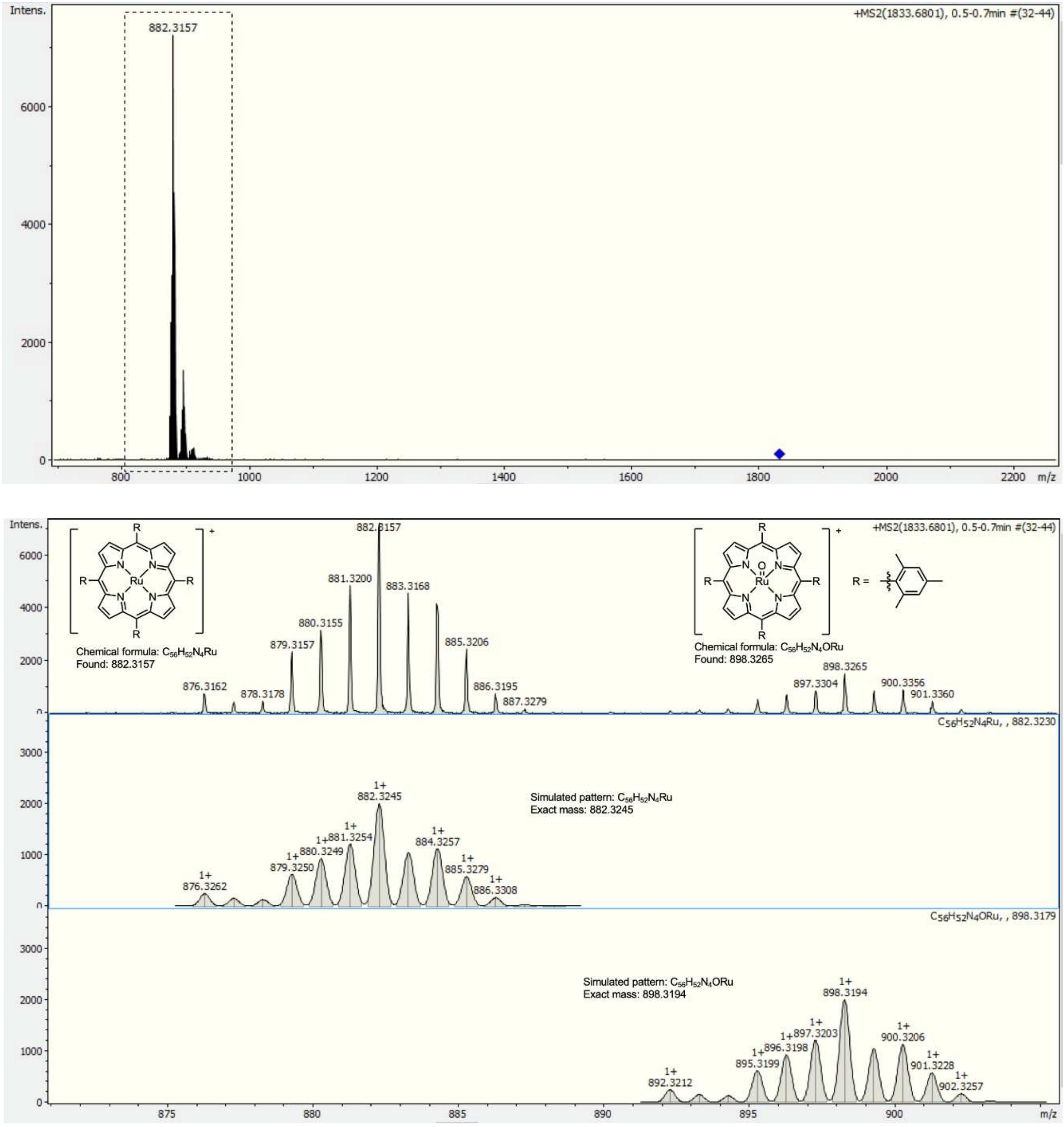

Figure S20. MS/MS fragmentation of m/z = 1833.67

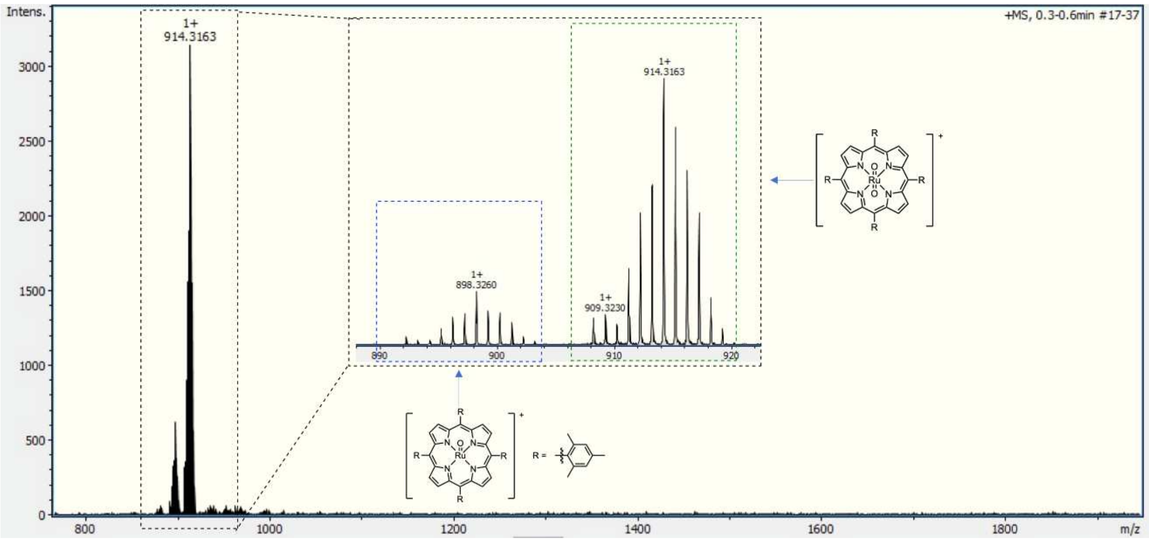

Figure S21. ESI-MS of [(**L1**)Ru(O)$_2$] in acetone/H_2O without additive

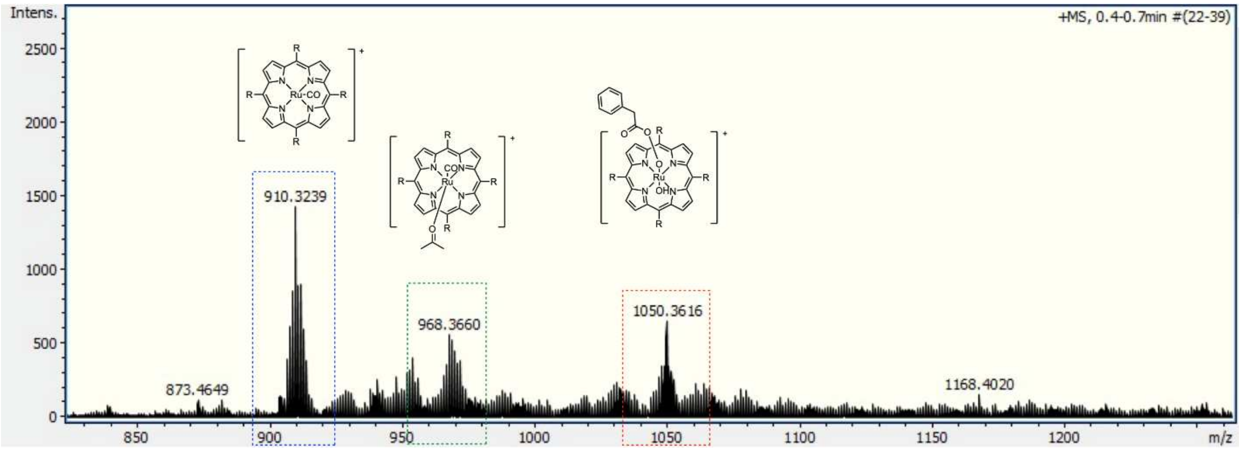

Figure S22. ESI-MS of reaction mixture (conditions see Figure S19c) in acetone with aq. HCOOH

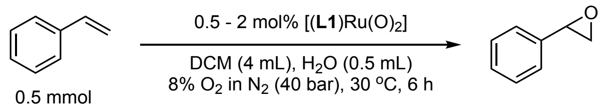

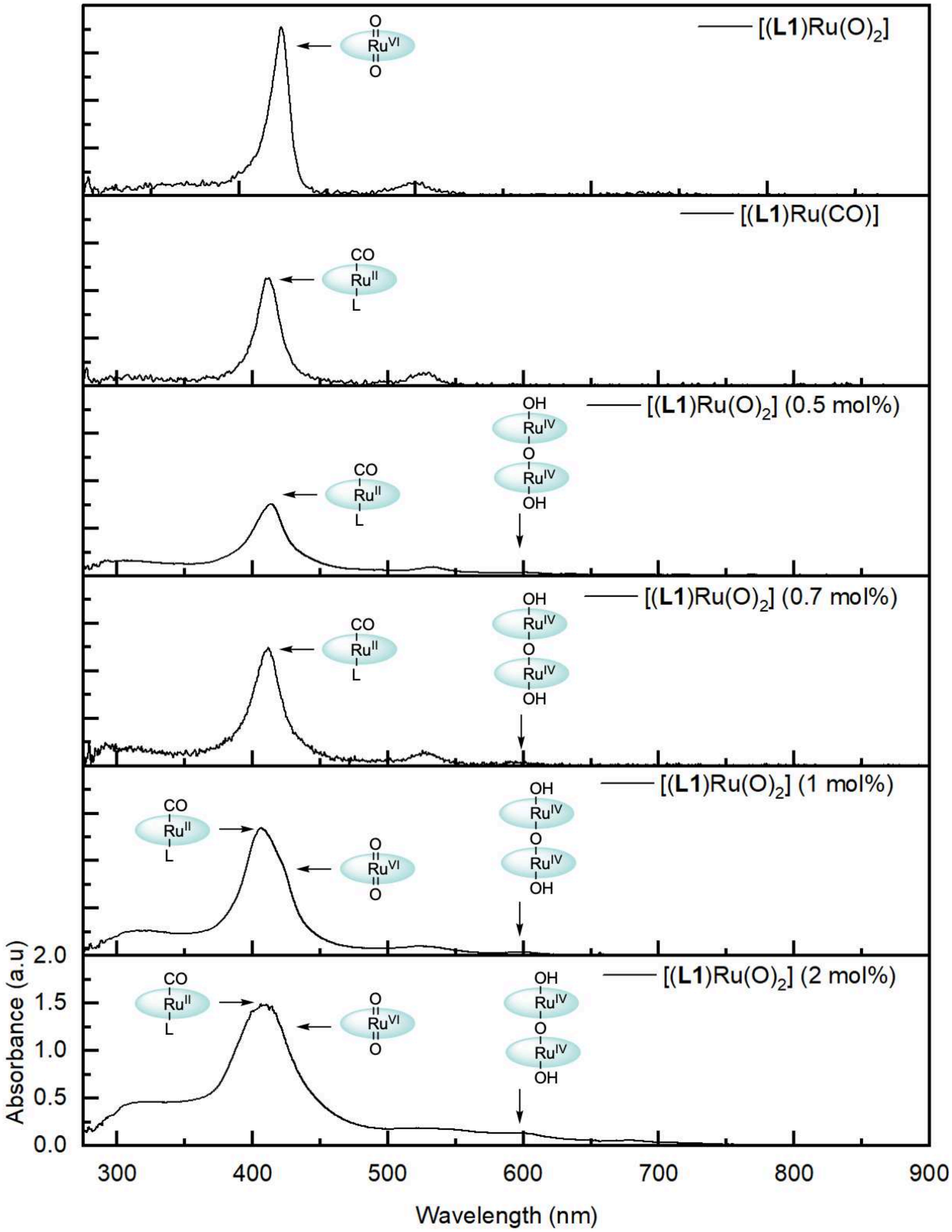

Figure S23. UV-vis spectra of reaction mixtures with different catalyst loadings after 6 h compared to pure samples of [(**L1**)Ru(O)$_2$] and [(**L1**)Ru(CO)] in DCM

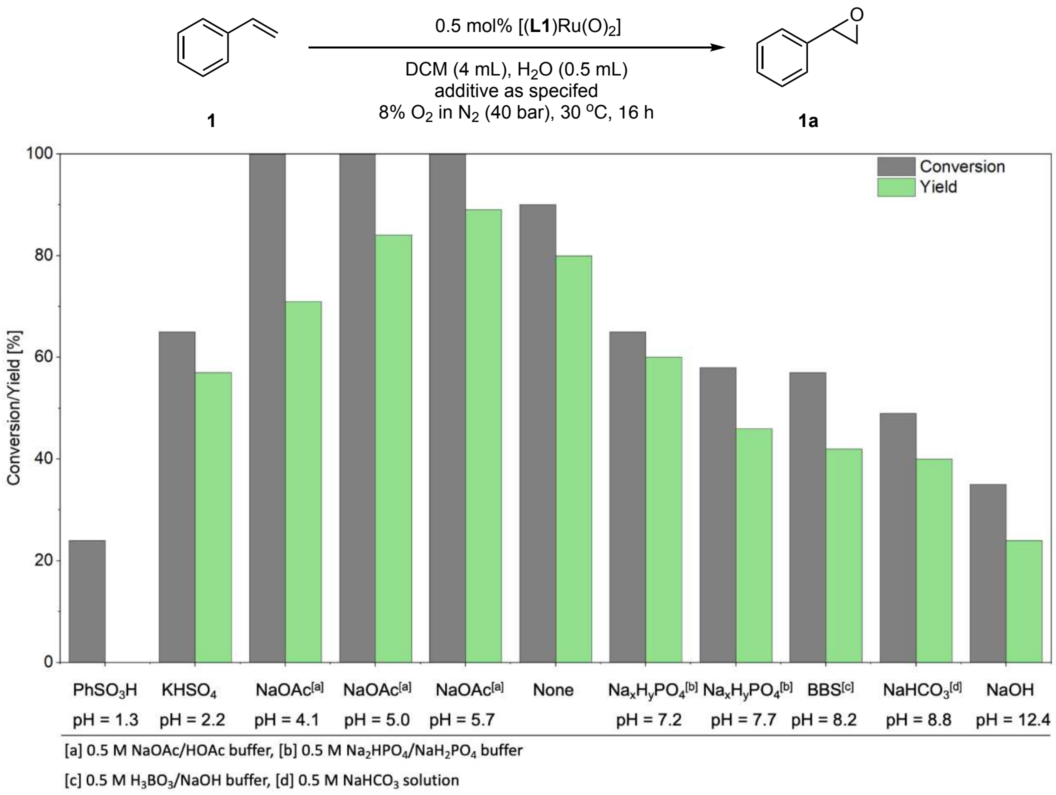

Figure S24. Effect of additive and pH on [(L1)Ru(O)$_2$] catalyzed aerobic epoxidation of styrene.

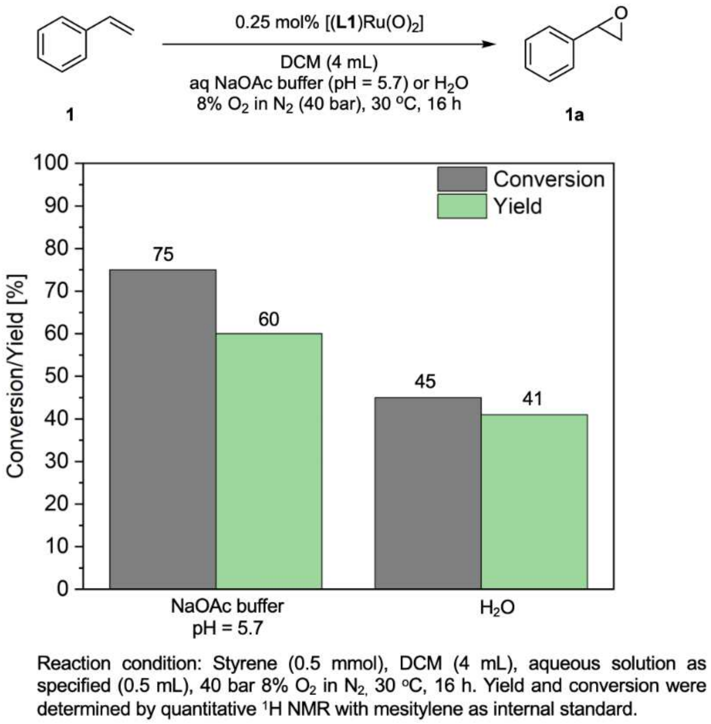

Figure S25. Aerobic epoxidation of styrene with low loadings of [(L1)Ru(O)$_2$].

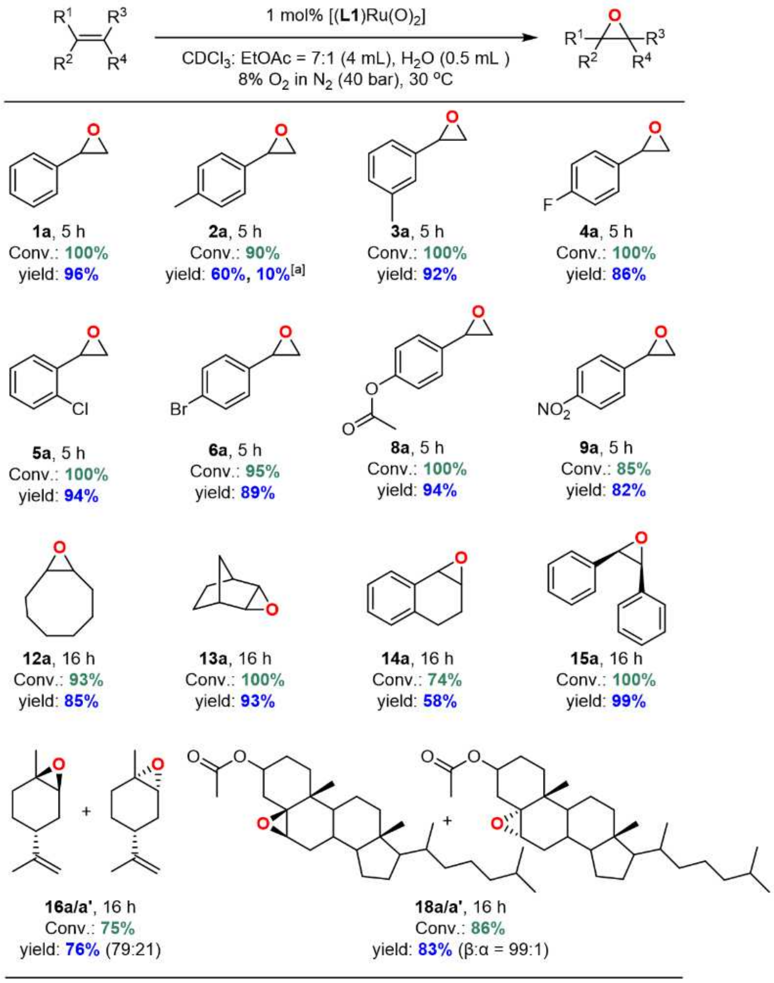

Figure S26. Substrate scope of [(L1)Ru(O)$_2$] catalyzed aerobic epoxidation in solvent mixtures of $CDCl_3$ and EtOAc. Conversion and yield were determined by quantitative ^{1}H NMR using mesitylene as internal standard. [a]Yield of corresponding aldehyde (isomerized from corresponding epoxide).

Table S5. Substrate limitations of [(L1)Ru(O)$_2$] catalyzed aerobic epoxidation

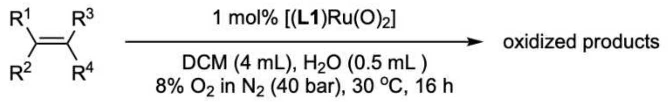

Entry	Substrate	Product	Conv. [%][a]	Yield [%][a]
1	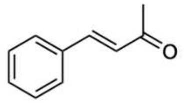	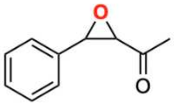	0	0
2	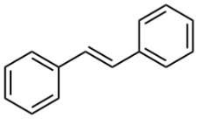	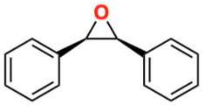	0	0
3	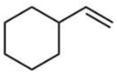	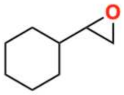	30	0
4	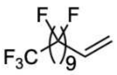	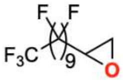	0	0
5	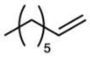	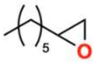	23<br>46[b]	21<br>43[b]
6[c]	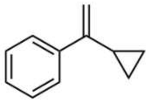	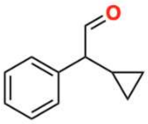	100	90 (84)

[a] Yield and conversion were calculated using ^1H NMR with mesitylene as internal standard. Isolated yields provided in parenthesis.
[b] 2 mol% [(**L1**)Ru(O)$_2$] was used.
[c] 5 h

Note: The formation of 2-cyclopropyl-2-phenylacetaldehyde from (1-cyclopropylvinyl)benzene (Entry 6) is consistent with the observation by Che and co-workers, in which corresponding aldehydes could be formed from various α-substituted styrenes/naphthenes *via* ruthenium porphyrin catalyzed epoxidation-Isomerization pathway.[6]

3 Characterization Data for porphyrins and Ru complexes

5,10,15,20-Tetramesitylporphyrin (L1)

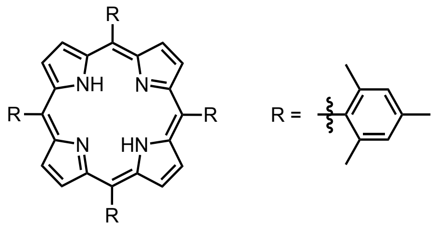

This compound was prepared using a method modified from literature.[7] A 1 L two-neck round-bottomed flask fitted with a septum, reflux condenser, was charged with 500 mL of $CHCl_3$ (stabilized with ethanol), mesitaldehyde (0.7410 g, 5 mmol), and pyrrole (0.3427 g, 5 mmol) under N_2. Then, 0.66 mL of 2.5 M BF_3-OEt_2 (1.65 mmol) was added *via* syringe. After the reaction was stirred for 2 h at room temperature *p*-chloranil (0.922 g, 3.75 mmol) was added in powder form and the reaction mixture was gently refluxed (~60 °C) for 1 h. The reaction mixture then was cooled to room temperature, triethylamine (1.65 mmol, 230 µL) was added, and the mixture was dried under reduced pressure. The crude dry product was scraped from the flask, placed on a filter, and washed with methanol (~75 mL) until the filtrate was colourless. The product was further purified by column chromatography (DCM:Petroleum ether = 1:3) then the product containing fractions were combined and concentrated and dried under reduced pressure to give the product as purple crystals. Yield: 0.1564 g, 20%. **^{1}H NMR** (400 MHz, $CDCl_3$) δ 8.61 (s, 8H), 7.26 (s, 8H), 2.62 (s, 12H), 1.85 (s, 24H), -2.51 (s, 2H). NMR data is consistent with literature values.[8] **HRMS** (ES+) calcd. for $[M+H]^+$ $C_{56}H_{55}N_4$: 783.4427, found: 783.4426.

5,10,15,20-Tetrakis(2,6-dichlorophenyl)porphyrin (L2)

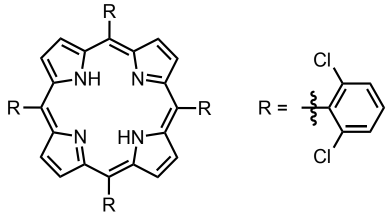

This compound was prepared using a method modified from literature.[7] A 1 L two-neck round-bottomed flask fitted with a septum, reflux condenser, was charged with 500 mL of $CHCl_3$ (stabilized with ethanol), 2,6-dichlorobenzaldehyde (0.8750 g, 5 mmol), and pyrrole (0.3427 g, 5 mmol) under N_2. Then, 0.66 mL of 2.5 M BF_3-OEt_2 (1.65 mmol) was added via syringe. After the reaction was stirred for 2 h at room temperature *p*-chloranil (0.922 g, 3.75 mmol) was added in powder form and the reaction mixture was gently refluxed (~ 60 °C) for 1 h. The reaction mixture then was cooled to room temperature, triethylamine (1.65 mmol, 230 μL) was added, and the solution was dried under reduced pressure. The crude dry product was scraped from the flask, placed on a filter, and washed with methanol (~75 mL). Then the crude product was further purified by column chromatography (DCM:Petroleum ether = 2:3) then the product containing fractions were combined and concentrated and dried under reduced pressure to give the product as purple crystals. Yield: 0.1918 g, 43%. **^{1}H NMR** (500 MHz, $CDCl_3$) δ 8.67 (s, 8H), 7.83-7.76 (m, 8H), 7,74-7.67 (m, 4H), -2.53 (s, 2H). NMR data is consistent with literature values.[9] **HRMS** (ES+) calcd. for $[M+H]^+$ $C_{44}H_{23}N_4Cl_8$: 886.9431, found: 886.9431.

5,10,15,20-Tetrakis(perfluorophenyl)porphyrin (L3)

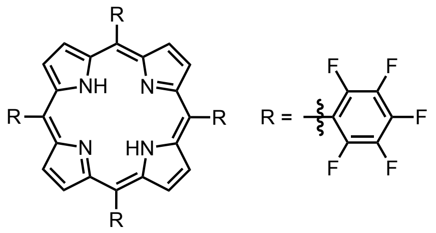

This compound was prepared using a method modified from literature.[10] A two-neck 1 L round-bottomed flask fitted with a septum, reflux condenser, was charged with 400 mL of DCM (stabilized by amylene), 2,3,4,5,6-pentafluorobenzaldehyde (3.82 g, 19.4 mmol), and pyrrole (1.355 g, 20.2 mmol) under N_2. Then, 0.3 mL BF_3-OEt_2 (2.38 mmol) was added via syringe. After the reaction was stirred for 16 h at room temperature *p*-chloranil (1.90 g, 7.7 mmol) was added in powder form and the reaction mixture was gently refluxed (~60 °C) for 3 h. The reaction mixture then was cooled to room temperature, triethylamine (1.65 mmol, 230 µL) was added, and the reaction mixture was dried under reduced pressure. The crude dry product was scraped from the flask, placed on a filter, and washed with methanol (~75 mL) until the filtrate was nearly clear. The product was further purified by column chromatography ($CHCl_3$:petroleum ether = 2:3) then the product containing fractions were combined and concentrated and dried under reduced pressure to give the product as purple crystals. Yield: 0.7328 g, 15%. **^{1}H NMR** (500 MHz, $CDCl_3$) δ 8.93 (s, 8H), -2.90 (s, 2H). **^{19}F-NMR** (470 MHz, $CDCl_3$) δ -139.5 (m, 8F), -151.2 (t, *J* = 20.8 Hz, 4F), -161.3 (m, 8F). NMR data is consistent with literature values[10]. **HRMS** (ES+) calcd. for $[M+H]^+$ $C_{44}H_{11}N_4F_{20}$: 975.0664, found: 975.0660.

2,6-Diphenoxybenzaldehyde

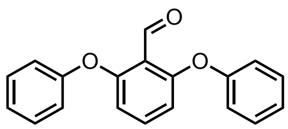

This compound was prepared using a method modified from literature[11]. To a stirred solution of 1,3-diphenoxybenzene (2.6210 g, 10 mmol) in dry THF (60 mL) at 0 °C, n-BuLi (4.8 mL, 12 mmol) was added dropwise for 30 min. Then the mixture was stirred at room temperature for 2 h, followed by slow addition of DMF (1,83 g, 25 mmol). After 2 h, the mixture was poured into ice water. The organic phase was separated, and the aqueous phase was extracted with diethyl ether (3 x 30 mL). The combined organic layers were dried over anhydrous Na_2SO_4. After the removal of solvent under reduced pressure, the product was purified by column chromatography with petroleum ether/EtOAc = 9:1 as eluent. After drying, the titled compound was obtained as a white solid. Yield: 1.5465 g, 53%. **^{1}H NMR** (500 MHz, $CDCl_3$) δ 10.60 (s, 1H), 7.42-7.36 (m, 4H), 7.32 (t, *J* = 8.3, 1H), 7.21-7.15 (m, 2H), 7.12-7.06 (m, 4H), 6.58 (d, *J* = 8.3 Hz, 2H). **^{13}C NMR** (126 MHz, $CDCl_3$) δ 188.4, 160.3, 156.3, 135.3, 130.1, 124.5, 119.8, 118.6, 112.9. NMR data is consistent with literature values.[11] **HRMS** (ES+) calcd. for $[M+H]^+$ $C_{19}H_{15}O_3$: 291.1016, found: 291.0998.

5,10,15,20-Tetrakis(2,6-diphenoxyphenyl)porphyrin (L4)

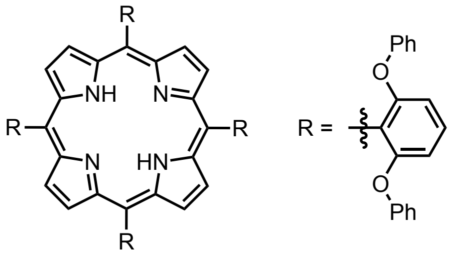

This compound was prepared using a method modified from literature.[7] A 1 L two-neck round-bottomed flask fitted with a septum, reflux condenser, was charged with 500 mL of $CHCl_3$ (stabilized with ethanol), 2,6-diphenoxybenzaldehyde (1.450 g, 5 mmol), and pyrrole (0.3427 g, 5 mmol) under N_2. Then, 0.66 mL of 2.5 M BF_3-OEt_2 (1.65 mmol) was added via syringe. After the reaction was stirred for 2 h at room temperature *p*-chloranil (0.922 g, 3.75 mmol) was added in powder form and the reaction mixture was gently refluxed (~60 °C) for 1 h. The reaction mixture then was cooled to room temperature, triethylamine (1.65 mmol, 230 μL) was added, and the solution was dried under reduced pressure. The crude dry product was scraped from the flask, and further purified by column chromatography (DCM:Petroleum ether = 2:3) then the product containing fractions were combined and concentrated under reduced pressure to give the product as dark purple crystals. Yield: 0.2194 g, 13%. **^{1}H NMR** (500 MHz, $CDCl_3$) δ 8.88 (s, 8H), 7.61 (t, *J* = 8.4 Hz, 4H), 7.08 (d, *J* = 8.4 Hz, 8H), 6.76-6.68 (m, 16H), 6.58-6.54 (m, 16H), 6.51 (t, *J* = 7.3 Hz, 8H), -2.96 (s, 2H). NMR data is consistent with literature values.[7] **HRMS** (ES+) calcd. for $[M+H]^+$ $C_{92}H_{63}N_4O_8$: 1351.4646, found: 1351.4642.

(*Z*)-1,3,5-Trimethyl-2-(2-nitroprop-1-en-1-yl)benzene

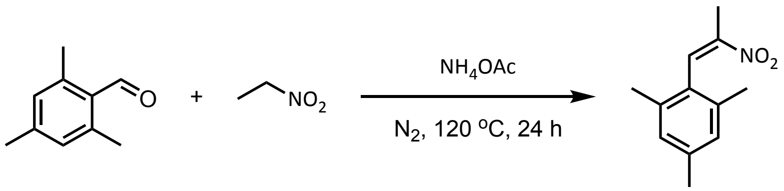

This compound was prepared using a method modified from literature[12]. Mesitaldehyde (9.8572 g, 66.51 mmol) ammonium acetate (5.127 g, 66.51 mmol) and nitroethane (20 mL, 279.73 mmol) were added to a 100 mL round bottom flask, which was fitted with a waterless reflux condenser. The reaction mixture was then heated to reflux at 120 °C for 24 h under N_2. After the reaction was cooled to room temperature, ethanol (30 mL) was added to the reaction mixture, the resulting yellow solid product was filtered and washed with cold ethanol. Yield: 6.9573 g, 51%. **^{1}H NMR** (500 MHz, $CDCl_3$) δ 8.00 (s, 1H), 6.91 (s, 2H), 2.30 (s, 3H), 2.16 (s, 6H), 2.03 (s, 3H). **^{13}C NMR** (126 MHz, $CDCl_3$) δ 149.1, 138.4, 135.5, 133.4, 128.6, 128.6, 21.0, 20.0, 13.7. **HRMS** (ES+) calcd. for $[M+H]^+$ $C_{12}H_{16}NO_2$: 206.1176, found: 206.1210.

Ethyl 3-mesityl-4-methyl-1*H*-pyrrole-2-carboxylate

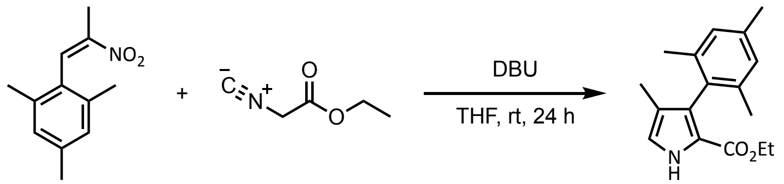

This compound was prepared using a method modified from literature.[12] In a 100 mL round bottom flask, (*Z*)-1,3,5-trimethyl-2-(2-nitroprop-1-en-1-yl)benzene (1.0083 g, 4.912 mmol) and ethyl isocyanoacetate (0.8377 g, 7.406 mmol) were dissolved in dry THF (45 mL). The flask was flushed with nitrogen in an ice bath before the slow addition of 1,8-Diazabicyclo[5.4.0]undec-7-ene (DBU) (2.232 g, 14.661 mmol). Then the reaction mixture was left to stir at room temperature for 24 h. When reaction was finished, an orange-brown residue was obtained after removal of solvent under reduced pressure. The crude product was purified by column chromatography (ethyl acetate:petroleum ether = 1:10), and the product containing fractions were combined and concentrated under reduced pressure to give the product as white solid. Yield: 1.3479 g, 68%. **^{1}H NMR** (400 MHz, $CDCl_3$) δ 8.95 (s, 1H), 6.89 (s, 2H), 6.83 (d, *J* = 2.6 Hz, 1H), 4.07 (q, *J* = 7.1 Hz, 2H), 2.31 (s, 3H), 1.96 (s, 6H), 1.79 (s, 3H), 1.02 (t, *J* = 7.1 Hz, 3H). **^{13}C NMR** (126 MHz, $CDCl_3$) δ 161.3, 136.7, 136.1, 131.7, 130.0, 127.6, 120.6, 120.4, 118.9, 59.7, 21.3, 20.4, 14.2, 10.3. **HRMS** (ES+) calcd. for $[2M+K]^+$ $C_{34}H_{42}N_2O_4K$: 581.2782, found: 581.2764.

2,7,12,17-Tetramesityl-3,8,13,18-tetramethylporphyrin (L5)

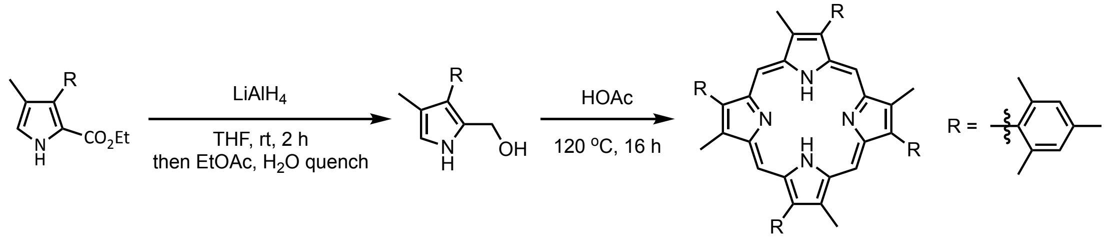

This compound was prepared using a method modified from literature.[12] To a 25 mL round bottom flask, ethyl 3-mesityl-4-methyl-1H-pyrrole-2-carboxylate (1.0608 g, 2.9838 mmol) was dissolved in dry THF (10 mL) and the flask flushed with nitrogen before being placed in an ice bath. While stirring, $LiAlH_4$ (6 mL, 6 mmol) was added slowly to the dissolved ester *via* syringe over 10 minutes. The reaction was left to stir for 2 hours before transferring to a 250 mL round bottom flask and quenching with ethyl acetate (5 mL) and water (0.5 mL) to deactivate excess $LiAlH_4$. Then the the solvent of reaction mixture was removed under reduced pressure. After the addition of acetic acid (160 mL), the reaction mixture was heated to reflux at 120°C under air. After 3 hours, the reaction was cooled to room temperature and the acetic acid was removed under reduced pressure. The dark brown residue was washed with saturated aqueous $NaHCO_3$ solution and extracted with DCM (3 x 50 mL). The combined organic layers were dried over $MgSO_4$, filtered and concentrated under reduced pressure. The crude material was then purified by silica gel flash chromatography (DCM: petroleum ether 1:2); the product containing fractions were combined and concentrated under reduced pressure to give the product as a dark brown solid. Yield: 0.0500 g, 8%. **^{1}H NMR** (400 MHz, $CDCl_3$): δ 9.68 (s, 4H), 7.29 (s, 8H), 3.24 (s, 12H), 2.59 (s, 12H), 2.14 (s, 24H), -3.39 (s, 2H). NMR data is consistent with literature values.[13] **HRMS** (ES^+) calcd. for $[M+H]^+$ $C_{60}H_{63}N_4$: 839.5053, found: 839.5037.

[1,1':3',1''-Terphenyl]-2'-carbaldehyde

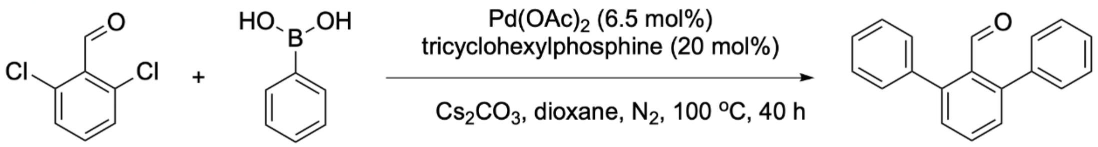

This compound was prepared using a method modified from literature.[14] To 250 mL round bottom flask equipped with a condenser, 2,6-dichlorobenzaldehyde (4.349 g, 25 mmol) was dissolved in anhydrous dioxane (75 mL) and treated, under a nitrogen atmosphere, with cesium carbonate (27.5 g, 3.4 equiv), phenylboronic acid (9.144 g, 75 mmol), $Pd(OAc)_2$ (0.3648 g, 1.625 mmol), and a 20% solution of tricyclohexylphosphine in toluene (1.4015 g, 5 mmol). The reaction mixture was heated at 100 ^{o}C overnight. After being cooled to room temperature, $Pd(OAc)_2$ (0.1800 g, 0.8 mmol), tricyclohexylphosphine (0.5605 g, 2 mmol) and phenylboronic acid (4.572 g, 37.5 mmol) were added. The mixture was heated again at 100 ^{o}C for further 24h. The reaction mixture was then cooled to room temperature, diluted with EtOAc, and filtered through a Celite pad. The organic filtrate was concentrated under vacuum, and the crude product was purified by flash chromatography (EtOAc: petroleum ether = 5:100) to yield the product as a crystalline light yellow solid (3.8715 g, 60% yield). **^{1}H NMR** (400 MHz, $CDCl_3$): δ 9.96 (s, 1H), 7.60 (t, *J* = 7.6 Hz, 1H), 7.60–7.30 (m, 12H). **^{13}C NMR** (101 MHz, $CDCl_3$) δ 193.7, 144.5, 139.8, 133.4, 131.7, 130.5, 129.7, 128.3, 127.8. NMR data is consistent with literature values.[14] **HRMS** (ES^+) calcd. for $[M+H]^+$ $C_{19}H_{15}O$: 259.1117, found: 259.1113.

(*Z*)-2'-(2-Nitroprop-1-en-1-yl)-1,1':3',1''-terphenyl

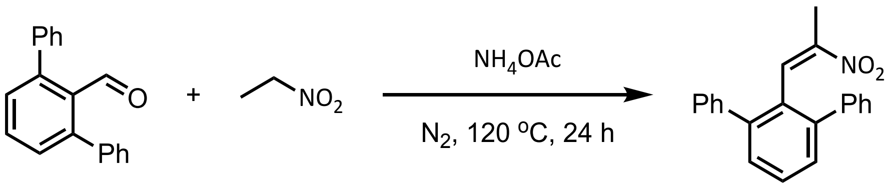

This compound was prepared using a method modified from literature.[12] [1,1':3',1''-Terphenyl]-2'-carbaldehyde (3.8748 g, 15 mmol), ammonium acetate (1.1562 g, 15 mmol) and nitroethane (10 mL, 150 mmol) were added to a 25 mL round bottom flask, which was fitted with a waterless reflux condenser. The reaction mixture was then heated to reflux at 120 °C for 24 h under N_2. When reaction was finished, an orange-brown residue was obtained after removal of solvent under reduced pressure. The crude was purified by column chromatography over silica (ethyl acetate:petroleum ether = 1:20), then the product containing fractions were combined and concentrated under reduced pressure to give the product as light yellow solid. Yield: 3.3088 g, 70%. **^{1}H NMR** (500 MHz, $CDCl_3$): δ 7.89 (s, 1H), 7.53 (t, *J* = 7.8 Hz, 1H), 7.44-7.41 (m, 2H), 7.40-7.31 (m, 6H), 7.30-7.26 (m, 4H), 1.53 (s, 3H). **^{13}C NMR** (126 MHz, $CDCl_3$) δ 148.9, 142.5, 140.6, 133.8, 129.6, 129.4(3C), 128.5, 127.8, 13.8. **HRMS** (ES^+) calcd. for $[M+H]^+$ $C_{21}H_{17}NO_2$: 316.1322, found: 316.1288.

Ethyl 3-([1,1':3',1''-terphenyl]-2'-yl)-4-methyl-1*H*-pyrrole-2-carboxylate

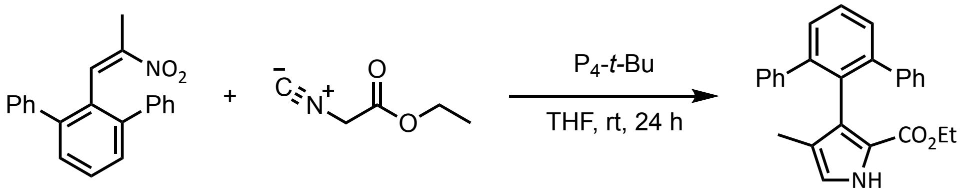

This compound was prepared using a method modified from literature.[12] In a 50 mL round bottom flask, (*Z*)-2'-(2-nitroprop-1-en-1-yl)-1,1':3',1''-terphenyl (1.8900 g, 6 mmol) and ethyl isocyanoacetate (0.6787 g, 6 mmol) were dissolved in dry THF (15 mL). The flask was flushed with nitrogen gas, while cooling in an ice bath before the slow addition of phosphazene base P_4-*t*-Bu solution (10 mL, 8 mmol, ~0.8 M in hexane). Then the reaction mixture was left to stir at room temperature for 24 h. When reaction was finished, a brown residue was obtained after removal of solvent under reduced pressure. The crude product was purified by column chromatography over silica (ethyl acetate:petroleum ether = 1:5), then the product containing fractions were combined and concentrated under reduced pressure to give the product as a beige solid. Yield: 1.3928 g, 46%. **^{1}H NMR** (500 MHz, $CDCl_3$): δ 8.56 (s, 1H), 7.45 (dd, *J* = 8.4, 6.8 Hz, 1H), 7.39-7.35 (m, 2H), 7.19-7.08 (m, 10H), 6.43 (d, *J* = 3.0 Hz, 1H), 4.03 (q, *J* = 7.1 Hz, 2H), 1.56 (s, 3H), 1.05 (t, *J* = 7.1 Hz, 3H). ^{13}C NMR (126 MHz, $CDCl_3$) δ 161.0, 142.8, 142.5, 132.3, 129.2, 128.9, 128.8, 127.3, 127.4, 126.3, 121.5, 120.6, 119.9, 59.8, 14.2, 10.6. **HRMS** (ES^+) calcd for $[M+H]^+$ $C_{26}H_{24}N_2O$: 382.1802, found: 382.1790.

2,7,12,17-Tetra([1,1':3',1''-terphenyl]-2'-yl)-3,8,13,18-tetramethylporphyrin (L6)

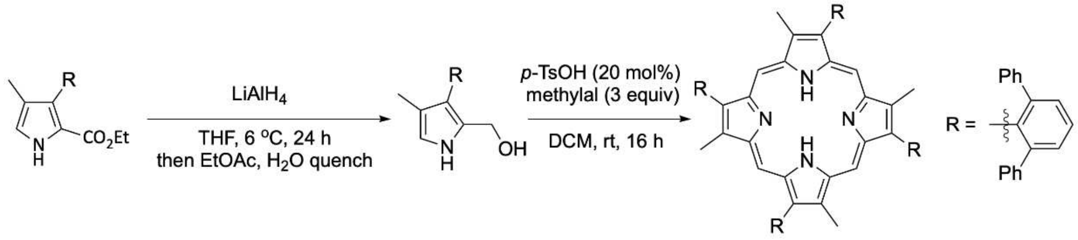

This compound was prepared using a method modified from literature.[12] To a 25 mL round bottom flask, ethyl 3-([1,1':3',1''-terphenyl]-2'-yl)-4-methyl-1*H*-pyrrole-2-carboxylate (0.2400 g, 0.63 mmol) was dissolved in dry THF (3 mL) and the flask flushed with nitrogen before being placed in an ice bath. While stirring, a solution of $LiAlH_4$ in THF (2.5 mL, 6 mmol, 2.4 M) was added slowly to the dissolved ester *via* syringe over 10 minutes. The reaction was placed in the fridge at 6 °C without stirring for 24 hours before quenching with ethyl acetate (5 mL) and water (0.5 mL) to deactivate excess $LiAlH_4$. The reaction mixture was extracted with NH_4Cl (aq) and EtOAc, the combined organic phases were dried using $MgSO_4$, and evaporated under reduced pressure. Then the residue was dissolved in DCM (10 mL) with the addition of methylal (3 equiv., 0.14 mL) and *p*-TsOH (20 mol%, 0.0243 g). The reaction mixture was stirred at room temperature overnight. *p*-Chloranil (0.4917 g, 2 mmol) was then added in powder form and the reaction mixture was gently refluxed for 8 h. After removal of the solvent under reduced pressure, the reaction mixture was purified by silica gel flash chromatography (DCM: petroleum ether 1:4); the product containing fractions were combined and concentrated under reduced pressure to give the product as dark red solid. Yield: 0.1445 g, 72%. **^{1}H NMR** (500 MHz, $CDCl_3$): 9.28 (s, 4H), 7.75 (dd, J = 8.7, 7.6 Hz, 4H), 7.75-7.71 (m, 8H), 7.09-7.03 (m, 16H), 6.69-6.61 (m, 8H), 6.57 (t, J = 7.5 Hz, 16H), 2.87 (s, 12H), -4.2 (s, 2H). NMR data is consistent with literature values.[12] **HRMS** (ES^+) calcd for $[M+H]^+$ $C_{96}H_{71}N_4$:1279.5673, found: 1279.5579.

(1-Cyclopropylvinyl)benzene

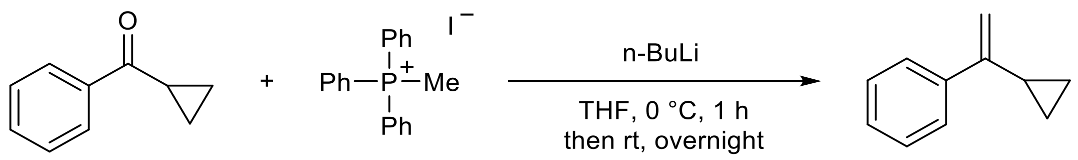

This compound was prepared using a method modified from literature.[15] Methyltriphenylphosphonium iodide (6.6 g, 15 mmol) were placed in a 50 mL two-necked reaction flask, and the flask was flushed with nitrogen. After THF (15 mL) and n-BuLi (2.5 M hexane solution, 6.6 mL, 1.1 equiv) were added at 0 °C, the mixture was stirred at room temperature for 1 hour. A solution of cyclopropyl(phenyl)methanone (2.19 g, 8.0 mmol) in THF (4.0 mL) was then added, and the mixture was stirred for additional 12 h. After completion (TLC), the resulting mixture was quenched by slow addition of isopropanol and water before extracted with ethyl acetate, and the combined organic layer was dried over sodium sulfate. After evaporation in vacuo, purification by column chromatography on silica gel with hexane/ethyl acetate (40:1, v/v) to give (1-cyclopropylvinyl)benzene as colorless oil. Yield: 1.82 g, 85%. **^{1}H NMR** (400 MHz, $CDCl_3$) δ 7.62 - 7.57 (m, 2H), 7.37 – 7.25 (m, 3H), 5.28 (1s, 1H), 4.94 (1s, 1H), 1.69-1.62 (m, 1H), 0.86-0.82 (m, 2H), 0.62-0.58 (m, 2H); **^{13}C NMR** (101 MHz, $CDCl_3$) δ 149.5, 181.8, 128.3, 127.6, 126.3, 109.2, 15.8, 6.8. NMR data is consistent with literature values. [15] HRMS (APCI) calcd. for $[M]^+$ $C_{11}H_{12}$: 144.0944, found: 145.0942.

2-Cyclopropyl-2-phenylacetaldehyde

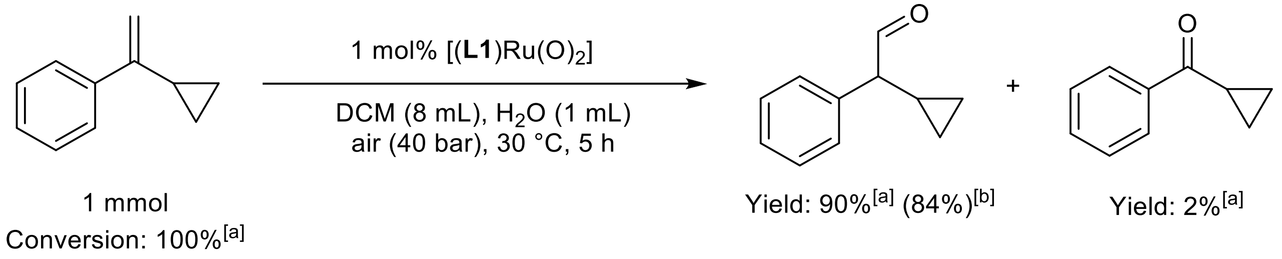

[a] Yield and conversion were determined by ^{1}H NMR with mesitylene as internal standard
[b] Isolated yield

Reaction was carried out following Supplementary Method A. The major aldehyde product was purified by column chromatography over silica gel with ethyl acetate/petroleum ether (1/10, v/v) to give the product (0.1361 g, 84%) as a yellow liquid. **^{1}H NMR** (400 MHz, $CDCl_3$) δ 9.75 (d, *J* = 2.6 Hz, 1H), 7.41-7.35 (m, 2H), 7.34-7.24 (m, 3H), 2.80 (dd, *J* = 9.6, 2.6 Hz, 1H), 1.37-1.27 (m, 1H), 0.81-0.55 (m, 1H), 0.30 (m, 2H), 0.30 (m, 2H). **^{13}C NMR** (101 MHz, $CDCl_3$) δ 200.5, 136.4, 129.0, 128.8, 127.7, 63.4, 11.1, 4.7, 3.5. NMR data is consistent with literature values.[16] HRMS (APCI) calcd. for $[M]^+$ $C_{11}H_{11}O$: 160.0894, found: 160.0846.

Synthesis of [(Ln)Ru(CO)] complexes

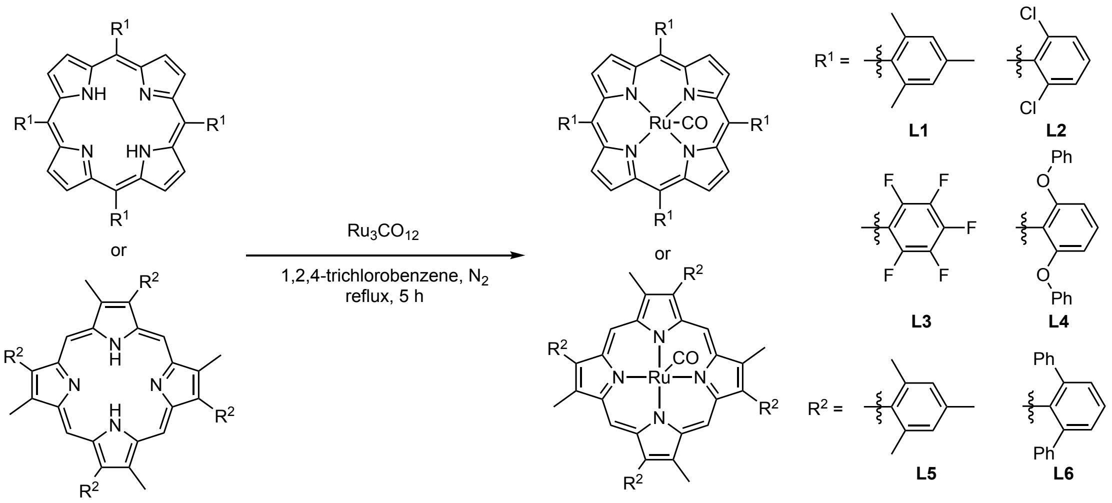

In a 50 mL two-neck round-bottom flask containing 1,2,4-trichlorobenzene (20 mL), triruthenium dodecacarbonyl (0.3017 g, 0.48 mmol) and porphyrin ligand (~0.3 g, 0.4 mmol) were added. The flask was fitted with a waterless reflux condenser and flushed with N_2 for 10-15 minutes. Then the reaction mixture was heated to 220 ^{o}C in oil bath and allowed to stir for 5 h under continuous nitrogen gas flow. After cooling to room temperature, petroleum ether (20 mL) was added to the reaction mixture which was loaded on a neutral alumina column. Trichlorobenzene was removed by washing with petroleum ether (approx. 250 mL) and the product, typically a red-orange band, was then eluted with DCM (approx. 500 mL). The solvent was then evaporated and the deep-red product was dried under high vacuum.

[(L1)Ru(CO)] Dark red solid. Yield: 0.3059 g, 84%. **^{1}H NMR** (500 MHz, $CDCl_3$) δ 8.47 (s, 8H), 7.26 (s, 8H), 2.61 (s, 12H), 1.92 (s, 12H), 1.83 (s, 12H). NMR data is consistent with literature values.[17] **HRMS** (ES$^+$) calcd. for $[M+H]^+$ $C_{57}H_{56}N_5ORu$: 928.3532 found: 928.3538.

[(L2)Ru(CO)] Red solid. Yield: 0.2028 g, 50%.**^{1}H NMR** (500 MHz, $CDCl_3$) δ 8.50 (s, 8H), 7.80 – 7.74 (m, 8H), 7.67 (dd, *J* = 8.1, 8.1 Hz, 4H). NMR data is consistent with literature value.[17] **HRMS** (ES$^+$) calcd. for $[M+NH_4]^+$ $C_{45}H_{24}Cl_8N_5ORu$: 1035.8487 found: 1035.8513.

[(L3)Ru(CO)] Dark red solid. **^{1}H NMR** (500 MHz, $CDCl_3$) δ 8.71 (s, 8H). ^{19}F-NMR (470 MHz, $CDCl_3$) δ -136.0 (d, *J* = 20.5 Hz, 4F), -138.0 (d, *J* = 20.5 Hz, 4F), -152.0 (t, *J* = 20.1 Hz, 4F), -161.3 (t, *J* = 18.0 Hz, 4F), -162.0 (t, *J* = 18.0 Hz,

4F). NMR data is consistent with literature value.[18] **HRMS** (ES^+) calcd. for $[M+H]^+$ $C_{45}H_{13}F_{20}N_4ORu$: 1120.9851 found: 1120.9569.

[(L4)Ru(CO)] Dark red solid. Yield: 0.3680 g, 62%. **^{1}H NMR** (500 MHz, $CDCl_3$) δ 8.61 (s, 1H), 7.66 (t, *J* = 8.4 Hz, 4H), 7.27 (d, *J* = 8.3 Hz, 4H), 7.14 (d, *J* = 8.3 Hz, 4H), 6.59 (t, *J* = 7.8 Hz, 1H), 6.45 (d, *J* = 8.1 Hz, 8H), 6.42-6.31 (m, 20 H), 6.16-6.07 (m, 4H). **HRMS** (ES^+) calcd. for $[M+NH_4]^+$ $C_{93}H_{64}N_5O_9Ru$: 1495.3757 found: 1495.3672.

[(L5)Ru(CO)] Dark purple solid. Yield: 0.1584 g, 41%.**^{1}H NMR** (400 MHz, $CDCl_3$) δ 9.53 (s, 4H), 7.30 (s, 8H), 3.18 (s, 12H), 2.58 (s, 12H), 2.29 (s, 24H). **HRMS** (ES^+) calcd. for $[M]^+$ $C_{61}H_{60}N_4ORu$: 966.3822 found: 966.3906.

[(L6)Ru(CO)] Dark purple solid. Yield: 0.2985 g, 53%. ^{1}H NMR (500 MHz, $CDCl_3$) δ 8.89 (s, 4H), 7.63 (m, 12H), 6.99 (m, 20H), 6.34 (m, 20H), 2.61 (s, 12H). NMR data is consistent with literature value.[6] **HRMS** (ES^+) calcd. for $[M]^+$ $C_{97}H_{68}N_4ORu$: 1406.4436 found: 1406.4398.

Synthesis of [(Ln)Ru(O)$_2$] complexes

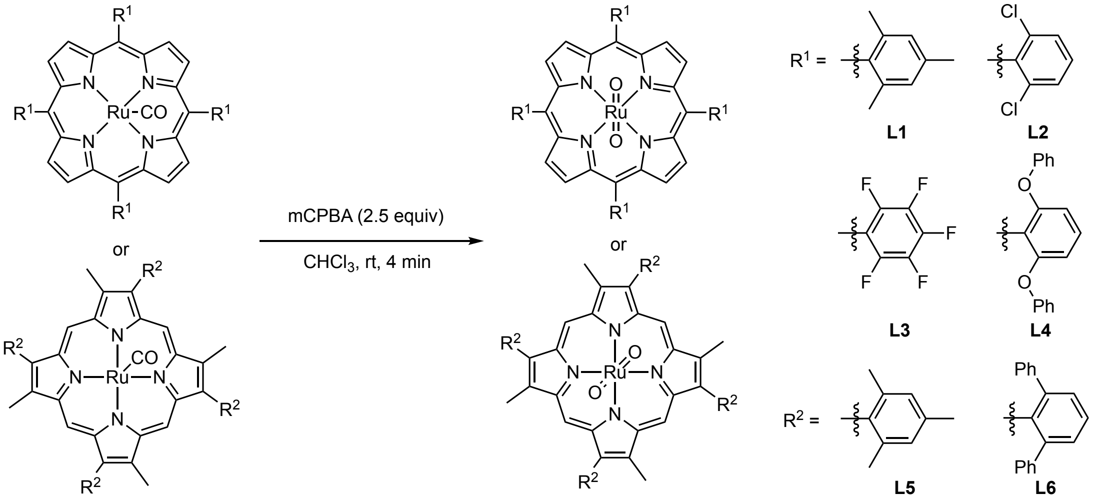

To a 50 mL round bottom flask, [(**Ln**)Ru(CO)] complex (~0.25 g, 0.25 mmol) was added and dissolved in chloroform (20 mL, amylene stabilized). At room temperature, *m*-CPBA (0.1540 g, 0.625 mmol, dissolved in 5 mL of chloroform) was added to the flask and the reaction mixture was allowed to stir for 4 minutes. The reaction mixture in the flask was then quickly passed through a basic alumina plug, washed with DCM (approx. 200 mL). A dark purple fraction was collected and DCM was removed under reduced pressure to give the product as purple solid, which was then transferred to a sample vial shielded from light with aluminum foil and further dried under high vacuum.

[(L1)Ru(O)$_2$] Purple solid. Yield: 0.1360 g, 60%. **^{1}H NMR** (500 MHz, $CDCl_3$) δ 8.81 (s, 8H), 7.31 (s, 8H), 2.65 (s, 12H), 1.90 (s, 24H). NMR data is consistent with literature values.[19] **HRMS** (ES$^+$) calcd. for $[M+H]^+$ $C_{56}H_{53}N_4O_2Ru$: 915.3222 found: 915.3159.

[(L2)Ru(O)$_2$] Purple solid. Yield: 0.1272 g, 50%.**^{1}H NMR** (400 MHz, $CDCl_3$) δ 8.89 (s, 8H), 7.86-7.82 (m, 8H), 7.77-7.73 (m, 4H). NMR data is consistent with literature value.[20] **HRMS** (ES$^+$) calcd. for $[M-O+H]^+$ $C_{44}H_{21}Cl_8N_4ORu$: 1002.8267 found: 1002.8225.

[(L3)Ru(O)$_2$] Purple solid. Yield: 0.0859 g, 31%. **^{1}H NMR** (500 MHz, $CDCl_3$) δ 9.19 (s, 8H).^{19}F-NMR (470 MHz, $CDCl_3$) δ -135.5 (m, 8F), -150.3 (t, *J* = 21.0 Hz, 4F), -160.7 (td, *J* = 21.0, 6.9 Hz, 8F). NMR data is consistent with literature value.[18] **HRMS** (ES$^+$) calcd. for $[M+3H]^+$ $C_{44}H_{11}F_{20}N_4O_2Ru$: 1108.9612 found: 1108.9697.

[(L4)Ru(O)$_2$] Purple solid. Yield: 0.2434 g, 66%. **^{1}H NMR** (500 MHz, $CDCl_3$) δ 9.00 (s, 8H), 7.70 (t, *J* = 8.4 Hz, 4H), 7.21 (d, *J* = 8.4 Hz, 8H), 6.44 (dd, *J* = 8.7, 7.2 Hz, 16H), 6.41-6.35 (m, 16H), 6.23 (t, *J* = 7.2 Hz, 8H). **HRMS** (ES^+) calcd. for $[M+2H]^+$ $C_{92}H_{62}N_4O_{10}Ru$: 1484.3530 found 1484.3453.

[(L5)Ru(O)$_2$] Purple solid. Yield: 0.1430 g, 43%.^{1}H NMR (400 MHz, $CDCl_3$ δ 10.12 (s, 4H), 7.36-7.32 (m, 8H), 3.39 (s, 12H), 2.62 (s, 12H), 2.25 (s, 24H). **HRMS** (ES^+) calcd. for $[M-O+H]^+$ $C_{60}H_{61}N_4ORu$: 955.3900 found: 955.3903.

[(L6)Ru(O)$_2$] Purple solid. Yield: 0.2609 g, 74%. ^{1}H NMR (500 MHz, $CDCl_3$) δ 9.64 (s, 4H), 7.94-7.74 (m, 12H), 7.30-7.26 (m, 12 H), 7.18-6.99 (m, 12H), 6.50-6.38 (m, 16H), 2.98 (s, 12H). NMR data is consistent with literature value.[6] **HRMS** (ES^+) calcd. for $[M+2H]^+$ $C_{96}H_{70}N_4O_2Ru$: 1412.4542 found: 1412.4600.

4 Product Characterization data

2-(4-Nitrophenyl)oxirane (9a)

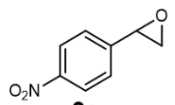

9a

Purified by column chromatography over silica (ethyl acetate/petroleum ether = 20%) to give the product (0.0735 g, 89%) as a white solid. **^{1}H NMR** (500 MHz, $CDCl_3$) δ 8.22 (d, *J* = 8.8 Hz, 2H), 7.45 (d, *J* = 8.7 Hz, 2H), 3.96 (dd, *J* = 4.1 Hz, 2.5 Hz, 1H), 3.23 (dd, *J* = 5.5 Hz, 4.1 Hz, 1H), 2.78 (dd, *J* = 5.5 Hz, 2.5 Hz, 1H). **^{13}C NMR** (126 MHz, $CDCl_3$) δ 148.0, 145.4, 126.4, 124.0, 51.8, 51.6. NMR data is consistent with literature values[21]. **HRMS** (EI^+) calcd. for $[M+H]^+$ $C_8H_8NO_3$: 166.0499 found: 166.0553.

2,3-Diphenyloxirane (15a)

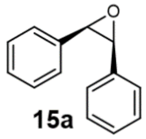

15a

Purified by column chromatography over silica (ethyl acetate/petroleum ether = 5%) to give the product (0.0882 g, 90%) as a white solid.**^{1}H NMR** (500 MHz, $CDCl_3$) δ 7.23-7.10 (m, 10 H), 4.37 (s, 2H). **^{13}C NMR** (126 MHz, $CDCl_3$) δ 134.5, 127.9, 127.6, 127.0, 59.9. NMR data is consistent with literature values[21]. **HRMS** (EI^+) calcd. for $[M+H]^+$ $C_{14}H_{13}O$: 197.0961 found: 197.0976.

(4a*S*,5a*R*,9a*R*,11b*R*)-9a,11b-Dimethyl-9-(6-methylheptan-2-yl) hexadecahydrocyclopenta [1,2]phenanthro[8a,9-*b*]oxiren-3-yl acetate (18a/a')

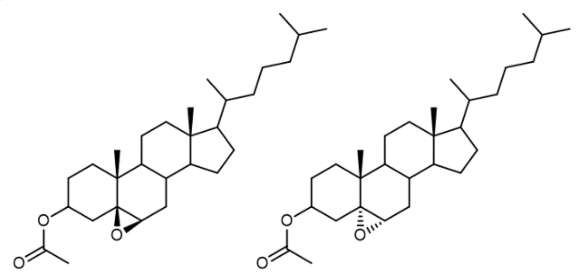

Purified by column chromatography over silica (ethyl acetate/petroleum ether = 5%) to give the product (0.1923 g, 92%) as a white solid. β- and α epoxides could not be separated but were identified on the basis of the comparison of the ^{1}H NMR spectra. The integration of the two signals belonging to H-6 gave a 99:1 β/α ratio. Spectroscopic data corresponds to the major β isomer. **^{1}H NMR** (500 MHz, $CDCl_3$) δ 4.84-4.71 (m, 1H), 3.07 (d, *J* = 2.5 Hz, 1H), 2.15-2.04 (m, 2H), 2.03 (s, 3H), 1.99-1.92 (m, 2H), 1.86-1.76 (m, 1H), 1.62-1.17 (m, 14 H), 1.16-1.02 (m, 6H), 1.00 (s, 3H), 0.92-0.82 (m, 11H), 0.64 (s, 3H). **^{13}C NMR** (126 MHz, $CDCl_3$) δ 170.6, 71.4, 63.6, 62.5, 56.2, 51.0, 42.3, 39.8, 39.5, 38.0,36.7, 36.1, 35.7, 35.0, 32.5, 29.7, 28.2, 28.0, 27.2, 24.2, 23.8, 22.8, 22.6, 21.9, 21.3, 18.7, 17.0, 11.8. NMR data is consistent with literature values[22]**.** **HRMS** (EI^{+}) calcd. for $[M+Na]^{+}$ $C_{29}H_{48}O_3Na$: 467.3496 found: 467.3494.

5 ESI-MS data of Ru complexes and their simulated spectra

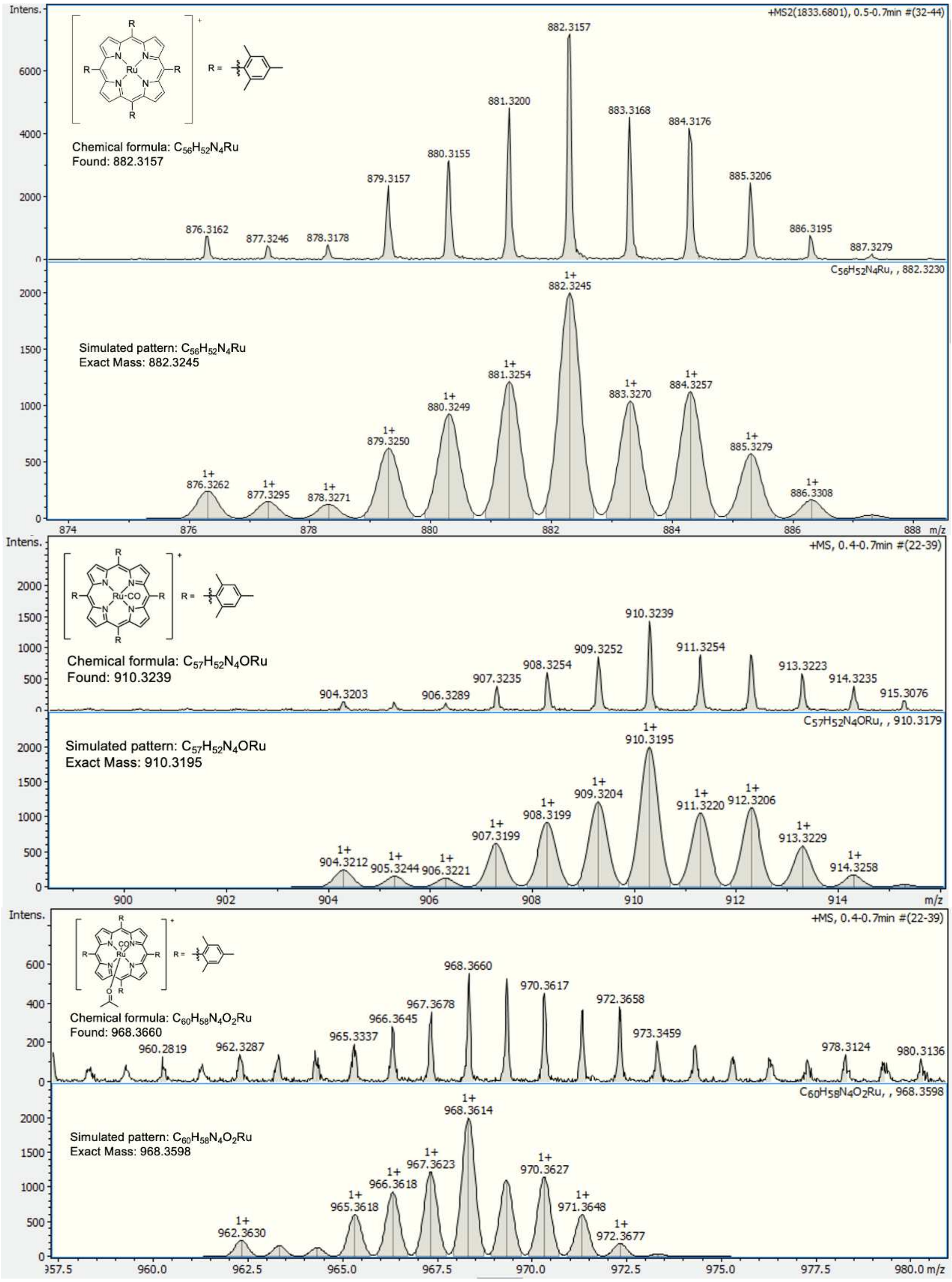

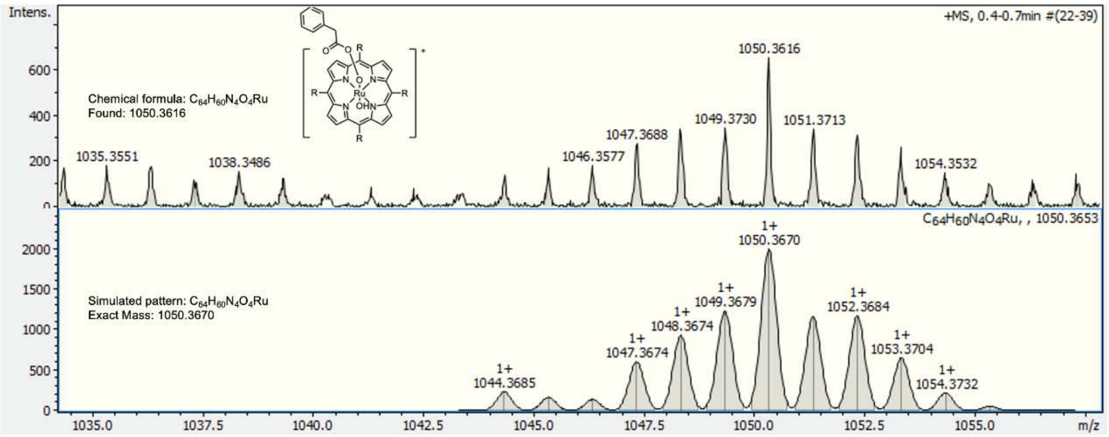
Chemical formula: $C_{64}H_{60}N_4O_4Ru$
Found: 1050.3616
Simulated pattern: $C_{64}H_{60}N_4O_4Ru$
Exact Mass: 1050.3670
+MS, 0.4-0.7min #(22-39)
$C_{64}H_{60}N_4O_4Ru$, , 1050.3653
m/z

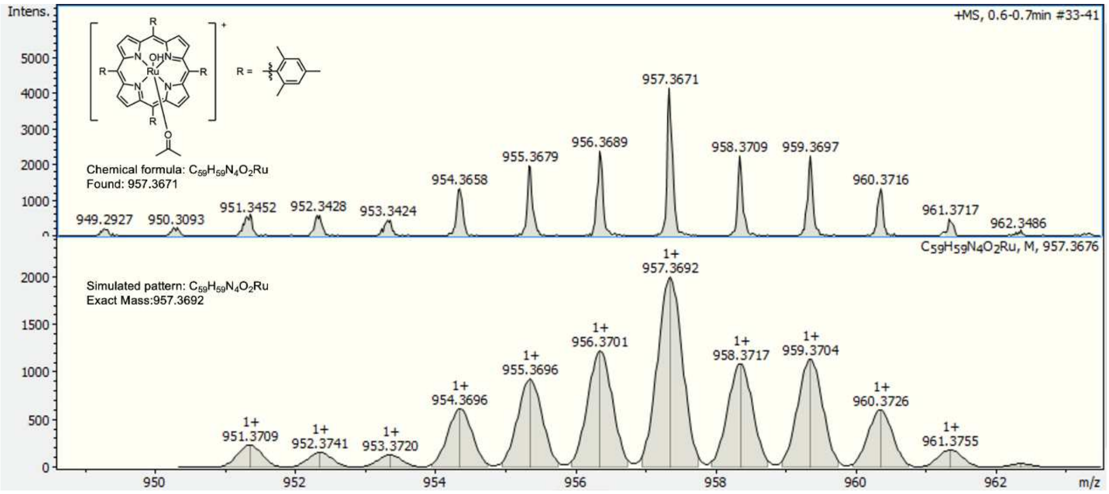
Chemical formula: $C_{59}H_{59}N_4O_2Ru$
Found: 957.3671
Simulated pattern: $C_{59}H_{59}N_4O_2Ru$
Exact Mass:957.3692
+MS, 0.6-0.7min #33-41
$C_{59}H_{59}N_4O_2Ru$, M, 957.3676
m/z

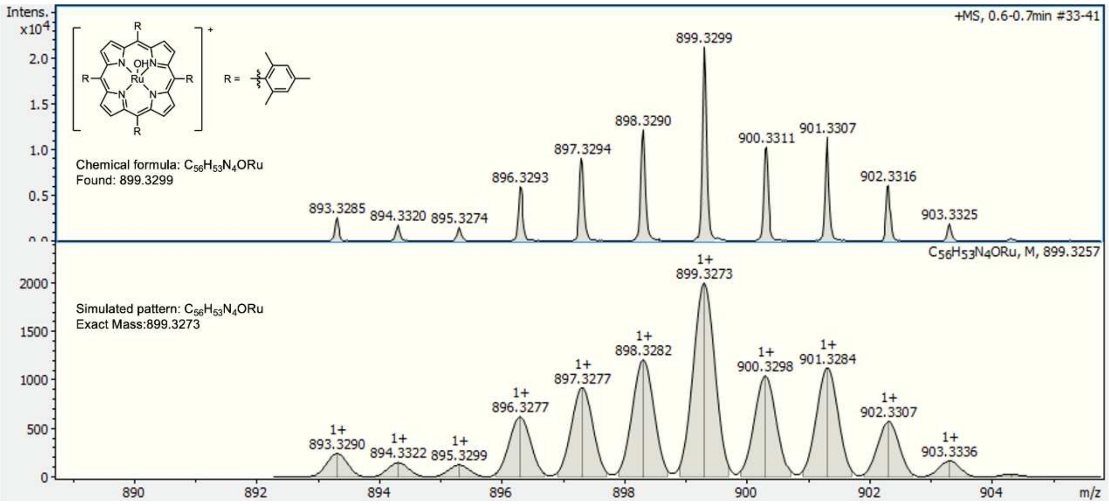
Chemical formula: $C_{56}H_{53}N_4ORu$
Found: 899.3299
Simulated pattern: $C_{56}H_{53}N_4ORu$
Exact Mass:899.3273
+MS, 0.6-0.7min #33-41
$C_{56}H_{53}N_4ORu$, M, 899.3257
m/z

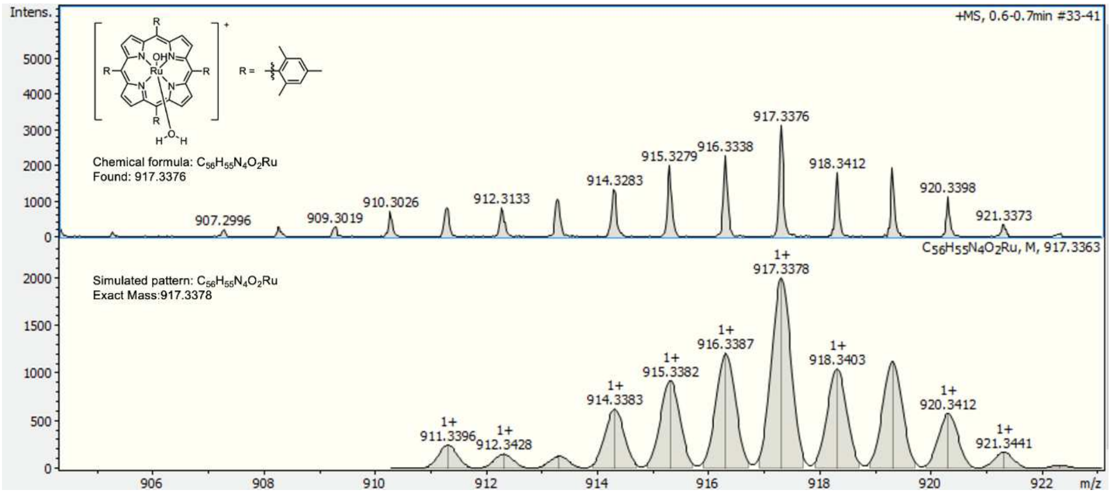
Chemical formula: $C_{56}H_{55}N_4O_2Ru$
Found: 917.3376
Simulated pattern: $C_{56}H_{55}N_4O_2Ru$
Exact Mass:917.3378
+MS, 0.6-0.7min #33-41
$C_{56}H_{55}N_4O_2Ru$, M, 917.3363
m/z

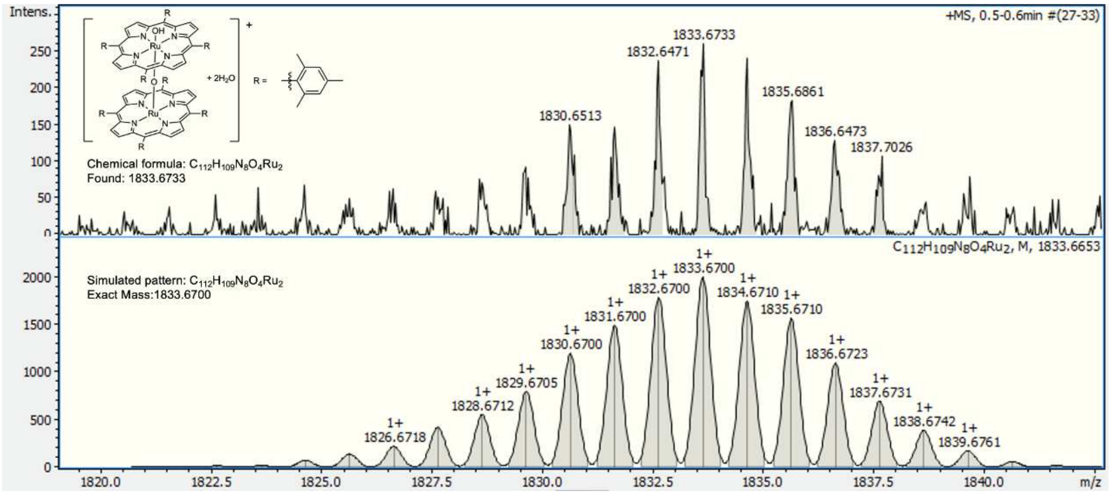
Chemical formula: $C_{112}H_{109}N_8O_4Ru_2$
Found: 1833.6733
Simulated pattern: $C_{112}H_{109}N_8O_4Ru_2$
Exact Mass:1833.6700
+MS, 0.5-0.6min #(27-33)
$C_{112}H_{109}N_8O_4Ru_2$, M, 1833.6653
m/z

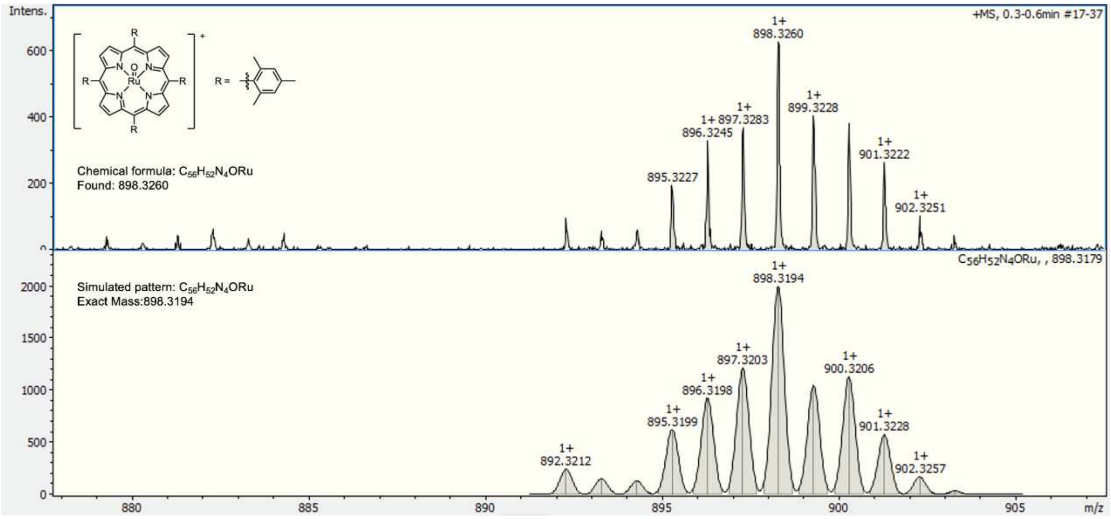
Chemical formula: $C_{56}H_{52}N_4ORu$
Found: 898.3260
Simulated pattern: $C_{56}H_{52}N_4ORu$
Exact Mass:898.3194
+MS, 0.3-0.6min #17-37
$C_{56}H_{52}N_4ORu$, , 898.3179
m/z

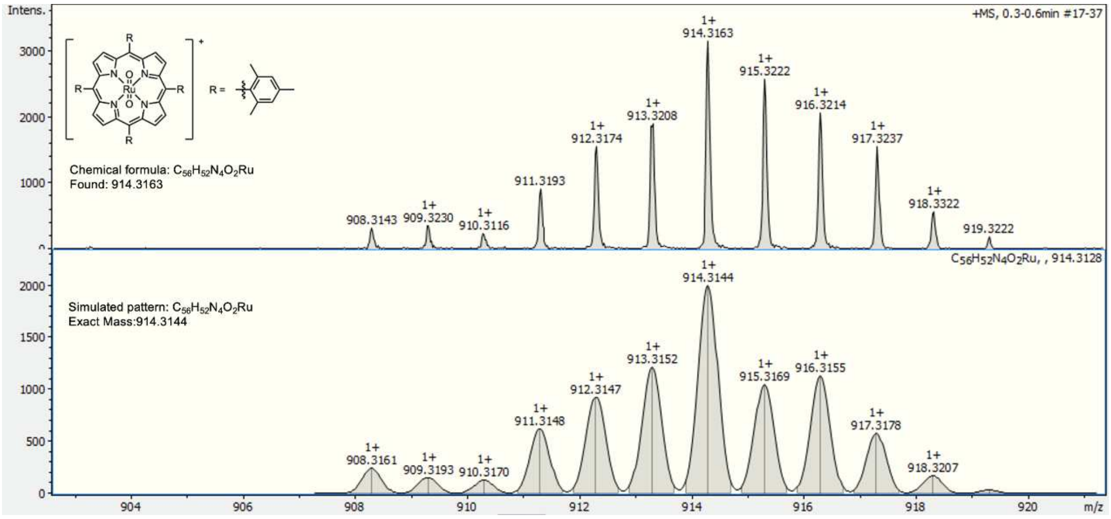
Intens.
+MS, 0.3-0.6min #17-37
R =
Chemical formula: C56H52N4O2Ru
Found: 914.3163
908.3143
909.3230
910.3116
911.3193
912.3174
913.3208
914.3163
915.3222
916.3214
917.3237
918.3322
919.3222
C56H52N4O2Ru, , 914.3128
Simulated pattern: C56H52N4O2Ru
Exact Mass:914.3144
908.3161
909.3193
910.3170
911.3148
912.3147
913.3152
914.3144
915.3169
916.3155
917.3178
918.3207
m/z

6 NMR data

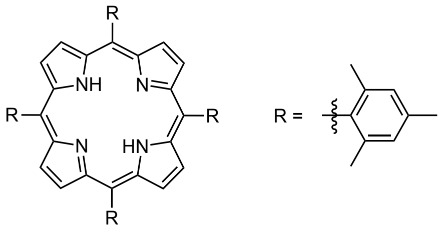
R
NH
N
HN
R =

L1

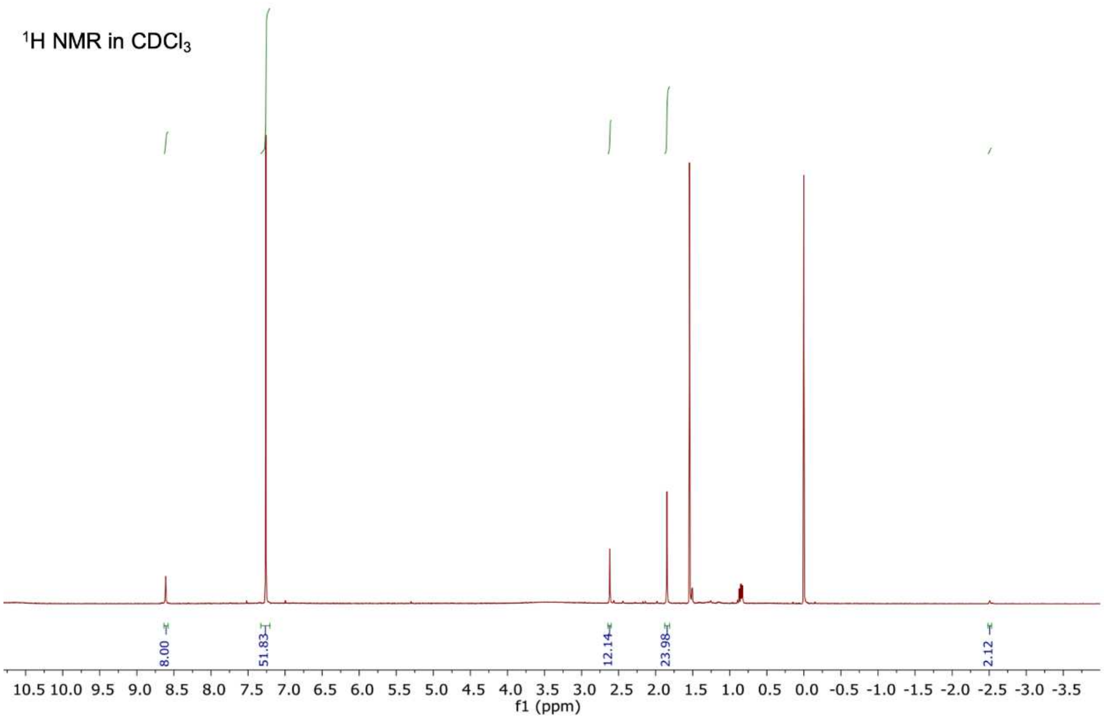
^{1}H NMR in CDCl$_3$
8.00
51.83
12.14
23.98
2.12
10.5 10.0 9.5 9.0 8.5 8.0 7.5 7.0 6.5 6.0 5.5 5.0 4.5 4.0 3.5 3.0 2.5 2.0 1.5 1.0 0.5 0.0 -0.5 -1.0 -1.5 -2.0 -2.5 -3.0 -3.5
f1 (ppm)

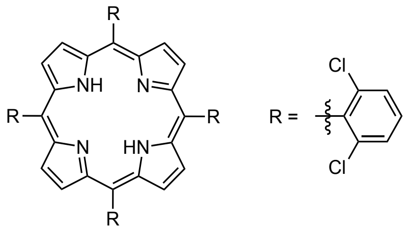
R
NH
N
HN
R =
Cl
Cl

L2

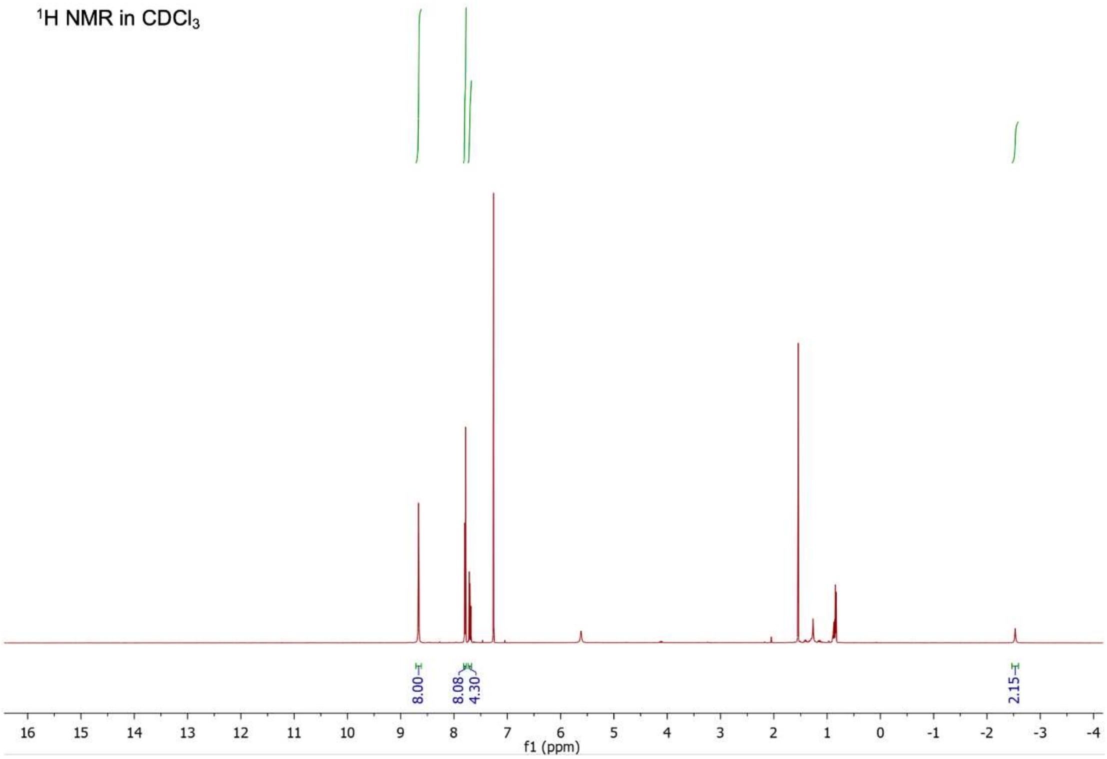
^{1}H NMR in $CDCl_3$
8.00
8.08
4.30
2.15
16 15 14 13 12 11 10 9 8 7 6 5 4 3 2 1 0 -1 -2 -3 -4
f1 (ppm)

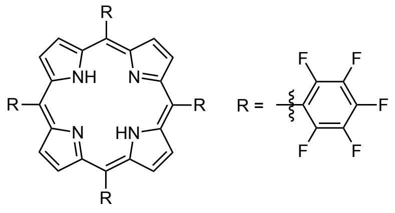

L3

^{1}H NMR in $CDCl_3$

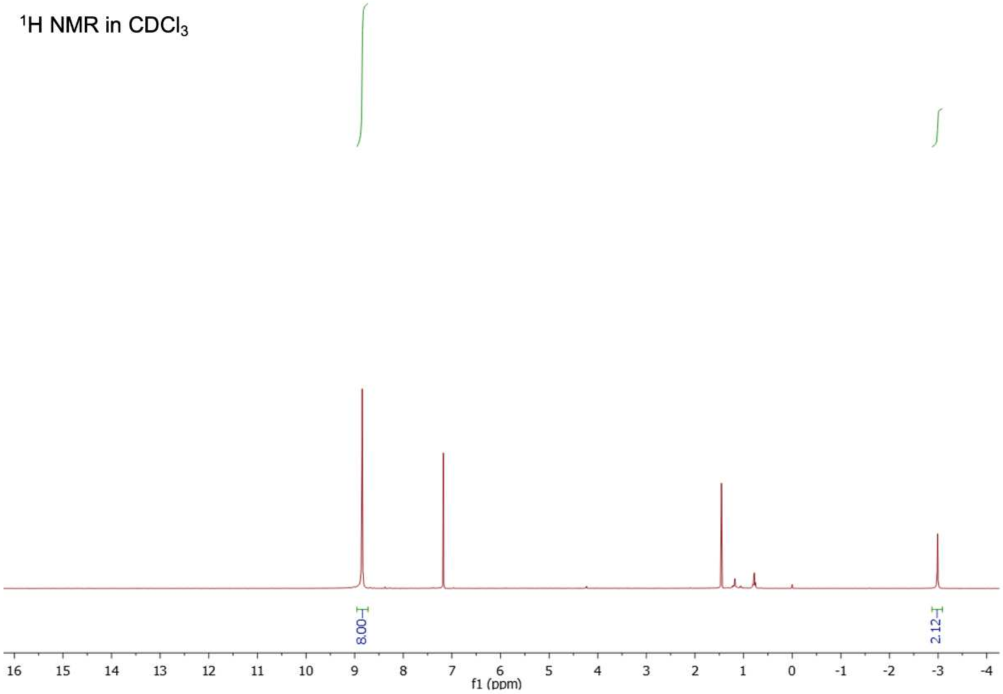

^{19}F NMR in $CDCl_3$

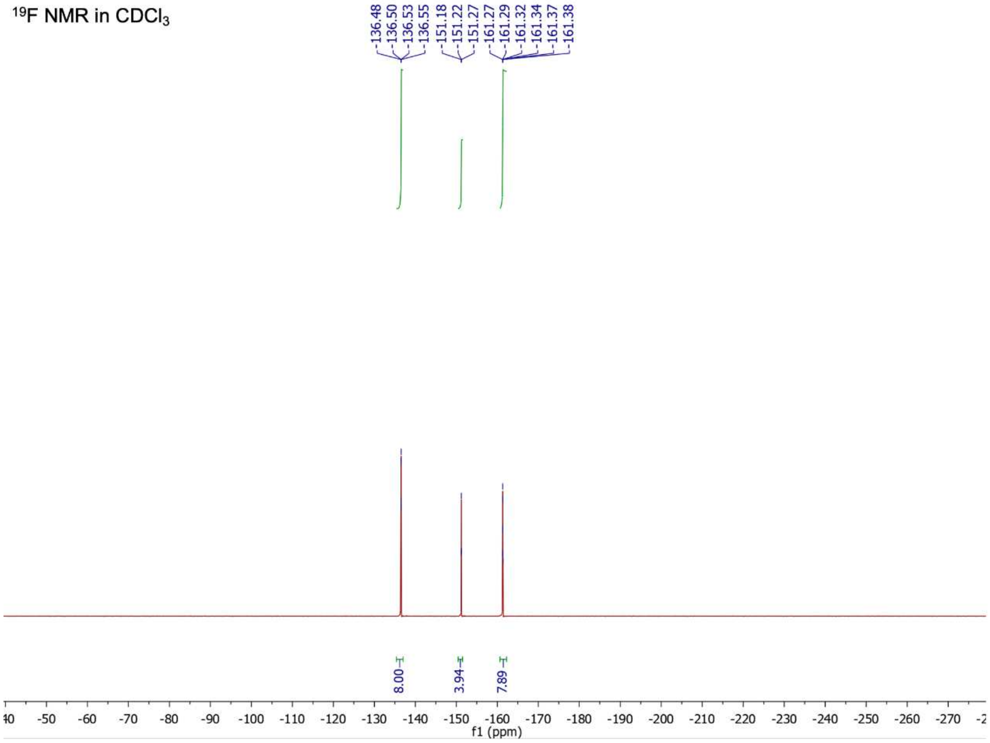

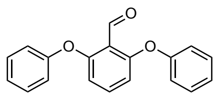

¹H NMR in $CDCl_3$

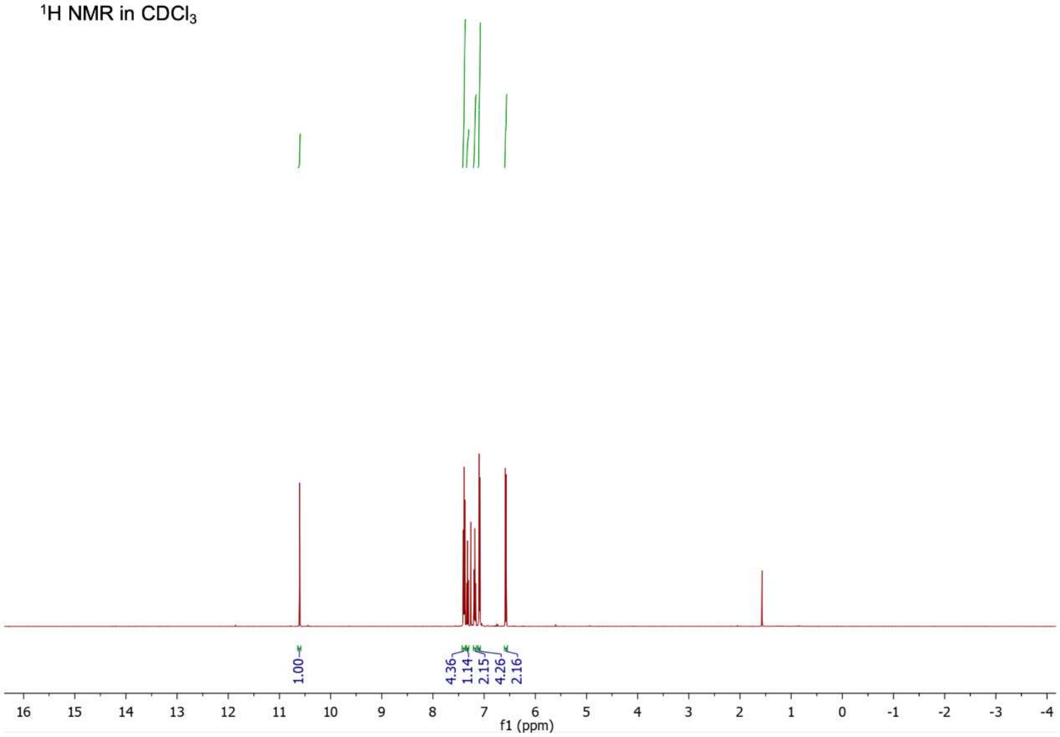

^{13}C NMR in $CDCl_3$

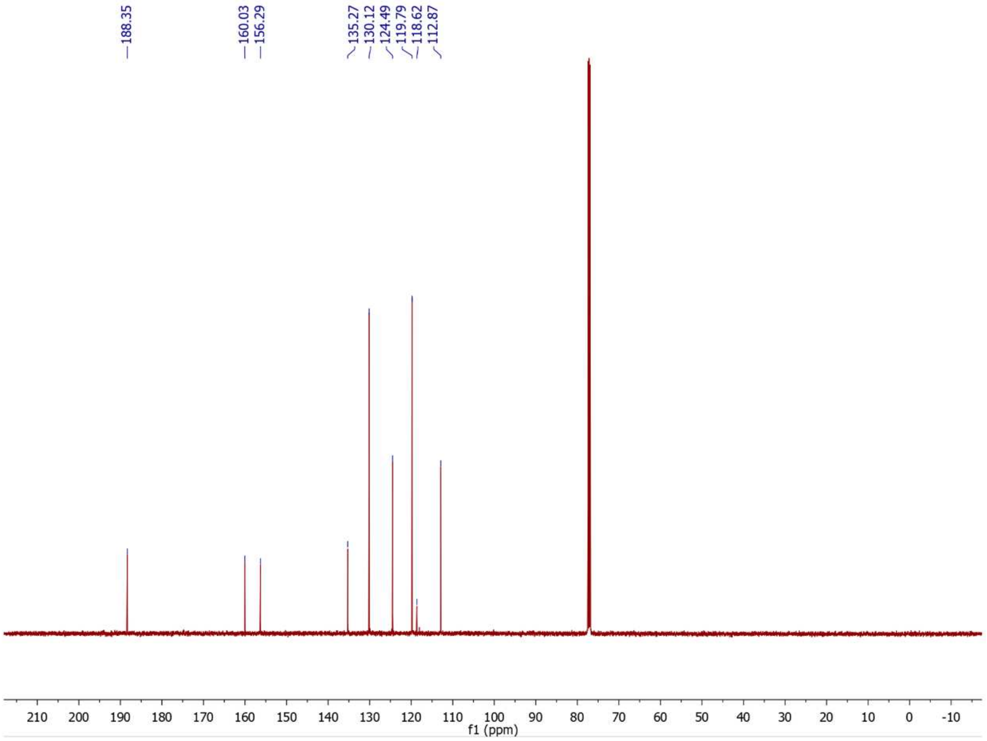

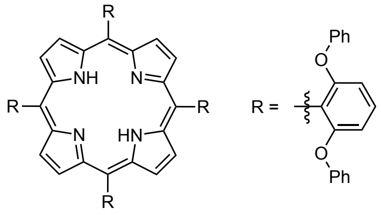
R
NH
N
R
R
N
HN
R
R =
O
Ph
O
Ph

L4

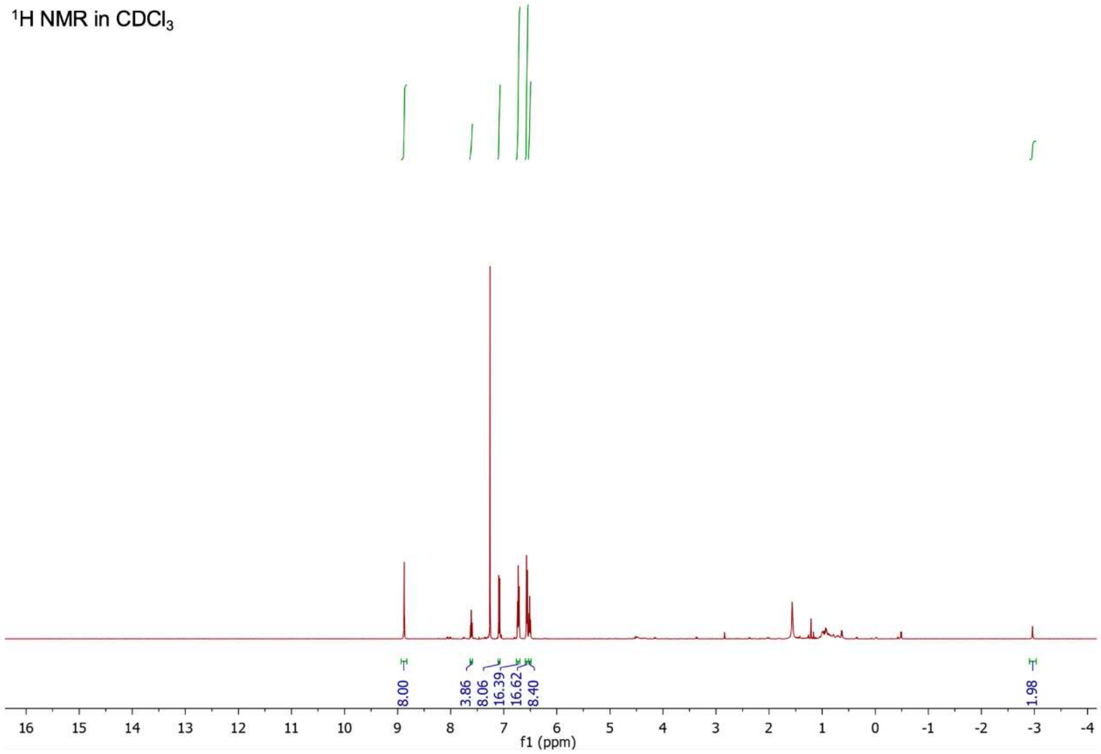
^{1}H NMR in $CDCl_3$
8.00
3.86
8.06
16.39
16.62
8.40
1.98
f1 (ppm)

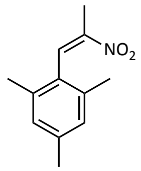

^{1}H NMR in $CDCl_3$

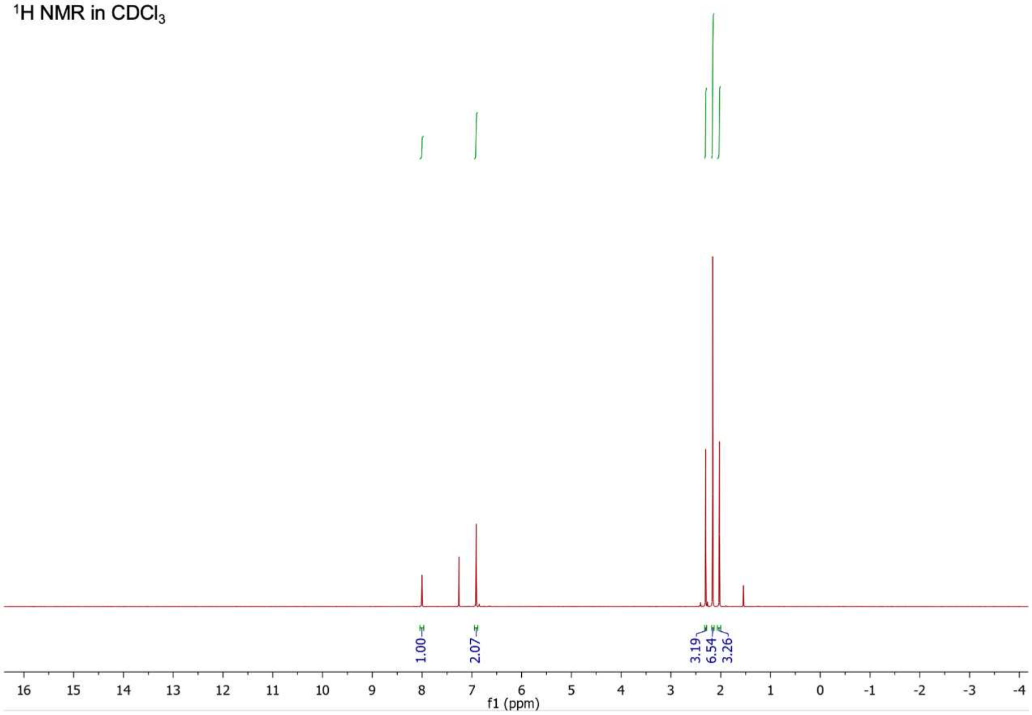

^{13}C NMR in $CDCl_3$

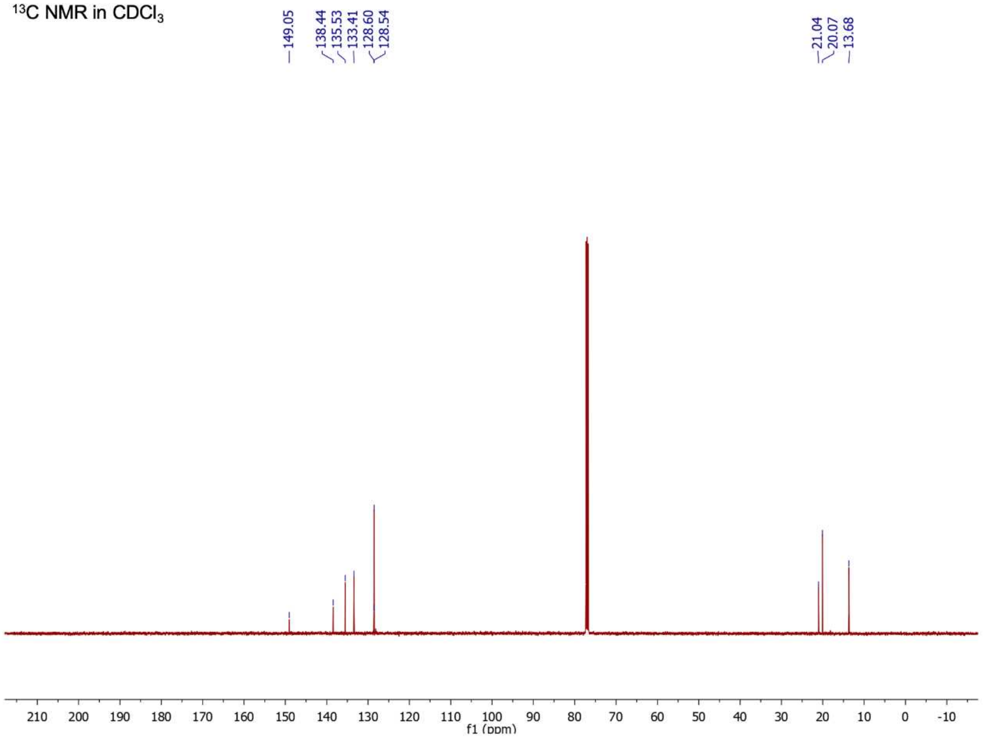

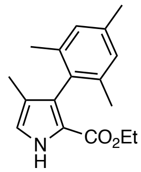

^{1}H NMR in $CDCl_3$

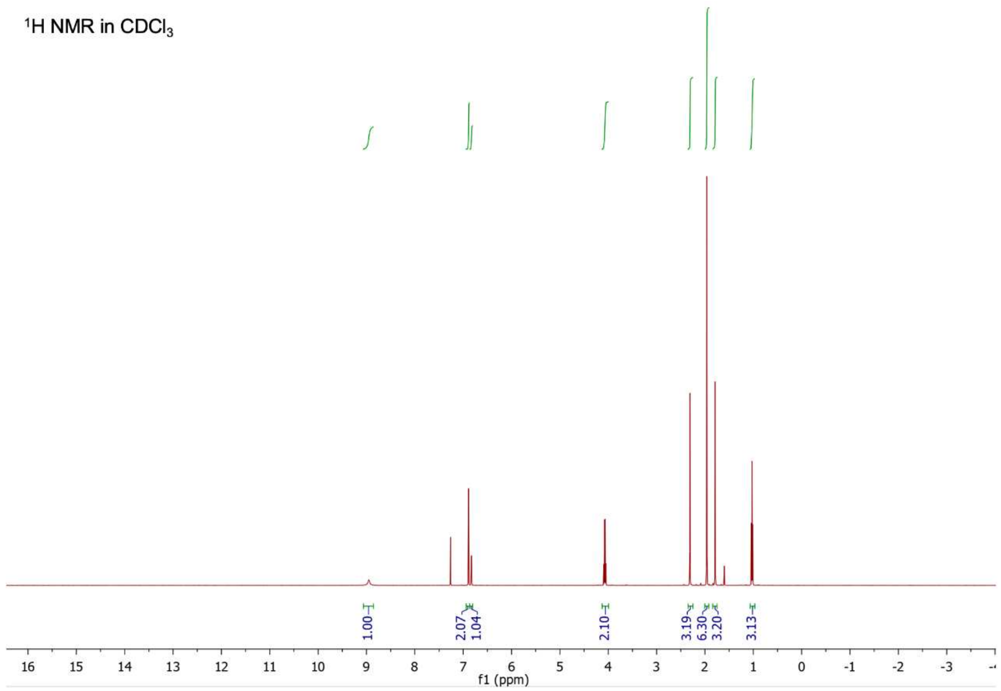

^{13}C NMR in $CDCl_3$

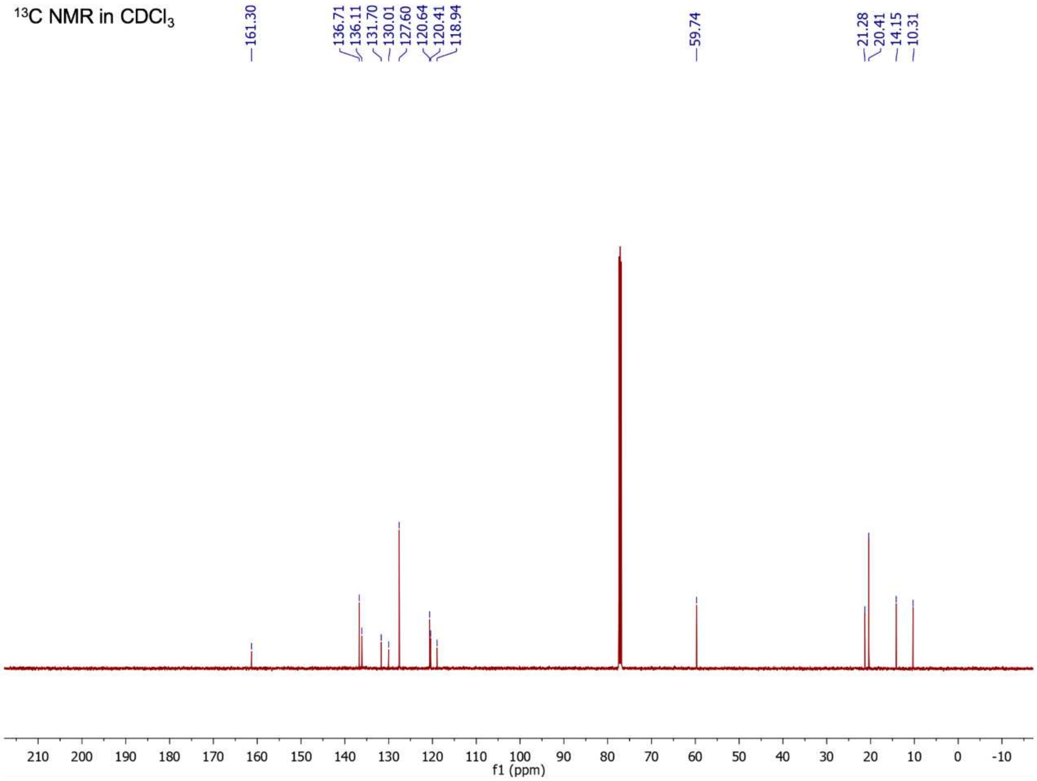

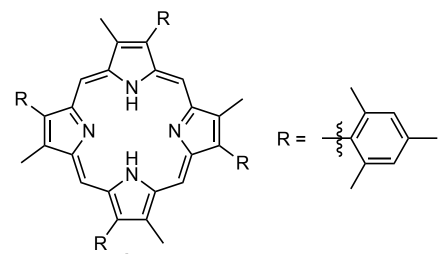

L5

^{1}H NMR in $CDCl_3$

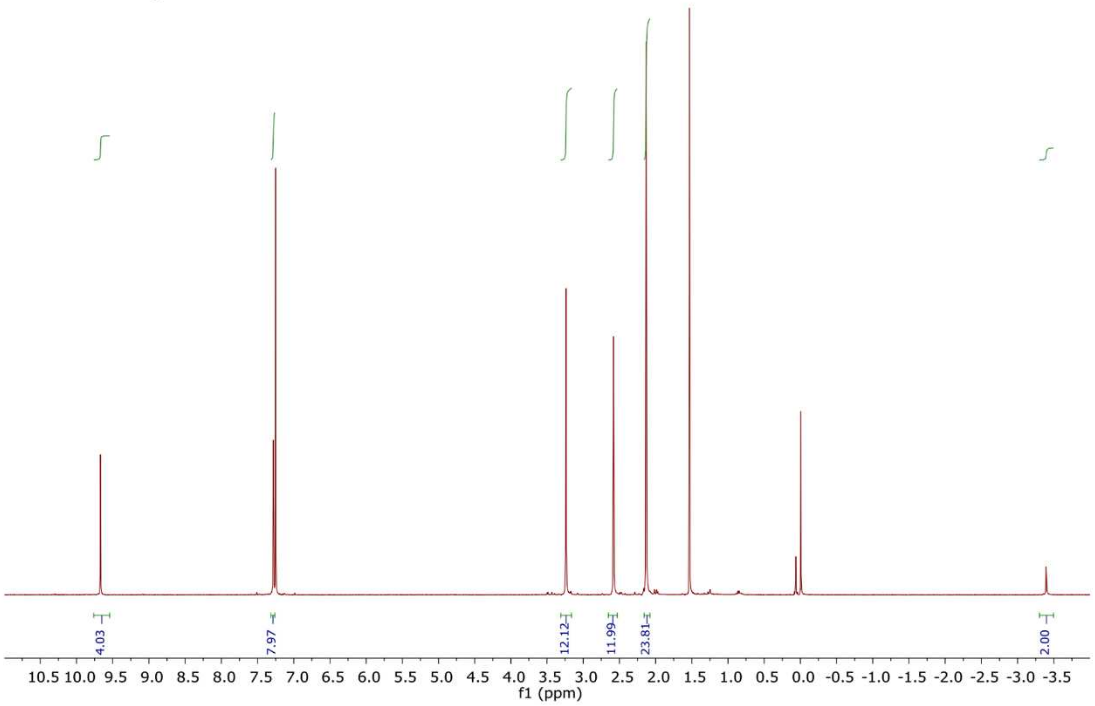

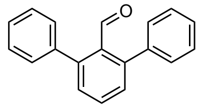

^1H NMR in $CDCl_3$

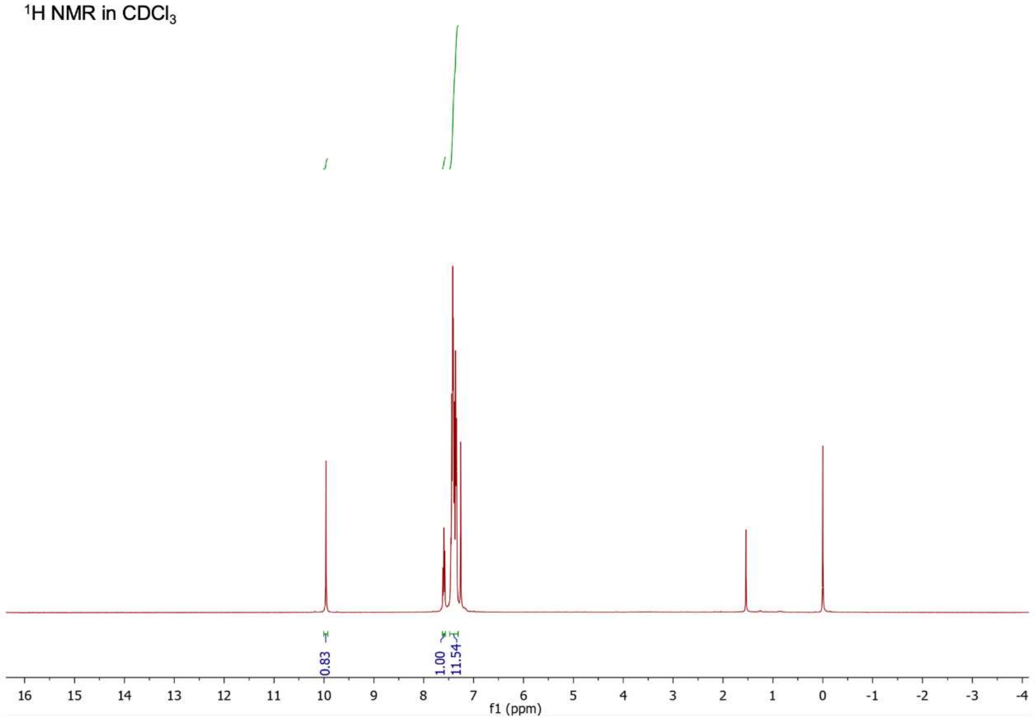

^{13}C NMR in $CDCl_3$

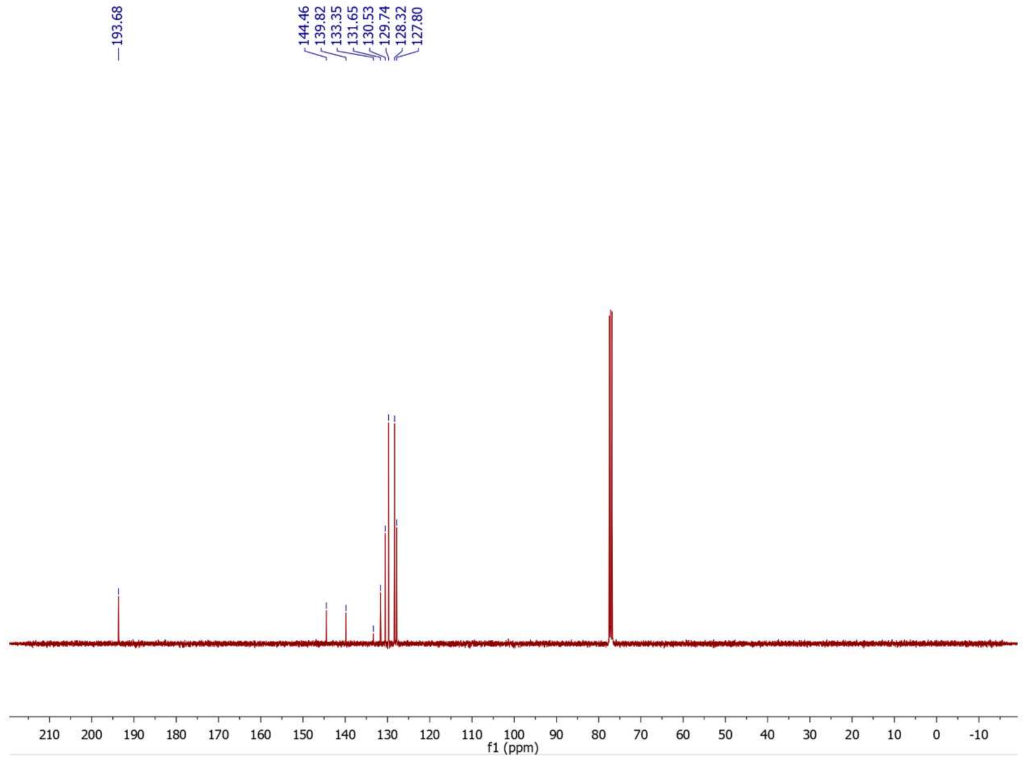

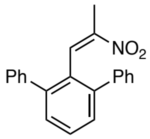

^{1}H NMR in $CDCl_3$

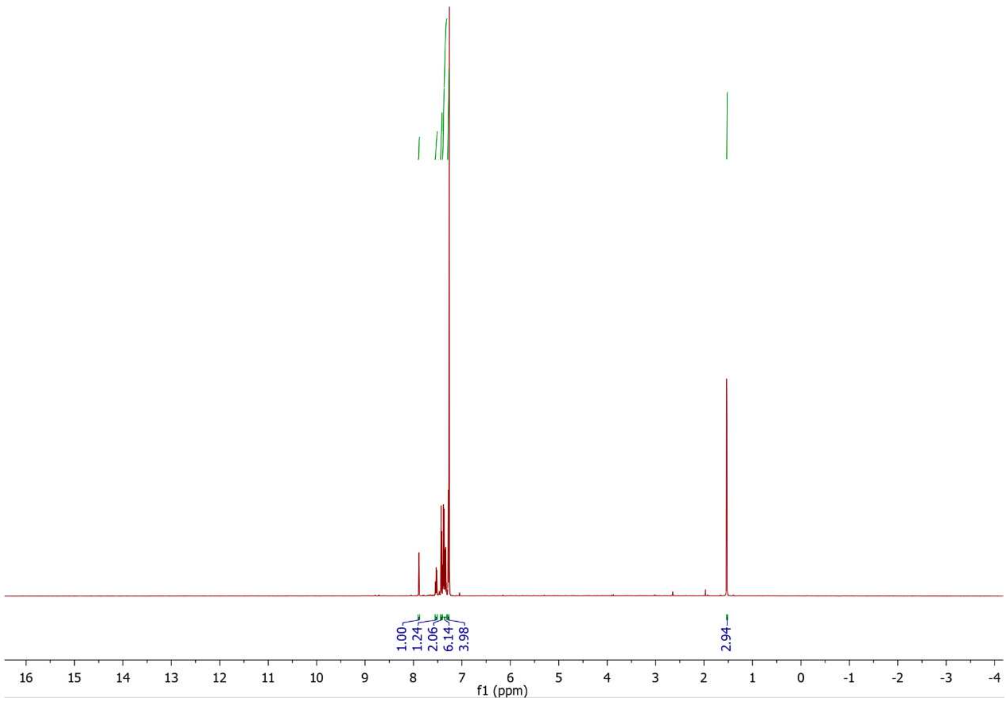

^{13}C NMR in $CDCl_3$

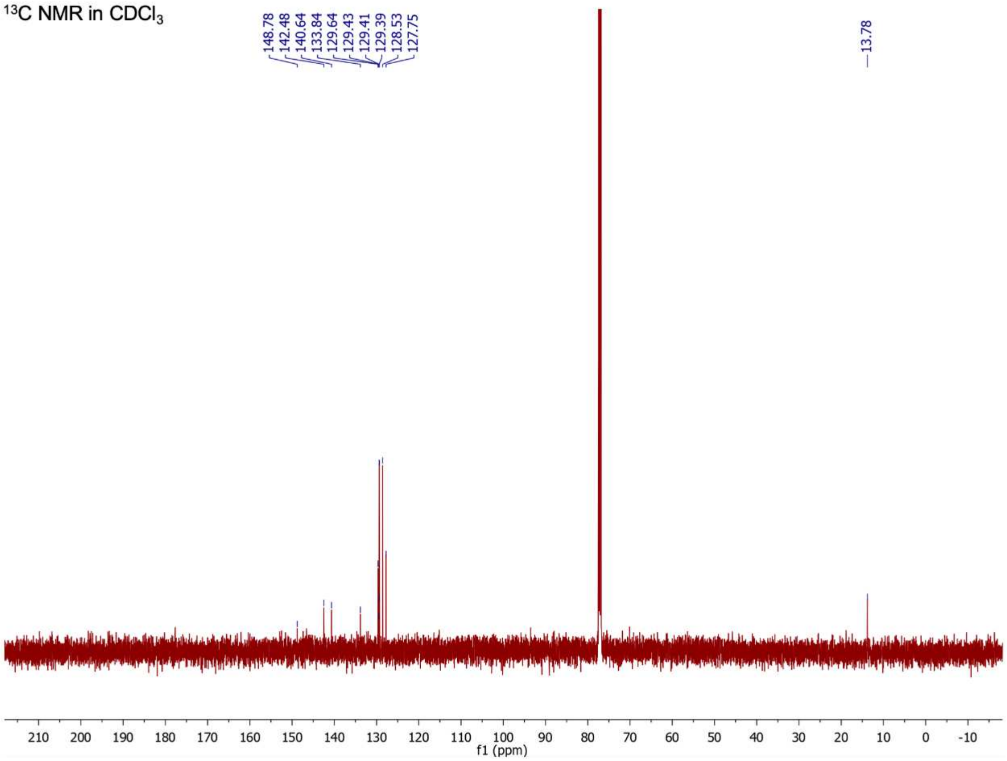

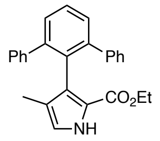

^{1}H NMR in $CDCl_3$

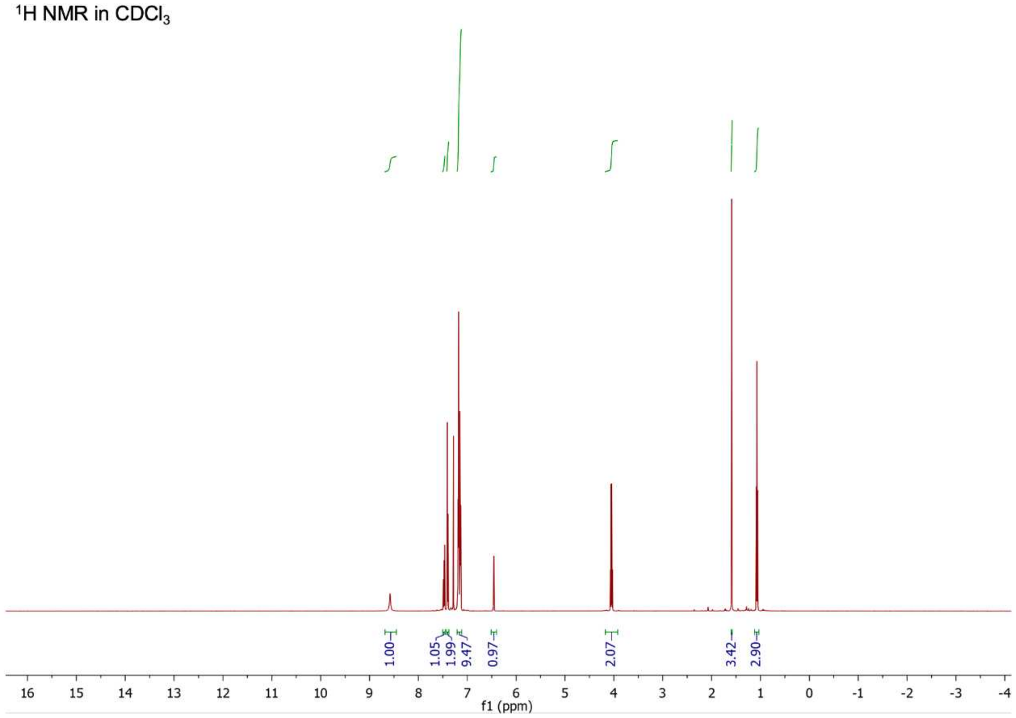

^{13}C NMR in $CDCl_3$

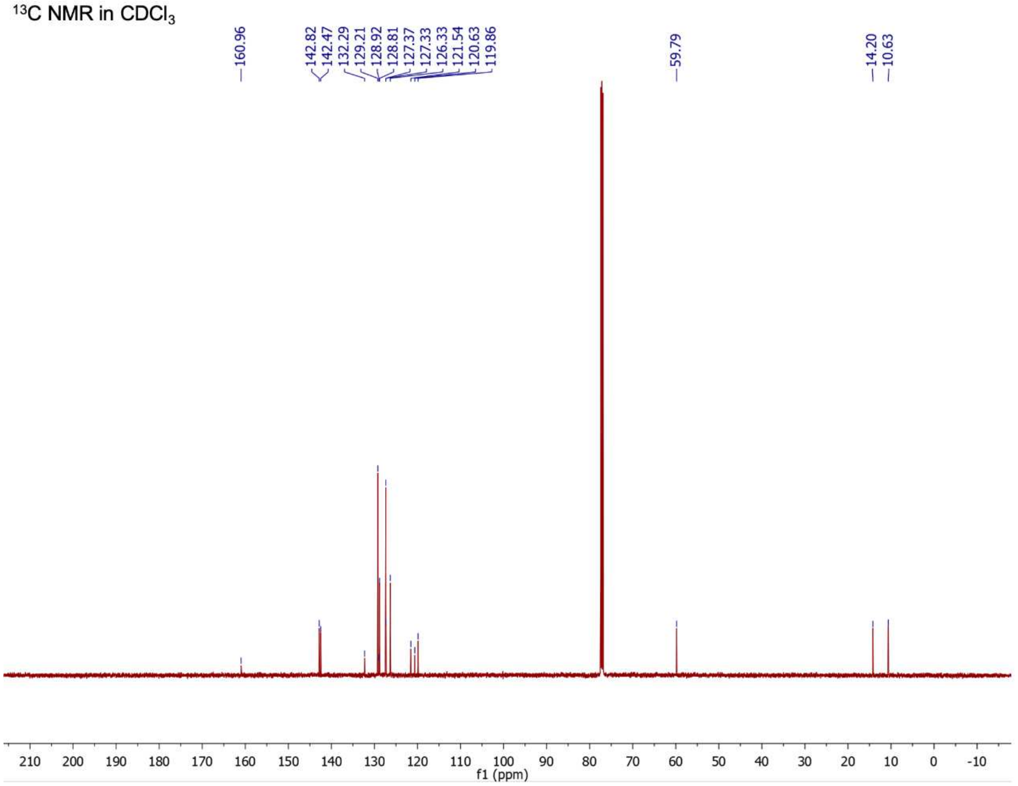

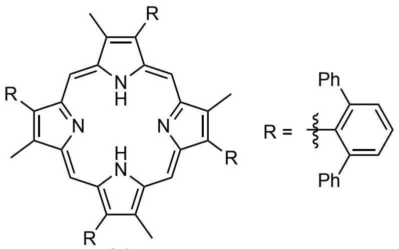

L6

^{1}H NMR in $CDCl_3$

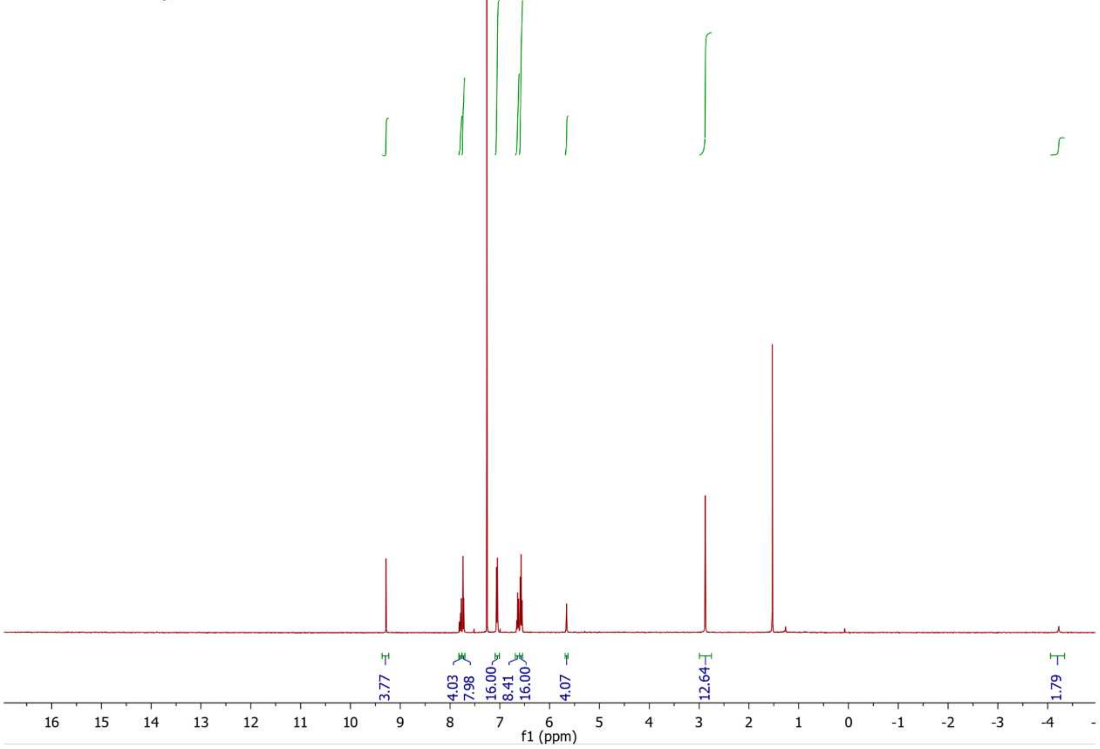

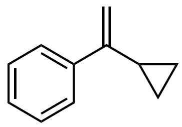

^1H NMR in $CDCl_3$

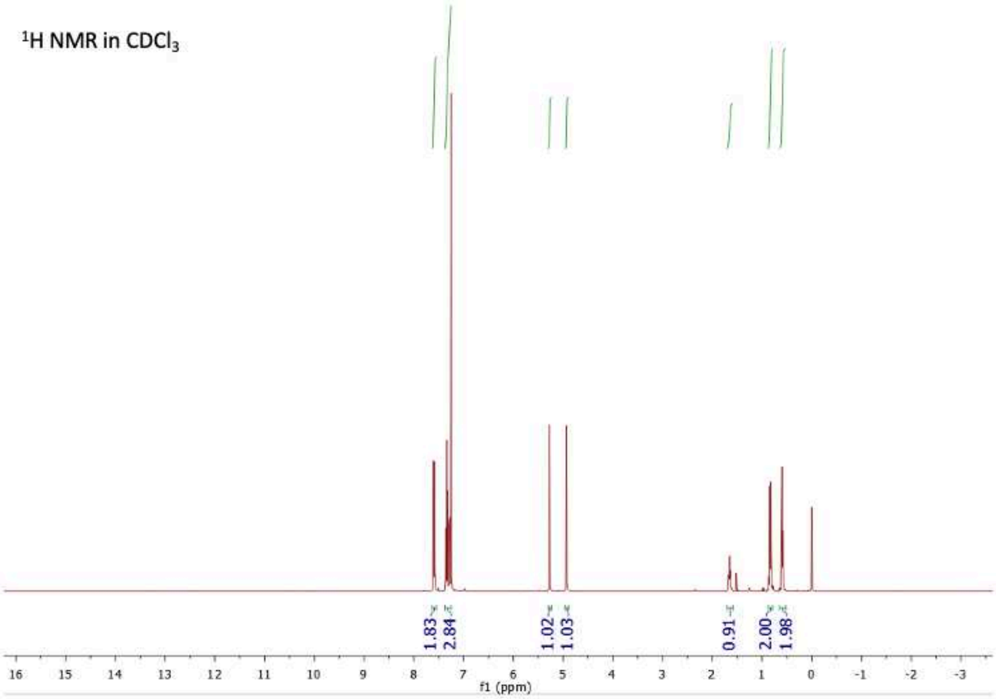

^{13}C NMR in $CDCl_3$

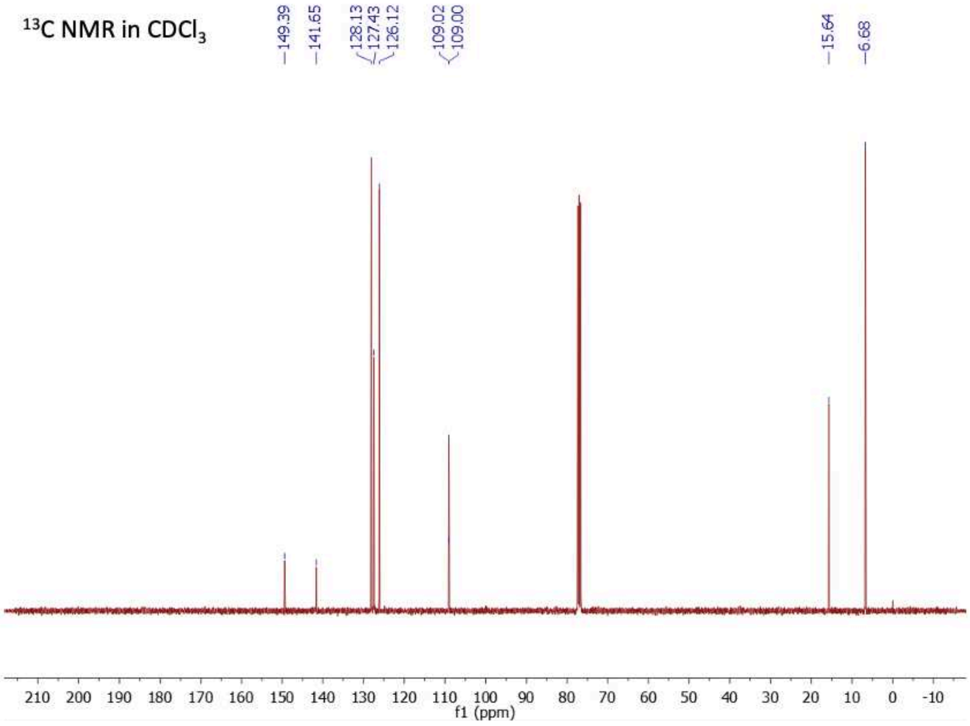

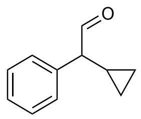

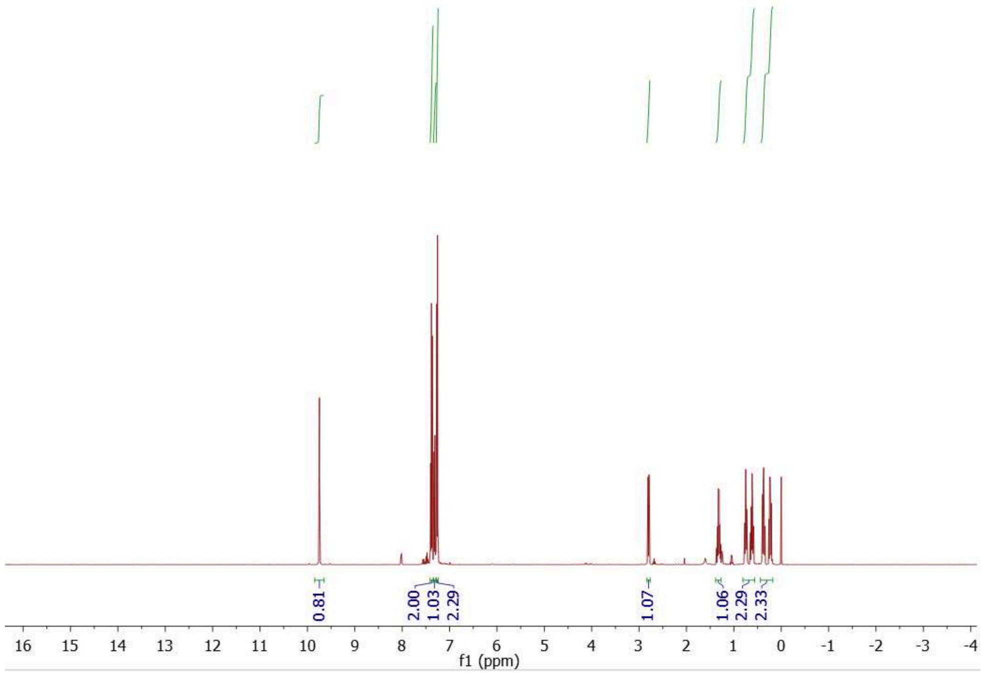
0.81
2.00
1.03
2.29
1.07
1.06
2.29
2.33
16 15 14 13 12 11 10 9 8 7 6 5 4 3 2 1 0 -1 -2 -3 -4
f1 (ppm)

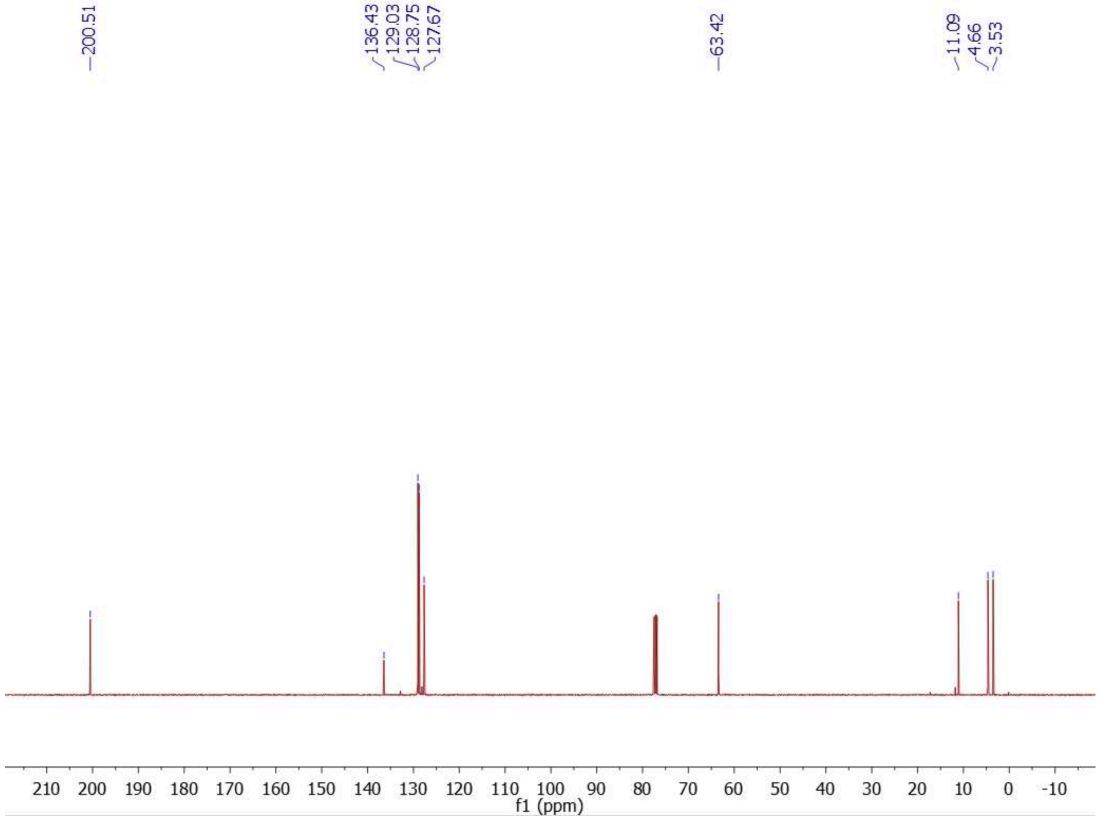
200.51
136.43
129.03
128.75
127.67
63.42
11.09
4.66
3.53
210 200 190 180 170 160 150 140 130 120 110 100 90 80 70 60 50 40 30 20 10 0 -10
f1 (ppm)

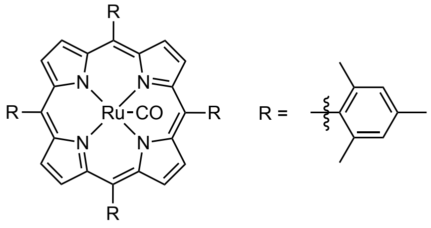
R
N
Ru
CO
R =

[(**L1**)]Ru(CO)]

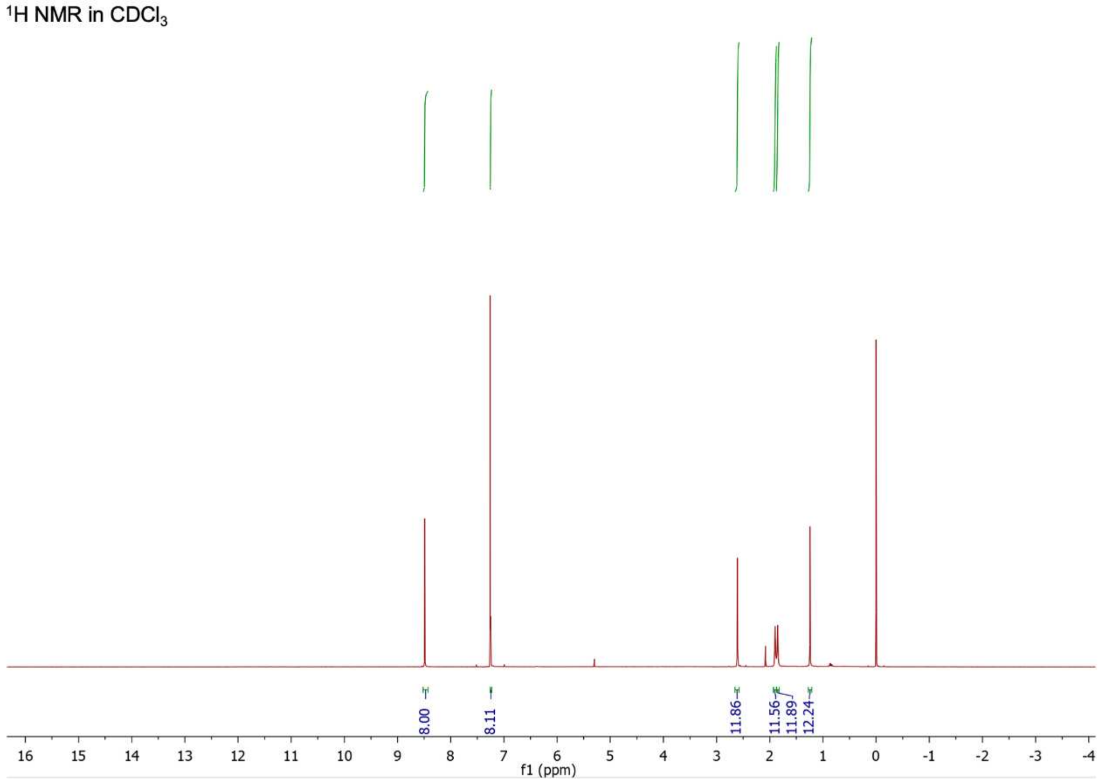
^{1}H NMR in CDCl$_3$
8.00
8.11
11.86
11.56
11.89
12.24
16
15
14
13
12
11
10
9
8
7
6
5
4
3
2
1
0
-1
-2
-3
-4
f1 (ppm)

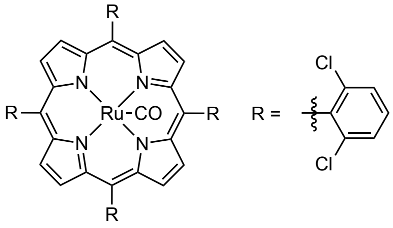

[(**L2**)]Ru(CO)]

^{1}H NMR in $CDCl_3$

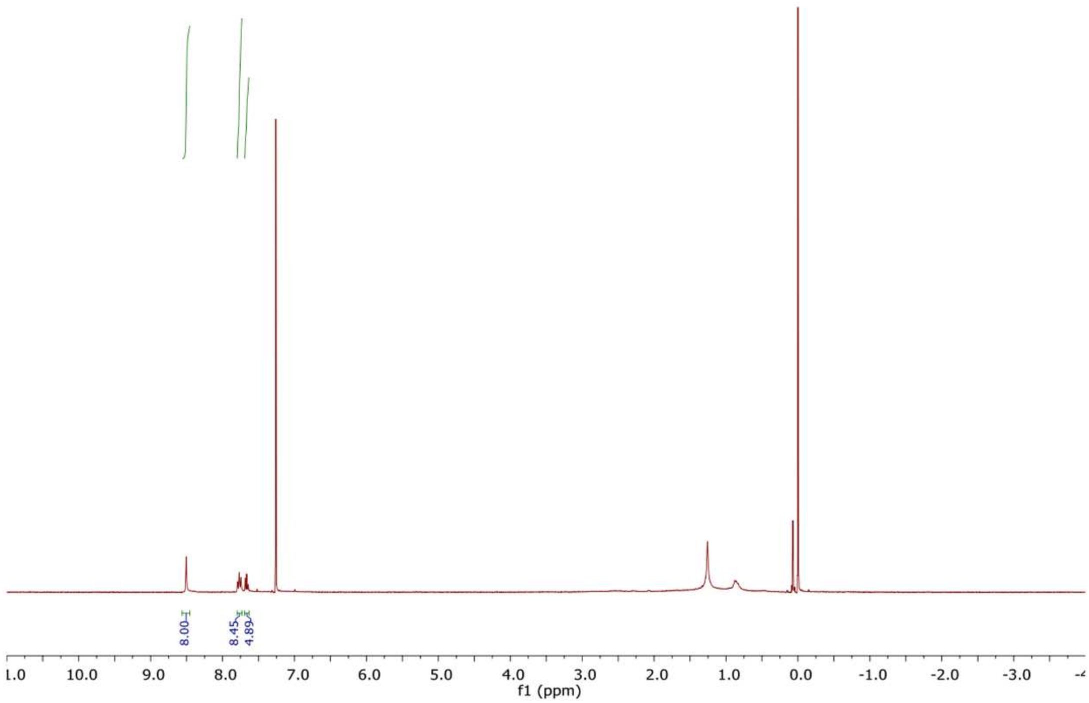

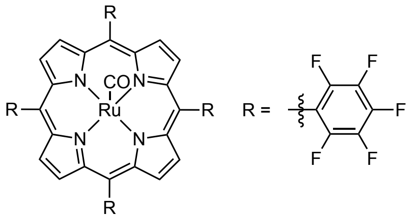

[(**L3**)]Ru(CO)]

^1H NMR in $CDCl_3$

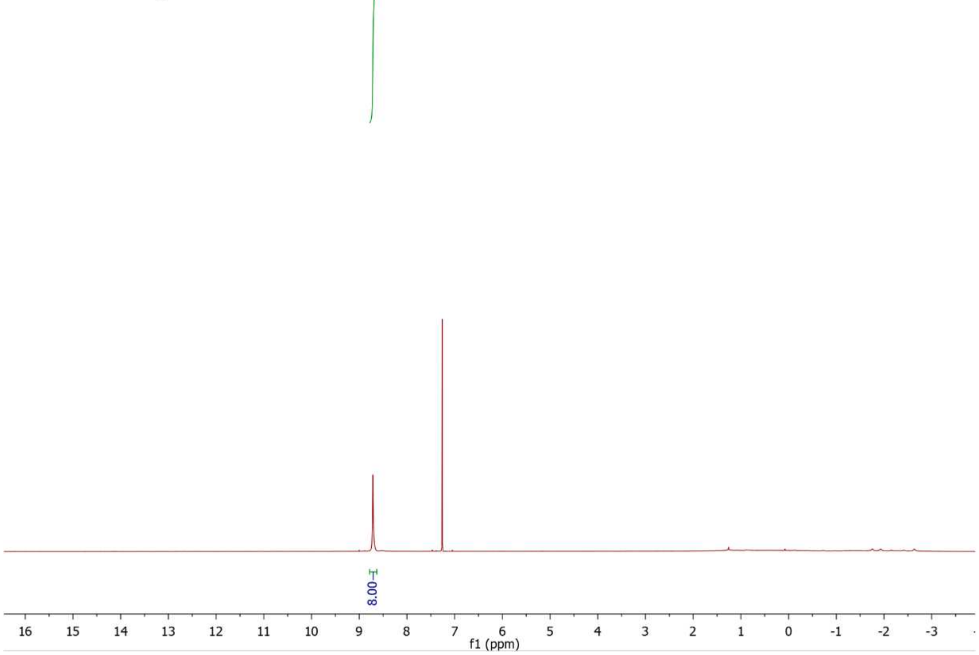

^{19}F NMR in $CDCl_3$

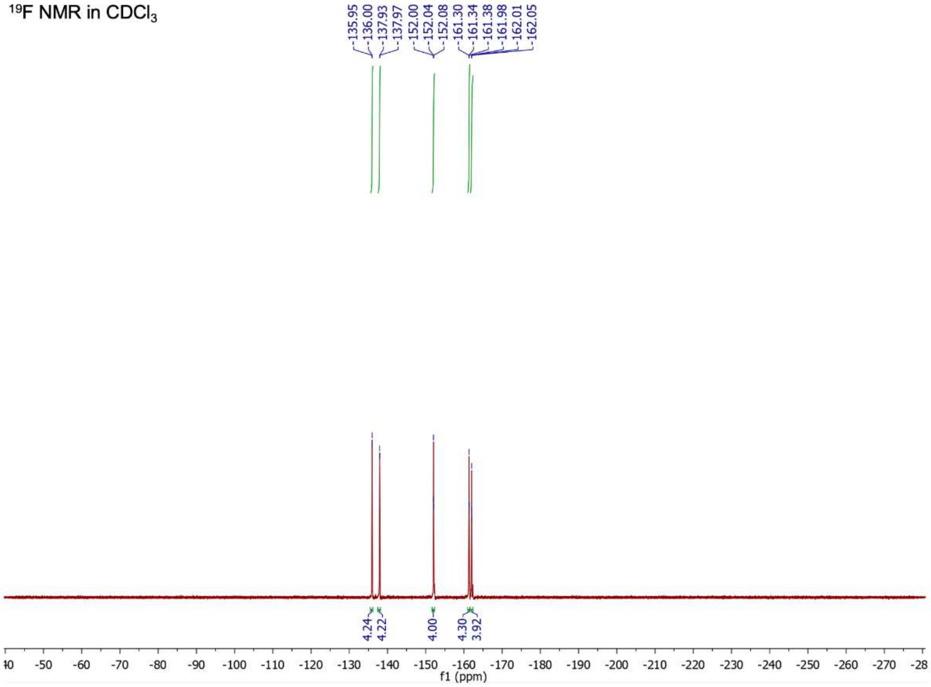

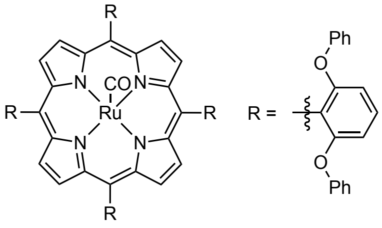
R
N
CO
N
Ru
N
N
R =
O
Ph

[(**L4**)]Ru(CO)]

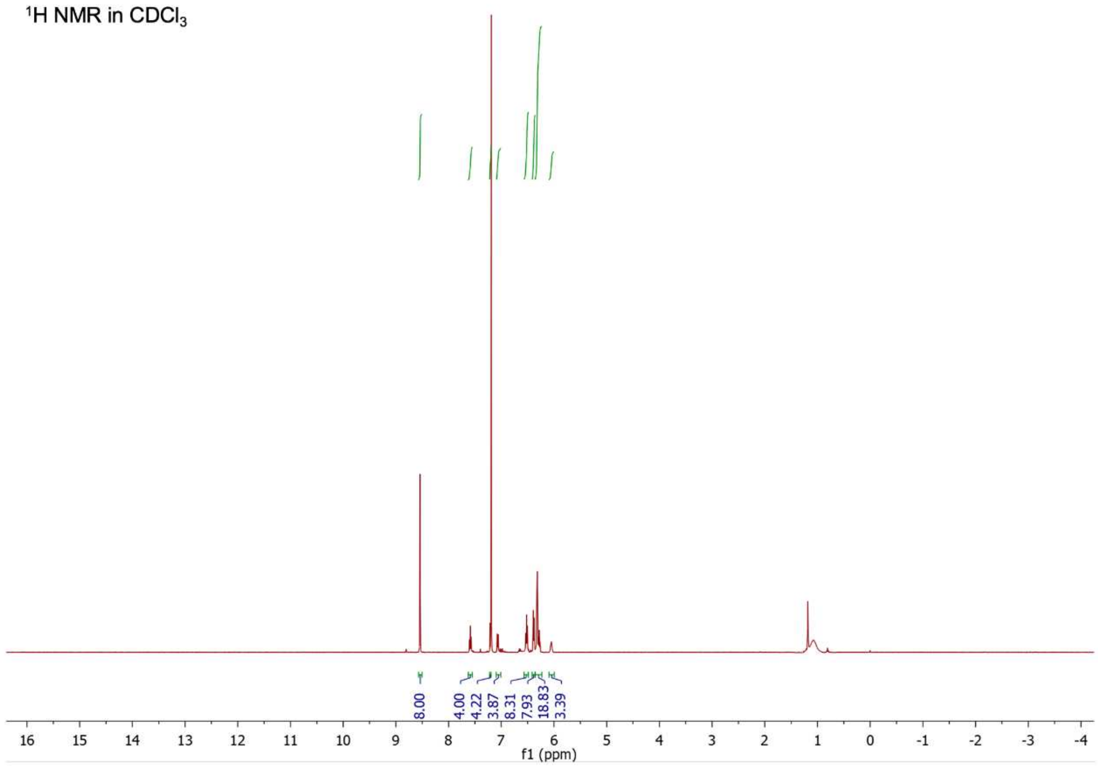
^{1}H NMR in $CDCl_3$
8.00
4.00
4.22
3.87
8.31
7.93
18.83
3.39
16
15
14
13
12
11
10
9
8
7
6
5
4
3
2
1
0
-1
-2
-3
-4
f1 (ppm)

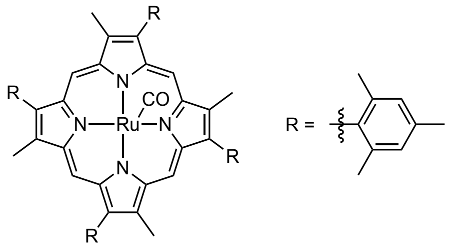

[(**L5**)]Ru(CO)]

^{1}H NMR in $CDCl_3$

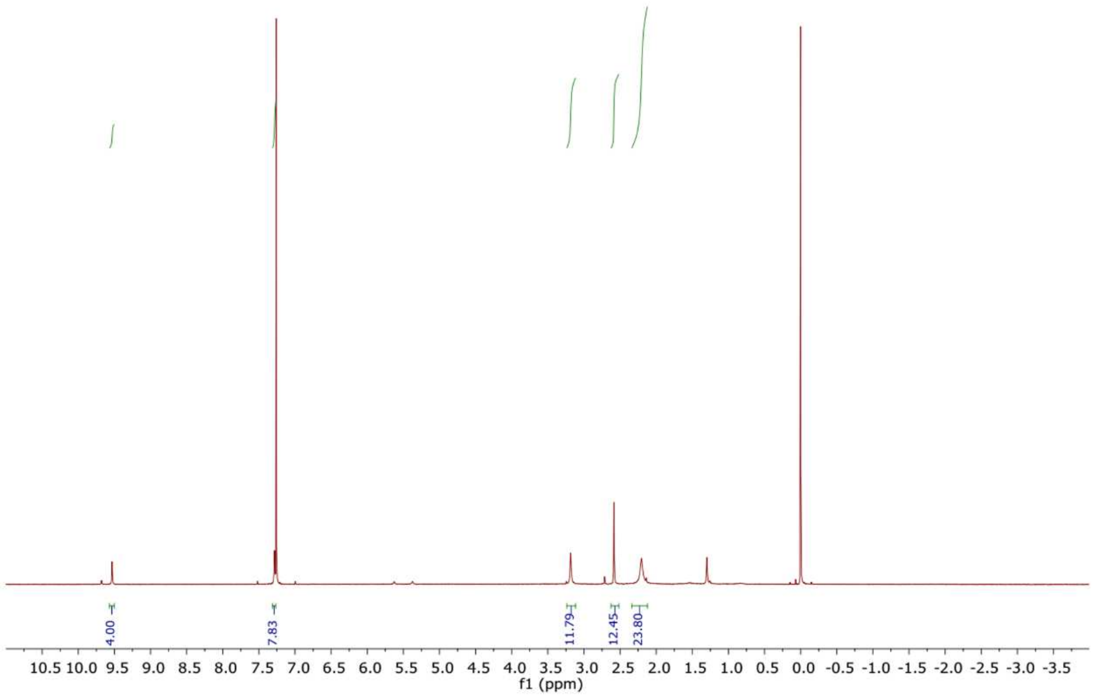

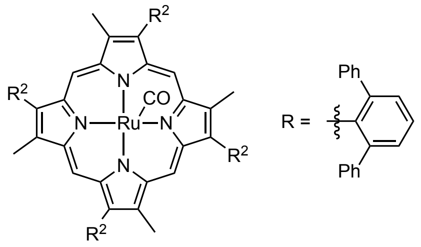
R2
CO
N
Ru
R =
Ph
Ph

[(**L6**)]Ru(CO)]

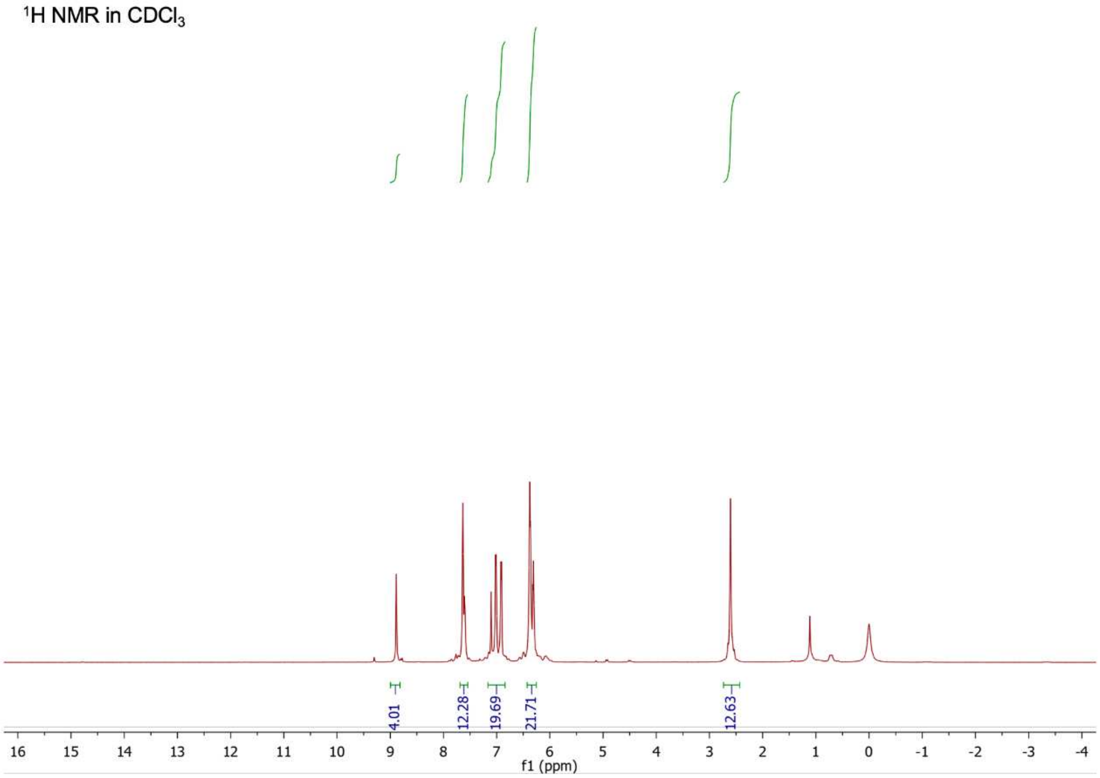
1H NMR in CDCl3
4.01
12.28
19.69
21.71
12.63
16
15
14
13
12
11
10
9
8
7
6
5
4
3
2
1
0
-1
-2
-3
-4
f1 (ppm)

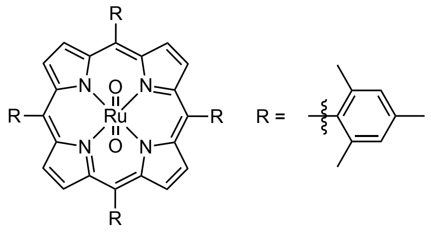

[(**L1**)]Ru(O)$_2$]

^{1}H NMR in $CDCl_3$

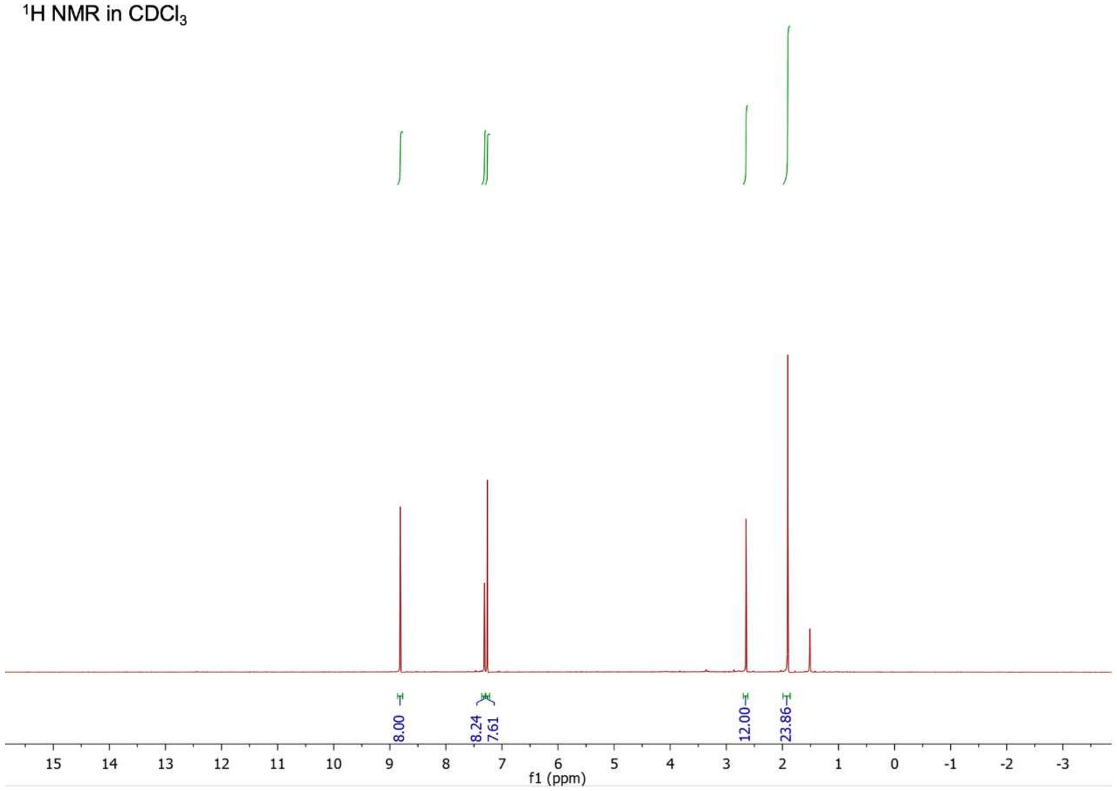

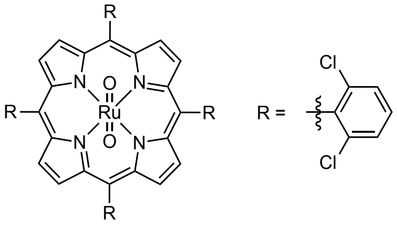
R
N
O
Ru
O
N
R =
Cl
Cl

[(**L2**)]Ru(O)$_2$]

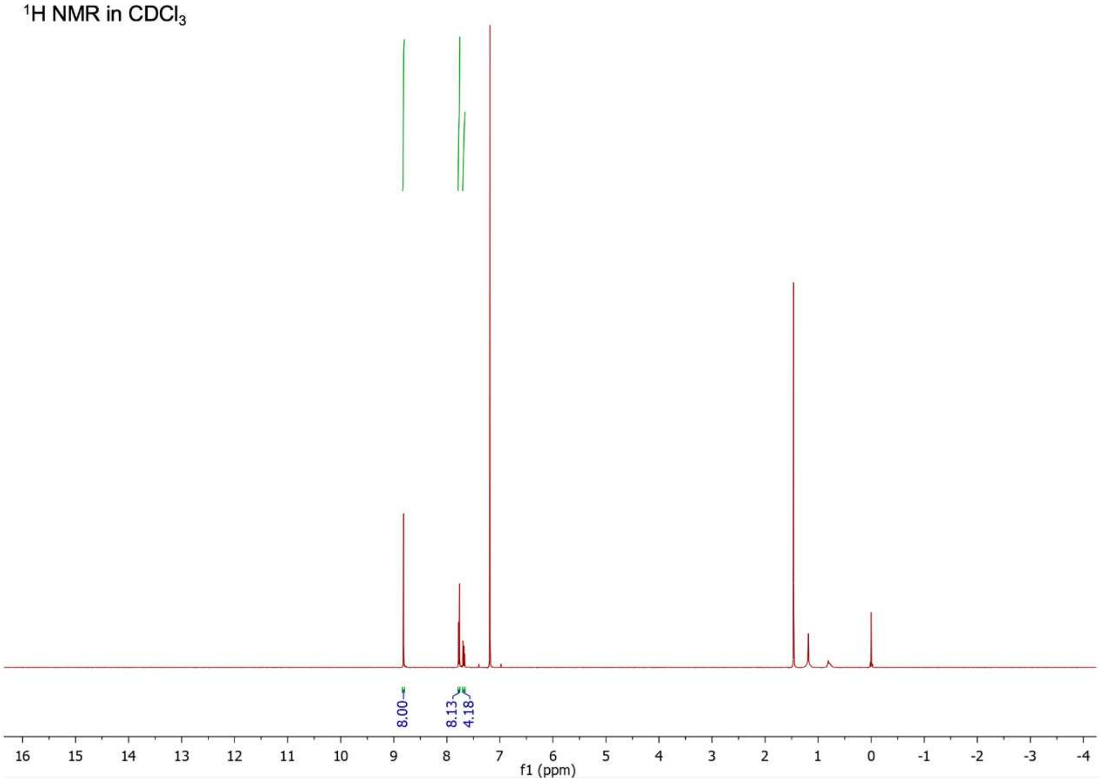
^{1}H NMR in $CDCl_3$
8.00
8.13
4.18
16 15 14 13 12 11 10 9 8 7 6 5 4 3 2 1 0 -1 -2 -3 -4
f1 (ppm)

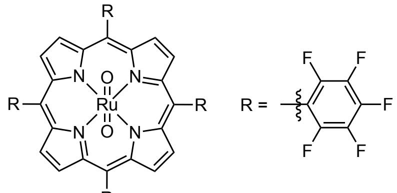

[(**L3**)]Ru(O)$_2$]

^{1}H NMR in $CDCl_3$

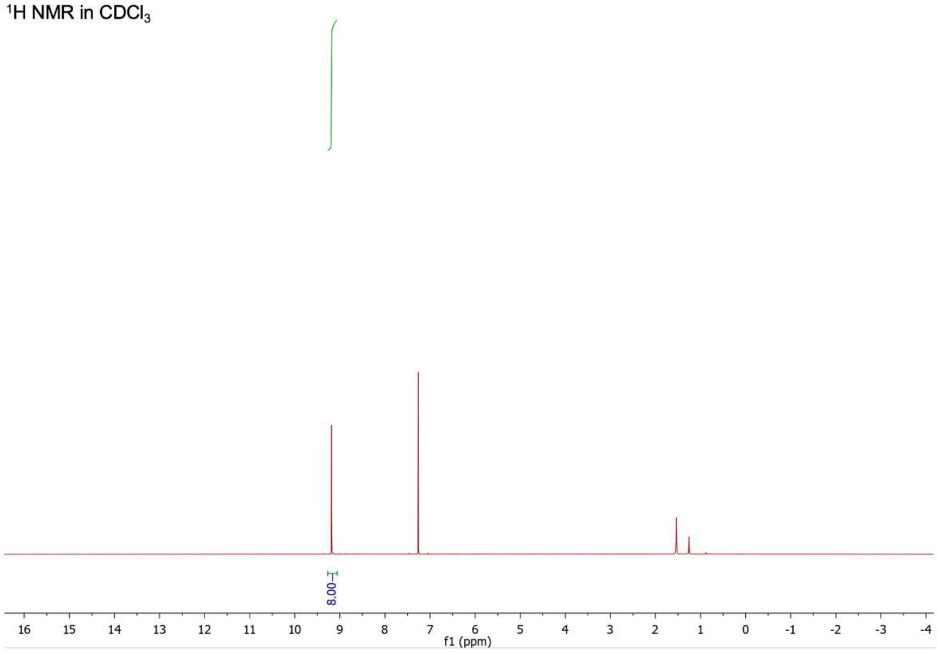

^{19}F NMR in $CDCl_3$

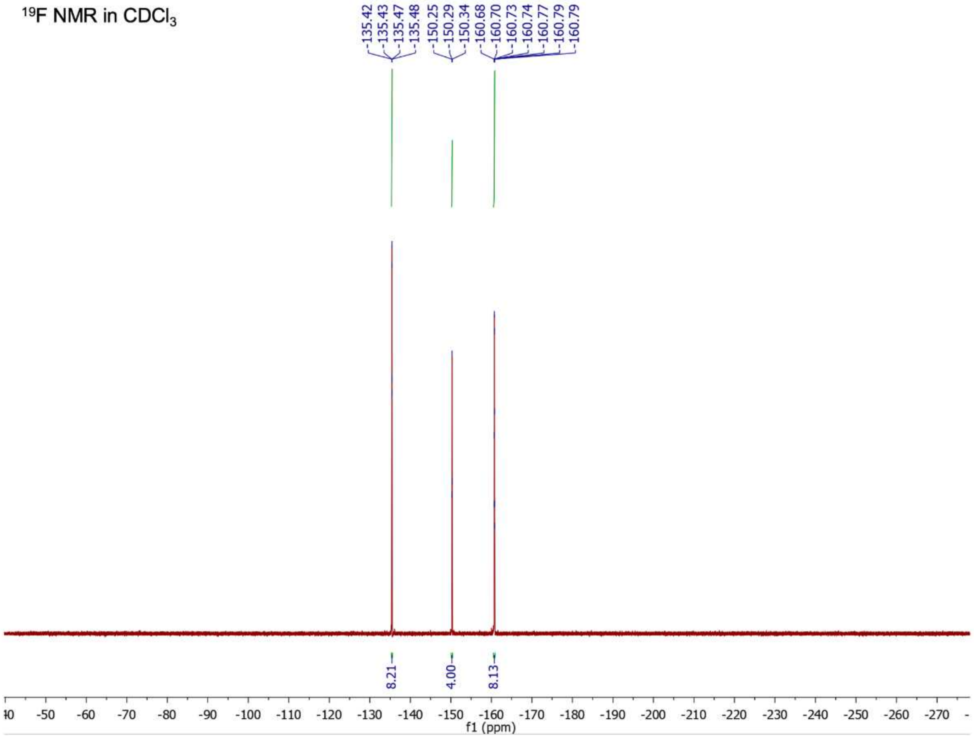

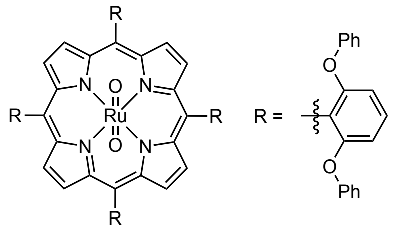
R
N
O
Ru
O
N
R
R
R =
O
Ph
O
Ph

[(**L4**)]Ru(O)$_2$]

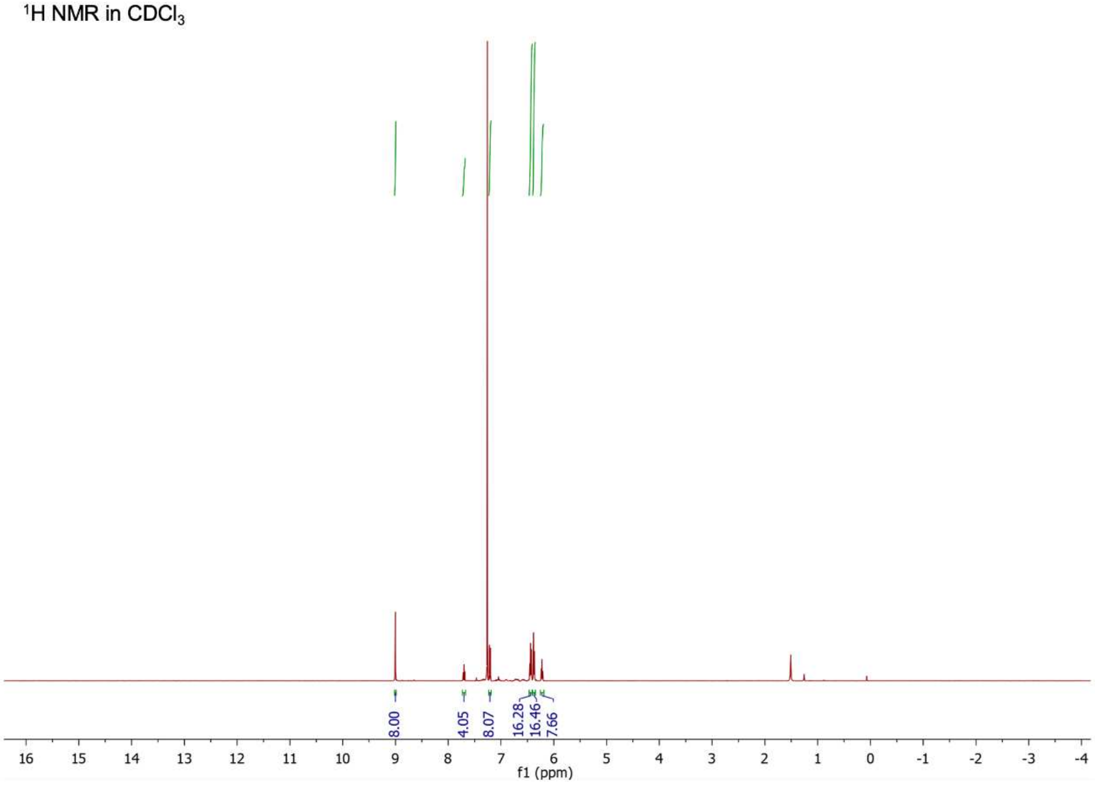
^{1}H NMR in CDCl$_3$
8.00
4.05
8.07
16.28
16.46
7.66
16
15
14
13
12
11
10
9
8
7
6
5
4
3
2
1
0
-1
-2
-3
-4
f1 (ppm)

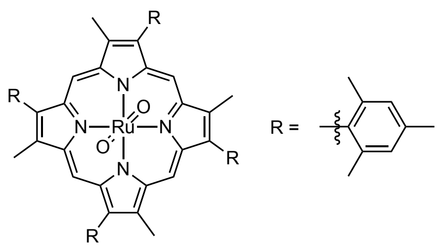

[(**L5**)]Ru(O)$_2$]

^{1}H NMR in $CDCl_3$

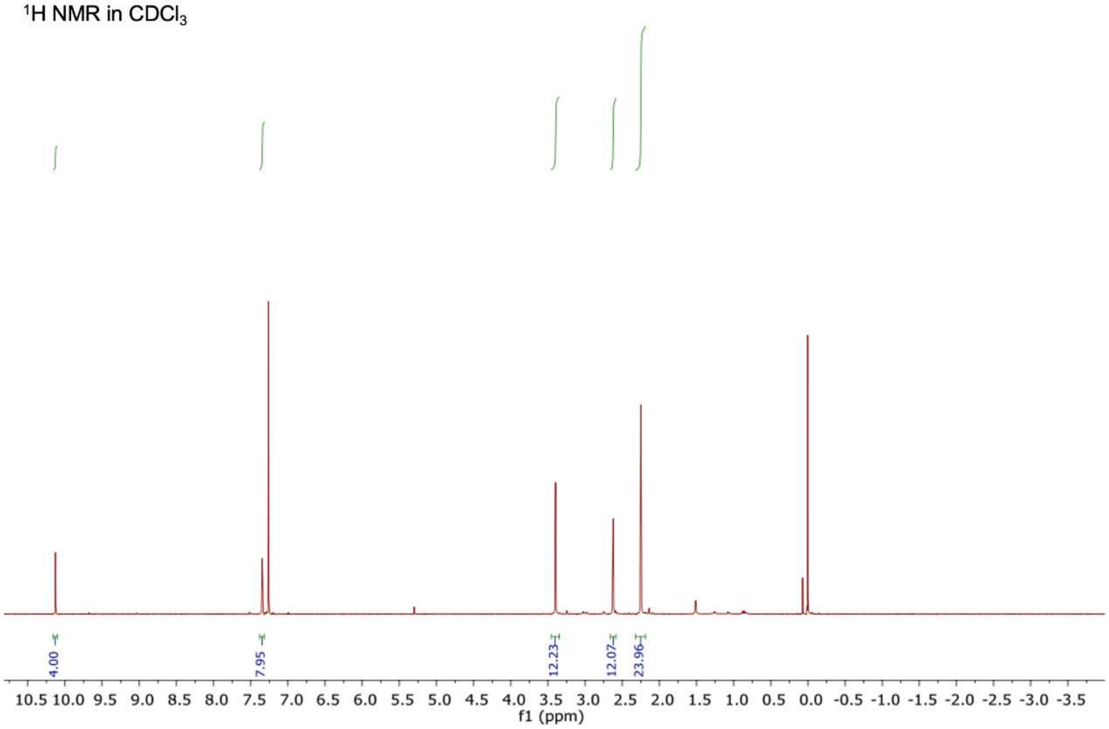

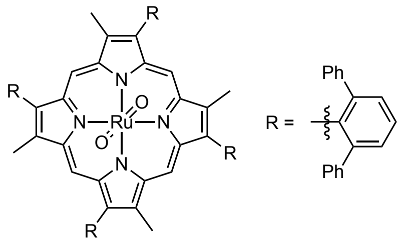

[(**L6**)]Ru(O)$_2$]

^{1}H NMR in $CDCl_3$

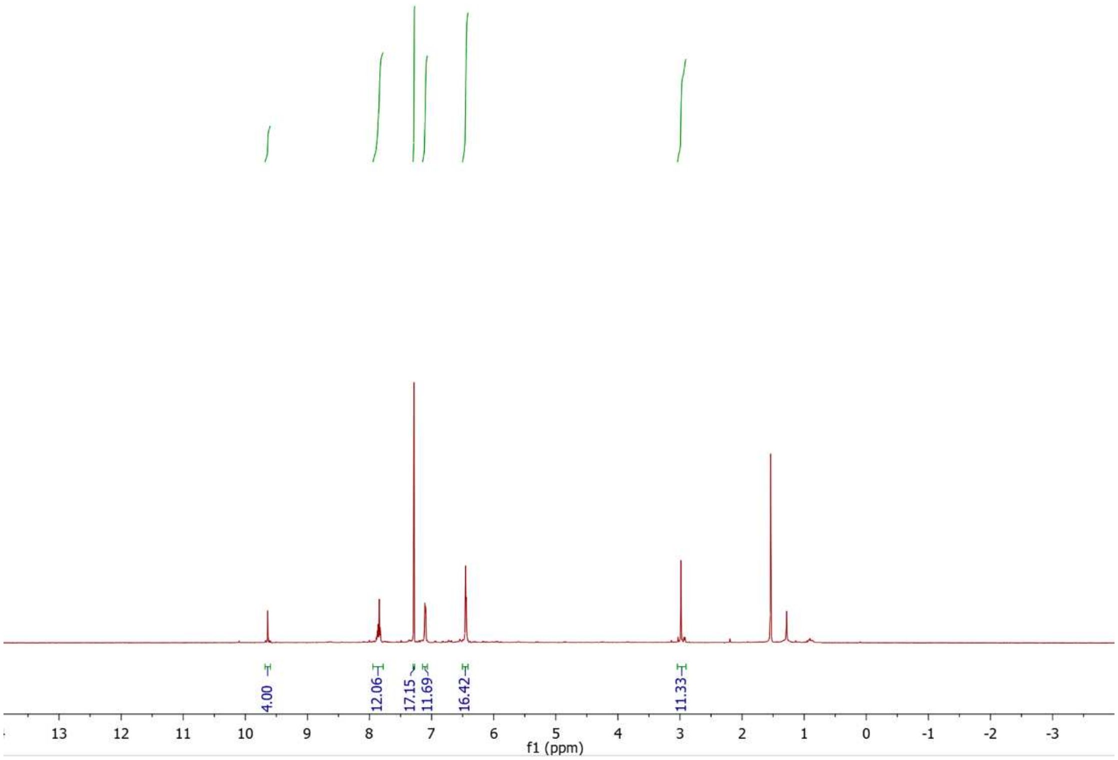

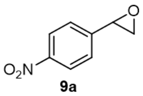
O
O_2N

9a

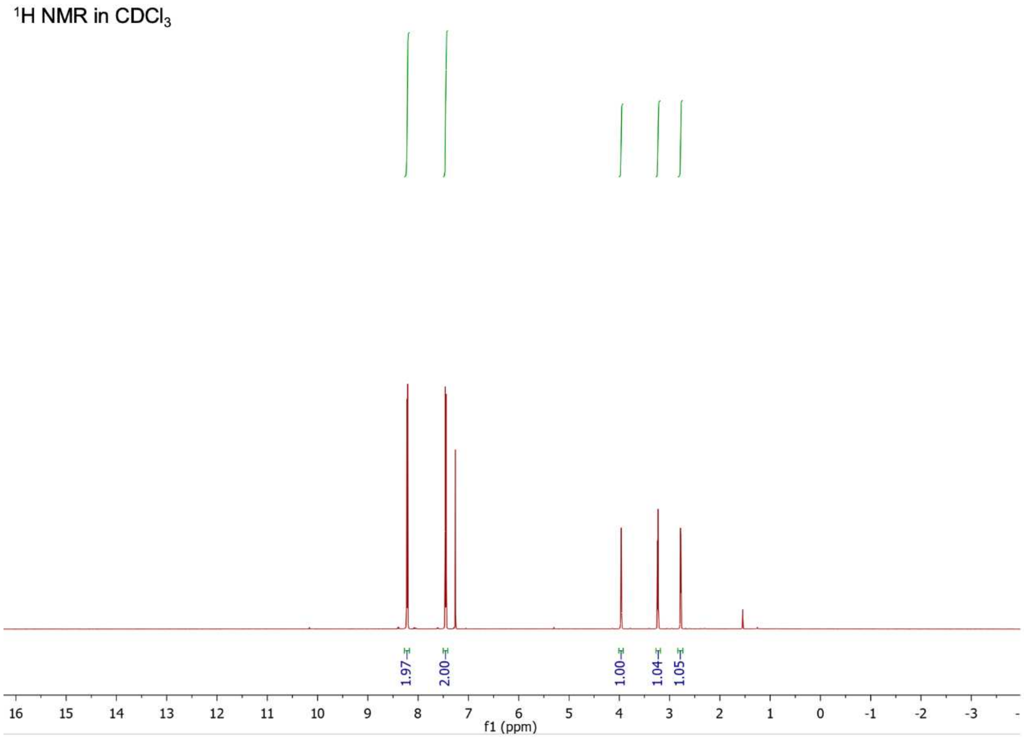
^1H NMR in $CDCl_3$
1.97
2.00
1.00
1.04
1.05
16
15
14
13
12
11
10
9
8
7
6
5
4
3
2
1
0
-1
-2
-3
f1 (ppm)

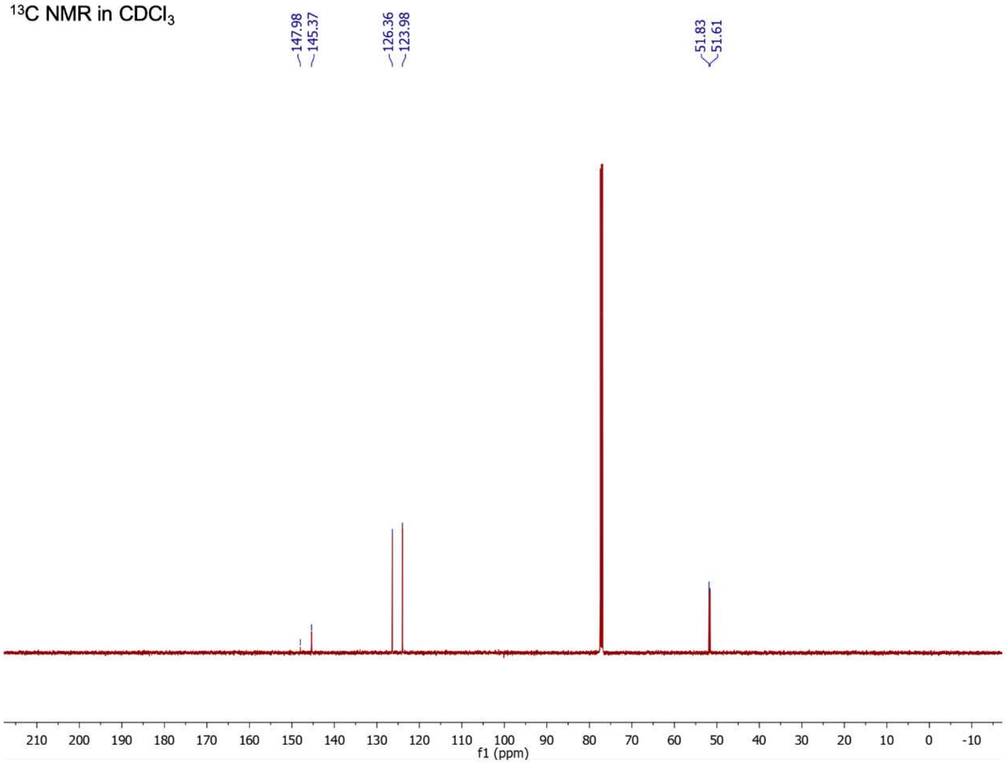
^{13}C NMR in $CDCl_3$
147.98
145.37
126.36
123.98
51.83
51.61
210
200
190
180
170
160
150
140
130
120
110
100
90
80
70
60
50
40
30
20
10
0
-10
f1 (ppm)

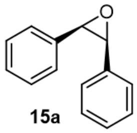

15a

^{1}H NMR in $CDCl_3$

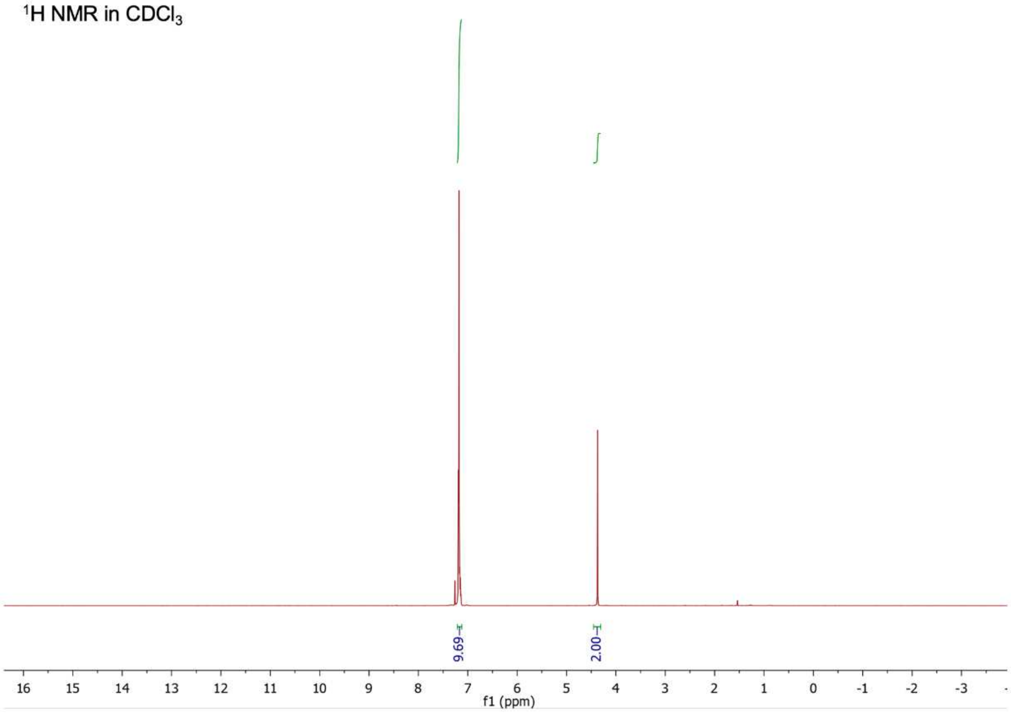

^{13}C NMR in $CDCl_3$

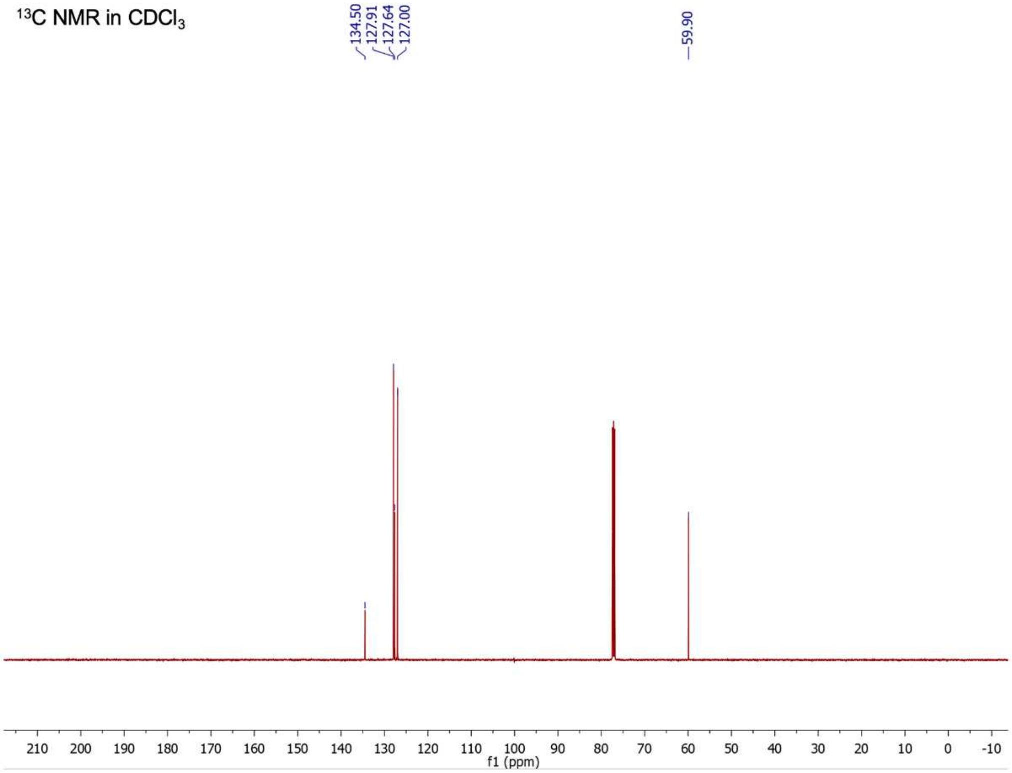

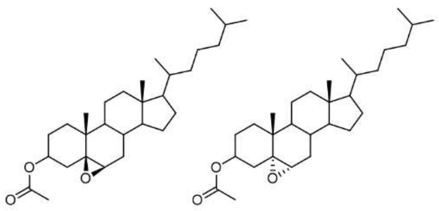

β-epoxide (**18a**)
(major product)

α-epoxide (**18a'**)

^{1}H NMR in $CDCl_3$

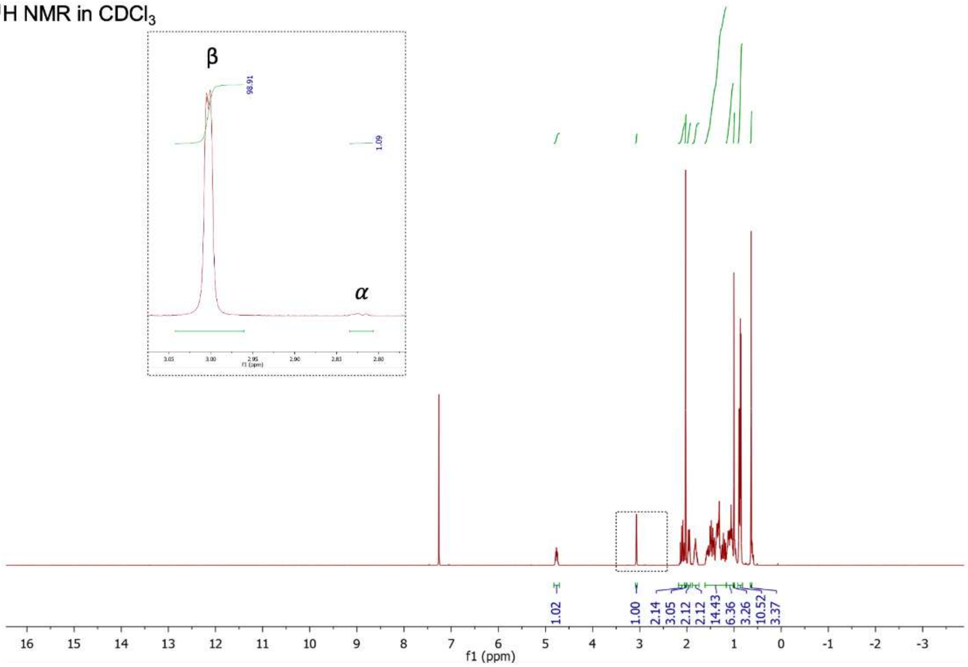

^{13}C NMR in $CDCl_3$

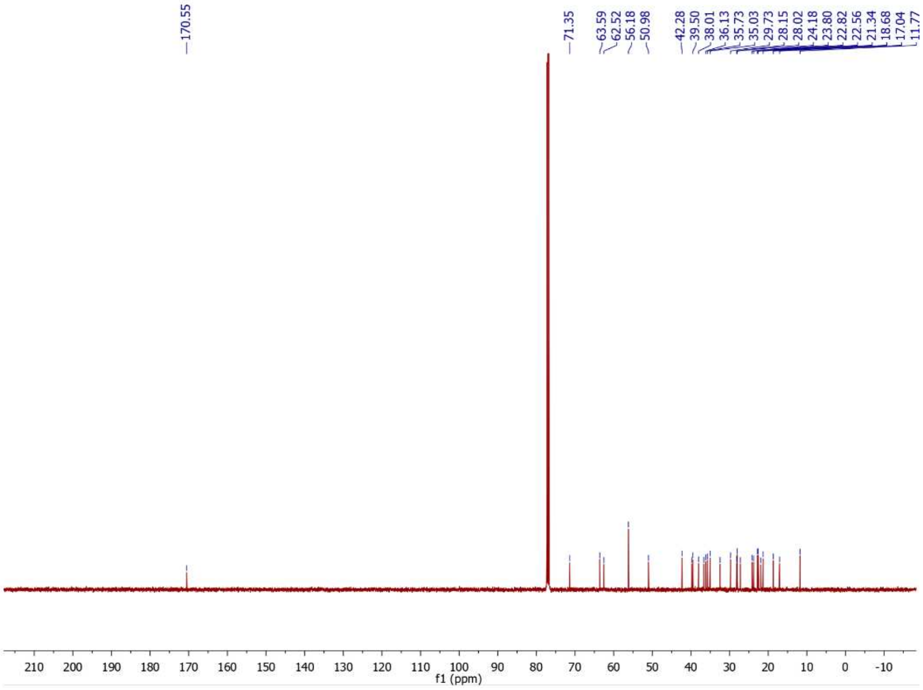

7 Quantum-Chemical Studies

7.1 Computational Methods

Quantum chemical calculations were carried out with the Gaussian 16[23] and ORCA 5[24,25] programs. With Gaussian 16, molecular geometries were optimzed at the density functional theory (DFT) level employing the generalized gradient approximation (GGA) via the M06L[26] functional in conjunction with the D3 atom-pairwise dispersion correction without damping[27] and the implicit continuum solvent model SMD[28] utilizing dichloromethane as the solvent. The triple-zeta valence polarized def2-TZVP[29] basis set was used together with the corresponding auxiliary Coulomb-fitting basis set of Weigend.[30] At this GGA DFT level, abbreviated as M06L-D3(SMD)/def2-TZVP, frequency calculations were performed on the optimized stationary points to characterize minima and transition structures, and to extract thermal contributions to enthalpies and Gibbs energies at 298.15 K and 1 atm under ideal gas conditions. Minimum energy crossing points (MECP) were obtained employing the algorithm developed by Harvey et al.[31] via the *EasyMECP*[32] python routine. Broken-symmetry (BS) unrestricted Kohn–Sham (UKS) geometry optimizations were performed to obtain a molecular structure for relevant species. Singlet energies for these species were obtained after spin-projection according to Yamaguchi:[33–35]

$$E_\mathrm{S} = E_\mathrm{T} - 2\,\frac{E_\mathrm{T} - E_\mathrm{BS}}{\langle S^2\rangle_\mathrm{T} - \langle S^2\rangle_\mathrm{BS}}$$

To ascertain relative energies for selected species, correlated *ab initio* calculations were performed with ORCA 5 utilizing the domain-based local pair natural orbital approximation for coupled-cluster calculations including single and double excitations and semicanonical (T0) as well as full iterative (T1) perturbative triple excitations, DLPNO-CCSD(T).[36,37] The one-particle space was described with the triple-zeta def2-TZVPP and quadruple-zeta def2-QZVPP basis sets[29] on all atoms. The corresponding auxiliary Coulomb[30] and correlation[38] fitting basis sets were used throughout together with the RIJCOSX[39] algorithm. The reference and correlation energies were extrapolated to the complete basis set (CBS) limit, CBS(T,Q),[40] according to

$$E_\mathrm{ref}(\mathrm{CBS}) = E_\mathrm{ref}(Q) + \frac{E_\mathrm{ref}(Q) - E_\mathrm{ref}(T)}{\exp(\alpha\,(\sqrt{4} - \sqrt{3})) - 1}$$

$$E_\mathrm{corr}(\mathrm{CBS}) = \frac{3^\beta\,E_\mathrm{corr}(T) - 4^\beta\,E_\mathrm{corr}(Q)}{3^\beta - 4^\beta}$$

with cardinal numbers 3 for T and 4 for Q, and α = 7.88, β = 2.97.

The `TightPNO` settings in ORCA convention were employed with pair natural orbital (PNO) thresholds set to `TCutPNO` = 10^{-6} and 10^{-7}, in order to arrive at an extrapolated PNO limit, PNO(6,7),[41] for the correlation energies according to

$$E_\mathrm{corr}(\mathrm{PNO(6,7)}) = E_\mathrm{corr}(\mathrm{PNO6}) + F_\mathrm{PNO} \times (E_\mathrm{corr}(\mathrm{PNO7}) - E_\mathrm{corr}(\mathrm{PNO6}))$$

where F_PNO = 1.5 for a set of `TCutPNO` thresholds differing by one order of magnitude.

7.2 Formation of transient hydroxo-hydroperoxo intermediate [(L1)Ru(OH)(OOH)]

DFT calculations using M06L-D3(SMD)/def2-TZVP predict the reaction of [(**L1**)Ru(O)$_2$], **A**, with water towards the transient hydroxo-hydroperoxo intermediate [(**L1**)Ru(OH)(OOH)], **B**, to be energetically uphill by $\Delta_r H^{298}$ = +20 kcal mol^{-1} and highly endergonic ($\Delta_r G^{298}$ = +35 kcal mol^{-1}). In an effort to further scrutinize the energetics of transient hydroxo-hydroperoxo intermediate [(**L1**)Ru(OH)(OOH)], the above reaction was computed for the smaller 5,10,15,20-unsubstitued porphyrinato (**L** = **Por**) congeners, [(**Por**)Ru(O)$_2$] and [(**Por**)Ru(OH)(OOH)], which allows inspection of the species at higher levels of *ab initio* theory. Table S6 illustrates that (i) DFT predicts comparable energetics for both, substituted **L** = **L1** as well as unsubstituted **L** = **Por**, and (ii) an increasing level of correlation energy, CCSD → CCSD(T0) → CCSD(T1), decreases the stability of the hydroxo-hydroperoxo intermediate significantly further; note that DLPNO extrapolation to the pair-natural orbital (PNO) threshold limit, as well as basis set extrapolation to the complete basis set (CBS) limit, will be a slight counterpoise to this correlation effect.

Table S6: Comparison of the effect of 5,10,15,20-porphyrinate substituents, R, on the reaction energy of the ruthenium dioxo complex with water to the hydroxo-hydroperoxo intermediate, [(**L**)Ru(O)$_2$] + H_2O ⟶ [(**L**)Ru(OH)(OOH)], computed for **L** = TMP, Por, i.e., R = Mes, H. The influence of correlation effects via coupled cluster theory is shown for unsubstituted systems with **L** = Por. Relative energies E_{rel}, enthalpies H_{rel} and Gibbs energies G_{rel} given in kcal mol^{-1}, with molecular geometries, thermal contributions to enthalpies (H_{tot}) and Gibbs energies (G_{tot}) taken from the corresponding M06L-D3(SMD,CH_2Cl_2)/def2-TZVP calculations.

Ligand[a]	Level			Relative Energy		
	theory	basis set	thresh	E_{rel}	H_{rel}	G_{rel}
**L1**	M06L-D3(SMD)	def2-TZVP		18.2	19.9	34.7
**Por**	M06L-D3(SMD)	def2-TZVP		19.4	20.9	31.7
**Por**	DLPNO-CCSD	def2-TZVPP	PNO6	8.7	10.2	20.8
**Por**	DLPNO-CCSD(T0)	def2-TZVPP	PNO6	29.9	31.4	42.2
**Por**	DLPNO-CCSD(T1)	def2-TZVPP	PNO6	34.7	36.2	47.0
**Por**	DLPNO-CCSD	def2-TZVPP	PNO7	6.3	7.8	18.6
**Por**	DLPNO-CCSD(T0)	def2-TZVPP	PNO7	26.5	28.0	38.8
**Por**	DLPNO-CCSD(T1)	def2-TZVPP	PNO7	31.0	32.5	43.3
**Por**	DLPNO-CCSD	def2-TZVPP	PNO(6,7)	5.2	6.6	17.4
**Por**	DLPNO-CCSD(T0)	def2-TZVPP	PNO(6,7)	24.8	26.3	37.1
**Por**	DLPNO-CCSD(T1)	def2-TZVPP	PNO(6,7)	29.2	30.6	41.5
**Por**	DLPNO-CCSD	def2-QZVPP	PNO6	8.7	10.2	21.0
**Por**	DLPNO-CCSD(T0)	def2-QZVPP	PNO6	30.3	31.7	42.6
**Por**	DLPNO-CCSD(T1)	def2-QZVPP	PNO6	34.9	36.3	47.2
**Por**	DLPNO-CCSD	def2-QZVPP	PNO7	6.3	7.8	18.6
**Por**	DLPNO-CCSD(T0)	def2-QZVPP	PNO7	26.9	28.3	39.2
**Por**	DLPNO-CCSD(T1)	def2-QZVPP	PNO7	31.2	32.7	43.5
**Por**	DLPNO-CCSD	def2-QZVPP	PNO(6,7)	5.1	6.6	17.4
**Por**	DLPNO-CCSD(T0)	def2-QZVPP	PNO(6,7)	25.2	26.6	37.5
**Por**	DLPNO-CCSD(T1)	def2-QZVPP	PNO(6,7)	29.4	30.8	41.7
**Por**	DLPNO-CCSD	CBS(T,Q)	PNO(6,7)	4.5	6.0	16.8
**Por**	DLPNO-CCSD(T0)	CBS(T,Q)	PNO(6,7)	24.8	26.3	37.1
**Por**	DLPNO-CCSD(T1)	CBS(T,Q)	PNO(6,7)	29.0	30.4	41.3

[a]For substituent R = Mes, ligand **L** = TMP = 5,10,15,20-tetramesitylporphyrinate-dianion ≡ **L1**;
for R = H, ligand **L** = Por = porphyrinate-dianion.

Table S7: Total energies (Hartree) for individual species corresponding to Table S6, including DFT-corrections to enthalpies and to Gibbs energies, correlation energy contributions, as well as CC diagnostics for singles amplitudes. Relative energies (kcal mol^{-1}) are also given for comparison.

**M06L-D3(SMD)/def2-TZVP**						
Species	E_{tot}	H_{tot}^{298}	G_{tot}^{298}	E_{rel}	H_{rel}	G_{rel}
H_2O	−76.450 684	0.024 877	0.003 447			
[(**L1**)Ru(O)$_2$]	−2630.475 908	0.994 559	0.824 803			
[(**L1**)Ru(OH)(OOH)]	−2706.897 520	1.022 049	0.854 423	18.2	19.9	34.7
[(**Por**)Ru(O)$_2$]	−1234.133 374	0.301 649	0.234 678			
[(**Por**)Ru(OH)(OOH)]	−1310.553 096	0.328 831	0.257 705	19.4	20.9	31.7

**DLPNO-CCSD(T)**										
	thresh	E_{tot}(ref)	E_{corr}(CCSD)	E_{corr}(T0)	E_{corr}(T1)	$\mathcal{T}_1$	E_{rel}^{ref}	E_{rel}^{CCSD}	$E_{rel}^{CCSD(T0)}$	$E_{rel}^{CCSD(T1)}$
**def2-TZVPP**										
H_2O	PNO6	−76.062 179	−0.267 555	−0.007 369	−0.007 645	0.008				
[(**Por**)Ru(O)$_2$]	PNO6	−1226.192 340	−5.093 793	−0.303 174	−0.331 333	0.018				
[(**Por**)Ru(OH)(OOH)]	PNO6	−1302.326 594	−5.275 397	−0.276 743	−0.297 556	0.020	−45.2	8.7	29.9	34.7
H_2O	PNO7	−76.062 179	−0.267 533	−0.007 390	−0.007 671	0.008				
[(**Por**)Ru(O)$_2$]	PNO7	−1226.192 340	−5.101 590	−0.316 536	−0.347 251	0.019				
[(**Por**)Ru(OH)(OOH)]	PNO7	−1302.326 594	−5.286 955	−0.291 780	−0.315 603	0.023	−45.2	6.3	26.5	31.0
H_2O	PNO(6,7)	−76.062 179	−0.267 522	−0.007 400	−0.007 684					
[(**Por**)Ru(O)$_2$]	PNO(6,7)	−1226.192 340	−5.105 488	−0.323 217	−0.355 211					
[(**Por**)Ru(OH)(OOH)]	PNO(6,7)	−1302.326 594	−5.292 734	−0.299 298	−0.324 627		−45.2	5.2	24.8	29.2
**def2-QZVPP**										
H_2O	PNO6	−76.066 419	−0.286 316	−0.008 766	−0.009 079	0.008				
[(**Por**)Ru(O)$_2$]	PNO6	−1226.244 167	−5.385 112	−0.324 567	−0.352 818	0.017				
[(**Por**)Ru(OH)(OOH)]	PNO6	−1302.381 105	−5.587 046	−0.298 969	−0.320 157	0.019	−44.3	8.7	30.3	34.9
H_2O	PNO7	−76.066 419	−0.286 099	−0.008 804	−0.009 126	0.008				
[(**Por**)Ru(O)$_2$]	PNO7	−1226.244 167	−5.388 177	−0.340 344	−0.371 352	0.019				
[(**Por**)Ru(OH)(OOH)]	PNO7	−1302.381 105	−5.593 704	−0.316 392	−0.340 781	0.022	−44.3	6.3	26.9	31.2
H_2O	PNO(6,7)	−76.066 419	−0.285 991	−0.008 824	−0.009 149					
[(**Por**)Ru(O)$_2$]	PNO(6,7)	−1226.244 167	−5.389 710	−0.348 232	−0.380 619					
[(**Por**)Ru(OH)(OOH)]	PNO(6,7)	−1302.381 105	−5.597 032	−0.325 103	−0.351 092		−44.3	5.1	25.2	29.4
**CBS(T,Q)**										
H_2O	PNO6	−76.067 003	−0.300 213	−0.009 801	−0.010 143					
[(**Por**)Ru(O)$_2$]	PNO6	−1226.251 305	−5.600 904	−0.340 414	−0.368 731					
[(**Por**)Ru(OH)(OOH)]	PNO6	−1302.388 613	−5.817 897	−0.315 432	−0.336 898		−44.1	8.1	29.9	34.4
H_2O	PNO7	−76.067 003	−0.299 852	−0.009 852	−0.010 205					
[(**Por**)Ru(O)$_2$]	PNO7	−1226.251 305	−5.600 464	−0.357 979	−0.389 203					
[(**Por**)Ru(OH)(OOH)]	PNO7	−1302.388 613	−5.820 924	−0.334 623	−0.359 430		−44.1	5.7	26.5	30.8
H_2O	PNO(6,7)	−76.067 003	−0.299 671	−0.009 878	−0.010 236					
[(**Por**)Ru(O)$_2$]	PNO(6,7)	−1226.251 305	−5.600 244	−0.366 761	−0.399 441					
[(**Por**)Ru(OH)(OOH)]	PNO(6,7)	−1302.388 613	−5.822 438	−0.344 218	−0.370 697		−44.1	4.5	24.8	29.0

The reaction sequence of a water-assisted epoxidation involving a hydroperoxo intermediate **B**, [(**L1**)Ru(OH)(OOH)] — computed here with a single water molecule only, ignoring potential neighboring water particles, which may induce acid/base catalysis — commences on the closed-shell singlet spin surface from ^{1}**A** with addition of a water molecule hydrogen-bonded to the oxo-ruthenium moiety (^{1}**A**$_{\mathbf{w1}}$), see Figure S27. Rearrangement to the hydroxo-hydroperoxo intermediate ^{1}**B** is energetically strongly disfavored. If this intermediate were accessible, however, the triplet spin state of this intermediate ^{3}**B** would be close in energy, and could proceed via oxygen atom transfer to the substrate yielding bis-hydroxo ruthenium species ^{3}**C**, [(**L1**)Ru(OH)$_2$], in its triplet ground state, which then may undergo oxo-hydroxo tautomerization towards ^{3}**D**$_{\mathbf{w1}}$, [(**L1**)Ru(O)(OH$_2$)]. The reaction profile for the two spin-state reaction surfaces is sketched in Figure S28.

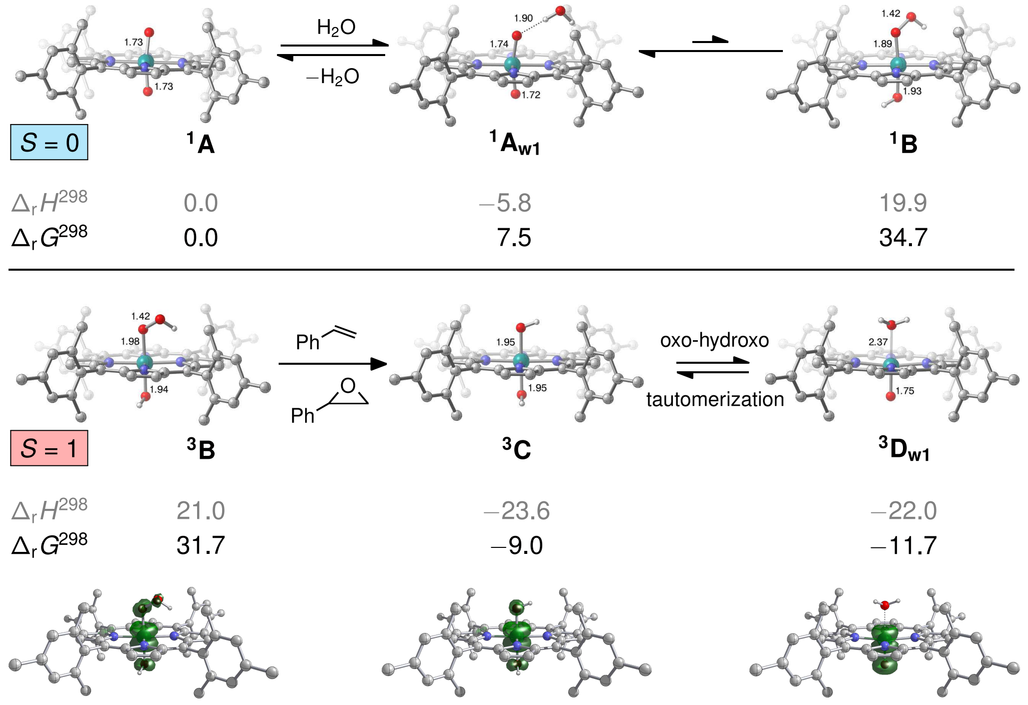

Figure S27: Selected intermediates of a water-assisted epoxidation cycle involving hydroperoxo intermediate **B**, [(**L1**)Ru(OH)(OOH)]. Relative enthalpies and Gibbs energies, computed at the M06L-D3(SMD)/def2-TZVP level, are given in kcal mol^{-1} for the lowest-energy closed-shell singlet (S = 0) and triplet (S = 1) species, respectively. For the latter, the corresponding spin density isosurfaces are plotted beneath at 0.01 a_0^{-3}.

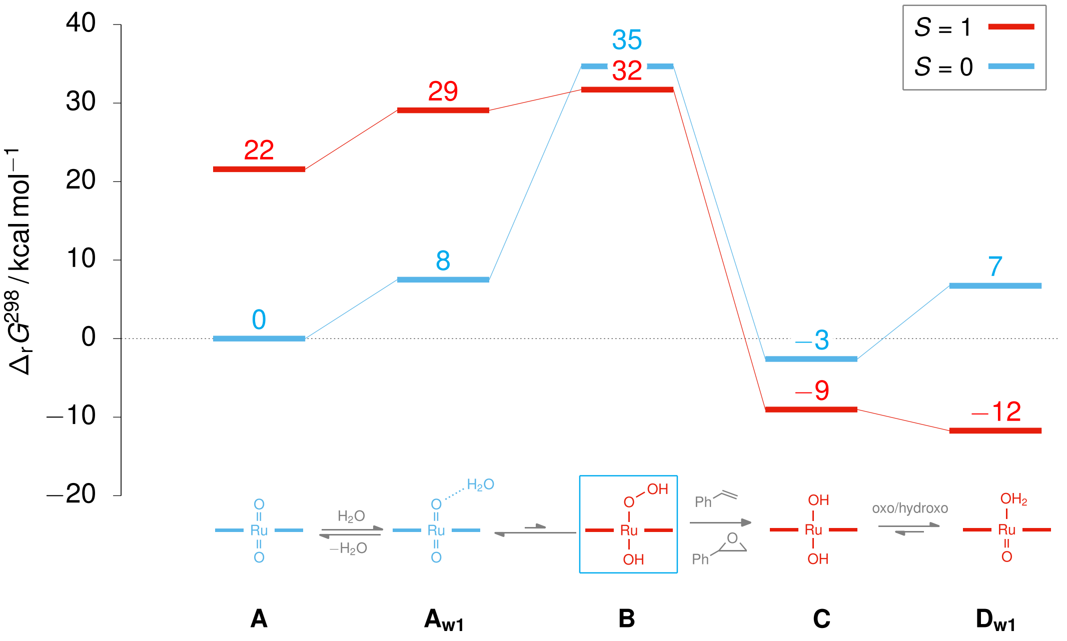

Figure S28: Reaction profile for selected intermediates of the water-assisted epoxidation cycle involving hydroperoxo intermediate **B**, [(**L1**)Ru(OH)(OOH)]. Relative Gibbs energies, computed at the M06L-D3(SMD)/def2-TZVP level, are given in kcal mol^{-1} for the lowest-energy closed-shell singlet (S = 0, blue) and triplet (S = 1, red) species, respectively.

Table S8: Total energies (Hartree) for individual species corresponding to Figure S28, including spin expectation values, DFT-corrections to enthalpies and to Gibbs energies. Relative energies (kcal mol^{-1}) are also given for comparison.

M06L-D3(SMD)/def2-TZVP							
Species[a]	$\langle S^2 \rangle$	E_{tot}	H_{tot}^{298}	G_{tot}^{298}	E_{rel}	H_{rel}	G_{rel}
H_2O	0.000	−76.450 684	0.024 877	0.003 447			
**1**	0.000	−309.720 509	0.141 252	0.102 735			
**1a**	0.000	−384.949 891	0.147 050	0.106 730			
^{1}**A**	0.000	−2630.475 908	0.994 559	0.824 803	0.0	0.0	0.0
^{1}**A**$_{\mathbf{w1}}$	0.000	−2706.938 381	1.022 025	0.851 994	−7.4	−5.8	7.5
^{1}**B**	0.000	−2706.897 520	1.022 049	0.854 423	18.2	19.9	34.7
^{1}**C**	0.000	−2631.729 107	1.017 428	0.852 024	−20.0	−17.6	−2.6
^{1}**D**$_{\mathbf{w1}}$	0.000	−2631.708 596	1.017 156	0.846 387	−7.1	−4.9	6.7
^{3}**A**	2.031	−2630.432 916	0.990 929	0.816 197	27.0	24.7	21.6
^{3}**A**$_{\mathbf{w1}}$	2.032	−2706.899 209	1.019 357	0.847 196	17.2	17.1	29.1
^{3}**B**	2.014	−2706.894 807	1.021 036	0.847 006	20.0	21.0	31.7
^{3}**C**	2.014	−2631.737 874	1.016 691	0.850 530	−25.5	−23.6	−9.0
^{3}**D**$_{\mathbf{w1}}$	2.013	−2631.735 895	1.017 315	0.844 246	−24.3	−22.0	−11.7

[a]State denoted by superscript: ^{1} (RKS singlet); ^{3} (UKS triplet); single water ligation is indicated with subscript '**w1**'.

7.3 Direct epoxidation via oxygen atom transfer

Direct epoxidation of styrene by [(**L1**)Ru(O)$_2$], **A**, yields a monoxo-ruthenium epoxide, **F**, which is thermochemically accessible at $\Delta_r H^{298}$ = -12 kcal mol^{-1} and $\Delta_r G^{298}$ = +6 kcal mol^{-1}. The resulting complex, however, has a triplet ground state, -17 kcal mol^{-1} below the corresponding singlet. The net reaction, **^{1}A** $\longrightarrow$ **^{3}F**, of Figure S29 thus involves a spin-crossing from the closed-shell singlet to the triplet spin surface; this is analogous to the epoxidation reaction with the tetra-(pentafluorophenyl) substituted congener [(**L3**)Ru(O)$_2$] reported in Ref. 42.

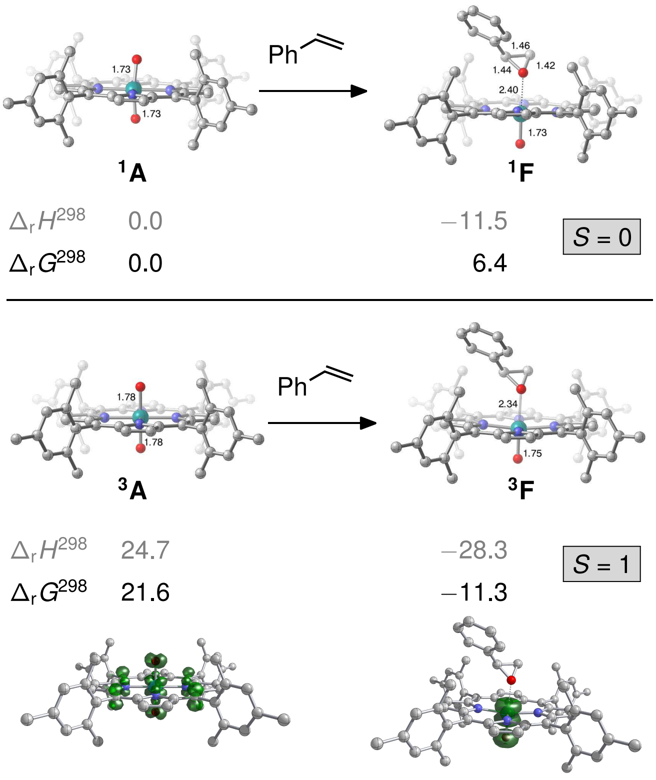

Figure S29: Relative enthalpies and Gibbs energies for the epoxidation of styrene by [(**L1**)Ru(O)$_2$], computed at the M06L-D3(SMD)/def2-TZVP level, are given in kcal mol^{-1} for the lowest-energy closed-shell singlet (S = 0) and triplet (S = 1) species, respectively. For the latter, the corresponding spin density isosurfaces are plotted beneath at 0.01 a_0^{-3}.

Figure S30 shows the corresponding reaction sequence with an initial barrier of 26 kcal mol^{-1} for the addition of styrene to the ruthenium dioxo complex [(**L1**)Ru(O)$_2$], **^{1}A**, forming a closed shell adduct, **^{1}E**, 19 kcal mol^{-1} above the reactants. Prior to formation of this adduct, the computed lowest free energy pathway involves a spin-surface crossing towards the corresponding triplet species via a minimum energy crossing point (**^{1,3}MECP**) at 23 kcal mol^{-1}. Following Popp and Stahl,[43] the free-energy correction for the MECP was estimated from the average of the Gibbs energy contributions from the adjacent minima. Without further inclusion of spin-orbit coupling terms and assuming a surface crossing probability smaller than unity, we introduce an additional energy penalty of 1–4 kcal mol^{-1} according to the suggestions of Harvey,[44] thus arriving at a range of 24–27 kcal mol^{-1} for the activation barrier related to this MECP. On the triplet spin surface the ruthenium–alkoxide complex **^{3}E** (ΔG = 7 kcal mol^{-1}) lies 12 kcal mol^{-1} below the corresponding singlet electromer. Commencing with a broken-symmetry singlet TS at 24 kcal mol^{-1} provides a slightly lower initial barrier that allows passage via the low-energy **^{BS,3}MECP** on the broken-symmetry singlet–triplet crossing seam at 12 kcal mol^{-1}, which translates to an energy estimate of 13–16 kcal mol^{-1}, and on the triplet surface then also leads to the ruthenium–alkoxide complex **^{3}E** at 7 kcal mol^{-1}. After passing a small barrier of 3 kcal mol^{-1} via **^{3}TS(E,F)** en route to the epoxide complex **^{3}F** at -11 kcal mol^{-1}, liberation of the epoxide product via a barrier of 8 kcal mol^{-1} through **^{3}TS(F,D)** leads to the coordinatively unsaturated ruthenium monoxide [(**L1**)Ru(O)], **D**.

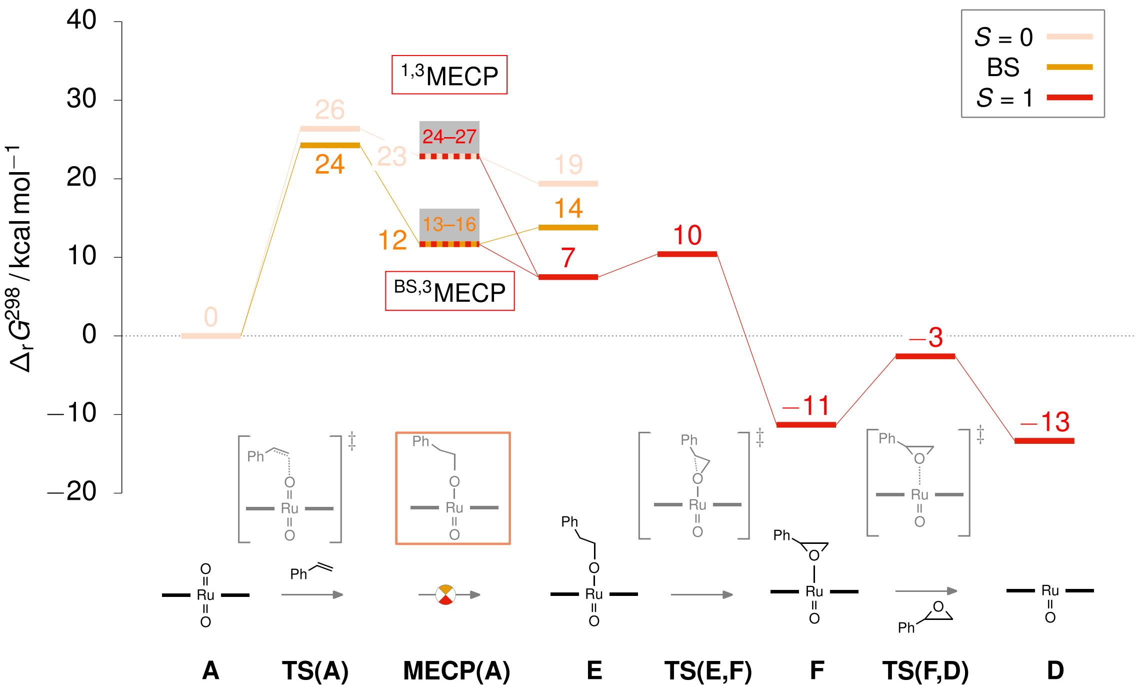

Figure S30: Reaction profile for selected intermediates of the anhydrous epoxidation cycle including two singlet-triplet MECP estimates and transition structures; relative Gibbs energies computed at the M06L-D3(SMD)/def2-TZVP level. Relative energies are given in kcal mol^{-1} for the lowest-energy closed-shell singlet (S = 0, light orange), broken-symmetry singlet (BS, orange) and triplet (S = 1, red) species, respectively.

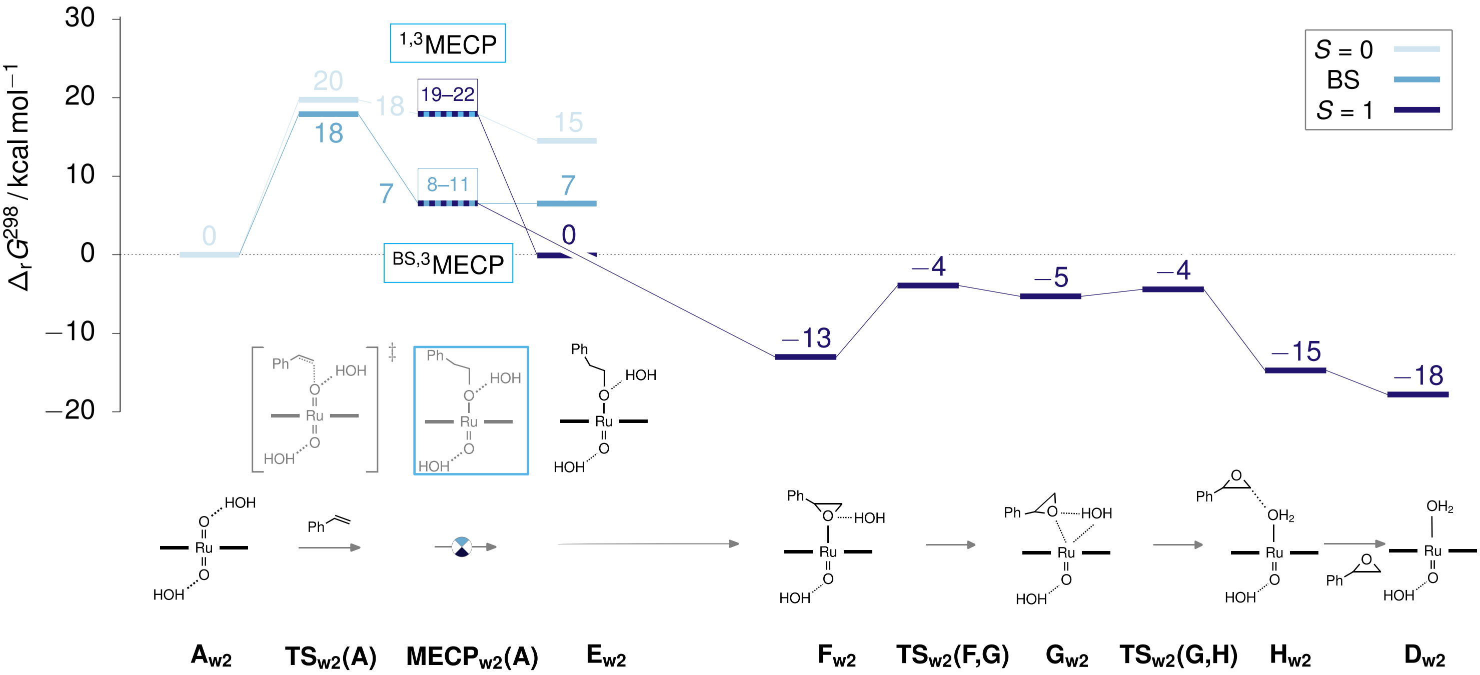

Figure S31: Reaction profile for selected intermediates of the microsolvated epoxidation cycle including two singlet-triplet MECP estimates and transition structures; relative Gibbs energies computed at the M06L-D3(SMD)/def2-TZVP level. Relative energies are given in kcal mol^{-1} for the lowest-energy closed-shell singlet (S = 0, light blue), broken-symmetry singlet (BS, blue) and triplet (S = 1, dark blue) species, respectively.

Introducing the effect of microsolvation through water ligation via a bis-aqua ligated ruthenium dioxo complex [(**L1**)Ru(O)$_2$(H$_2$O)$_2$], **^{1}A$_{w2}$**, significantly lowers the initial barrier for OAT by 6 kcal mol^{-1}, Figure S31. The initial transition structure **^{1}TS$_{w2}$(A)** for epoxide formation is located 20 kcal mol^{-1} above the reactants. A broken-symmetry TS is yet somewhat lower in energy. The lowest-energy path thus proceeds via **^{BS}TS$_{w2}$(A)** at 18 kcal mol^{-1} and passes a corresponding broken-symmetry singlet-triplet **$^{BS/3}$MECP$_{w2}$** (8–11 kcal mol^{-1}). In contrast to the complementary, energetically less favorable closed-shell singlet-triplet **$^{1/3}$MECP$_{w2}$**, the $^{BS/3}$MECP is a "late" structure on the potential energy surface closer to C–O bond formation, and it therefore yields swift downhill access to the triplet bis-aqua complexed ruthenium–epoxide **^{3}F$_{w2}$** at −13 kcal mol^{-1}, i.e., without passage of a ruthenium-alkoxide intermediate **^{3}E$_{w2}$** analogous to that of Figure S30. After liberation of the epoxide and a following rebound of the water molecule via **^{3}G$_{w2}$** and **^{3}H$_{w2}$**, the water-ligated ruthenium-oxo complex [(**L1**)Ru(OH$_2$)(O)(H$_2$O)], **^{3}D$_{w2}$**, is formed (see Figure S31).

Table S9: Total energies (Hartree) for individual species corresponding to Figure S30, including spin expectation values, DFT-corrections to enthalpies and to Gibbs energies. For transition structures the imaginary frequency is stated in cm^{-1}. Relative energies (kcal mol^{-1}) are also given for comparison.

**M06L-D3(SMD)/def2-TZVP**								
Species[a]	$\langle S^2 \rangle$	E_{tot}	H_{tot}^{298}	G_{tot}^{298}	ν_{imag}	E_{rel}	H_{rel}	G_{rel}
**1**	0.000	−309.720 509	0.141 252	0.102 735				
**1a**	0.000	−384.949 891	0.147 050	0.106 730				
**^{1}A**	0.000	−2630.475 908	0.994 559	0.824 803		0.0	0.0	0.0
**^{1}TS(A)**	0.000	−2940.186 685	1.136 683	0.959 820	548 i	6.1	6.7	26.4
**^{1,3}MECP(A)[singlet]**	0.000	−2940.190 935	1.137 347[d]	0.958 473[d]		3.4	4.4	22.9
**^{1,3}MECP(A)[triplet]**	2.033	−2940.190 934	1.137 347[d]	0.958 473[d]		3.4	4.4	22.9
**^{1}E**	0.000	−2940.197 218	1.138 004	0.959 226		−0.5	0.9	19.4
**^{BS}TS(A)**	0.268	−2940.187 407	1.136 368	0.959 511	720 i	5.7	6.0	25.7
**^{3}TS(A)**[b]	2.027	−2940.170 365				16.3		
**^{S}TS(A)**	0.268	−2940.189 742				4.2	4.5	24.3
**^{BS,3}MECP(A)[singlet]**[c]	1.042	−2940.209 217	1.136 853[e]	0.958 954[e]		−8.0	−7.4	11.7
**^{BS,3}MECP(A)[triplet]**	2.039	−2940.209 217	1.136 853[e]	0.958 954[e]		−8.0	−7.4	11.7
**^{BS}E**	1.024	−2940.210 392	1.137 016	0.960 188		−8.8	−8.0	11.7
**^{3}E**	2.055	−2940.214 659	1.136 690	0.957 720		−11.5	−10.9	7.5
**^{3}TS(E,F)**	2.052	−2940.212 588	1.137 252	0.960 293	136 i	−10.2	−9.2	10.4
**^{3}F**	2.013	−2940.245 197	1.139 535	0.958 299		−30.6	−28.3	−11.3
**^{3}TS(F,D)**	2.013	−2940.233 893	1.138 731	0.960 872	113 i	−23.5	−21.7	−2.6
**^{3}D**	2.013	−2555.270 105	0.989 677	0.823 112		−14.8	−14.2	−13.4

[a]State denoted by superscript: ^{1} (RKS singlet); ^{3} (UKS triplet); ^{BS} (UKS/broken-symmetry singlet); ^{S} (spin-projected BS singlet).

[b]Triplet energy was computed for spin projection, i.e., at the corresponding UKS/broken-symmetry singlet geometry.

[c]Spin-projection has no effect on **^{BS,3}MECP(A)[singlet]** because it is isoenergetic with the corresponding triplet species **^{BS,3}MECP(A)[triplet]**.

[d]Thermal contributions are taken from the average of the corresponding corrections to the adjacent singlet and triplet species **^{1}E** and **^{3}E**.

[e]Thermal contributions are taken from the average of the corresponding corrections to the adjacent broken-symmetry singlet and triplet species **^{BS}E** and **^{3}E**.

Table S10: Total energies (Hartree) for individual species corresponding to Figure S31, including spin expectation values, DFT-corrections to enthalpies and to Gibbs energies. For transition structures the imaginary frequency is stated in cm^{-1}. Relative energies ($kcal\,mol^{-1}$) are also given for comparison.

M06L-D3(SMD)/def2-TZVP								
Species[a]	$\langle S^2 \rangle$	E_{tot}	H_{tot}^{298}	G_{tot}^{298}	ν_{imag}	E_{rel}	H_{rel}	G_{rel}
**1**	0.000	−309.720 509	0.141 252	0.102 735				
**1a**	0.000	−384.949 891	0.147 050	0.106 730				
^{1}**A**$_{\mathbf{w2}}$	0.000	−2783.398 904	1.049 050	0.872 542		0.0	0.0	0.0
^{1}**TS**$_{\mathbf{w2}}$**(A)**	0.000	−3093.114 866	1.191 042	1.002 177	529 i	2.9	3.3	19.7
^{1,3}**MECP**$_{\mathbf{w2}}$**(A)[singlet]**	0.000	−3093.118 238	1.192 101[d]	1.002 698[d]		0.7	1.9	17.9
^{1,3}**MECP**$_{\mathbf{w2}}$**(A)[triplet]**	2.034	−3093.118 238	1.192 101[d]	1.002 698[d]		0.7	1.9	17.9
^{1}**E**$_{\mathbf{w2}}$	0.000	−3093.125 058	1.192 802	1.004 050		−3.5	−2.0	14.5
^{BS}**TS**$_{\mathbf{w2}}$**(A)**	0.252	−3093.115 469	1.190 749	1.001 935	688 i	2.5	2.8	19.2
^{3}**TS**$_{\mathbf{w2}}$**(A)**[b]	2.026	−3093.099 200				12.7		
^{S}**TS**$_{\mathbf{w2}}$**(A)**	0.252	−3093.117 537				1.2	1.5	17.9
^{BS,3}**MECP**$_{\mathbf{w2}}$**(A)[singlet]**[c]	0.970	−3093.136 793	1.190 212[e]	1.001 020[e]		−10.9	−11.0	5.3
^{BS,3}**MECP**$_{\mathbf{w2}}$**(A)[triplet]**	2.047	−3093.136 793	1.190 308[e]	0.999 779[e]		−10.9	−10.9	4.5
^{BS}**E**$_{\mathbf{w2}}$	0.977	−3093.137 118	1.192 258	1.002 699		−11.1	−9.9	6.1
^{3}**E**$_{\mathbf{w2}}$	2.034	−3093.145 611	1.191 399	1.001 345		−16.4	−15.8	−0.1
^{3}**F**$_{\mathbf{w2}}$	2.013	−3093.169 557	1.194 387	1.004 673		−31.5	−28.9	−13.0
^{3}**TS**$_{\mathbf{w2}}$**(F,G)**	2.013	−3093.158 353	1.195 435	1.007 979	185 i	−24.4	−21.2	−3.9
^{3}**G**$_{\mathbf{w2}}$	2.013	−3093.155 799	1.194 975	1.003 253		−22.8	−19.9	−5.3
^{3}**TS**$_{\mathbf{w2}}$**(G,H)**	2.013	−3093.155 642	1.193 925	1.004 495	117 i	−22.7	−20.5	−4.4
^{3}**H**$_{\mathbf{w2}}$	2.013	−3093.168 121	1.194 766	1.000 527		−30.6	−27.8	−14.7
^{3}**D**$_{\mathbf{w2}}$	2.013	−2708.199 896	1.045 014	0.870 566		−19.1	−18.0	−17.8

[a]State denoted by superscript: ^{1} (RKS singlet); ^{3} (UKS triplet); ^{BS} (UKS/broken-symmetry singlet); ^{S} (spin-projected BS singlet); microsolvation is indicated with subscript '**w2**'.

[b]Triplet energy was computed for spin projection, i.e., at the corresponding UKS/broken-symmetry singlet geometry.

[c]Spin-projection has no effect on ^{BS,3}**MECP**$_{\mathbf{w2}}$**(A)[singlet]** because it is isoenergetic with the corresponding triplet species ^{BS,3}**MECP**$_{\mathbf{w2}}$**(A)[triplet]**.

[d]Thermal contributions are taken from the average of the corresponding corrections to the adjacent singlet and triplet species ^{1}**E**$_{\mathbf{w2}}$ and ^{3}**E**$_{\mathbf{w2}}$.

[e]Thermal contributions are taken from the average of the corresponding corrections to the adjacent broken-symmetry singlet and triplet species ^{BS}**E**$_{\mathbf{w2}}$ and ^{3}**E**$_{\mathbf{w2}}$.

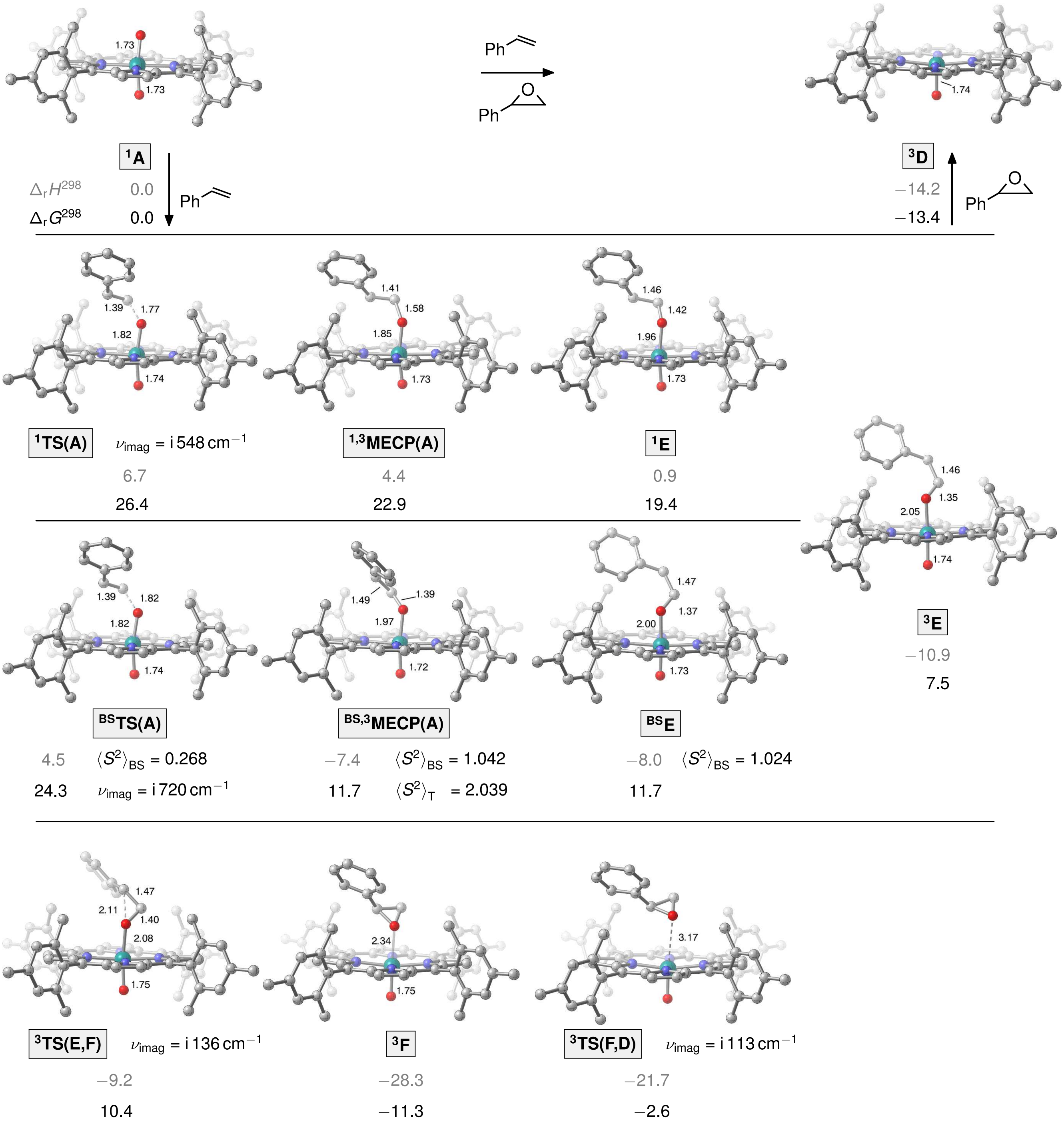

Figure S32: Molecular geometries of structures for anhydrous epoxide formation from styrene and **^{1}A** to **^{3}D** via **^{1}TS(A)**, **^{1,3}MECP(A)**, **^{1}E**, **^{3}E**, **^{BS}TS(A)**, **^{BS,3}MECP(A)**, **^{BS}E**, **^{3}TS(E,F)**, **^{3}F**, **^{3}TS(F,D)**. The relative energies $\Delta_r H^{298}$ and $\Delta_r G^{298}$ are given in kcal mol^{-1}.

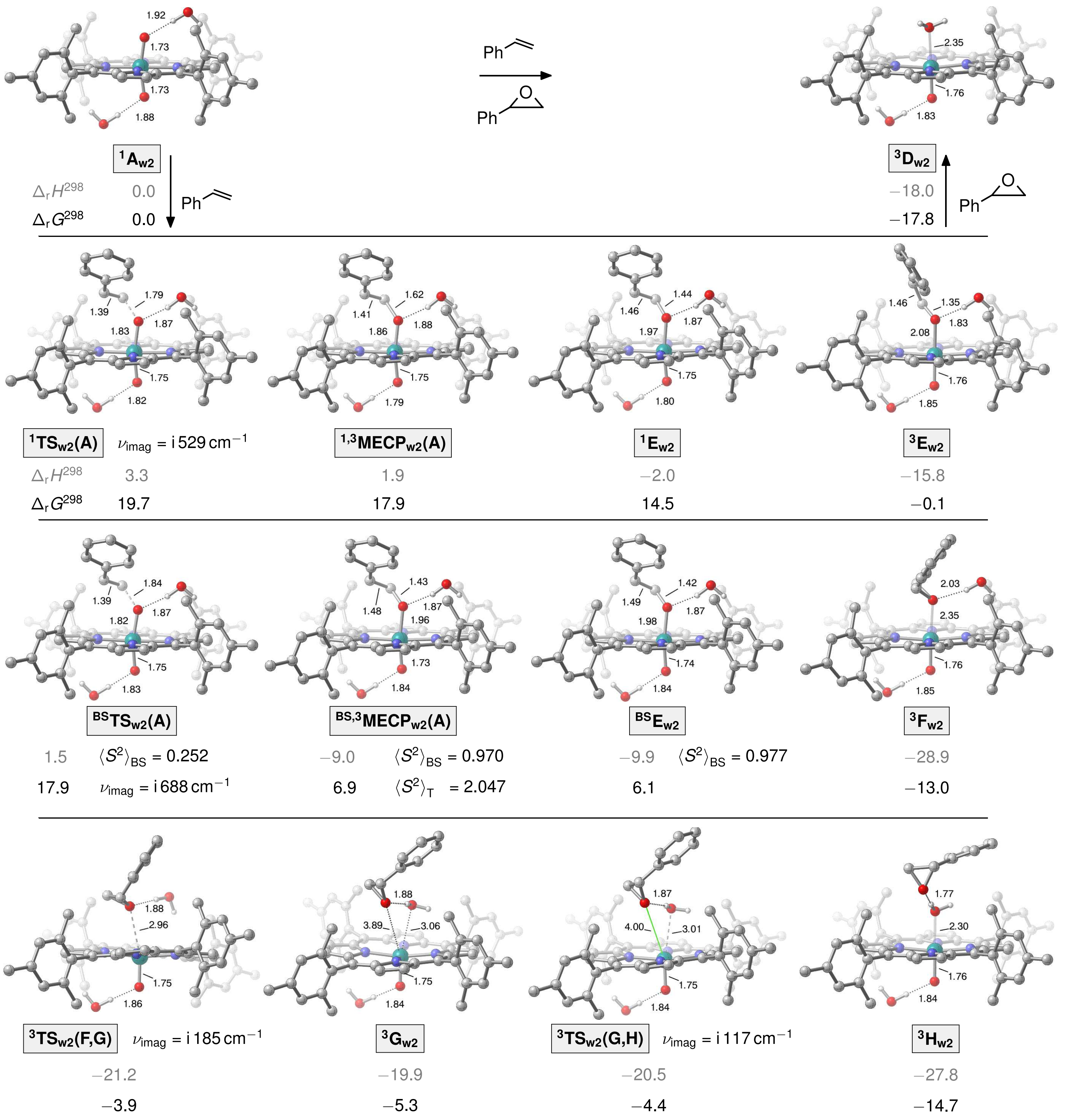

Figure S33: Molecular geometries of structures for microsolvated epoxide formation from styrene and ^{1}**A$_{w2}$** to ^{3}**D$_{w2}$** via ^{1}**TS$_{w2}$(A)**, ^{1,3}**MECP$_{w2}$(A)**, ^{1}**E$_{w2}$**, ^{3}**E$_{w2}$**, as well as ^{BS}**TS$_{w2}$(A)**, ^{BS,3}**MECP$_{w2}$(A)**, ^{BS}**E$_{w2}$**, ^{3}**F$_{w2}$**, including water rebound intermediates ^{3}**TS$_{w2}$(F,G)**, ^{3}**G$_{w2}$**, ^{3}**TS$_{w2}$(G,H)**, ^{3}**H$_{w2}$**. The relative energies $\Delta_r H^{298}$ and $\Delta_r G^{298}$ are given in kcal mol^{-1}.

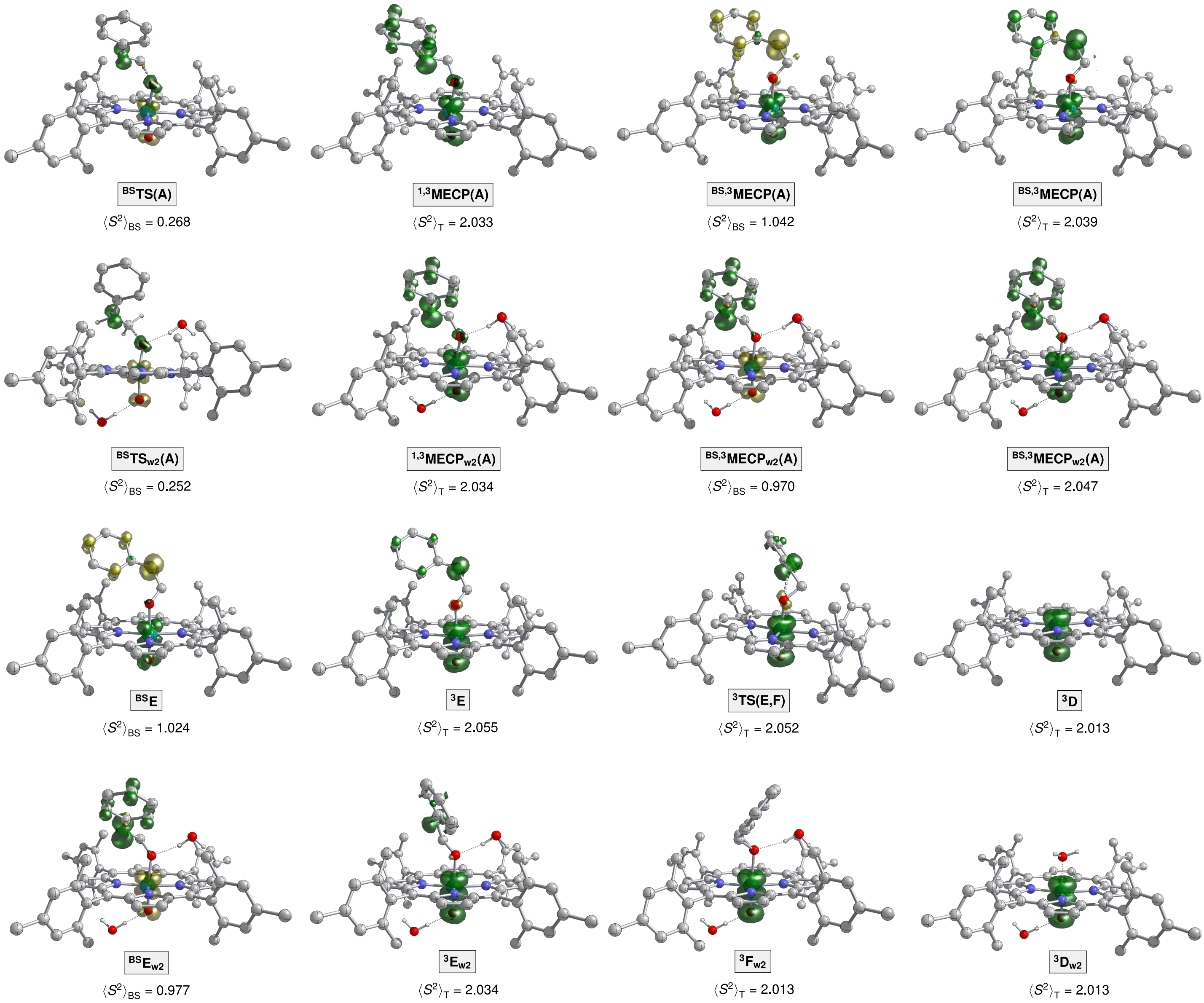

Figure S34: Spin density distributions computed at the M06L-D3(SMD)/def2-TZVP level for **^{BS}TS(A)**, **^{1,3}MECP(A)**, **^{BS,3}MECP(A)** (singlet and triplet), **^{BS}TS$_{w2}$(A)**, **^{1,3}MECP$_{w2}$(A)**, **^{BS,3}MECP$_{w2}$(A)** (singlet and triplet), **^{BS}E**, **^{3}E**, **^{3}TS(E,F)**, **^{3}D**, **^{BS}E$_{w2}$**, **^{3}E$_{w2}$**, **^{3}F$_{w2}$**, **^{3}D$_{w2}$**; α-spin: green, β-spin: yellow; isocontour surfaces at 0.01 a_0^{-3}.

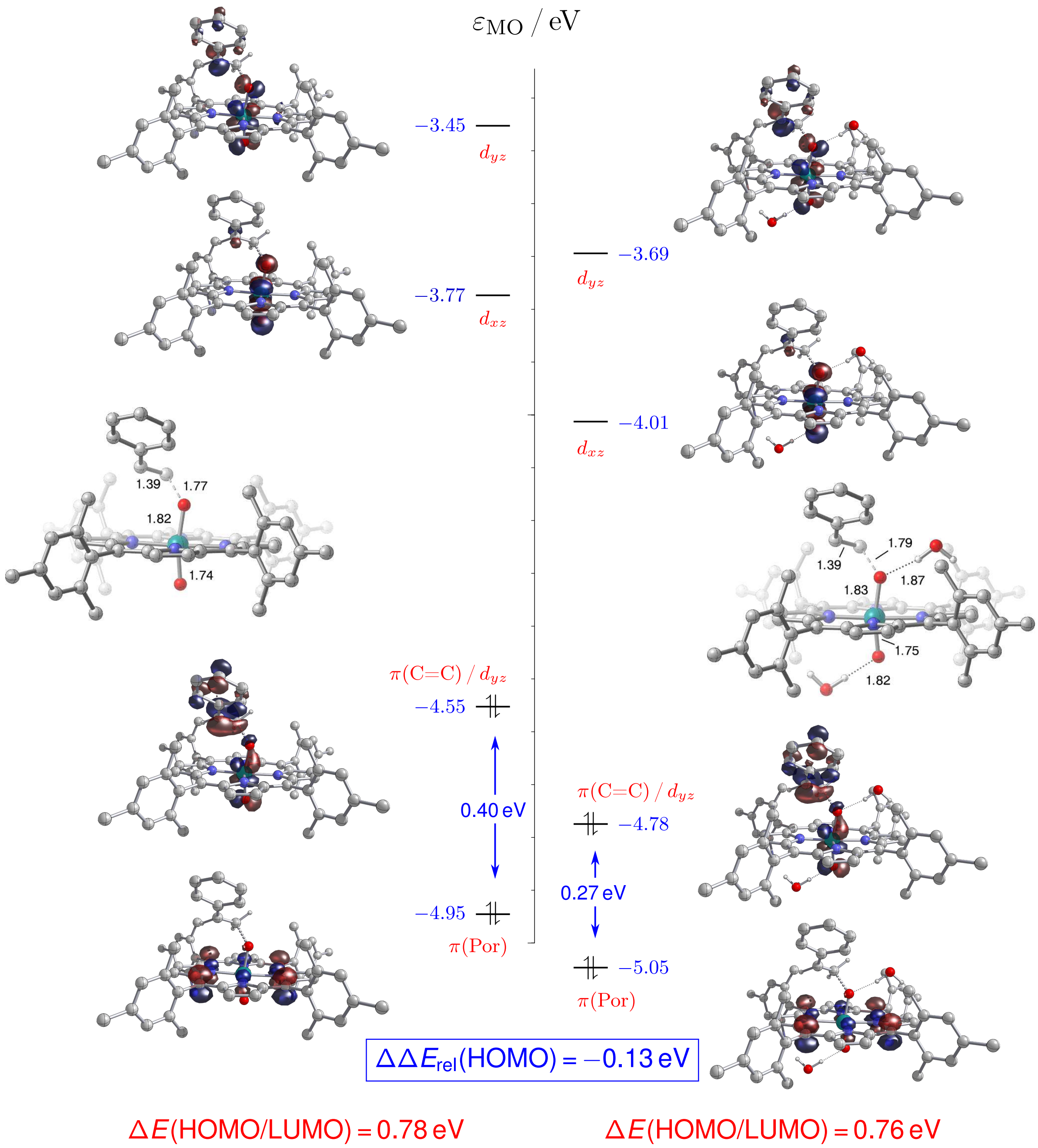

Figure S35: Molecular orbital energy diagram for the initial transition structures **[1]TS(A)** (left) and **[1]TS_{w2}(A)** (right) for anhydrous and microsolvated styrene epoxidation, respectively, showing selected frontier orbitals computed at the M06L-D3(SMD)/def2-TZVP level. The relative stabilization ΔΔE_{rel}(HOMO) of the highest occupied molecular orbital (HOMO) is computed with reference to the corresponding second-highest occupied molecular orbital (HOMO-1), which is orthogonal to the O=Ru=O axis. Isocontour surfaces at 0.05 $a_0^{-3/2}$, orbital energies are given in eV.

7.4 Cartesian coordinates of optimized geometries (Å)

7.4.1 Species pertinent to formation of hydroxo-hydroperoxo intermediate

3
H_2O E_{tot}(RM06L-D3(Dichloromethane)/def2TZVP/W06) = -76.45068431 $\langle S^2 \rangle$ = 0.0000

O	0.000000000000	0.000000000000	-0.065766315076
H	-0.757464069524	0.000000000000	0.528867019538
H	0.757464069524	0.000000000000	0.528867019538

16
1 (styrene, Ph-CH=CH_2) E_{tot}(RM06L-D3(Dichloromethane)/def2TZVP/W06) = -309.72050871 $\langle S^2 \rangle$ = 0.0000

C	0.534869654560	-0.219380807822	-0.000000000383
C	0.031754130787	1.087085244492	-0.000000000272
C	-1.330778083155	1.321961566475	-0.000000001405
C	-2.228099711329	0.259692224603	-0.000000000891
C	-1.747226756079	-1.040932545502	0.000000003295
C	-0.381432201616	-1.275345632262	0.000000000995
H	0.717656093519	1.925436386758	-0.000000001119
H	-1.699209635201	2.340295966028	-0.000000000134
H	-3.294196741347	0.448016005476	0.000000000122
H	-2.436985471390	-1.875659363109	-0.000000000976
H	-0.006848349113	-2.292741394018	0.000000001277
C	1.963492682213	-0.520122888959	-0.000000000882
H	2.200568974867	-1.580900354140	-0.000000003091
C	2.970465078062	0.351282475156	-0.000000000094
H	3.998966531802	0.016087538791	-0.000000000092
H	2.816794547420	1.424028070033	0.000000003650

17
1a (styrene epoxide, Ph-CH(O)CH_2) E_{tot}(RM06L-D3(Dichloromethane)/def2TZVP/W06) = -384.94989120 $\langle S^2 \rangle$ = 0.0000

C	0.029850925598	0.545043194850	-0.165513243157
C	-0.972651340616	-0.417215609665	-0.272103033214
C	-0.669581029281	-1.762539380208	-0.142778934041
C	0.638182149427	-2.165321298918	0.096776583632
C	1.642051501574	-1.213513025457	0.199507843017
C	1.339691771974	0.133385022083	0.064396406492
H	-1.990204807208	-0.101361557578	-0.470174943383
H	-1.456068254297	-2.501649611013	-0.231691418046
H	0.873354079162	-3.217389322059	0.196386069637
H	2.665017133827	-1.520112862735	0.378502017845
H	2.125132412379	0.877250903964	0.135529652220
C	-0.277860836151	1.987417644682	-0.280658494308
H	0.581248938826	2.628887459325	-0.464476673766
C	-1.434017898509	2.593700923013	0.372748318440
H	-1.401878264274	3.638796008709	0.669683172809
H	-2.096723554371	1.962867089494	0.960606707789
O	-1.441916543060	2.351004360514	-1.019509849963

39
^{1}[(Por)Ru(O)$_2$] E_{tot}(RM06L-D3(Dichloromethane)/def2TZVP/W06) = -1234.13337374 $\langle S^2 \rangle$ = 0.0000

Ru	-0.038099379040	-0.000623976723	-0.000000542858
O	0.148878622132	0.001807714465	1.713554557590
O	0.148878436042	0.001807905228	-1.713555523225
C	3.411447094203	0.113279049626	0.000000109121
C	-0.118346243064	3.423816348437	0.000000185305
C	-3.440474472685	-0.113967162313	0.000000159408
C	0.108877206178	-3.423469245864	0.000000152171
N	-1.521827122830	1.406411783021	-0.000000172133
N	1.411771112735	1.525463580649	-0.000000309438
N	1.510039201055	-1.428519034056	-0.000000473404
N	-1.425800618625	-1.503625950065	-0.000000293289
C	1.340608741115	-2.774633854536	0.000000071680
C	2.842194540397	-1.156352962644	0.000000036555
C	2.759530589883	1.342428334909	-0.000000011773
C	1.153415279608	2.857310732053	0.000000028466
C	-1.154079326342	-2.845471018514	0.000000016659
C	-2.781799381542	-1.337152509542	0.000000013389
C	-2.863917910500	1.149934793638	0.000000161396
C	-1.340273111812	2.763703511398	0.000000169131
C	2.634941972494	-3.397945647207	0.000000715572
C	3.557858675022	-2.402480901589	0.000000688632

C	3.390474874279	2.633560622893	0.000000392238
C	2.403161996158	3.565222100480	0.000000414042
C	-2.629017065624	3.386735858356	0.000000626182
C	-3.562265660574	2.398566841547	0.000000619780
C	-2.398242026372	-3.553321166492	0.000000387225
C	-3.395460514922	-2.629753739833	0.000000374818
H	2.802151995325	-4.463742232560	0.000001129957
H	4.633251687621	-2.489405973660	0.000001077891
H	4.457632915033	2.792309560306	0.000000633906
H	2.498768819218	4.639794262833	0.000000671874
H	-2.789013540115	4.453628415071	0.000000912782
H	-4.636557175619	2.496397391491	0.000000911382
H	-2.486233383157	-4.628544322930	0.000000634274
H	-4.460816987122	-2.799260082273	0.000000610960
H	4.493451465035	0.148773259923	0.000000465627
H	-0.162637161814	4.505298236722	0.000000481551
H	-4.522028758801	-0.149337842250	0.000000432721
H	0.135256795019	-4.505553220065	0.000000525769

42

^{1}**[(Por)Ru(OH)(OOH)]** E_{tot}(RM06L-D3(Dichloromethane)/def2TZVP/W06) = -1310.55309593 $\langle S^2 \rangle$ = 0.0000

Ru	0.033485850279	0.000000208307	-0.086033825692
O	0.090358917645	-0.000001266180	-2.010680674055
O	0.042613562079	0.000000026108	1.794464605727
C	3.481722694804	-0.000016012125	0.007077672435
C	0.045629121981	-3.404975675487	-0.090011247884
C	-3.393823468372	0.000016050761	-0.206792357012
C	0.045661144887	3.404975777746	-0.090010614195
N	-1.419436005477	-1.442080153340	-0.136869726833
N	1.509934343644	-1.446254279918	-0.048861315918
N	1.509947803614	1.446240808109	-0.048861287533
N	-1.419422380831	1.442093737424	-0.136868271160
C	1.292871348415	2.793377463301	-0.054991766939
C	2.861603155295	1.242646759624	-0.009225252299
C	2.861591586832	-1.242673027045	-0.009225183778
C	1.292845059455	-2.793388920637	-0.054991914772
C	-1.199868768266	2.789074201332	-0.128276402775
C	-2.772901415189	1.240771483225	-0.182974948823
C	-2.772912968613	-1.240745217272	-0.182976174322
C	-1.199895082690	-2.789062597160	-0.128277782932
C	2.558330485171	3.468063585991	-0.016790046956
C	3.523881591455	2.513527832444	0.011323419883
C	3.523857955747	-2.513560277020	0.011323588647
C	2.558297736919	-3.468086930564	-0.016789989285
C	-2.464354398837	-3.464302910175	-0.167868132624
C	-3.431966510502	-2.511162592190	-0.202412926929
C	-2.464321686699	3.464326237204	-0.167866477190
C	-3.431942872282	2.511194989061	-0.202411312927
H	2.680097572682	4.540313585253	-0.012012800978
H	4.594161723582	2.646046273703	0.043083464256
H	4.594136794799	-2.646088956909	0.043083690144
H	2.680054668106	-4.540338106369	-0.012012754827
H	-2.584591959567	-4.536753406851	-0.170254506876
H	-4.501762463279	-2.645915735237	-0.238296056480
H	-2.584549199189	4.536777896559	-0.170252804180
H	-4.501737545611	2.645958345924	-0.238294362855
H	4.563849271441	-0.000020977021	0.038190851900
H	0.043039534647	-4.487705145748	-0.088820988462
H	-4.475598050744	0.000021074203	-0.246277207172
H	0.043081327269	4.487705225789	-0.088820432325
H	1.008598556919	-0.000000516578	-2.320715699313
O	-1.235661137113	0.000000390054	2.437326030528
H	-0.963642967923	-0.000004034091	3.371239554180

118

^{1}**A_{w1}** E_{tot}(RM06L-D3(Dichloromethane)/def2TZVP/W06) = -2706.93838081 $\langle S^2 \rangle$ = 0.0000

Ru	0.296535296509	-0.010779474669	0.073750275269
O	0.033308959546	-0.140197608436	1.766882575681
C	3.721925613838	-0.128905467641	0.290324473104
C	0.365160687115	3.421314868326	0.032429942991
C	-3.163098826059	0.053111098277	-0.209291525171
C	0.179296975806	-3.483747790634	0.104114658773
N	-1.138068589926	1.473303387830	-0.054660156523
N	1.761543315049	1.378916761474	0.142464815675
N	1.702149428108	-1.531481388047	0.186732821738
N	-1.238242907885	-1.471910589350	-0.016345316630

C	1.434925658181	-2.872993998953	0.196492343998
C	3.048855041571	-1.358181880976	0.277577177245
C	3.110316824954	1.124268595615	0.225050284684
C	1.582106545956	2.741668699273	0.110934350871
C	-1.050211139778	-2.818243738985	-0.009586176613
C	-2.565674955650	-1.214975302940	-0.154594241031
C	-2.489463950582	1.278126611300	-0.152330818660
C	-0.894862533538	2.813176041472	-0.042899758127
C	2.680371243874	-3.574753782454	0.307184873836
C	3.670068110630	-2.646932822495	0.354310828519
C	3.799591904510	2.375759372742	0.241327345557
C	2.867886063437	3.361954219329	0.172953949399
C	-2.146231096332	3.503907304289	-0.129679242559
C	-3.122812128809	2.563219230199	-0.197467823736
C	-2.330507738425	-3.454032160053	-0.155769891983
C	-3.261896098765	-2.468575550108	-0.245972237839
H	2.778891881677	-4.648275469110	0.339830671717
H	4.732353906388	-2.815216130582	0.434201816898
H	4.871210705109	2.482864543758	0.299155315536
H	3.034505992732	4.427467332819	0.164357987288
H	-2.257636516183	4.576588718791	-0.140032337892
H	-4.187133020417	2.720317473192	-0.273593058335
H	-2.490991498193	-4.520348574854	-0.182642680071
H	-4.329130195696	-2.575412588446	-0.360408270184
C	5.210927913164	-0.164809819023	0.375525869319
C	5.840953410363	-0.140673911779	1.625442992511
C	5.973794249811	-0.228997241640	-0.800246604893
C	7.232128959796	-0.182559906665	1.678270596523
C	7.359505096266	-0.268112818576	-0.700646131244
H	7.717813439121	-0.165770992327	2.648389959037
H	7.946449677102	-0.317977248797	-1.612227327422
C	8.011002706809	-0.246224228015	0.529636511565
C	0.408880614115	4.912439379579	0.024838351267
C	0.461532613056	5.603978044103	-1.194723996779
C	0.396818082599	5.615678755079	1.235449409251
C	0.502715866080	6.993120391806	-1.177780304404
C	0.438521760913	7.007517080915	1.205434567024
H	0.545262918245	7.524852674268	-2.123000149623
H	0.428688498474	7.550157589318	2.145041897466
C	0.491756954433	7.716607220860	0.011819943255
C	-4.647523265850	0.095224222534	-0.351384630018
C	-5.220310803321	0.200896802726	-1.625851495105
C	-5.461752246855	0.028090316321	0.787404866706
C	-6.607019706232	0.230204225412	-1.738373071894
C	-6.843280451335	0.064132894361	0.628310660040
H	-7.047809208812	0.311866615416	-2.726615590533
H	-7.471107837445	0.017331375152	1.512216859338
C	-7.438140413353	0.160622093202	-0.625583892314
C	0.141248237466	-4.974885837804	0.120744624082
C	-0.082617529993	-5.652224550314	1.328854825734
C	0.321933665522	-5.690809820522	-1.068581181631
C	-0.122780187965	-7.041345927530	1.321999093548
C	0.270615441409	-7.082259172224	-1.029250335528
H	-0.294119878239	-7.562469887350	2.258549047015
H	0.406300642725	-7.635256359234	-1.952971054404
C	0.049731456342	-7.777870638452	0.152893511260
C	-4.860809593365	-0.076395240944	2.153265210454
H	-5.628645724194	-0.059805748619	2.924055909291
H	-4.289825636742	-0.999494101949	2.273386785483
H	-4.166572217200	0.742035609834	2.353186905493
C	-4.360993249726	0.290944497365	-2.846413726455
H	-3.783718670769	1.218361953377	-2.862086093762
H	-3.631484754989	-0.520378521186	-2.896567411484
H	-4.960611207913	0.261486579100	-3.754015308129
C	-8.925364649017	0.161378978154	-0.772940811196
H	-9.240347390671	0.674391839795	-1.680424287411
H	-9.314837978460	-0.857604663329	-0.831884035588
H	-9.414612757987	0.636879799376	0.076242172059
C	-0.002251911105	-9.271357904871	0.177335409713
H	-0.962340362558	-9.630830499667	0.551179376568
H	0.148604063529	-9.695068825086	-0.813753736865
H	0.761579742880	-9.685986384249	0.837219845807
C	-0.275938544602	-4.896876952123	2.605494351866
H	-0.370358547987	-5.573153996259	3.452646571001
H	0.557576345628	-4.221008459781	2.806083519253
H	-1.173863373571	-4.275974460193	2.575136964760
C	0.572028348766	-4.980637948810	-2.360621951000

H	0.559017521884	-5.672827389120	-3.200213574541
H	-0.168889264043	-4.201332819839	-2.552177208990
H	1.543846859629	-4.481343366357	-2.360900620294
C	5.040824914349	-0.071864505007	2.887278551916
H	4.427120190453	0.830313255851	2.927136925140
H	4.353532695805	-0.915410107126	2.976553094899
H	5.686082942349	-0.073707312271	3.763397890904
C	5.311746599005	-0.255593490923	-2.141209750536
H	6.046378146310	-0.302196789822	-2.942433462880
H	4.647575154841	-1.115703059999	-2.246895879814
H	4.694841320974	0.630286720703	-2.304426158229
C	9.503142687748	-0.285685433111	0.604574428429
H	9.853035998617	-0.316601038338	1.634688035128
H	9.906078579242	-1.157611797151	0.087301834611
H	9.948504318895	0.589829728284	0.128797343522
C	0.342599854724	4.891487621786	2.543143850073
H	-0.530946203759	4.240064106770	2.608702041422
H	1.215159632573	4.250738897801	2.685923090556
H	0.302801392427	5.588104060795	3.377988509278
C	0.530891611660	9.210554183432	-0.003081726367
H	-0.352121818075	9.626476547582	-0.491515236099
H	0.576508580821	9.621452152593	1.003709208272
H	1.394620766460	9.581965472793	-0.556401002936
C	0.474957148950	4.863602234805	-2.494301905119
H	1.318620454827	4.173654973428	-2.557462647493
H	-0.425588293039	4.260112423862	-2.624473009426
H	0.540310041926	5.548464498536	-3.337195720794
O	0.257470652238	-0.200859746778	-1.652332446046
H	-1.954576120682	-2.388106951509	-2.578259029549
O	-1.392969080034	-1.943275238554	-3.224813011064
H	-0.834589850183	-1.355275643801	-2.685246866402

115

^{1}A ≡ ^{1}[(**L1**)Ru(O)$_2$] E_{tot}(RM06L-D3(Dichloromethane)/def2TZVP/W06) = -2630.47590821 $\langle S^2 \rangle$ = 0.0000

Ru	-0.027936484506	-0.003174879387	-0.000170980489
O	0.174036211567	-1.716652716539	0.001009562354
O	0.173677607402	1.710409215349	-0.001328218989
C	3.451813679285	-0.001917515565	0.000029394919
C	0.007165953626	-0.000102212170	3.451265828986
C	-3.456953935904	-0.002826723602	-0.000375010753
C	0.007632164969	-0.004442558486	-3.451604954862
N	-1.460054229193	-0.002312607434	1.452045485408
N	1.472051336102	-0.002006826182	1.476046748590
N	1.472281376292	-0.003970343118	-1.476215285294
N	-1.459833733352	-0.004248097621	-1.452557780695
C	1.258310523973	-0.004232332615	-2.816960047579
C	2.814846204276	-0.003113320568	-1.248119754634
C	2.814629187569	-0.001609596832	1.248103739063
C	1.257912296780	-0.000782204285	2.816763587073
C	-1.232676441823	-0.004631514727	-2.806089558429
C	-2.812145628978	-0.003901752048	-1.241483687462
C	-2.812352144910	-0.002195273535	1.240817257052
C	-1.233068270770	-0.000964150128	2.805608108280
C	2.531148649963	-0.003742751447	-3.481686174857
C	3.486414334357	-0.003046688572	-2.518216158907
C	3.486048997595	-0.000291259154	2.518295974734
C	2.530667621707	0.000228272384	3.481643257367
C	-2.501176723833	-0.000131543716	3.468406009791
C	-3.466319345821	-0.000888916561	2.512460534024
C	-2.500709736792	-0.004738229744	-3.469040349368
C	-3.465966116201	-0.004304459103	-2.513217802038
H	2.669028567158	-0.003919242471	-4.551442852969
H	4.557155890248	-0.002554643619	-2.648448392364
H	4.556775848655	0.000171579970	2.648700073653
H	2.668426582911	0.001178592250	4.551414292710
H	-2.632859480914	0.000884867665	4.538892569194
H	-4.535651594171	-0.000605143658	2.653013991652
H	-2.632274308614	-0.005107672470	-4.539541000977
H	-4.535287026542	-0.004257095450	-2.653931440039
C	4.943878259378	0.000302277118	0.000473329199
C	5.643097916475	-1.212492030208	0.001404708501
C	5.639629978576	1.218389859875	0.002355445432
C	7.035696246002	-1.186060725260	0.004244577857
C	7.029392401050	1.198215403035	0.005141255408
H	7.575443666798	-2.127422726294	0.006123434577
H	7.564726162581	2.142417068840	0.007705261392
C	7.749140802520	0.006254281186	0.005862217510

C	0.000944925125	0.002691062662	4.943287341639
C	-0.001460129882	1.220904412516	5.638937934732
C	-0.000828445362	-1.209869867184	5.643260168035
C	-0.005889501742	1.201144032477	7.028748992874
C	-0.005341384991	-1.182951525243	7.035768987063
H	-0.008995809696	2.145559543250	7.563649090373
H	-0.008060168570	-2.124168320120	7.575744902042
C	-0.007337947228	0.009555008782	7.748913760391
C	-4.948856409053	-0.001310837404	-0.000706318081
C	-5.645909037824	1.216222489633	-0.003172166215
C	-5.648150631463	-1.214401564462	-0.001329406694
C	-7.035712981929	1.195303798960	-0.005855954099
C	-7.040684777823	-1.188706406594	-0.004116677125
H	-7.571399440103	2.139262329031	-0.009203927796
H	-7.579835561845	-2.130386610090	-0.006131418236
C	-7.754881926983	0.003146801819	-0.005803121353
C	0.001430284058	-0.003346388746	-4.943629275289
C	-0.000179745147	-1.216664676241	-5.642271676247
C	-0.001390417513	1.214114285063	-5.640613235042
C	-0.005052464179	-1.191261385841	-7.034817770222
C	-0.006166456634	1.192841849019	-7.030390746826
H	-0.007648975141	-2.133062859996	-7.573770077696
H	-0.009597216038	2.136674374438	-7.566322099956
C	-0.007591678204	0.000457693885	-7.749256765119
C	-4.918666044192	-2.520302668434	-0.000802262098
H	-5.611713830194	-3.359095186894	-0.000794147704
H	-4.271633268211	-2.621770704713	-0.874313517519
H	-4.272223008703	-2.621236809765	0.873190540101
C	-4.910290849223	2.518654280370	-0.004566370570
H	-4.263273854333	2.617620662470	0.869226010863
H	-4.262794760699	2.615631634657	-0.878240795806
H	-5.599410919488	3.360660252649	-0.005738557682
C	-9.249387658298	0.012668238529	0.003337987779
H	-9.651024804166	0.643889653448	-0.790224942828
H	-9.658613254747	-0.987932546883	-0.123080601204
H	-9.637562273989	0.410916876798	0.942803933373
C	-0.001237810453	0.009699875749	-9.243803536587
H	-0.800428491480	0.634744474436	-9.644048462525
H	0.934343357925	0.415033708633	-9.633948314397
H	-0.121116334331	-0.991904220180	-9.652545058111
C	0.000978692868	-2.522072022185	-4.911806791726
H	0.005305938894	-3.361471984892	-5.604119576660
H	0.872765643322	-2.620442032551	-4.261914692721
H	-0.874630019978	-2.625014335401	-4.267743699226
C	-0.001414549669	2.516383058352	-4.904603467728
H	0.002124417450	3.358687443706	-5.593364554817
H	-0.877081847640	2.615599707275	-4.260039488607
H	0.870275423419	2.612596218822	-4.254254560394
C	4.912703399077	-2.518028142072	0.000775063524
H	4.263988427535	-2.618309349732	0.873334261293
H	4.266757074796	-2.618876147131	-0.873762071891
H	5.604913018211	-3.357550019661	0.002122072316
C	4.901640078974	2.519625031837	0.002728117901
H	5.588980900173	3.363122015169	0.002767766690
H	4.253506139509	2.616342809298	-0.870650856131
H	4.253839529105	2.616110204753	0.876399236536
C	9.243727080680	0.016985222388	0.000889080248
H	9.634848634971	0.453728470931	-0.919901066486
H	9.642321852486	0.615467279229	0.821161533127
H	9.653204690624	-0.987575735592	0.089203569503
C	-0.000141353509	-2.516069577927	4.914213433276
H	-0.875908233689	-2.619507048605	4.270445092973
H	0.871487718925	-2.615342933871	4.264244184085
H	0.004141621062	-3.354720424176	5.607433745779
C	0.000044742774	0.020380538341	9.243445355692
H	-0.124627353963	-0.980162626659	9.653361039348
H	0.938098314044	0.421134108637	9.632433830716
H	-0.795490336804	0.650069800141	9.643634565991
C	-0.001341675025	2.522374973263	4.901517015261
H	0.870392910617	2.617803501278	4.251111046506
H	-0.876964189897	2.620979917611	4.256801888449
H	0.002258260820	3.365421858451	5.589369286913

118

^{1}B ≡ ^{1}[(**L1**)Ru(OH)(OOH)] E_{tot}(RM06L-D3(Dichloromethane)/def2TZVP/W06) = -2706.89752007 $\langle S^2 \rangle$ = 0.0000

Ru	-0.024957385884	-0.033511593141	-0.027293047087
O	-1.920936580052	-0.358663582134	0.090298087578

O	1.836852243190	0.251507553967	0.124089135169
C	0.559213142439	-3.449262655738	-0.013819201945
C	-0.000732294054	-0.029837890415	3.410231939703
C	-0.669970251827	3.371248611087	-0.022315598600
C	-0.002860847909	-0.032922682252	-3.452166077105
N	-0.293966503387	1.410252847534	1.423685675601
N	0.228128146522	-1.483441431419	1.428052118840
N	0.232484350104	-1.467565405201	-1.455351398733
N	-0.280721838422	1.397834658331	-1.461094650729
C	0.207621162778	-1.256030695308	-2.808009253609
C	0.456616337308	-2.809245161763	-1.249167235434
C	0.459524994138	-2.812990033563	1.229573398839
C	0.208403848930	-1.259074843161	2.775793728395
C	-0.236481453529	1.188240932013	-2.813438455049
C	-0.551212383957	2.735913448198	-1.258513175960
C	-0.546849298740	2.737719683890	1.223007773241
C	-0.239022323655	1.193995131737	2.770953653272
C	0.428380528197	-2.504424021126	-3.474804399377
C	0.577184190714	-3.456839532597	-2.519076388102
C	0.590205863216	-3.460019188503	2.502455702897
C	0.439868364824	-2.503666884802	3.452990375163
C	-0.468795780040	2.437631622256	3.444894130700
C	-0.658575526461	3.387850225975	2.492204054556
C	-0.479651985956	2.431951140062	-3.482706650318
C	-0.671080691256	3.379609404003	-2.530655609026
H	0.461484190546	-2.632198034312	-4.545485462468
H	0.757140897807	-4.511267885368	-2.657594098341
H	0.777439422644	-4.512817396282	2.643822874831
H	0.479066861782	-2.624245246862	4.524298271845
H	-0.483627401285	2.565188632739	4.515990317899
H	-0.856965361950	4.438505788433	2.633620136280
H	-0.502272416952	2.558932280871	-4.553763883424
H	-0.879089599002	4.428401106716	-2.672040578776
C	0.784536679581	-4.923487920618	-0.015719715614
C	-0.316056283528	-5.793218481566	-0.000166018747
C	2.087238033126	-5.435430676594	-0.030884026306
C	-0.090434704332	-7.164572395740	-0.001259022262
C	2.267969587176	-6.816607218864	-0.030799611447
H	-0.944616513893	-7.834192003981	0.010100053074
H	3.279051380795	-7.210388729153	-0.042342313493
C	1.195127107381	-7.699376566298	-0.016349215701
C	0.039736803399	-0.017260712284	4.901524631028
C	1.248337781424	0.252951923686	5.560864559293
C	-1.124059232247	-0.269422828221	5.636977730288
C	1.267472956441	0.266272323512	6.950584131287
C	-1.059722562845	-0.245964644000	7.028270287039
H	2.204155623535	0.475617934558	7.457564723212
H	-1.963957434030	-0.439872875155	7.596069908609
C	0.123897371804	0.019540761154	7.705772491091
C	-0.924308200700	4.840470490706	-0.023261520157
C	0.158982091641	5.731436792995	-0.011036212302
C	-2.236986801515	5.325791636708	-0.035012979707
C	-0.093506571403	7.097957628314	-0.011397959829
C	-2.444780951055	6.703180717822	-0.034450454981
H	0.747274698980	7.784314250137	-0.002224329194
H	-3.463427349436	7.076972599971	-0.043263473181
C	-1.389553793189	7.606984866780	-0.022851966015
C	0.023042420432	-0.029040474816	-4.943955819025
C	-1.147282790755	-0.289612666541	-5.666550359251
C	1.223440528793	0.241144608101	-5.618276688878
C	-1.097115551625	-0.275601121084	-7.058569293100
C	1.228723796715	0.244920195946	-7.008212288343
H	-2.006472544715	-0.476366572012	-7.615715727838
H	2.159549074207	0.453880711076	-7.526036536654
C	0.078547397840	-0.011102569185	-7.750064024131
C	-3.400232183389	4.385724913950	-0.049111380703
H	-4.344628671181	4.926170939230	-0.040467073189
H	-3.390097542381	3.745231367457	-0.933383489326
H	-3.389193940815	3.716721665846	0.813683515626
C	1.565624407687	5.222264851823	0.003703845386
H	1.764773413029	4.611313159816	0.887161449300
H	1.776483771045	4.593042139470	-0.864430577247
H	2.282660177177	6.040629398882	-0.000418406383
C	-1.630132601616	9.082166044034	-0.021148721477
H	-1.203836477955	9.554361467068	0.865524362517
H	-1.164017568868	9.563412884979	-0.882420610980
H	-2.692796848671	9.316262612265	-0.043573610031

```
C          0.115761206493    -0.001212510707    -9.244249389365
H         -0.865201728255    -0.199522136054    -9.672067713758
H          0.459468164395     0.960421204905    -9.628639882294
H          0.805652187101    -0.753315056871    -9.630758736524
C         -2.434985332474    -0.578843231799    -4.962314073974
H         -3.248744560403    -0.719550502524    -5.670883292848
H         -2.366589423604    -1.481000797887    -4.350896551260
H         -2.716098542369     0.229983159456    -4.285120228895
C          2.480552683552     0.521900153927    -4.857264002038
H          3.320331582463     0.685882610430    -5.529499466912
H          2.381388969849     1.407555656335    -4.225906469228
H          2.741327044777    -0.302147791763    -4.190211301454
C         -1.713074914682    -5.257859116876     0.017274399158
H         -1.897229715053    -4.639373866686     0.898469926790
H         -1.914790835843    -4.626647431840    -0.850763335300
H         -2.444439447859    -6.063475871142     0.019377009269
C          3.269518905108    -4.519359112997    -0.047545265708
H          4.202593830219    -5.079225313406    -0.045194809894
H          3.268595617382    -3.875702532834    -0.929572352881
H          3.276597797545    -3.853630019256     0.817845252588
C          1.406196823222    -9.179075807460    -0.015757720608
H          2.463971339636    -9.434311896091    -0.038721594035
H          0.970838048026    -9.643478596478     0.870628092879
H          0.930474657653    -9.650327411907    -0.877327060999
C         -2.418838689366    -0.560570273060     4.946404201343
H         -2.701895247983     0.241677077131     4.262080705862
H         -2.358908932534    -1.470023418560     4.344747153486
H         -3.227641721391    -0.690030669383     5.662754563225
C          0.176483545945     0.039889299774     9.199425248324
H          0.866904267018    -0.712629447756     9.584196137629
H          0.528464799399     1.002626224185     9.573411320196
H         -0.800913945181    -0.150911588884     9.638704834668
C          2.498265726063     0.522405021895     4.783997710847
H          2.753875313888    -0.312835545545     4.128707821678
H          2.392523990251     1.396253615872     4.137360953068
H          3.343939137838     0.698499381655     5.445707073185
H         -2.088246039292    -1.300925405218     0.246985568982
O          2.363898017812     1.571850913872     0.114939293636
H          1.635038229767     2.115353829508    -0.244262892821
```

117

[1]C E_{tot}(RM06L-D3(Dichloromethane)/def2TZVP/W06) = -2631.72910711 $\langle S^2 \rangle$ = 0.0000

```
Ru        -0.002404632934    -0.035737600981     0.001601797876
O          0.052605877835     0.256830133570    -1.899988869420
O         -0.025295086268     0.260065073636     1.903284718601
C         -3.459083904649     0.187097339394     0.008837692639
C          0.191045338397     3.410318147452    -0.001030564668
C          3.456719295795    -0.202068337058    -0.003240648142
C         -0.194493038553    -3.439736205288     0.002488252710
N          1.558386540085     1.351360975528     0.010487475727
N         -1.398831652917     1.518141285752    -0.008132140164
N         -1.523205289743    -1.361203600452     0.002467745475
N          1.359302440236    -1.523209909107     0.001663907475
C         -1.395446717906    -2.726893871580    -0.000240612296
C         -2.877879530861    -1.076532545080     0.003402362954
C         -2.750763243610     1.400055159413     0.010338571549
C         -1.092785895616     2.848774299716     0.002937433022
C          1.078589847045    -2.865902286736     0.005198781657
C          2.737601578746    -1.392726674388     0.002268394698
C          2.888487600493     1.082286247539    -0.007093775417
C          1.403822589562     2.708259118333    -0.003546236321
C         -2.700337630570    -3.316962953373    -0.001966849780
C         -3.606302054591    -2.307636310337     0.000120804819
C         -3.338913738290     2.707332395710     0.028781060729
C         -2.315626595766     3.600249632978     0.024157532520
C          2.703710437958     3.317272319433    -0.026137285764
C          3.619903120310     2.314860090073    -0.027977213179
C          2.308468600432    -3.598988920585     0.008592682432
C          3.322523233328    -2.698160098808     0.007242688668
H         -2.894247745177    -4.378236540898    -0.003393446825
H         -4.682143117250    -2.383793932559     0.000500072043
H         -4.397982690505     2.911200971820     0.044649641402
H         -2.378631748976     4.677170068257     0.036466562580
H          2.887993262947     4.380135325454    -0.040976253037
H          4.695272097349     2.397264446275    -0.043871723668
H          2.380815691200    -4.675409475423     0.010856851280
H          4.382773339702    -2.895873490900     0.008286957465
```

C	-4.948210287652	0.266205782071	0.009229419784
C	-5.642976005363	0.324419418920	-1.204693558030
C	-5.647019951312	0.291315651962	1.225330099342
C	-7.033237930936	0.406818018158	-1.182098191904
C	-7.034239367408	0.374873013171	1.201926869201
H	-7.569013752907	0.452072466533	-2.124659506163
H	-7.571560436792	0.395587983121	2.144810736260
C	-7.749142159991	0.433437377801	0.008411169911
C	0.275186382859	4.900128980950	-0.003019506100
C	0.348795343447	5.596207035606	1.209088209574
C	0.282361543207	5.596970483466	-1.220399386087
C	0.429011283586	6.986627581646	1.183006636424
C	0.365312434102	6.984269210274	-1.200403601405
H	0.485152210693	7.523941104050	2.124136802471
H	0.372795105878	7.520212283565	-2.144292439680
C	0.439191697145	7.700918380874	-0.008747819281
C	4.945349171058	-0.290166848712	-0.000254116584
C	5.639224782821	-0.306258306880	1.215547597862
C	5.645405651447	-0.351217873454	-1.214284471905
C	7.029691380021	-0.385535296749	1.196851934038
C	7.032931132777	-0.428762069875	-1.187012920880
H	7.564804884402	-0.399021703864	2.140780279336
H	7.571311476672	-0.475629069643	-2.128356187613
C	7.746837767762	-0.447348436282	0.008384010392
C	-0.278142065148	-4.929489748352	0.002478123428
C	-0.316926615466	-5.627609086346	-1.213917809975
C	-0.319745290713	-5.627158857959	1.215636665129
C	-0.397418139843	-7.015112397889	-1.192101419092
C	-0.400395506906	-7.017637624507	1.191693212092
H	-0.427724092073	-7.550903640461	-2.135625392206
H	-0.433186215317	-7.554931381384	2.133943165694
C	-0.440188976582	-7.732091356899	0.000761003062
C	4.913707452095	-0.335323716122	-2.518834509533
H	5.604400003001	-0.364291365892	-3.359108888159
H	4.238268586681	-1.188134679427	-2.612142881830
H	4.294786974502	0.557928196548	-2.624014094222
C	4.905065411881	-0.241725545362	2.517620920954
H	4.310880012690	0.670904614860	2.601125658097
H	4.208570809295	-1.075286441481	2.629917849472
H	5.592785093557	-0.267584273628	3.360428860915
C	9.239346351463	-0.527695716941	0.005103407468
H	9.641520313534	-0.572309966127	1.015493482489
H	9.590682569324	-1.408755576625	-0.534061201750
H	9.683574259870	0.336744449874	-0.491443302597
C	-0.523417162200	-9.224447223948	-0.007708751264
H	0.348190887126	-9.669315828870	-0.490981010411
H	-0.585710067974	-9.629101569368	1.000766053334
H	-1.396179289555	-9.572668680796	-0.562225169566
C	-0.274080222036	-4.894508933773	-2.517154228865
H	-0.307890993549	-5.583631767899	-3.358576303356
H	-1.112482877331	-4.202429220382	-2.618253352702
H	0.632550470272	-4.293870830492	-2.612894715680
C	-0.278911483622	-4.897836832032	2.521096613555
H	-0.326669280625	-5.588725859474	3.360413288323
H	0.634269673737	-4.308525464321	2.625488216058
H	-1.109607565920	-4.196061678228	2.618096150327
C	-4.910350834263	0.298337655318	-2.509005253472
H	-4.210829842169	1.132592676790	-2.596174853611
H	-4.319751497062	-0.613256253989	-2.621707273117
H	-5.598838408147	0.353885018352	-3.349753170776
C	-4.914481158582	0.228695485800	2.527997400906
H	-5.601584290467	0.283589526804	3.369924113572
H	-4.342127592008	-0.696184622074	2.623944261772
H	-4.196148573545	1.044896470772	2.627658408297
C	-9.241292386146	0.519886135876	0.015671519204
H	-9.588209369538	1.387408539351	0.579137998571
H	-9.644402370835	0.594018303221	-0.992611529892
H	-9.688712084660	-0.355871871544	0.488940887525
C	0.202644776698	4.862043137122	-2.521081959339
H	1.014448883032	4.139685989216	-2.627964287286
H	-0.726119191639	4.293724311573	-2.606181049260
H	0.251126011481	5.547093662984	-3.365097582544
C	0.523840096565	9.193195822668	-0.019283053200
H	-0.357811287373	9.639052086830	-0.483000301796
H	0.609235207297	9.597906224033	0.987471596356
H	1.384450016964	9.540284806705	-0.593186051948
C	0.343417106228	4.864377676506	2.514027051067

H	-0.545725049684	4.240329400568	2.624281362776
H	1.200915785165	4.193913832232	2.603390353761
H	0.373460516983	5.555100689668	3.354287075081
H	-0.842521176786	0.439357614316	-2.226365756299
H	0.884450755567	0.349869241117	2.228027025854

117

^{1}**D$_{w1}$** E_{tot}(RM06L-D3(Dichloromethane)/def2TZVP/W06) = -2631.70859622 $\langle S^2 \rangle$ = 0.0000

Ru	-0.005351809981	0.001914376260	0.369284230717
O	0.015429775932	0.011040838789	2.102866366486
O	-0.049824829736	-0.000406821395	-2.042448072219
C	3.439684037726	-0.008818896693	0.070199317328
C	-0.030472469000	3.449235732284	0.087897012403
C	-3.447085296326	0.016608734262	0.089239877199
C	0.022526371196	-3.441746814964	0.084762554729
N	-1.458569125020	1.453013756961	0.229508838237
N	1.415167409216	1.430209010194	0.031112291661
N	1.450408244053	-1.446717140693	0.214715191827
N	-1.422265182383	-1.421342472720	0.053722901592
C	1.252483413201	-2.803404404857	0.152781063240
C	2.806417932410	-1.241170939670	0.145380287937
C	2.778883377452	1.226279215767	0.016473648727
C	1.206914189728	2.793515123401	0.026534506639
C	-1.213526077245	-2.784058669653	0.032786372383
C	-2.785246579172	-1.217169112249	0.033633990104
C	-2.814011454226	1.248694436242	0.164058307119
C	-1.259604972534	2.809889620514	0.164088662552
C	2.526028238814	-3.469536208832	0.089090925725
C	3.478728597769	-2.511769957602	0.084307031836
C	3.430582361362	2.485308540041	-0.027013445284
C	2.463494499397	3.449641242452	-0.020608137962
C	-2.533429202290	3.476786628931	0.104610539179
C	-3.486193326813	2.519406824673	0.104251860897
C	-2.470088322148	-3.439907575167	-0.021512464281
C	-3.436925712365	-2.475837167489	-0.021419716001
H	2.656619876956	-4.538706104534	0.028795213585
H	4.548282754121	-2.637109943189	0.019563364876
H	4.500307928328	2.622906585325	-0.038305870707
H	2.597585950457	4.519830480095	-0.026827993028
H	-2.663655257534	4.545896507416	0.042901231612
H	-4.555924027283	2.644898753215	0.042865227695
H	-2.603886905708	-4.509977520850	-0.039094065799
H	-4.506599987396	-2.613103181179	-0.038107431044
C	4.930010686850	0.008555761455	0.017139384110
C	5.670726829957	-0.011646906435	1.205009552503
C	5.587308858968	0.044704228222	-1.221969096816
C	7.061817630831	0.008663574439	1.132903684531
C	6.976768269153	0.061369753551	-1.248660184122
H	7.632490238581	-0.003239402117	2.055836300504
H	7.480539517760	0.087503688630	-2.209755963833
C	7.735816526599	0.045080789264	-0.081354963813
C	-0.019974360604	4.939550455497	0.036645079085
C	0.015454830477	5.597274024413	-1.202314125585
C	-0.046943283860	5.679785257531	1.224597409950
C	0.024110808654	6.986771288998	-1.228757256892
C	-0.034272699325	7.070994583464	1.152789013255
H	0.049173198658	7.490929371590	-2.189675587634
H	-0.051376947178	7.641433207682	2.075788964561
C	0.000846276600	7.745421352443	-0.061275417225
C	-4.937254589772	-0.001063967866	0.033364149317
C	-5.591047840650	-0.033255865621	-1.207818927265
C	-5.680814795946	0.015378859238	1.219417184875
C	-6.980515559387	-0.049982801454	-1.237641240805
C	-7.071759222000	-0.004590849844	1.143900746896
H	-7.481957234929	-0.073312579812	-2.200052782873
H	-7.644756226585	0.004630360601	2.065442244349
C	-7.742554997073	-0.037339313087	-0.072229150230
C	0.010416731084	-4.932065769299	0.032238427917
C	0.029677818806	-5.672578060556	1.220168830211
C	-0.018959421856	-5.589207737024	-1.207196858384
C	0.015372931006	-7.063772207268	1.148122037194
C	-0.030158386070	-6.978768829829	-1.233670286718
H	0.026875471186	-7.634406049868	2.071099117705
H	-0.051511075525	-7.482709842268	-2.194812216864
C	-0.014602437842	-7.737779777859	-0.066312232038
C	-4.999772205782	0.056871745177	2.550635838999
H	-5.718275769573	-0.025521960842	3.363802166876

H	-4.273135290432	-0.750262251825	2.659718940354
H	-4.446347517274	0.987826132034	2.690229750886
C	-4.808645076936	-0.043796710635	-2.483121344442
H	-4.150039627208	0.824000254624	-2.558550998499
H	-4.166041815411	-0.923761145431	-2.556096618154
H	-5.466781521537	-0.039900219066	-3.349791979473
C	-9.235798580298	-0.058503168249	-0.136223854907
H	-9.623829926441	0.801102164971	-0.685223943899
H	-9.600441473093	-0.947022644593	-0.654315590753
H	-9.680787035263	-0.046808664880	0.857007659434
C	-0.030124094283	-9.231233751732	-0.126564462635
H	-0.013707944620	-9.673704806266	0.867712636297
H	-0.918872743842	-9.600544536135	-0.640960263710
H	0.829228841628	-9.617298126788	-0.677314472426
C	0.068451485800	-4.987899624533	2.549587603583
H	-0.009366402507	-5.704707175905	3.364685948994
H	0.996438641149	-4.428953963949	2.686950531794
H	-0.742590852336	-4.265430498512	2.657419945074
C	-0.031913020437	-4.810133996723	-2.484566686818
H	-0.032831728816	-5.470388491382	-3.349610419619
H	-0.910300225712	-4.165243529585	-2.556215941230
H	0.838363757311	-4.155201586921	-2.564841016998
C	4.986471551936	-0.057366165046	2.534408082360
H	4.256210528032	0.746600811787	2.642644433114
H	4.436540565078	-0.990772248250	2.671444724587
H	5.702602579415	0.026787991275	3.349460002935
C	4.808837695568	0.059366127229	-2.499833070563
H	5.469268361978	0.054627156929	-3.364703731695
H	4.148998689090	-0.807408648490	-2.578855992014
H	4.170797426978	0.942815811068	-2.573845258653
C	9.229186694421	0.065311606854	-0.141514891299
H	9.671483153425	0.060418665439	0.852949761261
H	9.618145139322	-0.798639661941	-0.683045795594
H	9.595645097995	0.949699894221	-0.665256652200
C	-0.092012153361	4.994897457483	2.553678768222
H	-1.024291011752	4.442817421966	2.689800283218
H	0.713516079423	4.266422567082	2.662162884307
H	-0.010211178698	5.710879905899	3.369107726238
C	0.012333393846	9.238898722345	-0.121091359920
H	-0.852749391365	9.622786321770	-0.664433162151
H	0.002601782859	9.680949341625	0.873447159230
H	0.895655237707	9.610756677391	-0.642824677733
C	0.038151039894	4.818801910979	-2.480111771967
H	0.930881690062	4.193763926660	-2.555813664863
H	-0.819203772532	4.146505543612	-2.557249314820
H	0.022540704011	5.478792683351	-3.345188558275
H	-0.030566107034	0.909600555433	-2.370558926510
H	0.752089362082	-0.407224891426	-2.399719881766

7.4.2 Further species related to anhydrous path

131
[1]E E_{tot}(RM06L-D3(Dichloromethane)/def2TZVP/W06) = -2940.19721806 $\langle S^2 \rangle$ = 0.0000

Ru	0.197337846224	-0.028147818727	0.191151706637
O	-0.026020988224	-0.210786865227	1.897818745092
C	3.638883856549	-0.142496395140	0.441780199927
C	0.298507919267	3.392924266361	-0.029236241147
C	-3.235994450746	0.033296122414	-0.211539608951
C	0.131937366355	-3.489000185018	0.043757435112
N	-1.199140310049	1.431906651839	-0.068275704321
N	1.679121482791	1.360234710525	0.222678889114
N	1.630086851130	-1.546904386409	0.298042841436
N	-1.284557678325	-1.464826505725	-0.094488102368
C	1.381934976003	-2.884799103261	0.213907931457
C	2.973564643669	-1.373522924305	0.398703844885
C	3.024758642472	1.108985967614	0.361197469918
C	1.511083223407	2.718034996871	0.146318251128
C	-1.092554591361	-2.818413065479	-0.081452263213
C	-2.623664963877	-1.228021285244	-0.179440412598
C	-2.555275638268	1.251236879159	-0.201782525817
C	-0.950069416302	2.777866449798	-0.144631242285
C	2.632707425843	-3.590837659114	0.289392910669
C	3.612214476624	-2.661410510343	0.405183120121
C	3.717460197728	2.358050901136	0.390560444141
C	2.790217575081	3.343580663073	0.260551894089
C	-2.190410181812	3.466216066500	-0.342735178389
C	-3.174949473583	2.532620819442	-0.364494459490
C	-2.368668615256	-3.459641837452	-0.186762270284
C	-3.311513748084	-2.481750518196	-0.236848765575
H	2.738860464677	-4.663689030768	0.251664692650
H	4.676183483089	-2.823794138786	0.476922901548
H	4.785307982610	2.465775558679	0.497380439864
H	2.957672287045	4.409037487269	0.240977079433
H	-2.289873245664	4.535248665082	-0.448015305634
H	-4.234073034065	2.689551338641	-0.496546130728
H	-2.522501194061	-4.527178991885	-0.204442306650
H	-4.381525495549	-2.596349680556	-0.309845799438
C	5.128076072081	-0.171233362296	0.521118712083
C	5.768800506139	-0.207423495327	1.764738284450
C	5.881943488181	-0.167804593284	-0.662659298509
C	7.160941777020	-0.238605862812	1.804359771297
C	7.268779739558	-0.200955841359	-0.576984379400
H	7.655066445103	-0.266793370197	2.770016365823
H	7.848679989968	-0.200658235692	-1.494545139472
C	7.930548613576	-0.235897431267	0.647724270023
C	0.346890544059	4.882686527752	-0.091117041691
C	0.678521214996	5.525687553121	-1.290170270503
C	0.069289725284	5.637364830567	1.059412084539
C	0.721341853952	6.917784025927	-1.319745892355
C	0.126407690876	7.024183775947	0.985535581613
H	0.974566213289	7.412044227655	-2.252035320761
H	-0.085141482738	7.603042319525	1.879060622105
C	0.449212954244	7.687044313483	-0.195483406155
C	-4.728121705822	0.070511363148	-0.222495080941
C	-5.443708186673	-0.025558041782	-1.421494977652
C	-5.413218388222	0.191941592426	0.997405593179
C	-6.835943255198	0.001010284665	-1.382045701303
C	-6.802785903630	0.214452290068	0.990936992698
H	-7.385546549468	-0.074530218571	-2.314681180421
H	-7.326798913380	0.304900332758	1.937152878380
C	-7.536479435422	0.120005336658	-0.188518766344
C	0.097844826725	-4.979447655974	-0.014714931475
C	0.049855291063	-5.725787512838	1.172282459277
C	0.112936483893	-5.628809869289	-1.255061530934
C	0.017071029480	-7.113135135911	1.093708575681
C	0.082610153967	-7.021013566601	-1.287379346409
H	-0.024031656541	-7.686859246917	2.014203777278
H	0.097929399521	-7.521353881331	-2.250174953302
C	0.034246921619	-7.783112533476	-0.126787527627
C	-4.665041801701	0.294828327666	2.288926096860
H	-5.345673701569	0.310047930570	3.137765571348
H	-3.974591461574	-0.539789694545	2.425443787218
H	-4.058825399535	1.202192741442	2.332908951623
C	-4.735497332423	-0.134431331921	-2.733847380441
H	-4.189922038522	0.783146358039	-2.966467488470
H	-4.000676353775	-0.941881430010	-2.738084943145
H	-5.435760967912	-0.313074243452	-3.547505193031
C	-9.030652058921	0.142967992726	-0.162656516710

```
H      -9.451051416290     0.076332313962    -1.164380916155
H      -9.431797864003    -0.686212477312     0.422561148296
H      -9.407705187023     1.057530726448     0.297785191270
C       0.008291809645    -9.276638991518    -0.179185053128
H      -0.841927455663    -9.681532609215     0.371467302543
H      -0.051133544286    -9.641796810980    -1.202803566205
H       0.903729046317    -9.706090828289     0.273889962290
C       0.032203362543    -5.044260800572     2.503774858848
H      -0.086819617707    -5.762587978097     3.312454742399
H       0.955658936821    -4.490390277209     2.685155520777
H      -0.779511160943    -4.317879693950     2.576006352171
C       0.153729481434    -4.847602192635    -2.530663931128
H       0.288019471148    -5.502299407381    -3.389531440379
H      -0.770378556529    -4.285864281050    -2.686176940908
H       0.963689423590    -4.115562937312    -2.533941548983
C       4.977198537105    -0.212753933820     3.033756077659
H       4.328998593945     0.662467168932     3.108426846284
H       4.322967299452    -1.084534794338     3.097356611423
H       5.628722576223    -0.221350614333     3.905293620506
C       5.208245991241    -0.131157505130    -1.998434209580
H       5.935007928823    -0.170822706133    -2.807373452382
H       4.517714604173    -0.967516408778    -2.126389702407
H       4.615130616776     0.776995634294    -2.127571017664
C       9.423750932246    -0.264303942997     0.708591835200
H       9.782507834822    -0.357679524556     1.731932074607
H       9.831793173963    -1.096441129844     0.133089824359
H       9.856484174922     0.645096560849     0.287641377913
C      -0.277768733947     4.967258927002     2.351204940915
H      -1.218378160982     4.416879643921     2.281901194835
H       0.481447787387     4.240639095815     2.646282003623
H      -0.378612748575     5.692883968360     3.155825412653
C       0.496503703043     9.180132181664    -0.244959570166
H      -0.479460596523     9.616952240582    -0.025991010556
H       1.188882499675     9.582667442470     0.495951336825
H       0.808315816164     9.540271742783    -1.223592213788
C       0.984828775502     4.738961040336    -2.524652284099
H       1.905433817923     4.160078789686    -2.418540259069
H       0.196635253906     4.018623063467    -2.751235993962
H       1.103211732383     5.390619028694    -3.388280826310
O       0.490685719994    -0.206073402769    -1.736091351216
H      -0.835805682234    -1.556707725415    -2.583102859226
C      -0.221905978108    -0.693209674342    -2.865139337207
H       0.498068592845    -1.030396927550    -3.617304654271
C      -1.033783292303     0.447241585392    -3.280682837756
H      -1.951321535249     0.613199899283    -2.727179029679
C      -0.637891425295     1.446005019183    -4.182480030403
H       1.332604517349     0.655592644121    -4.612978778194
C       0.619608250205     1.438369463776    -4.838594061499
H       1.919789630296     2.406475323382    -6.223056939020
C       0.951138859474     2.426212864287    -5.739415012544
C       0.053843247489     3.451799645915    -6.031177125265
H       0.321499128664     4.221820231657    -6.743013754064
C      -1.184551473700     3.488009069570    -5.392812093645
H      -1.880740186722     4.288496515486    -5.609598069249
C      -1.522212280568     2.516015826781    -4.478505690624
H      -2.482555663744     2.546733726368    -3.976657635424
```

131

[1]**F** E_{tot}(RM06L-D3(Dichloromethane)/def2TZVP/W06) = -2940.21836139 $\langle S^2 \rangle$ = 0.0000

```
Ru      0.135083882921    -0.023031527966     0.377157658281
O      -0.002281002343     0.028789425524     2.104054686473
C       3.585957213052    -0.127942500050     0.222868460194
C       0.210852372412     3.419324958399     0.043219293068
C      -3.279546339151     0.065983136270    -0.190125775517
C       0.089218602756    -3.473736427031     0.194208130067
N      -1.270195171130     1.457427556808     0.077654114978
N       1.600769169094     1.360032393977     0.089794364085
N       1.558241053622    -1.509918005535     0.358944298427
N      -1.291395862053    -1.419190921968    -0.034903814541
C       1.328207249462    -2.862272694166     0.316865982326
C       2.919731355394    -1.340636993289     0.319172896890
C       2.957966078423     1.120821122057     0.126907312379
C       1.429603493799     2.729099761648     0.058772171454
C      -1.120908761627    -2.788527625289     0.022189436082
C      -2.646003054698    -1.183905979633    -0.163188467050
C      -2.621655688621     1.283754625935    -0.099537750868
C      -1.034629687743     2.807423550201     0.007816324330
```

C	2.585644424421	-3.562117757579	0.313957601368
C	3.561524986807	-2.628781998045	0.308265545395
C	3.644350072558	2.361435284273	0.097259108662
C	2.704307668457	3.350953909515	0.065125080111
C	-2.279460355110	3.501948552443	-0.184902135312
C	-3.252724676126	2.567125701154	-0.253136965348
C	-2.386444735259	-3.414057050344	-0.106059688516
C	-3.323951547754	-2.427372516030	-0.224001537211
H	2.689838865272	-4.635343046059	0.282420195305
H	4.628767082109	-2.782198161835	0.274411443026
H	4.717150868453	2.469895371354	0.123248040109
H	2.866917049860	4.417089717100	0.058091872832
H	-2.375649207845	4.571806850957	-0.284367445303
H	-4.308304911377	2.715739515879	-0.418991450879
H	-2.548369085502	-4.480270937302	-0.089920567496
H	-4.392496289234	-2.539603663827	-0.318540038326
C	5.076198108568	-0.152463899256	0.181173818854
C	5.811749124122	-0.240160187899	1.369254568068
C	5.739067871646	-0.094745425900	-1.054733735193
C	7.202875423977	-0.267995976335	1.301066404454
C	7.128296908321	-0.130800392407	-1.077351746853
H	7.769480053609	-0.331711301812	2.224409930511
H	7.636067896634	-0.092617182627	-2.035963767388
C	7.882078800926	-0.215910724884	0.090221520812
C	0.256144616265	4.910114959377	0.064806988717
C	0.505288530247	5.637041037694	-1.105823346881
C	0.042769720425	5.584723542519	1.278128290320
C	0.533100728820	7.028486866632	-1.044793820966
C	0.083933937321	6.973990466590	1.294290410054
H	0.720057547016	7.586475212512	-1.956616645167
H	-0.077225139230	7.489251574920	2.235981238273
C	0.326383693837	7.718239988577	0.143045322194
C	-4.763470116366	0.078834641707	-0.341981927425
C	-5.339225595252	0.062611435399	-1.617767534975
C	-5.580528882627	0.104342159017	0.798361696072
C	-6.727088299692	0.069696723917	-1.734182971476
C	-6.961307625412	0.108773106136	0.637573623733
H	-7.168282013508	0.058662222501	-2.725540604755
H	-7.589346245364	0.125794626987	1.522704391247
C	-7.557556757392	0.091248981222	-0.620506758116
C	0.040022534365	-4.964267602069	0.210249523825
C	-0.003007383212	-5.645367913739	1.436436729337
C	0.038245679980	-5.682169455695	-0.991966884426
C	-0.051517933434	-7.034587541299	1.434445799485
C	-0.007177104191	-7.073658037843	-0.948318553447
H	-0.089335402773	-7.556570460820	2.385394254147
H	-0.004507717221	-7.626125104313	-1.882380123231
C	-0.053756996615	-7.770699481021	0.252675911047
C	-4.979408723086	0.127898533195	2.167797400147
H	-5.748341182698	0.089891312922	2.936832956286
H	-4.302401357230	-0.713563842102	2.326879298220
H	-4.388314033590	1.031133364289	2.332201795344
C	-4.481628767638	0.052883359711	-2.843163319175
H	-3.840796790764	0.936746237848	-2.885626643045
H	-3.816682253259	-0.813797022992	-2.868145363627
H	-5.084611725323	0.036230411180	-3.749025214071
C	-9.045644387167	0.096472798001	-0.760427508906
H	-9.351396393033	0.081939479427	-1.804908494748
H	-9.495459529776	-0.768049554804	-0.269427890580
H	-9.486804417299	0.980048434274	-0.296248480215
C	-0.100898991592	-9.264345322395	0.283365390776
H	-0.974417579745	-9.625136179056	0.828563481769
H	-0.135873402757	-9.686066273395	-0.719443203879
H	0.772704171345	-9.680253245343	0.788356606153
C	0.003735121151	-4.893185791609	2.729523539918
H	-0.133595770918	-5.563495606254	3.575715369765
H	0.944249932531	-4.358989831143	2.879227489431
H	-0.784927221791	-4.139359899288	2.764129942029
C	0.081937850169	-4.974394369710	-2.309492956120
H	0.192643028744	-5.678695965204	-3.131730983990
H	-0.831796214289	-4.402383836406	-2.487324580541
H	0.908772899991	-4.263337634189	-2.361106203451
C	5.123178630796	-0.307085981950	2.695589082053
H	4.388361563417	0.491255851893	2.811952658396
H	4.578573193592	-1.245592583939	2.819284918164
H	5.836594514004	-0.231115035015	3.513911438560
C	4.968771533672	0.008852353808	-2.333670636582

H	5.617887506043	-0.125255047787	-3.196930745783
H	4.170940869933	-0.734623731758	-2.389917986594
H	4.488322462310	0.985251258407	-2.432045220296
C	9.375457351871	-0.246468996504	0.034371730114
H	9.812639304819	-0.351957912915	1.025518157508
H	9.735371923996	-1.073602369720	-0.579487628431
H	9.776383287227	0.666228610069	-0.409999711342
C	-0.224827383870	4.828324840784	2.541215984909
H	-1.189859912928	4.318079970649	2.509729886755
H	0.524322586672	4.054406408194	2.717815708116
H	-0.232505138893	5.493236339713	3.402725558183
C	0.366711431750	9.211542500628	0.192487594045
H	-0.556560462207	9.623373031590	0.602760635386
H	1.175664936214	9.566690638826	0.833436267669
H	0.514488638711	9.642578690542	-0.796012554623
C	0.743967379972	4.942951606331	-2.408604404589
H	1.698059023340	4.411244329491	-2.409519848114
H	-0.023671319877	4.196205060166	-2.620724691502
H	0.762855341582	5.651353596427	-3.234758089090
O	0.428949109017	-0.102856377520	-1.999242136097
H	-0.652311377985	-1.849187577551	-2.560475226526
C	-0.044876099937	-1.041999642763	-2.959343919545
H	0.693490842604	-1.322137215104	-3.704641640842
C	-0.570413345197	0.322156875477	-2.940589311080
H	-1.553352139707	0.452889733012	-2.493597350772
C	-0.156106547803	1.371699908429	-3.892646344572
H	1.944389457737	1.008708549361	-3.667099364413
C	1.189473186388	1.597886329508	-4.174251373951
H	2.604656623687	2.754700520769	-5.286976775706
C	1.556138269343	2.580234596037	-5.079640243569
C	0.585071041102	3.344594950034	-5.713687638612
H	0.874344568178	4.112924728039	-6.419403547222
C	-0.755962222805	3.129213724959	-5.429995626361
H	-1.517023431155	3.729242045981	-5.912542993070
C	-1.124856425104	2.151662094915	-4.518130439498
H	-2.171110109576	1.987401286203	-4.286050978884

115

^{3}A E_{tot}(UM06L-D3(Dichloromethane)/def2TZVP/W06) = -2630.43291554 $\langle S^2 \rangle$ = 2.0310

Ru	-0.000000555186	0.000001398172	0.007775803165
O	-0.000016856050	0.000043097204	1.788999217352
O	0.000015645523	-0.000043773359	-1.773549214837
C	3.450702184116	0.004484611246	0.006804273344
C	-0.008855788054	3.444078259681	0.006229360150
C	-3.450702824339	-0.004482446769	0.006749151184
C	0.008855232977	-3.444075915001	0.006377004494
N	-1.468292764855	1.457285493867	0.007703401547
N	1.468776523649	1.468908665051	0.007538378507
N	1.468292286371	-1.457283397010	0.007798838118
N	-1.468777456319	-1.468906368244	0.007582314895
C	1.257700390453	-2.804947030236	0.006093073183
C	2.811011885124	-1.247280693954	0.006153013823
C	2.810956174560	1.263127773389	0.008370347233
C	1.252236908154	2.813644656661	0.007939757626
C	-1.252237560878	-2.813641933152	0.008042461191
C	-2.810957307191	-1.263125553209	0.008383681787
C	-2.811012671510	1.247282750209	0.006048199742
C	-1.257700842633	2.804948912327	0.005948781901
C	2.526141303388	-3.474435930788	0.002627872701
C	3.484942587142	-2.515030651488	0.002781846078
C	3.478648543029	2.527443960074	0.010376563170
C	2.511988153544	3.486965209801	0.010163811481
C	-2.526141643323	3.474437806191	0.002438359687
C	-3.484943010999	2.515032556316	0.002616850960
C	-2.511988806215	-3.486962801970	0.010273783042
C	-3.478649376441	-2.527441780717	0.010431378772
H	2.659430241863	-4.544591492137	0.000576155783
H	4.555219388150	-2.645867481312	0.000829396854
H	4.548210594844	2.663658763839	0.011657967165
H	2.641983215794	4.557507751958	0.011185319085
H	-2.659430561622	4.544593304503	0.000343124742
H	-4.555219733561	2.645869581138	0.000644298824
H	-2.641983687869	-4.557505342107	0.011336974528
H	-4.548211402745	-2.663656938243	0.011701132786
C	4.938804479386	0.005946847259	0.004543206665
C	5.635138081438	-0.006688317453	1.218914643695
C	5.631536916727	0.019025188071	-1.215005206978

C	7.027181924232	-0.004823235472	1.191183111128
C	7.020904887214	0.019748631261	-1.193478854870
H	7.566602340290	-0.015426093924	2.132574184708
H	7.555887407748	0.027909077605	-2.137726785750
C	7.740264889566	0.008976050134	-0.001384769502
C	-0.012094978139	4.932784724301	0.003415032356
C	0.007197501806	5.624460106062	-1.216636192509
C	-0.030105756460	5.628907255718	1.217617411011
C	0.008089416072	7.013723298452	-1.195317762713
C	-0.027405183258	7.021010814903	1.189035474186
H	0.023400474961	7.548222252876	-2.139740027259
H	-0.039136989733	7.560643082761	2.130258780356
C	-0.008732873592	7.733619313136	-0.003580442650
C	-4.938805282250	-0.005945185492	0.004469194319
C	-5.631523006572	-0.019027538904	-1.215087166983
C	-5.635153392204	0.006691106472	1.218832493235
C	-7.020891459235	-0.019754503670	-1.193577333025
C	-7.027196711033	0.004824813816	1.191084441919
H	-7.555862532447	-0.027919897237	-2.137831674461
H	-7.566628437692	0.015431890859	2.132469020692
C	-7.740265615723	-0.008979528177	-0.001492045587
C	0.012094038647	-4.932782438515	0.003614790315
C	0.030073376284	-5.628864837915	1.217841131964
C	-0.007177337578	-5.624498209209	-1.216413359309
C	0.027364818096	-7.020968773061	1.189305463391
C	-0.008078384863	-7.013761252775	-1.195048757499
H	0.039065720475	-7.560569947560	2.130546999636
H	-0.023379175538	-7.548291171445	-2.139453570826
C	0.008716847487	-7.733617367571	-0.003287496068
C	-4.901625703766	0.025292909088	2.522202062110
H	-5.591308279147	0.013029063272	3.363496959024
H	-4.235371807655	-0.833852685805	2.623143349303
H	-4.273708897568	0.913472992984	2.618653724541
C	-4.890486492093	-0.028651628169	-2.514280104933
H	-4.244396950162	0.845657441179	-2.616638995919
H	-4.240871537418	-0.901970691866	-2.601766597567
H	-5.575594428024	-0.037386529929	-3.359331415580
C	-9.234558572768	-0.023927962101	-0.011720435209
H	-9.638584171218	0.754455654927	-0.659943073117
H	-9.618269047505	-0.973256031109	-0.390403517955
H	-9.644958516615	0.121481877670	0.985758100410
C	0.013559341602	-9.227933939882	-0.014804539966
H	-0.055539002223	-9.637847651287	0.990988989296
H	-0.818098579040	-9.625678248448	-0.597858050270
H	0.925772319402	-9.618228680046	-0.469698636086
C	0.052605130308	-4.896550826608	2.521853684826
H	0.035636494482	-5.587107609443	3.362306254679
H	0.945112312127	-4.275022838283	2.619345419524
H	-0.802094912952	-4.224770461677	2.623326684238
C	-0.025817021474	-4.883615770759	-2.515615162550
H	-0.032919285224	-5.569061769979	-3.360360830892
H	-0.903752166551	-4.239949787209	-2.600573855327
H	0.843980537920	-4.232035042858	-2.620933590661
C	4.901595178479	-0.025289655012	2.522275656257
H	4.235292196827	0.833821495805	2.623183545111
H	4.273726871230	-0.913502294037	2.618745824018
H	5.591266794353	-0.012964675667	3.363578664106
C	4.890515640631	0.028644514460	-2.514206855120
H	5.575633417998	0.037354896152	-3.359250450829
H	4.244410677181	-0.845653798844	-2.616560330441
H	4.240918239845	0.901974639253	-2.601713767261
C	9.234558012140	0.023919703596	-0.011596036423
H	9.644945849984	-0.121465480729	0.985891054434
H	9.638588285979	-0.754483152278	-0.659792829688
H	9.618276274153	0.973235918908	-0.390300670440
C	-0.052669747962	4.896636913561	2.521653922395
H	-0.945187095263	4.275124204163	2.619150994765
H	0.802019435218	4.224848386424	2.623164124985
H	-0.035706896199	5.587221321413	3.362083932084
C	-0.013531211738	9.227935664191	-0.015151972388
H	-0.925455262729	9.618246250004	-0.470606108154
H	0.055003381634	9.637881935425	0.990666338151
H	0.818490769974	9.625628938196	-0.597725477082
C	0.025864922478	4.883534109697	-2.515812741726
H	0.903811801431	4.239879628087	-2.600737800628
H	-0.843920550723	4.231935679641	-2.621120749186
H	0.032967837640	5.568951896695	-3.360581302514

118

^{3}B E_{tot}(UM06L-D3(Dichloromethane)/def2TZVP/W06) = -2706.89480671 $\langle S^2 \rangle$ = 2.0135

Ru	0.011863435300	-0.000155711102	0.056642472989
O	-0.006233403591	0.043268710409	1.998842878249
O	0.038400167605	-0.104720840582	-1.916462655587
C	3.457422805295	0.009994356793	0.076503283475
C	0.002673046941	3.447707284456	-0.043032499782
C	-3.444729614917	-0.004032624266	0.081764209345
C	0.013631516619	-3.442213951767	-0.010765015932
N	-1.448678369519	1.449469025966	-0.018881174042
N	1.456434555655	1.452420548109	0.039791213953
N	1.463910261862	-1.442714173923	0.027757842906
N	-1.442974436749	-1.451538050195	0.067954156145
C	1.253343927864	-2.796874262607	0.005608808621
C	2.815147212729	-1.231973428794	0.055261256263
C	2.809757499854	1.247652574542	0.056408201904
C	1.242839446976	2.804963367809	-0.011793574994
C	-1.229969485032	-2.802796368410	0.019054407372
C	-2.796046016044	-1.243848694626	0.068028142894
C	-2.805698986974	1.239168324149	0.053680320309
C	-1.239150486183	2.804915048319	-0.020969369945
C	2.521419628012	-3.460297336245	0.013977130931
C	3.480381905685	-2.499588142335	0.048652106329
C	3.471395649868	2.517810469508	0.022228726990
C	2.509556924092	3.473509914127	-0.025017008577
C	-2.504698439342	3.466857549507	0.029840521553
C	-3.465751056435	2.506649704156	0.078313627659
C	-2.496260451420	-3.470350776199	-0.000325275806
C	-3.458333728048	-2.512765910901	0.032308701753
H	2.656877447148	-4.530262396814	-0.000336094025
H	4.550578302714	-2.633318505423	0.066445919673
H	4.541291674372	2.655023035339	0.025410189625
H	2.640577105309	4.543389824773	-0.064437455406
H	-2.639926050778	4.536946417397	0.037493660222
H	-4.534416690524	2.643288330741	0.130937621228
H	-2.628373703641	-4.540168094041	-0.038117856589
H	-4.528298410055	-2.648976185690	0.025129785632
C	4.948664313826	0.012776629961	0.108918239856
C	5.618891995812	0.028634029184	1.341328383405
C	5.674273901466	-0.000120902052	-1.088274552270
C	7.008789771299	0.030978429181	1.351012120317
C	7.066071056329	0.003359210069	-1.032243317228
H	7.523927942097	0.043043204459	2.306317398769
H	7.625836764754	-0.005889558399	-1.961833010345
C	7.753950599340	0.018652863326	0.174852680364
C	-0.000713292088	4.938089013239	-0.098029150381
C	-0.103607530438	5.586307181166	-1.338195467831
C	0.095598776984	5.684772222165	1.082138813542
C	-0.104786094353	6.975684998066	-1.372875194398
C	0.087784205761	7.075340138077	1.001306747306
H	-0.181596505063	7.473541757099	-2.334270489798
H	0.159281877859	7.651753855582	1.917919633242
C	-0.010323880477	7.741647115773	-0.213901076078
C	-4.935750436686	-0.005908445348	0.110006640364
C	-5.656022780216	0.060500645034	-1.092076696703
C	-5.611172287742	-0.069122971739	1.334510753239
C	-7.044999235959	0.062102799169	-1.044614992758
C	-7.003991232948	-0.062962916737	1.336126377226
H	-7.598940500012	0.112394661349	-1.976650138746
H	-7.525159818475	-0.108213378019	2.286790107962
C	-7.740918101232	0.001454349583	0.159951075703
C	0.015948901747	-4.932764790611	-0.063379087306
C	0.003504084528	-5.675096688920	1.123194778689
C	0.028589516601	-5.584895034056	-1.305513543514
C	0.005131785741	-7.065901437983	1.047782641328
C	0.028507234134	-6.974507571579	-1.334635058228
H	-0.003407306316	-7.638904351976	1.969266835615
H	0.037441429735	-7.476168957254	-2.297078541446
C	0.017392553700	-7.736337328197	-0.169142793664
C	-4.855084842349	-0.141121374298	2.623427100316
H	-5.528938409386	-0.127308188456	3.477679683279
H	-4.256089236855	-1.052096631167	2.688021302743
H	-4.158569479136	0.692670388344	2.731484086976
C	-4.944446143602	0.125045765598	-2.406603699087
H	-4.318425114160	1.017284083673	-2.483984035031
H	-4.281744238495	-0.731008790591	-2.548835183862
H	-5.648419499566	0.143624030769	-3.236054817397

```
C        -9.235390841631   -0.001708931494    0.178971836579
H        -9.644398161991    0.847972106115   -0.369335100207
H        -9.636697591744   -0.899024320339   -0.295732182223
H        -9.624440207006    0.036056192140    1.194737550031
C         0.019479424466   -9.229611354782   -0.233666709708
H         0.001517927892   -9.674912079673    0.759306046630
H        -0.844260940994   -9.606227129151   -0.783948308504
H         0.903906153538   -9.604886242732   -0.751166994678
C        -0.011754075910   -4.992006736617    2.454153285777
H         0.002387938537   -5.713486285734    3.268572172533
H         0.848221459238   -4.330727483686    2.578631565239
H        -0.900451474714   -4.369190523640    2.577291420769
C         0.042094631379   -4.800805583965   -2.579513813590
H         0.028296043155   -5.457485628394   -3.447035397093
H        -0.817694467427   -4.131353077240   -2.651977336416
H         0.928626462700   -4.167625561225   -2.654038479050
C         4.853632738033    0.042786438846    2.626723007763
H         4.209154872599    0.920844790279    2.703323179153
H         4.198112076927   -0.825874244700    2.715922408329
H         5.523197886768    0.045046256948    3.484465957975
C         4.972730586693   -0.016157830692   -2.409353483815
H         5.683199447559   -0.014896115594   -3.233520874405
H         4.336713517254   -0.896959412511   -2.518239368479
H         4.319068000733    0.850043568307   -2.529819284701
C         9.248005209802    0.021486427793    0.218042768448
H         9.633103994625   -0.850390539031    0.749462070870
H         9.679004637539    0.018574876988   -0.781363414508
H         9.630150490954    0.898112521213    0.743606683651
C         0.206316567224    5.008115210451    2.411664196604
H        -0.600367886587    4.289936648913    2.569461615639
H         1.138465194640    4.446140268629    2.501266606371
H         0.177268835765    5.730371138120    3.225075433562
C        -0.015185523289    9.234685330427   -0.283505656095
H        -0.924938096926    9.609133263043   -0.755565585437
H         0.055741793441    9.683443304155    0.705532384387
H         0.819098476231    9.608459587428   -0.879436162936
C        -0.211478365467    4.798642695790   -2.605748107019
H         0.613578217751    4.092036978347   -2.715285619595
H        -1.128786581909    4.205988284121   -2.635400437866
H        -0.212137677894    5.451024724951   -3.476659114583
H        -0.351178265664   -0.787488627441    2.355524745756
O        -1.185866420590    0.042006087977   -2.615908683526
H        -1.682927941506    0.679698502997   -2.069850190135
```

117

^{3}C E_{tot}(UM06L-D3(Dichloromethane)/def2TZVP/W06) = -2631.73787386 $\langle S^2 \rangle$ = 2.0139

```
Ru       -0.008052386982   -0.000240468617   -0.001137898794
O        -0.000081090302    0.038631504688   -1.952002937133
O        -0.002143573844   -0.037247141005    1.949818339772
C        -3.443807325945   -0.228432860282   -0.007505968712
C        -0.231011632470    3.439500331262   -0.001351949554
C         3.439258990320    0.228765173362    0.003593273180
C         0.225825583793   -3.439281127947    0.000924387192
N         1.350892037040    1.545826175262    0.049241150849
N        -1.547106334637    1.349853375510   -0.040399728655
N        -1.355138704181   -1.542159501794    0.033408871862
N         1.543563767140   -1.352962610033   -0.046888289357
C        -1.053751928094   -2.877754990028    0.029580968794
C        -2.718264864832   -1.422726586990    0.026580306095
C        -2.882365233106    1.051183284418   -0.038847388326
C        -1.425103702001    2.713726335506   -0.034909388341
C         1.422567848146   -2.715947997699   -0.029945252553
C         2.879111312897   -1.052290143386   -0.027926146274
C         2.714669990145    1.424664725961    0.033840001147
C         1.050631720459    2.880698546568    0.032711661999
C        -2.274411532044   -3.625971138495    0.037364248139
C        -3.296620102334   -2.732466998831    0.035691366485
C        -3.629092551593    2.272578151060   -0.049595367036
C        -2.734277906564    3.293626504253   -0.047028889991
C         2.271251041233    3.628431803295    0.029339217352
C         3.293450302227    2.734234646696    0.029653980705
C         2.731712593365   -3.295233232316   -0.022488683749
C         3.626344809472   -2.273406721775   -0.021165669120
H        -2.336596536171   -4.702847478374    0.037740146253
H        -4.355413262344   -2.938630690919    0.034255382162
H        -4.705905819307    2.335979237475   -0.052170265414
H        -2.939202997686    4.352651144062   -0.047073860320
```

H	2.333899116588	4.705242827095	0.018432877283
H	4.352256286809	2.940147007413	0.019299757349
H	2.937236451896	-4.354088087628	-0.010373618433
H	4.703112744677	-2.336461058316	-0.007966160444
C	-4.932248605840	-0.327319271539	-0.009469052970
C	-5.627663621926	-0.369961329751	-1.223796217191
C	-5.630230397617	-0.379287450310	1.206452727321
C	-7.017195284026	-0.465627022978	-1.201318439286
C	-7.016791192053	-0.473630850593	1.182848097601
H	-7.553412459593	-0.500205495868	-2.144108478435
H	-7.553265823071	-0.513162579203	2.125637225723
C	-7.732031699905	-0.518473747032	-0.011065755330
C	-0.330603510345	4.927931191695	-0.002180166152
C	-0.370173278747	5.625106978161	1.211214343597
C	-0.385693125540	5.624255539573	-1.218874740618
C	-0.466395476689	7.014519152677	1.187414063275
C	-0.480227094925	7.010825579816	-1.196743745197
H	-0.499144068788	7.551923060772	2.129584069431
H	-0.522180971256	7.546076806457	-2.140110261232
C	-0.522476655757	7.727693398194	-0.003692238481
C	4.927607837078	0.327359740111	0.005401998952
C	5.621799701769	0.397232563180	1.222475702807
C	5.627076567776	0.351461724276	-1.207076894200
C	7.008457301438	0.490093979146	1.202061197412
C	7.016459183122	0.447143629125	-1.181790303552
H	7.541969666770	0.542673261701	2.145864135433
H	7.555704165390	0.468202018330	-2.123226994218
C	7.727519262113	0.517027252030	0.009886030105
C	0.324019541147	-4.927789216272	0.002400136579
C	0.386160155266	-5.623664431505	-1.210785753133
C	0.356579457429	-5.625367148889	1.219182244040
C	0.480000744372	-7.013229683663	-1.186758413591
C	0.452212936758	-7.011868838772	1.197297852942
H	0.526721395615	-7.549877595474	-2.128772862361
H	0.478965930471	-7.547983643019	2.140725236679
C	0.514713269644	-7.727598207592	0.004457500575
C	4.900403072175	0.276599853016	-2.512718742357
H	5.588514041915	0.353280262128	-3.352132992944
H	4.354201699312	-0.663499991642	-2.616524286373
H	4.161617029309	1.074550298496	-2.611130218189
C	4.885057865334	0.372803101539	2.524397012513
H	4.213230550426	1.228199433439	2.623211295784
H	4.263208180862	-0.519547351089	2.620157812500
H	5.572597885536	0.392976008640	3.367502395424
C	9.218907219301	0.615265744685	0.019999106862
H	9.557023488696	1.502652439328	0.557268174814
H	9.671065282186	-0.241726510386	0.522224625763
H	9.625393737834	0.660784219283	-0.988611963319
C	0.613769803854	-9.218967880500	0.013164423807
H	0.682142527322	-9.622998712643	-0.995161737886
H	1.489502104444	-9.557623358651	0.568989102824
H	-0.253593672858	-9.673134418785	0.495311949976
C	0.353866073187	-4.892880100305	-2.515925228795
H	0.371866114204	-5.584241117687	-3.355988267013
H	-0.540334893498	-4.273622040757	-2.610693328598
H	1.207010281497	-4.219292964989	-2.621868796629
C	0.290411269675	-4.892461724253	2.521559154665
H	0.360031967747	-5.577959335099	3.363730726472
H	1.096316987545	-4.161890595750	2.615799225856
H	-0.642088271541	-4.333465288556	2.622821505550
C	-4.896783260469	-0.314089843117	-2.527861454553
H	-4.338015600856	0.617599877819	-2.637539874183
H	-4.166550125512	-1.120792437082	-2.617374910908
H	-5.583670139690	-0.390170477539	-3.368376729558
C	-4.897567310500	-0.334770586974	2.509933666122
H	-5.587273186700	-0.358677629025	3.351209836254
H	-4.211845616651	-1.177831041348	2.616421452630
H	-4.288936944411	0.567110641320	2.600462094476
C	-9.223393679528	-0.618204774250	-0.004343137624
H	-9.627001057216	-0.672277585882	-1.013710872724
H	-9.562032039268	-1.501876872474	0.538786577572
H	-9.678075867651	0.242053279761	0.489935103148
C	-0.343596212785	4.889769207728	-2.521381129781
H	0.558301881170	4.281240305006	-2.612712131825
H	-1.186593854641	4.203593931052	-2.625253207824
H	-0.369296706302	5.578258252676	-3.363584717729
C	-0.623046378805	9.218964243032	-0.012149233956

H	0.235341103754	9.673442150631	-0.509834232309
H	-1.508647901778	9.556281511623	-0.552931151021
H	-0.674185861169	9.623918842399	0.996826280399
C	-0.311033868522	4.896247517340	2.516464487347
H	-1.115277386906	4.163418224217	2.607631298137
H	0.623821775628	4.342540221939	2.626989112268
H	-0.389381400620	5.583919752920	3.356104585581
H	0.713481584879	-0.513075662779	-2.301199362292
H	0.632957748234	0.602174679315	2.300963980707

117

^{3}D$_{w1}$ E_{tot}(UM06L-D3(Dichloromethane)/def2TZVP/W06) = -2631.73589494 $\langle S^2 \rangle$ = 2.0126

Ru	-0.005440700115	0.004461823183	0.246915381135
O	0.002491244357	0.012084382604	1.995630754896
O	-0.032790509595	-0.020604411536	-2.124919150867
C	3.441598202770	0.012681234691	0.025679788874
C	-0.011338756403	3.453839719650	0.041461123214
C	-3.449173968750	-0.003107348751	0.033694781776
C	0.002507555753	-3.443288668245	0.050665157128
N	-1.456222997443	1.452374687549	0.058640441473
N	1.442820729569	1.460273970230	0.061387304702
N	1.447761615596	-1.443091264505	0.059405282466
N	-1.451277693594	-1.449894326790	0.080878126076
C	1.242521701299	-2.797723372978	0.045284903694
C	2.801513478912	-1.230456506713	0.033332259186
C	2.796318479327	1.253246973462	0.030047376140
C	1.231745018073	2.813429599085	0.040097274929
C	-1.239695167528	-2.801750188256	0.053842503310
C	-2.803519848614	-1.242996653821	0.044569289102
C	-2.809002000888	1.239706737179	0.032723662322
C	-1.250982392639	2.807022910132	0.038863134900
C	2.512343285085	-3.459475263994	0.007976601303
C	3.469688023543	-2.497145711555	0.000008870657
C	3.459824326826	2.522618891519	-0.011427100300
C	2.498798707086	3.480991622186	-0.004202988356
C	-2.521097287357	3.468674061062	-0.001744163376
C	-3.477796420978	2.506040747105	-0.006239891212
C	-2.506787519604	-3.470291691854	0.005551095653
C	-3.467580083625	-2.512553469177	-0.000391690610
H	2.649445205189	-4.529305295675	-0.013735725097
H	4.540028409258	-2.628828072045	-0.029003723670
H	4.529505941147	2.658408887668	-0.045208091558
H	2.631175875701	4.551301170795	-0.031590831080
H	-2.658324857178	4.538361118484	-0.028386459712
H	-4.548166688349	2.637015164472	-0.036936774055
H	-2.638289389161	-4.540525567752	-0.027090803433
H	-4.537140525749	-2.647829813437	-0.038682429923
C	4.933245161142	0.015416763101	-0.007028732792
C	5.657997602391	0.027768294586	1.194066409991
C	5.606704445930	0.003064725142	-1.234406982802
C	7.047148988008	0.027970024876	1.142450177225
C	6.999525185133	0.004130178476	-1.240781709218
H	7.603806308603	0.035748250781	2.074255981824
H	7.517788273397	-0.006606175119	-2.194111833495
C	7.740068568444	0.016938126072	-0.065125439708
C	-0.014988663881	4.945781264657	0.028409498557
C	-0.020494908817	5.637129897461	-1.188990491300
C	-0.010329396311	5.653022472893	1.240081683193
C	-0.021468042731	7.029896707369	-1.175034194824
C	-0.011698231650	7.042783920500	1.208730332146
H	-0.024219996806	7.561961905201	-2.120823420010
H	-0.006899319790	7.585757864053	2.148592659819
C	-0.017770715764	7.753236375333	0.011370852848
C	-4.940857168221	-0.006258845885	0.003802047616
C	-5.616769061492	0.005370628250	-1.222285401919
C	-5.662765523451	-0.018733308304	1.206602201510
C	-7.009675775892	0.003337657644	-1.225059645642
C	-7.052054167260	-0.019571477896	1.158314593869
H	-7.530186904260	0.013029862083	-2.177208612694
H	-7.606551029380	-0.027276333958	2.091428042567
C	-7.747604209595	-0.009478406273	-0.047743344696
C	0.004921357194	-4.935086638227	0.030600949461
C	-0.018523543855	-5.649275052837	1.237927809377
C	0.027810882092	-5.619048457950	-1.190801648338
C	-0.018713860598	-7.038784950235	1.198568176404
C	0.025620958234	-7.011966133137	-1.184732851216
H	-0.037350943813	-7.587258701848	2.135086397237

H	0.040854880865	-7.538609531816	-2.133432512326
C	0.002865298069	-7.742194389084	-0.002839008143
C	-4.952334687362	-0.029639817152	2.522954134863
H	-5.657118717402	-0.024176736007	3.352014239886
H	-4.316364706274	-0.910602572977	2.630430272566
H	-4.296207195355	0.835794635004	2.634749340628
C	-4.860881948292	0.022457022376	-2.513427703870
H	-4.232114525699	0.911129720595	-2.600558123017
H	-4.192344876714	-0.836310120393	-2.601832337296
H	-5.536390214083	0.008730161773	-3.366509178617
C	-9.242148176735	-0.023237271698	-0.068149510043
H	-9.631845304762	0.111233497300	-1.075538625728
H	-9.635633747155	-0.967377180479	0.313580110742
H	-9.659085456758	0.763356731449	0.561956968019
C	0.008605148079	-9.236879399941	-0.012951357202
H	-0.823566226046	-9.644274619396	0.562801390114
H	-0.058960562977	-9.632281431284	-1.024781471000
H	0.920397679054	-9.634070255980	0.437030045753
C	-0.044606684307	-4.930272433116	2.549393034230
H	-0.038447655741	-5.629457012459	3.383165204835
H	0.813993820291	-4.265479599648	2.662399957194
H	-0.932055326759	-4.301730343633	2.646872001157
C	0.054936511129	-4.871936322772	-2.486959789505
H	0.034214678947	-5.552750141471	-3.335648515778
H	-0.795504811688	-4.193609824987	-2.580970555068
H	0.952325236847	-4.255874002932	-2.577375100986
C	4.950492010570	0.038770316778	2.511919552074
H	4.308860303513	0.915784782345	2.617725784416
H	4.300428677928	-0.830669117214	2.628046589140
H	5.657119930546	0.040561955020	3.339395794233
C	4.848520812610	-0.013621770805	-2.524465242340
H	5.522391452803	-0.009486789919	-3.378899978980
H	4.212873464232	-0.898083696766	-2.607001374607
H	4.188942595294	0.852019045786	-2.616082820776
C	9.234531082647	0.029269703349	-0.088931441786
H	9.652002008932	-0.759047114061	0.538645843357
H	9.621794666656	-0.103647192081	-1.097451764419
H	9.629809616602	0.972188300880	0.293903740789
C	-0.002633300778	4.926506114429	2.547643207817
H	-0.871851200338	4.274086581701	2.651659525474
H	0.874541324108	4.284055473271	2.647084272304
H	-0.004222894063	5.621110244122	3.385232268013
C	-0.031226355385	9.247871030106	0.009646338716
H	-0.970456408482	9.636695402462	0.407883623258
H	0.763232670298	9.656762552854	0.635099699581
H	0.090863424573	9.649947257729	-0.994446109580
C	-0.023788169777	4.897964026521	-2.490015954269
H	0.850565691512	4.250818282877	-2.588215239215
H	-0.899886117448	4.252746318327	-2.584841661932
H	-0.024752589465	5.584243242565	-3.334499752664
H	0.013288490859	0.878268296651	-2.480272038279
H	0.756436465963	-0.463520760973	-2.467915778408

114

^{3}D E_{tot}(UM06L-D3(Dichloromethane)/def2TZVP/W06) = -2555.27010532 $\langle S^2 \rangle$ = 2.0125

Ru	-0.000018840967	0.000007566501	0.111722590582
O	-0.000161843286	0.000065213307	1.851215697439
C	0.024667033342	3.447366696111	-0.102223919428
C	-3.447368989235	0.024742977251	-0.102781528493
C	-0.024661326688	-3.447366836599	-0.102094002683
C	3.447373531363	-0.024743774003	-0.102450100145
N	-1.458118910101	-1.437270802800	-0.098036542429
N	-1.437342647623	1.458186941585	-0.098126080059
N	1.458122490941	1.437276371921	-0.097892629651
N	1.437352991988	-1.458195420410	-0.097796765962
C	2.812261729087	1.219892768345	-0.111980223671
C	1.260119535992	2.794519232006	-0.111767867996
C	-1.219975356313	2.812338983530	-0.112218216446
C	-2.794597584175	1.260201930548	-0.112440350870
C	2.794606313506	-1.260206489870	-0.112050282329
C	1.219985982015	-2.812344494256	-0.111916241564
C	-1.260110908776	-2.794516175960	-0.111818204361
C	-2.812257311998	-1.219893358222	-0.112233039473
C	3.485538558535	2.482913735046	-0.142267525701
C	2.532739309432	3.449559634362	-0.142061365178
C	-2.482964120042	3.485611857454	-0.143070787177
C	-3.449619700277	2.532801423662	-0.143185037022

C	-3.485529201458	-2.482916240972	-0.142513429567
C	-2.532727259191	-3.449559481570	-0.142194701455
C	3.449632292258	-2.532806848489	-0.142728954285
C	2.482977617224	-3.485618337085	-0.142663422488
H	4.556576706179	2.609406948925	-0.166250488643
H	2.674829631945	4.518639194951	-0.165875441426
H	-2.609383015154	4.556648193241	-0.167217255450
H	-4.518689684253	2.674830540687	-0.167456439417
H	-4.556565301836	-2.609412846400	-0.166571002353
H	-2.674813480550	-4.518640349735	-0.165974131836
H	4.518703844619	-2.674835635974	-0.166931793735
H	2.609398858977	-4.556654883673	-0.166788635919
C	0.035396977394	4.939414441765	-0.080707592824
C	0.038774414208	5.614800364447	1.146082445200
C	0.044124379869	5.661908270219	-1.282954765736
C	0.051077115967	7.007677396492	1.148624338589
C	0.056211665442	7.051015432943	-1.234594424161
H	0.055010465952	7.527703119335	2.101014877604
H	0.064031935070	7.605576675082	-2.167670473287
C	0.059546076410	7.746122422630	-0.028204634472
C	-4.939431493698	0.034558520589	-0.081791710550
C	-5.661476760245	0.037716050322	-1.284255799424
C	-5.615324523766	0.036421198167	1.144797807010
C	-7.050711357281	0.042934290323	-1.236411790868
C	-7.008178576934	0.041656907703	1.146827698846
H	-7.604929693954	0.043620088371	-2.169709450930
H	-7.528559535142	0.041284458470	2.099038651349
C	-7.746250671546	0.045398368126	-0.030346233518
C	-0.035397709388	-4.939415230217	-0.080609925862
C	-0.044164076415	-5.661873684025	-1.282879168931
C	-0.038738170416	-5.614837564779	1.146159208854
C	-0.056252387201	-7.050981777539	-1.234559835110
C	-0.051047665301	-7.007715119810	1.148660104527
H	-0.064095744920	-7.605515252868	-2.167652260622
H	-0.054955281129	-7.527769229320	2.101035246477
C	-0.059555082701	-7.746124854597	-0.028190549190
C	4.939437859695	-0.034557178059	-0.081545918833
C	5.615426491924	-0.036445352861	1.144988947948
C	5.661390203963	-0.037690375449	-1.284067934786
C	7.008281410055	-0.041680877080	1.146910555745
C	7.050627859351	-0.042911531281	-1.236332360208
H	7.528736758030	-0.041326920969	2.099080968745
H	7.604772933546	-0.043581676894	-2.169673593141
C	7.746261504124	-0.045399743840	-0.030321361754
C	-0.030680135819	-4.859220484225	2.437280957130
H	-0.035835814890	-5.535276966754	3.289902541355
H	0.848604402980	-4.217829946823	2.524448133452
H	-0.898005743446	-4.201987667016	2.526296821074
C	-0.041857290170	-4.953269160938	-2.600269719609
H	-0.911809965143	-4.302661959149	-2.709489611470
H	0.836841606987	-4.315134782261	-2.712960221041
H	-0.048570514704	-5.659380321195	-3.428167488807
C	-0.063754373004	-9.240655479489	-0.007449149177
H	0.857509774895	-9.646535956919	-0.429630183483
H	-0.160663937621	-9.628258487721	1.004980633178
H	-0.882556917289	-9.647172837808	-0.602580199429
C	9.240693650675	-0.063371801350	-0.009840029492
H	9.631273767746	-1.009797478967	-0.388976384163
H	9.660017564880	0.720349606981	-0.641848031430
H	9.630583249031	0.072095126108	0.997304226590
C	4.860270624042	-0.030662978905	2.436391736114
H	5.536644169931	-0.034400352385	3.288768461983
H	4.216604770121	0.846915222445	2.524139478934
H	4.205345821388	-0.899728818129	2.525282672010
C	4.952312211392	-0.033258518961	-2.601200089065
H	5.658129267114	-0.038343248843	-3.429360594412
H	4.301886255657	-0.903175899892	-2.711598959744
H	4.313920586926	0.845483286951	-2.712254982846
C	0.030766537860	4.859145962936	2.437182922383
H	-0.848425247958	4.217620959018	2.524292979295
H	0.898186792927	4.202040360836	2.526220801132
H	0.035788730023	5.535179801637	3.289823295145
C	0.041777746188	4.953341287087	-2.600365152917
H	0.048618111306	5.659474495385	-3.428243092626
H	0.911643925840	4.302615957083	-2.709565548791
H	-0.837005748920	4.315332190543	-2.713110187865
C	0.063722547905	9.240652450575	-0.007414830899

H	0.882383683567	9.647207972010	-0.602712737563
H	-0.857645238469	9.646527195086	-0.429376929790
H	0.160840784803	9.628223600610	1.005007347223
C	-4.860072084388	0.030610133377	2.436144718242
H	-4.216448615850	-0.847003355602	2.523855306082
H	-4.205091492417	0.899639613971	2.524981180101
H	-5.536383333647	0.034390883536	3.288570807201
C	-9.240681164295	0.063372228405	-0.009741318579
H	-9.630487097725	-0.072161759776	0.997426450952
H	-9.631290177482	1.009825830418	-0.388778530175
H	-9.660060140523	-0.720304102853	-0.641768208519
C	-4.952496688576	0.033312603088	-2.601440079530
H	-4.302128145936	0.903266403312	-2.711892262549
H	-4.314062510863	-0.845393268339	-2.712532769124
H	-5.658374471493	0.038352594085	-3.429549123973

131

^{3}E E_{tot}(UM06L-D3(Dichloromethane)/def2TZVP/W06) = -2940.21465921 $\langle S^2 \rangle$ = 2.0546

Ru	-0.055156637399	-0.039127270109	0.261329745429
O	-0.073366564838	-0.052382449407	2.005633708380
O	-0.102859969909	0.013618444984	-1.785709745883
C	3.337318390365	0.503688130703	0.019218343502
C	-0.604531350679	3.369269226518	0.132403424187
C	-3.458232839737	-0.583948355895	0.071777549677
C	0.488016557824	-3.452395109439	0.145882957765
N	-1.724709683138	1.172624139504	0.140715812887
N	1.144561071955	1.628895495361	0.143582508013
N	1.604133784203	-1.250767871946	0.125263408374
N	-1.261061057947	-1.709650204097	0.142392107910
C	1.610496408924	-2.619751828750	0.114975942455
C	2.901115046176	-0.825373956532	0.047496001684
C	2.509063085446	1.629864787124	0.070518799972
C	0.722843299694	2.928782621181	0.133592025999
C	-0.841241980327	-3.012548376581	0.151996933430
C	-2.627299871066	-1.709847088066	0.107187975176
C	-3.020973255313	0.745668903849	0.072662965425
C	-1.729712936506	2.537840153410	0.110267612234
C	2.967413819998	-3.073676131858	0.035483020079
C	3.759403812096	-1.972087465152	-0.009335102702
C	2.967547969553	2.987980863767	0.027571670192
C	1.870938725665	3.785769417616	0.073498628868
C	-3.087107896021	2.993253113702	0.023248970239
C	-3.879978985085	1.891949469997	-0.004629304135
C	-1.990105134845	-3.867732089285	0.132370061843
C	-3.086905038959	-3.067787588534	0.104147351157
H	3.270251479453	-4.108779234369	0.011900024836
H	4.835483741876	-1.933490820452	-0.075669743812
H	4.002151855046	3.287685198769	-0.031765996961
H	1.835253217438	4.863741628636	0.055436617164
H	-3.388469319467	4.028288647979	-0.019924818139
H	-4.955999564098	1.852181070410	-0.072007340941
H	-1.956698126045	-4.945907766167	0.134503128211
H	-4.122952217653	-3.367425668343	0.080874213377
C	4.804723199944	0.737869977006	-0.115174904253
C	5.597585338557	0.930782609583	1.022190727343
C	5.386128475339	0.762049003126	-1.392151335344
C	6.964771726650	1.145842356399	0.862440454968
C	6.753904987117	0.981377131171	-1.506090471745
H	7.576548809676	1.293695905966	1.746397973817
H	7.198565572886	1.002026984053	-2.496091266495
C	7.564144627183	1.175634302215	-0.390568191026
C	-0.834604988900	4.843543896494	0.126306029475
C	-0.929006004529	5.534840261491	-1.090764391994
C	-0.946570894803	5.535235152273	1.338283637200
C	-1.131370545985	6.909787535776	-1.071647075078
C	-1.150222510062	6.913167273080	1.312086066848
H	-1.202482736618	7.440290923175	-2.015997843736
H	-1.236071000271	7.446119781543	2.253490696679
C	-1.244472262763	7.620846703781	0.120243831475
C	-4.930926862585	-0.826162903678	0.040657153322
C	-5.579412630145	-1.086272866397	-1.176585313538
C	-5.662973281951	-0.802579399296	1.234528902152
C	-6.950573287630	-1.314411436831	-1.175451284797
C	-7.035300587856	-1.038549535300	1.190063334297
H	-7.447195074244	-1.511315021734	-2.120387816077
H	-7.597712420076	-1.022589320978	2.117994480655
C	-7.700051446481	-1.295965720008	-0.002236156027

C	0.721981626518	-4.925732876157	0.137193689863
C	0.898015880865	-5.610604625728	1.345416141292
C	0.761207551096	-5.622772749879	-1.080042093027
C	1.111013752144	-6.986955846077	1.315849876991
C	0.977319034084	-6.995721939192	-1.064155384655
H	1.245207944846	-7.514635494104	2.254541913018
H	1.007603428514	-7.530646045252	-2.008173908038
C	1.154872032457	-7.699623702311	0.124082496300
C	-4.990648873379	-0.530699078938	2.543114988665
H	-5.685703707682	-0.641373035559	3.373084864476
H	-4.150258976301	-1.205743374149	2.714994744499
H	-4.583513298888	0.481738979182	2.585242706659
C	-4.812773450346	-1.111840093619	-2.460515224287
H	-4.265622420397	-0.180448623276	-2.615793169715
H	-4.067676081208	-1.911132920264	-2.472991958609
H	-5.471949963443	-1.259761779271	-3.314117073880
C	-9.173080695046	-1.547832311493	-0.032626930331
H	-9.400734890349	-2.534829040952	-0.438792600894
H	-9.611587784370	-1.488774318157	0.961782150806
H	-9.688357940926	-0.825400103935	-0.667732890958
C	1.387196122402	-9.176102286184	0.109321610107
H	0.571528630556	-9.703793108220	-0.387477976355
H	2.297879871760	-9.430741934078	-0.435567345420
H	1.479921302675	-9.578453879189	1.116363722477
C	0.858828770684	-4.881955671739	2.651166239195
H	0.971546901457	-5.567407031649	3.488726654018
H	1.653930023157	-4.136869321225	2.721031148622
H	-0.079830595859	-4.340766520943	2.784254477452
C	0.572410290968	-4.905089341359	-2.379552453777
H	0.661507925332	-5.586965040560	-3.223030555380
H	-0.409862546489	-4.429598339787	-2.438500053986
H	1.309330691205	-4.109884491909	-2.512403712286
C	4.992891512399	0.907297970701	2.390069958012
H	4.260451152356	1.706431955054	2.520974416466
H	4.462979142134	-0.027709124353	2.581149110807
H	5.753033948076	1.024866719740	3.159897984973
C	4.553411525805	0.546135541402	-2.616583787694
H	5.135256190057	0.705565206090	-3.522316573693
H	4.154323315205	-0.470209156684	-2.655337605865
H	3.691560483322	1.215705453839	-2.646425757178
C	9.032375655110	1.410179375436	-0.544544619541
H	9.516705562792	0.582186198688	-1.064714808395
H	9.233338295843	2.306133298671	-1.134330206431
H	9.523538304222	1.530445398873	0.419308194205
C	-0.847841029368	4.812968831072	2.644477427128
H	-1.613623817362	4.040434777846	2.737865396913
H	0.112777309627	4.305788955782	2.753856646588
H	-0.960519089316	5.497340462969	3.482957812333
C	-1.459832078567	9.099981614329	0.109458593709
H	-1.535870111171	9.502497489261	1.117859552012
H	-0.642628453089	9.618223788091	-0.395077679623
H	-2.372611729128	9.366710237149	-0.425786715643
C	-0.825979888703	4.803496789312	-2.391173646201
H	0.104807462766	4.237714878103	-2.469139904201
H	-1.633926254552	4.077816517931	-2.502185705385
H	-0.875262491207	5.487643702389	-3.236056239256
C	0.007901500363	-1.052317429242	-2.611216334859
C	-0.688162342128	-1.035561490810	-3.898754015901
C	-1.611011722317	-0.110045943581	-4.402770771495
H	-0.469835735013	-1.898058756867	-4.523354459656
H	1.074981296985	-1.275915983057	-2.838052978151
H	-0.300415497283	-2.010612936141	-2.133767797154
H	-1.489282754585	1.329942958694	-2.798996218371
C	-1.982164712918	1.085444886610	-3.729697231548
C	-2.957315025628	1.905604440642	-4.257369547286
H	-3.238892712495	2.807922016267	-3.728917209469
H	-4.345744821348	2.241056129972	-5.862803743816
C	-3.583828837722	1.586740769725	-5.458716972622
C	-3.223143697511	0.428223644340	-6.146718312874
C	-2.254399026889	-0.401589253669	-5.635344484688
H	-1.973857461791	-1.306336009228	-6.161470590112
H	-3.707024221140	0.182919492188	-7.083394278896

131

^{3}F E_{tot}(UM06L-D3(Dichloromethane)/def2TZVP/W06) = -2940.24519724 $\langle S^2 \rangle$ = 2.0128

Ru	0.144136483754	-0.022543332220	0.314651845399
O	0.001492833021	0.014687234125	2.057215824980

C	3.594175071259	-0.105766736959	0.263438899933
C	0.241784874983	3.422728213205	0.053598285507
C	-3.275643676172	0.052781892545	-0.183292394707
C	0.072851819661	-3.474761075723	0.162162016151
N	-1.250977029699	1.457088507570	-0.018128706500
N	1.635264538050	1.392989949453	0.209973071082
N	1.564997914143	-1.510523162793	0.251013437592
N	-1.323122210449	-1.442597618226	0.038496686647
C	1.326594568150	-2.858803416865	0.220828407973
C	2.922605402617	-1.332030271643	0.258091618967
C	2.981579282896	1.150266792481	0.246741941405
C	1.462252583897	2.750109424457	0.162034499892
C	-1.148774464043	-2.802366415700	0.065700535967
C	-2.665499919816	-1.202505361819	-0.098045785354
C	-2.602391887447	1.278001583853	-0.165681591228
C	-1.007341864326	2.804563794319	-0.061683474628
C	2.579911794217	-3.553096561116	0.220181154062
C	3.560502037060	-2.615088081786	0.236762137313
C	3.680819658411	2.401410338336	0.241062350279
C	2.747206398435	3.384679099916	0.196887984578
C	-2.247024966438	3.495515247305	-0.256848296561
C	-3.226793012796	2.558011214994	-0.320724827867
C	-2.426408209075	-3.437406835236	-0.058774700529
C	-3.357824376840	-2.454877083150	-0.161204735793
H	2.689885724396	-4.626013792339	0.197606351845
H	4.627565134683	-2.773376550898	0.232875453160
H	4.754122357086	2.507706555505	0.263017116012
H	2.909305372781	4.450904995159	0.174247224962
H	-2.349408061704	4.566343671011	-0.338198108466
H	-4.284387508664	2.714644294380	-0.464881062400
H	-2.585656194310	-4.504301983133	-0.072699010054
H	-4.425156007984	-2.564124727579	-0.273850632701
C	5.085056909038	-0.142451934066	0.235979308425
C	5.810848432516	-0.269227458903	1.425998896261
C	5.756203678542	-0.053833342547	-0.993372922431
C	7.202115793496	-0.307165633176	1.366350809158
C	7.145298786927	-0.099448044506	-1.007804804410
H	7.761637270676	-0.403326593495	2.291196437340
H	7.660093505584	-0.035025293266	-1.961254832487
C	7.889999496662	-0.225424038515	0.161959161693
C	0.272287298948	4.914702503967	0.062396502465
C	0.532050132724	5.629893320706	-1.112962166631
C	0.038963665214	5.599644481170	1.265597139139
C	0.549877024304	7.022173869860	-1.066006633991
C	0.068274445920	6.989290718171	1.267465321597
H	0.748321057566	7.572068727313	-1.980330008450
H	-0.109835067909	7.513373528159	2.201225983876
C	0.320689997526	7.722798082676	0.111422321459
C	-4.761254432478	0.083690671263	-0.321236577699
C	-5.348984805271	0.050504547831	-1.591152169516
C	-5.567272896548	0.141208323551	0.825731109762
C	-6.737610538468	0.076011916076	-1.695257334602
C	-6.949278434454	0.164211026382	0.677216198435
H	-7.188034561321	0.051400877279	-2.682206118910
H	-7.568716125204	0.208396785468	1.567448842109
C	-7.557351362599	0.132290720871	-0.574910030064
C	0.038585353957	-4.966464444206	0.173613143358
C	0.000686158812	-5.652172607202	1.397204822450
C	0.043814097390	-5.679589663941	-1.031249937435
C	-0.032444070627	-7.041848240491	1.390085948277
C	0.011533084988	-7.071596774509	-0.992858291327
H	-0.064094991449	-7.567672180527	2.339136009964
H	0.016939436266	-7.620311188778	-1.929106301686
C	-0.026842527607	-7.773539432575	0.205567522743
C	-4.952814667862	0.175891479586	2.188949967760
H	-5.714922039505	0.222345802506	2.964269925779
H	-4.337658448422	-0.706397757276	2.376547309021
H	-4.295237062706	1.038521007850	2.313365373249
C	-4.502901596465	-0.005135466471	-2.823267936831
H	-3.832625480086	0.855321809456	-2.884323245429
H	-3.867810671484	-0.894310469973	-2.840052689664
H	-5.113761946488	-0.015601449225	-3.723900302854
C	-9.046424990621	0.157316093503	-0.701881530277
H	-9.361420113591	0.138705038915	-1.743558561945
H	-9.503755609768	-0.697288683228	-0.200464601935
H	-9.471362365131	1.050289404347	-0.240674956714
C	-0.055752804581	-9.267762947558	0.230419017690

H	-0.910599124033	-9.641787444749	0.795692772670
H	-0.110055693877	-9.685402093533	-0.773251060868
H	0.835555174420	-9.675107504356	0.711070611072
C	-0.004946964498	-4.903328670038	2.692137859178
H	-0.074704784361	-5.582026503167	3.539827564564
H	0.900863408629	-4.306476465435	2.816416135961
H	-0.841572912477	-4.204504813223	2.751304571660
C	0.077948574230	-4.965360517144	-2.345576809776
H	0.140146400497	-5.667610807433	-3.174600742935
H	-0.816948856448	-4.356548264094	-2.494256573472
H	0.930549820225	-4.286806477059	-2.416319020659
C	5.110143959067	-0.364698302505	2.744020187718
H	4.428556223080	0.473229721022	2.900893783715
H	4.503199644478	-1.269885697914	2.812834811520
H	5.820344275728	-0.379350327732	3.568405643699
C	4.993425941682	0.097767399028	-2.272165996496
H	5.650199350872	0.013622437342	-3.135896111998
H	4.206945956275	-0.653791212375	-2.369409064674
H	4.497875915193	1.070111896362	-2.327906699575
C	9.383406999071	-0.268651326216	0.115252038360
H	9.813223313966	-0.387717362570	1.108072851975
H	9.739782666108	-1.093548155090	-0.503810500131
H	9.795424767193	0.644247468801	-0.318232900271
C	-0.237561543009	4.851534649753	2.531478250582
H	-1.180426210222	4.302982952975	2.478469248543
H	0.535555370016	4.110004455362	2.741072577350
H	-0.296179667598	5.526820006664	3.382807607027
C	0.345148681730	9.216936893724	0.144769511504
H	-0.603380518466	9.623929192271	0.498869524782
H	1.113971004066	9.588112454420	0.824576078950
H	0.541102810848	9.637854357754	-0.839684064911
C	0.791823170883	4.921918865768	-2.404586440766
H	1.710847028879	4.333068969631	-2.363881905922
H	-0.008422203801	4.222448104451	-2.655003389156
H	0.888929417154	5.628210838065	-3.226999063374
O	0.456435447260	-0.050693119086	-2.006921883172
H	-0.552269496897	-1.859742099619	-2.502389418467
C	0.019096174277	-1.043268240832	-2.933904171552
H	0.769439818943	-1.320722928656	-3.668087511124
C	-0.564796994276	0.295424780560	-2.965562537895
H	-1.550422639038	0.403218139745	-2.519537096862
C	-0.195665110330	1.331678199854	-3.949362220645
H	1.907378455105	0.907297002375	-3.897383124645
C	1.129698489145	1.521249453234	-4.336482062944
H	2.484452768677	2.645237165057	-5.552589363289
C	1.451502570943	2.499465973057	-5.262383057453
C	0.455066656201	3.294791588002	-5.814406134403
H	0.709688834659	4.059495064350	-6.537190269363
C	-0.864977005845	3.114211767978	-5.428525967881
H	-1.644354637172	3.738074096832	-5.847293363079
C	-1.188123913541	2.141290053510	-4.494287705324
H	-2.216857289764	2.005465490980	-4.179618031389

131

[1]TS(A) E_{tot}(RM06L-D3(Dichloromethane)/def2TZVP/W06) = -2940.18668517 $\langle S^2 \rangle$ = 0.0000

Ru	-0.091651035562	-0.010804040045	0.090250816087
O	0.035445761345	-0.044119975274	1.821778327878
O	0.130987283586	0.226685576499	-1.700013110398
C	3.330866733412	0.539159389590	0.122325947490
C	-0.601494547445	3.408831910012	0.113045067991
C	-3.477001399341	-0.536928505775	0.009204811855
C	0.475852240358	-3.407545636853	-0.138261866841
N	-1.735971126375	1.208476814649	0.059876319031
N	1.148555580626	1.684476155971	0.180833622929
N	1.602724108406	-1.225766215959	0.005515970709
N	-1.272843175431	-1.652580367902	-0.096309633761
C	1.608594225280	-2.585030998113	-0.056867256398
C	2.895735437573	-0.791103255773	0.054299856090
C	2.508900263591	1.672557358214	0.171573311191
C	0.730718321995	2.975859775121	0.160600047300
C	-0.846383919886	-2.957144341847	-0.169925822300
C	-2.645140741367	-1.655646820405	-0.095893800567
C	-3.036537540435	0.790924806743	0.049068983548
C	-1.726123467206	2.578646667334	0.082668402608
C	2.968018893488	-3.039323187228	-0.046318972650
C	3.758629517661	-1.936671905855	0.017677396308
C	2.977751266769	3.032525226331	0.172636247153

C	1.884225965815	3.834234998117	0.162948403716
C	-3.082414340925	3.038282653576	0.082130965822
C	-3.885576198311	1.942844440737	0.073739513799
C	-1.997119994956	-3.804844487427	-0.246136174610
C	-3.096880332513	-3.009454435419	-0.194602460207
H	3.274139060529	-4.072843962063	-0.087271639560
H	4.836336419686	-1.897160260351	0.042269870168
H	4.015442358752	3.327406506036	0.172580914582
H	1.852405952009	4.912467013973	0.154082942493
H	-3.379307987516	4.075194216021	0.093323321166
H	-4.963786936103	1.912999644751	0.073347452298
H	-1.962768551351	-4.880270862797	-0.322540523799
H	-4.132353105774	-3.310481985156	-0.222830524608
C	4.804624714766	0.769569591563	0.106630170955
C	5.507327307743	0.902649218766	1.309518183605
C	5.480253409508	0.851999864435	-1.120343877219
C	6.883234364642	1.115430308908	1.265187287110
C	6.853369740092	1.067279399060	-1.118537582385
H	7.426290298823	1.216209360768	2.199273512218
H	7.372828526263	1.132984976601	-2.069344212732
C	7.576320120832	1.201408258721	0.064071491520
C	-0.839656376221	4.880159243292	0.058699192586
C	-0.989747433459	5.509155194496	-1.186889370502
C	-0.912245539492	5.627534242410	1.239760419246
C	-1.209845314216	6.881031064730	-1.224717038009
C	-1.136723638618	6.999658395584	1.155386211866
H	-1.323589184989	7.364816736447	-2.189685272410
H	-1.195988124831	7.576376447208	2.072688854977
C	-1.287847283140	7.647123480884	-0.064596245112
C	-4.945167529549	-0.773872531027	0.137833917215
C	-5.770558255466	-0.806920419725	-0.994686965645
C	-5.494503675471	-0.955516790466	1.413939113536
C	-7.134505256601	-1.018117355290	-0.830157672224
C	-6.866267044096	-1.165372194017	1.533538918536
H	-7.768281789429	-1.043825826843	-1.710934779241
H	-7.287821030685	-1.305534834785	2.523556400589
C	-7.705397049666	-1.198986533386	0.426677896411
C	0.703875537385	-4.882090596628	-0.162724679869
C	0.710680313698	-5.600648214180	1.039519019321
C	0.919795592079	-5.543699443058	-1.380362903824
C	0.934163936986	-6.974967994845	1.001532992480
C	1.137825933162	-6.916452715872	-1.372654906297
H	0.941902044208	-7.528131766568	1.935050584111
H	1.304384640073	-7.423505085606	-2.317787884899
C	1.149537487405	-7.653554794332	-0.191571092310
C	-4.629871006399	-0.926290861228	2.634555795893
H	-5.219134410622	-1.070098408819	3.537953677097
H	-3.865381350105	-1.705578512762	2.607683570621
H	-4.096661906741	0.021689838615	2.730445380477
C	-5.189788029150	-0.631137583183	-2.359728263554
H	-4.684496268117	0.331154326425	-2.468678129510
H	-4.438277058505	-1.394370538151	-2.570402727949
H	-5.955616620988	-0.695177036183	-3.130467949269
C	-9.177067872563	-1.414918817004	0.573403733796
H	-9.522733494640	-2.247118161921	-0.041356768215
H	-9.451984856016	-1.623929730633	1.605547805199
H	-9.741188353776	-0.537241183976	0.252405084142
C	1.379109483035	-9.130261444046	-0.214560954217
H	0.565220032037	-9.650799176086	-0.722793964355
H	2.293269616699	-9.385082897600	-0.752325875076
H	1.455648146659	-9.540785912406	0.790546372431
C	0.482693699119	-4.911540099948	2.347596717633
H	0.566743796490	-5.607942304157	3.179416165082
H	1.200227233783	-4.105257803403	2.511443438753
H	-0.507898759349	-4.454355281959	2.395551794507
C	0.916402820059	-4.786830586596	-2.670105628691
H	1.067363883961	-5.450575540946	-3.519066099152
H	-0.026239586783	-4.257351389133	-2.822712804858
H	1.703503840246	-4.029905976258	-2.695427205110
C	4.798041867264	0.818603773808	2.623785196856
H	4.063458216874	1.618589664826	2.737046269606
H	4.247919313971	-0.118641045751	2.727163575612
H	5.496949461906	0.890241447253	3.454713377958
C	4.737952817911	0.712311756665	-2.412141684215
H	5.400383530487	0.849451739254	-3.264451492105
H	4.273557601395	-0.272090455800	-2.507058548567
H	3.929014764544	1.441378910625	-2.494656240765

```
C    9.052982785339    1.431062476378    0.033840758101
H    9.573133835257    0.615965444195   -0.472038211674
H    9.303713690043    2.343087734485   -0.510338739291
H    9.466930699571    1.517763372318    1.036714639795
C   -0.754826815973    4.969089689636    2.573545127183
H   -1.480613402316    4.166185839234    2.715714561242
H    0.231377669120    4.513829923047    2.684679466692
H   -0.883204095323    5.683719967359    3.384025799046
C   -1.521778277489    9.121601441210   -0.137959700273
H   -1.612522905133    9.563983712536    0.852344315490
H   -0.704654356587    9.628821123256   -0.654079959213
H   -2.431138892368    9.355014503698   -0.693703455818
C   -0.916984566271    4.719933226263   -2.456378039771
H    0.012974651918    4.151708849997   -2.527974021282
H   -1.726511738420    3.989745666846   -2.525260873513
H   -0.981856562903    5.368508545661   -3.327946735024
C    0.362357590540   -0.939537501040   -3.011727610148
C   -0.898853368329   -1.348893887020   -3.432644448568
C   -1.751769162262   -0.563082653391   -4.264087807115
H   -1.298371536253   -2.286073856170   -3.057453051522
H    0.916411413041   -0.256496727119   -3.643864841875
H    0.987600044091   -1.671825857953   -2.514042391303
H   -0.657988906770    1.281317377499   -4.021223548867
C   -1.506223087993    0.805962214044   -4.499467483225
C   -2.355344333355    1.550279338099   -5.294318711990
H   -2.157846420072    2.603950685661   -5.449209987960
H   -4.123854119440    1.544736706114   -6.517066042494
C   -3.463988389574    0.957686484234   -5.891333986800
C   -3.722912141136   -0.391456531620   -5.672996853380
C   -2.889289371316   -1.137200558032   -4.864114520352
H   -3.098474906995   -2.185802875495   -4.685113394608
H   -4.586922007451   -0.857427180132   -6.130539281654
```

131

[1,3]MECP(A) [singlet] E_{tot}(RM06L-D3(Dichloromethane)/def2TZVP/W06) = -2940.19093475 $\langle S^2 \rangle$ = 0.0000

```
Ru   0.176482000000   -0.062384250000    0.035630040000
O   -0.030363170000   -0.197900150000    1.748115180000
C    3.609093630000   -0.199150800000    0.410927180000
C    0.306411060000    3.349838560000   -0.213029340000
C   -3.253627290000    0.006906180000   -0.361171330000
C    0.082878840000   -3.530554110000    0.034054160000
N   -1.200727470000    1.390913400000   -0.268277080000
N    1.675738680000    1.318051090000    0.082886630000
N    1.596700550000   -1.596530900000    0.247023340000
N   -1.321340380000   -1.504276490000   -0.180334640000
C    1.336754570000   -2.931443680000    0.208405020000
C    2.936430780000   -1.427425820000    0.383153200000
C    3.010332860000    1.056997350000    0.275342680000
C    1.515467020000    2.673082120000   -0.008556280000
C   -1.135240680000   -2.857038380000   -0.130837510000
C   -2.655226810000   -1.259347100000   -0.287502270000
C   -2.559762390000    1.218098970000   -0.388915470000
C   -0.941070990000    2.737566180000   -0.352177120000
C    2.579339980000   -3.645422920000    0.345835030000
C    3.563325230000   -2.720406940000    0.456062790000
C    3.709914490000    2.302553050000    0.311473910000
C    2.793770500000    3.292998260000    0.141145900000
C   -2.176735070000    3.429458980000   -0.557266270000
C   -3.167904230000    2.501735020000   -0.566170560000
C   -2.415902690000   -3.492659230000   -0.235790820000
C   -3.351318120000   -2.510592030000   -0.321078990000
H    2.676859850000   -4.719708460000    0.352426520000
H    4.622998240000   -2.888681000000    0.567499250000
H    4.774115680000    2.405700260000    0.453534850000
H    2.968634280000    4.357080150000    0.116390730000
H   -2.270214760000    4.497988970000   -0.672070190000
H   -4.226309970000    2.665417550000   -0.695722350000
H   -2.576843100000   -4.559249050000   -0.230164940000
H   -4.421103980000   -2.620224110000   -0.405089260000
C    5.092345230000   -0.234941390000    0.562932210000
C    5.665770130000   -0.267871360000    1.839171440000
C    5.907321370000   -0.238359020000   -0.579273610000
C    7.053674480000   -0.301824720000    1.952701420000
C    7.287538920000   -0.275110480000   -0.419978630000
H    7.495573600000   -0.325319980000    2.943483350000
H    7.915154640000   -0.279833120000   -1.305560430000
C    7.883341260000   -0.306257220000    0.838268180000
```

C	0.355401580000	4.840732630000	-0.241639440000
C	0.733389340000	5.515285950000	-1.408895610000
C	0.033132890000	5.564602140000	0.917635470000
C	0.774372070000	6.907784800000	-1.400043870000
C	0.091251620000	6.952782680000	0.882653750000
H	1.061913120000	7.426701920000	-2.308702320000
H	-0.154828380000	7.507657040000	1.782515790000
C	0.457637070000	7.646759000000	-0.267289060000
C	-4.745641460000	0.060273550000	-0.360493950000
C	-5.472561500000	0.017082540000	-1.555396700000
C	-5.418371640000	0.146602850000	0.869472070000
C	-6.864013610000	0.060792690000	-1.502137850000
C	-6.807363910000	0.188897510000	0.876388140000
H	-7.422571530000	0.025397060000	-2.431822630000
H	-7.321830630000	0.254451900000	1.829820090000
C	-7.552462190000	0.146843430000	-0.298974620000
C	0.037778020000	-5.021780060000	0.023001190000
C	-0.038091620000	-5.729706930000	1.231779370000
C	0.070292240000	-5.709285280000	-1.196098650000
C	-0.082605260000	-7.118402750000	1.195742080000
C	0.027769630000	-7.101539470000	-1.185799260000
H	-0.145542090000	-7.662656140000	2.132764600000
H	0.057027420000	-7.631964080000	-2.132034700000
C	-0.049864420000	-7.826267500000	-0.002972930000
C	-4.657728100000	0.193360390000	2.156958200000
H	-5.331295870000	0.232948250000	3.010552260000
H	-4.012871220000	-0.679301800000	2.278739030000
H	-4.002680310000	1.065649210000	2.208150820000
C	-4.778146700000	-0.054382120000	-2.877302510000
H	-4.249692300000	0.876309910000	-3.097954130000
H	-4.031674290000	-0.850099950000	-2.906203620000
H	-5.485103880000	-0.226622050000	-3.686538640000
C	-9.045839740000	0.187893240000	-0.258260070000
H	-9.475403220000	0.187712250000	-1.258294990000
H	-9.452854790000	-0.670829940000	0.278426680000
H	-9.406622450000	1.076978580000	0.261098720000
C	-0.093695570000	-9.320278300000	-0.008567250000
H	-0.978896360000	-9.695533510000	0.507119960000
H	-0.103761210000	-9.718225500000	-1.021524780000
H	0.768833120000	-9.746136220000	0.507034170000
C	-0.071771240000	-5.006385550000	2.540715570000
H	-0.213185770000	-5.697613620000	3.369099420000
H	0.854702160000	-4.457267810000	2.721436490000
H	-0.876205550000	-4.269262200000	2.575537230000
C	0.141256120000	-4.967591510000	-2.493764790000
H	0.292804650000	-5.648260700000	-3.329130600000
H	-0.777762700000	-4.409135870000	-2.686801890000
H	0.952852260000	-4.237256250000	-2.501370830000
C	4.808126940000	-0.264893010000	3.064582940000
H	4.151201500000	0.606518990000	3.095475330000
H	4.156931390000	-1.140515850000	3.103759240000
H	5.412898710000	-0.259929430000	3.969151620000
C	5.304295620000	-0.203564090000	-1.948245970000
H	6.070413890000	-0.267024140000	-2.718374600000
H	4.603777030000	-1.026818620000	-2.102472540000
H	4.738537970000	0.715483160000	-2.117033980000
C	9.371027270000	-0.344355920000	0.977354990000
H	9.676759090000	-0.361506440000	2.021766230000
H	9.794286200000	-1.225158500000	0.491673200000
H	9.837978230000	0.522708520000	0.507309500000
C	-0.363277030000	4.861877870000	2.177665930000
H	-1.317216630000	4.341590140000	2.068071610000
H	0.367599060000	4.104322290000	2.466232490000
H	-0.464667770000	5.563316920000	3.003367850000
C	0.509967520000	9.140351530000	-0.273359330000
H	-0.454717050000	9.574644310000	-0.006333140000
H	1.230661430000	9.516738940000	0.454563670000
H	0.791650890000	9.528671990000	-1.250270320000
C	1.090205100000	4.762532970000	-2.650400230000
H	2.009039000000	4.184860230000	-2.525029320000
H	0.315040210000	4.045093010000	-2.926242300000
H	1.238512400000	5.437710630000	-3.490871630000
O	0.479251550000	-0.347087810000	-1.763803020000
H	-0.971300100000	-1.501625770000	-2.745838660000
C	-0.360807360000	-0.657613110000	-3.064157790000
H	0.405796390000	-0.983162660000	-3.763209850000
C	-1.077864260000	0.506579630000	-3.405259020000

H	-2.023246110000	0.679415750000	-2.906741870000
C	-0.609167640000	1.534930420000	-4.253480470000
H	1.397760970000	0.772529000000	-4.528181000000
C	0.695828750000	1.550863890000	-4.801082350000
H	2.097655890000	2.548003880000	-6.061562510000
C	1.091428850000	2.554303840000	-5.660783600000
C	0.213026320000	3.575122420000	-6.014184640000
H	0.529621340000	4.355634110000	-6.693721160000
C	-1.070054700000	3.592389740000	-5.473645480000
H	-1.755324630000	4.389641820000	-5.733807750000
C	-1.471018430000	2.603100240000	-4.601554120000
H	-2.470245890000	2.618758590000	-4.180659940000

131

[1,3]MECP(A) [triplet] E_{tot}(UM06L-D3(Dichloromethane)/def2TZVP/W06) = -2940.19093430 $\langle S^2 \rangle$ = 2.0329

Ru	0.176482000000	-0.062384250000	0.035630040000
O	-0.030363170000	-0.197900150000	1.748115180000
C	3.609093630000	-0.199150800000	0.410927180000
C	0.306411060000	3.349838560000	-0.213029340000
C	-3.253627290000	0.006906180000	-0.361171330000
C	0.082878840000	-3.530554110000	0.034054160000
N	-1.200727470000	1.390913400000	-0.268277080000
N	1.675738680000	1.318051090000	0.082886630000
N	1.596700550000	-1.596530900000	0.247023340000
N	-1.321340380000	-1.504276490000	-0.180334640000
C	1.336754570000	-2.931443680000	0.208405020000
C	2.936430780000	-1.427425820000	0.383153200000
C	3.010332860000	1.056997350000	0.275342680000
C	1.515467020000	2.673082120000	-0.008556280000
C	-1.135240680000	-2.857038380000	-0.130837510000
C	-2.655226810000	-1.259347100000	-0.287502270000
C	-2.559762390000	1.218098970000	-0.388915470000
C	-0.941070990000	2.737566180000	-0.352177120000
C	2.579339980000	-3.645422920000	0.345835030000
C	3.563325230000	-2.720406940000	0.456062790000
C	3.709914490000	2.302553050000	0.311473910000
C	2.793770500000	3.292998260000	0.141145900000
C	-2.176735070000	3.429458980000	-0.557266270000
C	-3.167904230000	2.501735020000	-0.566170560000
C	-2.415902690000	-3.492659230000	-0.235790820000
C	-3.351318120000	-2.510592030000	-0.321078990000
H	2.676859850000	-4.719708460000	0.352426520000
H	4.622998240000	-2.888681000000	0.567499250000
H	4.774115680000	2.405700260000	0.453534850000
H	2.968634280000	4.357080150000	0.116390730000
H	-2.270214760000	4.497988970000	-0.672070190000
H	-4.226309970000	2.665417550000	-0.695722350000
H	-2.576843100000	-4.559249050000	-0.230164940000
H	-4.421103980000	-2.620224110000	-0.405089260000
C	5.092345230000	-0.234941390000	0.562932210000
C	5.665770130000	-0.267871360000	1.839171440000
C	5.907321370000	-0.238359020000	-0.579273610000
C	7.053674480000	-0.301824720000	1.952701420000
C	7.287538920000	-0.275110480000	-0.419978630000
H	7.495573600000	-0.325319980000	2.943483350000
H	7.915154640000	-0.279833120000	-1.305560430000
C	7.883341260000	-0.306257220000	0.838268180000
C	0.355401580000	4.840732630000	-0.241639440000
C	0.733389340000	5.515285950000	-1.408895610000
C	0.033132890000	5.564602140000	0.917635470000
C	0.774372070000	6.907784800000	-1.400043870000
C	0.091251620000	6.952782680000	0.882653750000
H	1.061913120000	7.426701920000	-2.308702320000
H	-0.154828380000	7.507657040000	1.782515790000
C	0.457637070000	7.646759000000	-0.267289060000
C	-4.745641460000	0.060273550000	-0.360493950000
C	-5.472561500000	0.017082540000	-1.555396700000
C	-5.418371640000	0.146602850000	0.869472070000
C	-6.864013610000	0.060792690000	-1.502137850000
C	-6.807363910000	0.188897510000	0.876388140000
H	-7.422571530000	0.025397060000	-2.431822630000
H	-7.321830630000	0.254451900000	1.829820090000
C	-7.552462190000	0.146843430000	-0.298974620000
C	0.037778020000	-5.021780060000	0.023001190000
C	-0.038091620000	-5.729706930000	1.231779370000
C	0.070292240000	-5.709285280000	-1.196098650000
C	-0.082605260000	-7.118402750000	1.195742080000

C	0.027769630000	-7.101539470000	-1.185799260000
H	-0.145542090000	-7.662656140000	2.132764600000
H	0.057027420000	-7.631964080000	-2.132034700000
C	-0.049864420000	-7.826267500000	-0.002972930000
C	-4.657728100000	0.193360390000	2.156958200000
H	-5.331295870000	0.232948250000	3.010552260000
H	-4.012871220000	-0.679301800000	2.278739030000
H	-4.002680310000	1.065649210000	2.208150820000
C	-4.778146700000	-0.054382120000	-2.877302510000
H	-4.249692300000	0.876309910000	-3.097954130000
H	-4.031674290000	-0.850099950000	-2.906203620000
H	-5.485103880000	-0.226622050000	-3.686538640000
C	-9.045839740000	0.187893240000	-0.258260070000
H	-9.475403220000	0.187712250000	-1.258294990000
H	-9.452854790000	-0.670829940000	0.278426680000
H	-9.406622450000	1.076978580000	0.261098720000
C	-0.093695570000	-9.320278300000	-0.008567250000
H	-0.978896360000	-9.695533510000	0.507119960000
H	-0.103761210000	-9.718225500000	-1.021524780000
H	0.768833120000	-9.746136220000	0.507034170000
C	-0.071771240000	-5.006385550000	2.540715570000
H	-0.213185770000	-5.697613620000	3.369099420000
H	0.854702160000	-4.457267810000	2.721436490000
H	-0.876205550000	-4.269262200000	2.575537230000
C	0.141256120000	-4.967591510000	-2.493764790000
H	0.292804650000	-5.648260700000	-3.329130600000
H	-0.777762700000	-4.409135870000	-2.686801890000
H	0.952852260000	-4.237256250000	-2.501370830000
C	4.808126940000	-0.264893010000	3.064582940000
H	4.151201500000	0.606518990000	3.095475330000
H	4.156931390000	-1.140515850000	3.103759240000
H	5.412898710000	-0.259929430000	3.969151620000
C	5.304295620000	-0.203564090000	-1.948245970000
H	6.070413890000	-0.267024140000	-2.718374600000
H	4.603777030000	-1.026818620000	-2.102472540000
H	4.738537970000	0.715483160000	-2.117033980000
C	9.371027270000	-0.344355920000	0.977354990000
H	9.676759090000	-0.361506440000	2.021766230000
H	9.794286200000	-1.225158500000	0.491673200000
H	9.837978230000	0.522708520000	0.507309500000
C	-0.363277030000	4.861877870000	2.177665930000
H	-1.317216630000	4.341590140000	2.068071610000
H	0.367599060000	4.104322290000	2.466232490000
H	-0.464667770000	5.563316920000	3.003367850000
C	0.509967520000	9.140351530000	-0.273359330000
H	-0.454717050000	9.574644310000	-0.006333140000
H	1.230661430000	9.516738940000	0.454563670000
H	0.791650890000	9.528671990000	-1.250270320000
C	1.090205100000	4.762532970000	-2.650400230000
H	2.009039000000	4.184860230000	-2.525029320000
H	0.315040210000	4.045093010000	-2.926242300000
H	1.238512400000	5.437710630000	-3.490871630000
O	0.479251550000	-0.347087810000	-1.763803020000
H	-0.971300100000	-1.501625770000	-2.745838660000
C	-0.360807360000	-0.657613110000	-3.064157790000
H	0.405796390000	-0.983162660000	-3.763209850000
C	-1.077864260000	0.506579630000	-3.405259020000
H	-2.023246110000	0.679415750000	-2.906741870000
C	-0.609167640000	1.534930420000	-4.253480470000
H	1.397760970000	0.772529000000	-4.528181000000
C	0.695828750000	1.550863890000	-4.801082350000
H	2.097655890000	2.548003880000	-6.061562510000
C	1.091428850000	2.554303840000	-5.660783600000
C	0.213026320000	3.575122420000	-6.014184640000
H	0.529621340000	4.355634110000	-6.693721160000
C	-1.070054700000	3.592389740000	-5.473645480000
H	-1.755324630000	4.389641820000	-5.733807750000
C	-1.471018430000	2.603100240000	-4.601554120000
H	-2.470245890000	2.618758590000	-4.180659940000

131

^{BS,3}**MECP(A)** [singlet] E_{tot}(UM06L-D3(Dichloromethane)/def2TZVP/W06) = -2940.20921705 $\langle S^2 \rangle$ = 1.0421

Ru	-0.080900200000	-0.034609070000	0.099160000000
O	-0.018821980000	-0.103880260000	1.820383980000
O	0.145382460000	0.094224050000	-1.854804150000
C	3.339573200000	0.475450710000	0.088517430000
C	-0.570889240000	3.383145710000	0.036226560000

C	-3.468735140000	-0.546445730000	-0.128969110000
C	0.452731340000	-3.453182370000	-0.029977100000
N	-1.707364270000	1.185375090000	-0.036958440000
N	1.157228760000	1.633203730000	0.102103660000
N	1.595791840000	-1.272447570000	0.031596880000
N	-1.279057540000	-1.684552730000	-0.096012480000
C	1.590366130000	-2.635093240000	0.018203440000
C	2.893811250000	-0.852016810000	0.057774250000
C	2.522186270000	1.611202520000	0.108163760000
C	0.754356250000	2.932610480000	0.090340400000
C	-0.864815950000	-2.994569520000	-0.092486910000
C	-2.646742630000	-1.677452280000	-0.138920920000
C	-3.013430060000	0.775427430000	-0.108527440000
C	-1.695945460000	2.559838560000	-0.035054700000
C	2.944584500000	-3.101386720000	0.032951880000
C	3.746059520000	-2.004827300000	0.054269480000
C	3.000169500000	2.964402590000	0.116052790000
C	1.913613690000	3.776899300000	0.108563030000
C	-3.046124580000	3.024638480000	-0.115512820000
C	-3.852761160000	1.932629230000	-0.157916490000
C	-2.020533540000	-3.837278420000	-0.151363790000
C	-3.112031470000	-3.031070630000	-0.176394440000
H	3.239728660000	-4.138815220000	0.025218790000
H	4.824192810000	-1.973832110000	0.069419690000
H	4.040396870000	3.249614570000	0.123360240000
H	1.891874240000	4.855265520000	0.105197230000
H	-3.336966340000	4.063095040000	-0.137926110000
H	-4.929209430000	1.907568420000	-0.222161780000
H	-1.993636540000	-4.915352340000	-0.168735730000
H	-4.149441150000	-3.323125240000	-0.218899580000
C	4.815063860000	0.696929130000	0.089642120000
C	5.500450240000	0.838407430000	1.301915120000
C	5.510470860000	0.760919850000	-1.127211250000
C	6.877940970000	1.043532180000	1.276576340000
C	6.884532350000	0.969332270000	-1.106304340000
H	7.406890880000	1.151379640000	2.217909100000
H	7.418970770000	1.020747840000	-2.049643710000
C	7.589923640000	1.113334580000	0.085533910000
C	-0.790475610000	4.858779860000	0.044128070000
C	-0.821522730000	5.568051450000	-1.165860080000
C	-0.951528910000	5.532801920000	1.260436310000
C	-1.013212350000	6.944197810000	-1.134821050000
C	-1.142370260000	6.912590730000	1.245513030000
H	-1.035399080000	7.489341050000	-2.073150150000
H	-1.266386700000	7.432308660000	2.189991710000
C	-1.176105170000	7.638322940000	0.061256940000
C	-4.944639340000	-0.767469370000	-0.099530470000
C	-5.654442960000	-1.004752860000	-1.285916890000
C	-5.618216130000	-0.739051870000	1.128842280000
C	-7.028584510000	-1.203408110000	-1.221254600000
C	-6.995571530000	-0.946105050000	1.147622470000
H	-7.573203940000	-1.380913890000	-2.143271510000
H	-7.513040180000	-0.925693580000	2.101208950000
C	-7.721299520000	-1.178554290000	-0.013903180000
C	0.670378590000	-4.928826770000	-0.021138610000
C	0.791383770000	-5.606610750000	1.197907190000
C	0.756135010000	-5.632335180000	-1.231816250000
C	0.996842840000	-6.984218000000	1.184825870000
C	0.963369960000	-7.006197630000	-1.198847590000
H	1.088254440000	-7.507123310000	2.131244650000
H	1.030524940000	-7.546525940000	-2.137813840000
C	1.086703050000	-7.703919330000	-0.000160100000
C	-4.880843080000	-0.489556010000	2.406643360000
H	-5.532585340000	-0.619645160000	3.268268240000
H	-4.029879220000	-1.163222350000	2.522646090000
H	-4.477507350000	0.524585310000	2.448714790000
C	-4.948399820000	-1.043833480000	-2.602601040000
H	-4.333961230000	-0.154603530000	-2.757215290000
H	-4.271432810000	-1.898775070000	-2.671804170000
H	-5.653956820000	-1.115029990000	-3.428158050000
C	-9.198917830000	-1.399994870000	0.024185560000
H	-9.462656960000	-2.392145110000	-0.346358890000
H	-9.593246200000	-1.308075180000	1.034481610000
H	-9.726292820000	-0.683542660000	-0.607456890000
C	1.309298200000	-9.181861690000	0.003450730000
H	0.500678310000	-9.708247760000	-0.506220680000
H	2.229503630000	-9.447538010000	-0.519521330000

H	1.376739890000	-9.575687280000	1.015852040000
C	0.701640200000	-4.869976770000	2.496678490000
H	0.771787050000	-5.551427000000	3.342036910000
H	1.499691780000	-4.131455070000	2.597766130000
H	-0.237432520000	-4.320341790000	2.585550790000
C	0.623565000000	-4.919710750000	-2.540330710000
H	0.756171300000	-5.602666770000	-3.377078220000
H	-0.358321720000	-4.452559260000	-2.644534200000
H	1.359570850000	-4.118800240000	-2.640773670000
C	4.772148010000	0.770913930000	2.606736020000
H	4.035669120000	1.571726560000	2.699609610000
H	4.221679440000	-0.165662500000	2.714590210000
H	5.459237430000	0.853532030000	3.446417980000
C	4.790602440000	0.600744530000	-2.428855490000
H	5.460918780000	0.755170250000	-3.271956650000
H	4.358562640000	-0.397703380000	-2.527751020000
H	3.961520460000	1.304734710000	-2.524404830000
C	9.067733900000	1.337004810000	0.075629870000
H	9.591025070000	0.523516320000	-0.429408670000
H	9.328716320000	2.251889410000	-0.458965910000
H	9.469231420000	1.415676190000	1.084203240000
C	-0.918173650000	4.790562370000	2.558702960000
H	-1.696167440000	4.026119480000	2.607132530000
H	0.031292480000	4.271085010000	2.702650690000
H	-1.060031100000	5.464065500000	3.401446860000
C	-1.383022470000	9.118583320000	0.062776150000
H	-1.465949940000	9.511839610000	1.074262820000
H	-0.559887900000	9.637267880000	-0.431126390000
H	-2.290714160000	9.394359340000	-0.476760990000
C	-0.660501480000	4.855279950000	-2.470538460000
H	0.274730690000	4.293198500000	-2.515742890000
H	-1.460488870000	4.129252070000	-2.626798930000
H	-0.674609360000	5.551079690000	-3.306997550000
C	0.357572390000	-0.940033470000	-2.754453090000
C	-0.727517230000	-1.140738290000	-3.752452630000
C	-1.710674610000	-0.245473980000	-4.210186600000
H	-0.684043590000	-2.104452830000	-4.252942870000
H	1.301106280000	-0.748416440000	-3.302550950000
H	0.520532930000	-1.901867810000	-2.246540070000
H	-1.252059490000	1.437587430000	-2.935000130000
C	-1.900433830000	1.074933780000	-3.721302430000
C	-2.893079430000	1.884435080000	-4.236310230000
H	-3.018189260000	2.885684700000	-3.841175580000
H	-4.510623580000	2.079988270000	-5.644544250000
C	-3.735991450000	1.434917880000	-5.250142360000
C	-3.568874640000	0.144034200000	-5.749788200000
C	-2.586813650000	-0.676859530000	-5.243447690000
H	-2.464631260000	-1.680952780000	-5.634117880000
H	-4.216543430000	-0.218490720000	-6.538946660000

131

$^{\text{BS},3}$**MECP(A)** [triplet] E_{tot}(UM06L-D3(Dichloromethane)/def2TZVP/W06) = -2940.20921745 $\langle S^2 \rangle$ = 2.0386

Ru	-0.080900200000	-0.034609070000	0.099160000000
O	-0.018821980000	-0.103880260000	1.820383980000
O	0.145382460000	0.094224050000	-1.854804150000
C	3.339573200000	0.475450710000	0.088517430000
C	-0.570889240000	3.383145710000	0.036226560000
C	-3.468735140000	-0.546445730000	-0.128969110000
C	0.452731340000	-3.453182370000	-0.029977100000
N	-1.707364270000	1.185375090000	-0.036958440000
N	1.157228760000	1.633203730000	0.102103660000
N	1.595791840000	-1.272447570000	0.031596880000
N	-1.279057540000	-1.684552730000	-0.096012480000
C	1.590366130000	-2.635093240000	0.018203440000
C	2.893811250000	-0.852016810000	0.057774250000
C	2.522186270000	1.611202520000	0.108163760000
C	0.754356250000	2.932610480000	0.090340400000
C	-0.864815950000	-2.994569520000	-0.092486910000
C	-2.646742630000	-1.677452280000	-0.138920920000
C	-3.013430060000	0.775427430000	-0.108527440000
C	-1.695945460000	2.559838560000	-0.035054700000
C	2.944584500000	-3.101386720000	0.032951880000
C	3.746059520000	-2.004827300000	0.054269480000
C	3.000169500000	2.964402590000	0.116052790000
C	1.913613690000	3.776899300000	0.108563030000
C	-3.046124580000	3.024638480000	-0.115512820000
C	-3.852761160000	1.932629230000	-0.157916490000

C	-2.020533540000	-3.837278420000	-0.151363790000
C	-3.112031470000	-3.031070630000	-0.176394440000
H	3.239728660000	-4.138815220000	0.025218790000
H	4.824192810000	-1.973832110000	0.069419690000
H	4.040396870000	3.249614570000	0.123360240000
H	1.891874240000	4.855265520000	0.105197230000
H	-3.336966340000	4.063095040000	-0.137926110000
H	-4.929209430000	1.907568420000	-0.222161780000
H	-1.993636540000	-4.915352340000	-0.168735730000
H	-4.149441150000	-3.323125240000	-0.218899580000
C	4.815063860000	0.696929130000	0.089642120000
C	5.500450240000	0.838407430000	1.301915120000
C	5.510470860000	0.760919850000	-1.127211250000
C	6.877940970000	1.043532180000	1.276576340000
C	6.884532350000	0.969332270000	-1.106304340000
H	7.406890880000	1.151379640000	2.217909100000
H	7.418970770000	1.020747840000	-2.049643710000
C	7.589923640000	1.113334580000	0.085533910000
C	-0.790475610000	4.858779860000	0.044128070000
C	-0.821522730000	5.568051450000	-1.165860080000
C	-0.951528910000	5.532801920000	1.260436310000
C	-1.013212350000	6.944197810000	-1.134821050000
C	-1.142370260000	6.912590730000	1.245513030000
H	-1.035399080000	7.489341050000	-2.073150150000
H	-1.266386700000	7.432308660000	2.189991710000
C	-1.176105170000	7.638322940000	0.061256940000
C	-4.944639340000	-0.767469370000	-0.099530470000
C	-5.654442960000	-1.004752860000	-1.285916890000
C	-5.618216130000	-0.739051870000	1.128842280000
C	-7.028584510000	-1.203408110000	-1.221254600000
C	-6.995571530000	-0.946105050000	1.147622470000
H	-7.573203940000	-1.380913890000	-2.143271510000
H	-7.513040180000	-0.925693580000	2.101208950000
C	-7.721299520000	-1.178554290000	-0.013903180000
C	0.670378590000	-4.928826770000	-0.021138610000
C	0.791383770000	-5.606610750000	1.197907190000
C	0.756135010000	-5.632335180000	-1.231816250000
C	0.996842840000	-6.984218000000	1.184825870000
C	0.963369960000	-7.006197630000	-1.198847590000
H	1.088254440000	-7.507123310000	2.131244650000
H	1.030524940000	-7.546525940000	-2.137813840000
C	1.086703050000	-7.703919330000	-0.000160100000
C	-4.880843080000	-0.489556010000	2.406643360000
H	-5.532585340000	-0.619645160000	3.268268240000
H	-4.029879220000	-1.163222350000	2.522646090000
H	-4.477507350000	0.524585310000	2.448714790000
C	-4.948399820000	-1.043833480000	-2.602601040000
H	-4.333961230000	-0.154603530000	-2.757215290000
H	-4.271432810000	-1.898775070000	-2.671804170000
H	-5.653956820000	-1.115029990000	-3.428158050000
C	-9.198917830000	-1.399994870000	0.024185560000
H	-9.462656960000	-2.392145110000	-0.346358890000
H	-9.593246200000	-1.308075180000	1.034481610000
H	-9.726292820000	-0.683542660000	-0.607456890000
C	1.309298200000	-9.181861690000	0.003450730000
H	0.500678310000	-9.708247760000	-0.506220680000
H	2.229503630000	-9.447538010000	-0.519521330000
H	1.376739890000	-9.575687280000	1.015852040000
C	0.701640200000	-4.869976770000	2.496678490000
H	0.771787050000	-5.551427000000	3.342036910000
H	1.499691780000	-4.131455070000	2.597766130000
H	-0.237432520000	-4.320341790000	2.585550790000
C	0.623565000000	-4.919710750000	-2.540330710000
H	0.756171300000	-5.602666770000	-3.377078220000
H	-0.358321720000	-4.452559260000	-2.644534200000
H	1.359570850000	-4.118800240000	-2.640773670000
C	4.772148010000	0.770913930000	2.606736020000
H	4.035669120000	1.571726560000	2.699609610000
H	4.221679440000	-0.165662500000	2.714590210000
H	5.459237430000	0.853532030000	3.446417980000
C	4.790602440000	0.600744530000	-2.428855490000
H	5.460918780000	0.755170250000	-3.271956650000
H	4.358562640000	-0.397703380000	-2.527751020000
H	3.961520460000	1.304734710000	-2.524404830000
C	9.067733900000	1.337004810000	0.075629870000
H	9.591025070000	0.523516320000	-0.429408670000
H	9.328716320000	2.251889410000	-0.458965910000

```
H        9.469231420000     1.415676190000     1.084203240000
C       -0.918173650000     4.790562370000     2.558702960000
H       -1.696167440000     4.026119480000     2.607132530000
H        0.031292480000     4.271085010000     2.702650690000
H       -1.060031100000     5.464065500000     3.401446860000
C       -1.383022470000     9.118583320000     0.062776150000
H       -1.465949940000     9.511839610000     1.074262820000
H       -0.559887900000     9.637267880000    -0.431126390000
H       -2.290714160000     9.394359340000    -0.476760990000
C       -0.660501480000     4.855279950000    -2.470538460000
H        0.274730690000     4.293198500000    -2.515742890000
H       -1.460488870000     4.129252070000    -2.626798930000
H       -0.674609360000     5.551079690000    -3.306997550000
C        0.357572390000    -0.940033470000    -2.754453090000
C       -0.727517230000    -1.140738290000    -3.752452630000
C       -1.710674610000    -0.245473980000    -4.210186600000
H       -0.684043590000    -2.104452830000    -4.252942870000
H        1.301106280000    -0.748416440000    -3.302550950000
H        0.520532930000    -1.901867810000    -2.246540070000
H       -1.252059490000     1.437587430000    -2.935000130000
C       -1.900433830000     1.074933780000    -3.721302430000
C       -2.893079430000     1.884435080000    -4.236310230000
H       -3.018189260000     2.885684700000    -3.841175580000
H       -4.510623580000     2.079988270000    -5.644544250000
C       -3.735991450000     1.434917880000    -5.250142360000
C       -3.568874640000     0.144034200000    -5.749788200000
C       -2.586813650000    -0.676859530000    -5.243447690000
H       -2.464631260000    -1.680952780000    -5.634117880000
H       -4.216543430000    -0.218490720000    -6.538946660000
```

131

^{BS}TS(A) E_{tot}(UM06L-D3(Dichloromethane)/def2TZVP/W06) = -2940.18740747 $\langle S^2 \rangle$ = 0.2678

```
Ru      -0.091329055288    -0.009453531119     0.092150491698
O        0.042426563993    -0.054994596631     1.824307389195
O        0.131047363587     0.240531291576    -1.694816126545
C        3.331023183016     0.539815208336     0.120992308407
C       -0.602404075332     3.408924964526     0.128078083407
C       -3.476794492937    -0.537268973354     0.017630724183
C        0.476379160322    -3.406952309818    -0.138475605133
N       -1.736351292226     1.208563344954     0.076323091792
N        1.148670276584     1.685320394694     0.188509357648
N        1.603771205015    -1.225422714405     0.004333094377
N       -1.273458129554    -1.652902938764    -0.095018359369
C        1.609372791129    -2.584451657161    -0.058597628093
C        2.896259643596    -0.790698687713     0.051898078145
C        2.509059924860     1.673101445310     0.174828034704
C        0.730313937663     2.976475880546     0.171799735698
C       -0.846275838027    -2.957000211602    -0.169042393128
C       -2.645007797494    -1.656134723363    -0.090328545935
C       -3.036623701081     0.790406474244     0.063423871648
C       -1.726948916682     2.578616804204     0.100964242816
C        2.969150814249    -3.038880315672    -0.050639940993
C        3.759656787754    -1.936307008975     0.012839289215
C        2.977445934345     3.033120130254     0.177563790323
C        1.883672925223     3.834790932713     0.172437147000
C       -3.083636005588     3.037842357272     0.102021489279
C       -3.886253556329     1.942259839640     0.090977879907
C       -1.996882449923    -3.805208007634    -0.243009317220
C       -3.096775291338    -3.010125994863    -0.189068900144
H        3.275258148529    -4.072367724308    -0.092772192693
H        4.837408402832    -1.896628882358     0.035386462145
H        4.015042288375     3.328325214073     0.175532840910
H        1.851836911892     4.913040069130     0.166208852911
H       -3.380909267717     4.074625546852     0.115302649341
H       -4.964458626334     1.911979296036     0.090262795723
H       -1.962398345383    -4.880653181553    -0.319071585742
H       -4.132200084701    -3.311537336353    -0.214732807981
C        4.804686224644     0.770682669062     0.102148975344
C        5.510092105425     0.902776358616     1.303571094376
C        5.477534656075     0.854404040114    -1.126267769818
C        6.885834275439     1.115949522129     1.256346725806
C        6.850596293937     1.070095081221    -1.127342042506
H        7.430979830691     1.215958304935     2.189299167011
H        7.367875647623     1.136916471687    -2.079258744890
C        7.576182322881     1.203290032542     0.053752096367
C       -0.840823868282     4.879997519816     0.069148413751
C       -0.995484706792     5.502789825245    -1.179052838862
```

C	-0.909215977454	5.633133604668	1.246731013854
C	-1.215739807424	6.874438019337	-1.222914911821
C	-1.134021012437	7.004829567550	1.156344259103
H	-1.332961033849	7.353483367813	-2.189833259589
H	-1.190092804996	7.586092886976	2.070973055711
C	-1.289534584434	7.646241863509	-0.066296364156
C	-4.945425949429	-0.774738876936	0.139636608031
C	-5.763994159481	-0.812443741630	-0.997747135442
C	-5.502123545844	-0.953104463765	1.412950044328
C	-7.128612398550	-1.025006804039	-0.840673649264
C	-6.874250421648	-1.164721045502	1.525003591602
H	-7.757122900201	-1.054162998630	-1.725111683780
H	-7.301482635359	-1.302766831848	2.512892304306
C	-7.706723936378	-1.202933389480	0.413280042169
C	0.704290453301	-4.881638249970	-0.160556212228
C	0.715789351147	-5.597071068621	1.043548800738
C	0.914949540071	-5.546624964701	-1.377238240052
C	0.938602960712	-6.971579579255	1.008283034730
C	1.132548725389	-6.919417592019	-1.366825604719
H	0.949755007570	-7.522295752258	1.943211431706
H	1.295027654684	-7.428986464321	-2.311317073689
C	1.148830610903	-7.653420631331	-0.183872191251
C	-4.644789467436	-0.918642630362	2.638553666690
H	-5.238134619354	-1.067928436610	3.538386610186
H	-3.874012807563	-1.691767144042	2.615164150059
H	-4.119745756353	0.033462667087	2.738611090448
C	-5.175632272629	-0.639281712604	-2.359991997037
H	-4.669049415477	0.322482575603	-2.467410270551
H	-4.423689989178	-1.403449162278	-2.565913533494
H	-5.937480580849	-0.703685787112	-3.134623271983
C	-9.178947182149	-1.420367274645	0.552009638343
H	-9.520517981992	-2.252835545556	-0.064648813976
H	-9.459192731329	-1.629607834718	1.582676780810
H	-9.742167245306	-0.543174360343	0.228079104700
C	1.378119093498	-9.130211883987	-0.203922416682
H	0.564631399394	-9.651592333819	-0.711896611819
H	2.292717600419	-9.386128629672	-0.740465646610
H	1.453938946046	-9.538854901920	0.802001269988
C	0.493428417186	-4.904482589258	2.350754277184
H	0.576091327628	-5.599661854659	3.183730803928
H	1.215009859882	-4.101211495910	2.511685599325
H	-0.494930652920	-4.442634089967	2.399777592296
C	0.906529327402	-4.793233092486	-2.668922370973
H	1.054434988782	-5.459156853161	-3.516716812744
H	-0.036806344841	-4.264374271644	-2.819316169199
H	1.693381554889	-4.036243589047	-2.699030567139
C	4.803841421874	0.817200807669	2.619382373100
H	4.069586138004	1.617106531026	2.735321410830
H	4.253896826777	-0.120134773374	2.722904973296
H	5.504673887433	0.887802683078	3.448780257090
C	4.732378986009	0.715471689319	-2.416506010529
H	5.392345925529	0.856049647716	-3.270171750682
H	4.270354926757	-0.269967102256	-2.512279259866
H	3.921226929872	1.442449074276	-2.495511407653
C	9.052725435630	1.433298219276	0.020415258805
H	9.572023702249	0.618162505850	-0.486288882296
H	9.302137491867	2.345214533730	-0.524542250784
H	9.468717381756	1.520345077805	1.022413710279
C	-0.747241505860	4.981075441335	2.583094418104
H	-1.472529324916	4.178841210116	2.731407835180
H	0.239363757353	4.526409397917	2.693029181731
H	-0.872933377316	5.699478063739	3.390661620190
C	-1.523271703073	9.120422237602	-0.146067183703
H	-1.614814956530	9.566965259897	0.842298794163
H	-0.705495538135	9.625370970590	-0.663418679418
H	-2.431995763395	9.351669851971	-0.703712770304
C	-0.928102115029	4.706878582850	-2.444724381197
H	-0.001785255166	4.132475766610	-2.514176448849
H	-1.742030903835	3.981075666789	-2.509035121624
H	-0.990193380135	5.351409855996	-3.319502691789
C	0.360258911767	-0.977133256351	-3.033030464736
C	-0.905294868918	-1.365685368157	-3.447593068335
C	-1.753144893363	-0.565006254857	-4.276343404215
H	-1.316063801098	-2.299174351639	-3.074098820599
H	0.919532060091	-0.283737801718	-3.647748749222
H	0.974827169713	-1.703155664357	-2.514948757492
H	-0.651894941985	1.268824362028	-4.003405928870

C	-1.499869821064	0.804342732484	-4.492858993793
C	-2.341598481546	1.562549701593	-5.283230531410
H	-2.138587303009	2.617169129521	-5.424018806216
H	-4.104646623080	1.579739627029	-6.513344457320
C	-3.450096706274	0.982164913027	-5.891901389245
C	-3.716649452444	-0.367841902111	-5.690547258956
C	-2.889426873850	-1.127179715455	-4.886724354053
H	-3.104607020504	-2.176680271946	-4.720524381461
H	-4.580939064876	-0.824265927133	-6.157055964264

131

BSE E_{tot}(UM06L-D3(Dichloromethane)/def2TZVP/W06) = -2940.21039174 $\langle S^2 \rangle$ = 1.0244

Ru	-0.060656335357	-0.043272837722	0.202256382402
O	-0.140704719967	-0.080544280127	1.931022740478
O	-0.044716272108	0.022820118995	-1.794553803434
C	3.340210506894	0.470328398717	0.089243632053
C	-0.579803218547	3.371808395822	0.095799217988
C	-3.464839295754	-0.558486155773	-0.049438428641
C	0.454184430454	-3.463336196404	0.088865192486
N	-1.717115270229	1.184201993112	0.054076922815
N	1.152853429540	1.613789559701	0.141719372117
N	1.588822781735	-1.269665558225	0.112792114293
N	-1.278293735445	-1.705219776674	0.041915832404
C	1.582968213273	-2.639545392495	0.095448473954
C	2.891593119009	-0.854524152982	0.087308111194
C	2.519571916732	1.602350062971	0.116215449438
C	0.742018290145	2.918433273713	0.127936998094
C	-0.870492468156	-3.011268071931	0.061222495073
C	-2.643827397682	-1.691479411644	-0.014783889185
C	-3.015872707303	0.767141641490	-0.031782555343
C	-1.710729577941	2.549943097014	0.042591905189
C	2.937186362355	-3.103235409135	0.061950090169
C	3.740002444422	-2.008184714253	0.054663785093
C	2.989527864709	2.956066423641	0.099688976644
C	1.898794080869	3.763559793130	0.112131546433
C	-3.062767216986	3.015900756035	-0.052314053100
C	-3.863851998332	1.921056687095	-0.101659781578
C	-2.026191453992	-3.854920987111	0.018156833900
C	-3.114938356610	-3.044821491255	-0.028117551683
H	3.231356747511	-4.140842655393	0.043020954984
H	4.818009142377	-1.978520855823	0.029244427156
H	4.028198582527	3.246428211760	0.079205124864
H	1.873188684750	4.841806537452	0.100671767300
H	-3.355656564753	4.053628336156	-0.084954717478
H	-4.939290074979	1.890531374825	-0.180281325554
H	-2.002421745169	-4.933249253872	0.019801406755
H	-4.153178029868	-3.334393642148	-0.069340557788
C	4.814472588047	0.692988835611	0.037776028811
C	5.540582131444	0.857683187618	1.223165252427
C	5.469050060854	0.734433183420	-1.202676895479
C	6.916221557619	1.062891216047	1.147697737214
C	6.842941383628	0.943380561645	-1.232107951838
H	7.476523927903	1.189031833526	2.068429773423
H	7.345271260793	0.977496900310	-2.193710096592
C	7.587872437541	1.110110022074	-0.067643723856
C	-0.796730354351	4.847909478991	0.096777308919
C	-0.874201755386	5.546047877433	-1.117575386236
C	-0.913408682023	5.533441344739	1.311758554298
C	-1.064526202734	6.922563549965	-1.092291005279
C	-1.105163369491	6.913073372149	1.291324348568
H	-1.122056481920	7.458920470272	-2.034208484938
H	-1.195332950265	7.441481256564	2.234850367668
C	-1.182452963866	7.627920457451	0.102495423862
C	-4.939814897304	-0.785335910272	-0.082195527093
C	-5.590117217779	-1.056967418884	-1.295910747081
C	-5.672205883372	-0.733136954852	1.110906800145
C	-6.964638706971	-1.264471861499	-1.291765626598
C	-7.047572993437	-0.950168557989	1.068869180511
H	-7.463217103541	-1.469270039956	-2.233953572445
H	-7.610122926880	-0.912537907107	1.996067585974
C	-7.714968809861	-1.215898090220	-0.120088826905
C	0.676383869561	-4.938252692425	0.080109211669
C	0.818142709868	-5.629253189046	1.289235518112
C	0.739706072658	-5.629436033224	-1.139429339359
C	1.021120577368	-7.007003143639	1.258348272220
C	0.944927871891	-7.003991536080	-1.124411158698
H	1.128962876739	-7.539880259394	2.197467418318

```
H     0.994414902144   -7.534784093083   -2.069907894202
C     1.088087577130   -7.714500944991    0.064541225864
C    -4.998155998075   -0.450953716148    2.416601272468
H    -5.691047735185   -0.561055102688    3.248436625008
H    -4.153574027080   -1.120268870535    2.590349134794
H    -4.597314911750    0.564295428000    2.453411699625
C    -4.824422992394   -1.121065404629   -2.578828011931
H    -4.240253893292   -0.214275345483   -2.745771159922
H    -4.111428431019   -1.949197143505   -2.584360616966
H    -5.490202309275   -1.253293245882   -3.429769609963
C    -9.191644012247   -1.445281710336   -0.148454365897
H    -9.434517351311   -2.432121277273   -0.545985359645
H    -9.629347423398   -1.370257141679    0.845227554226
H    -9.695386151977   -0.720562019682   -0.790191655268
C     1.307630041783   -9.192857653965    0.048410602360
H     0.489442131316   -9.712186169096   -0.453173678893
H     2.218345443561   -9.454719399750   -0.492805583770
H     1.391324335364   -9.597841081349    1.055171689510
C     0.753592627396   -4.905707894167    2.596801907368
H     0.841027817982   -5.595405203122    3.433852192889
H     1.552767070592   -4.167354696878    2.688835939079
H    -0.183841601836   -4.357834073559    2.709954766193
C     0.588515079426   -4.904118262560   -2.439518223014
H     0.697757098449   -5.582000213091   -3.283790261046
H    -0.390071055941   -4.424756561903   -2.522746489562
H     1.331536442047   -4.110805380979   -2.548488608972
C     4.856836746793    0.815619981610    2.552883448074
H     4.125917274699    1.620225081719    2.656055408238
H     4.308112974681   -0.117139545282    2.695998155637
H     5.572113647091    0.911317565034    3.367313939715
C     4.706258377402    0.548358820863   -2.476323293697
H     5.343799540971    0.704014108085   -3.344327757417
H     4.288095680163   -0.458352070241   -2.549411672501
H     3.862341989713    1.237131547547   -2.549695673314
C     9.064311600989    1.334212843099   -0.131295932179
H     9.571673853959    0.510680362349   -0.636356163536
H     9.306407832397    2.237922512054   -0.693096223262
H     9.498813232845    1.434103506644    0.861614023311
C    -0.831833316004    4.803157510483    2.614675539064
H    -1.593754263401    4.024921344555    2.690401153352
H     0.130552520243    4.302151518804    2.736926811169
H    -0.962508414744    5.481328694166    3.455527538594
C    -1.385953272771    9.108697266165    0.097964173053
H    -1.459109126436    9.507491334833    1.108041644983
H    -0.564802197105    9.622491153764   -0.404585451750
H    -2.296656770099    9.384630369552   -0.436180215027
C    -0.766438355510    4.820979868486   -2.421014780953
H     0.151510850288    4.233338803460   -2.487291823146
H    -1.589694740161    4.115868762671   -2.551199346018
H    -0.785344811409    5.511571931317   -3.261774247266
C     0.111930511337   -1.034711188472   -2.650068028870
C    -0.656073380331   -1.016215250197   -3.909125462800
C    -1.614226656076   -0.102800496640   -4.375595646393
H    -0.452936039366   -1.872453270484   -4.547619037345
H     1.184562596705   -1.138478761942   -2.919320740821
H    -0.101392743062   -2.006079837752   -2.163637646822
H    -1.464097681307    1.364814985685   -2.796310115828
C    -1.975710282363    1.101071404340   -3.711341219510
C    -2.964218622556    1.916064947767   -4.224526376236
H    -3.228728802157    2.825419754857   -3.698327306510
H    -4.400494237498    2.236046675428   -5.794464915321
C    -3.627306921540    1.586962284251   -5.403785430860
C    -3.281596804942    0.418079605030   -6.081421517905
C    -2.297827065240   -0.404901532217   -5.585110313351
H    -2.032656255052   -1.314610632375   -6.111395983888
H    -3.788950998411    0.156267307423   -7.001673052233
```

131

^{3}TS(E,F) E_{tot}(UM06L-D3(Dichloromethane)/def2TZVP/W06) = -2940.21258830 $\langle S^2 \rangle$ = 2.0524

```
Ru   -0.077601176006   -0.198016402609    0.067095180081
O    -0.069327950447   -0.163880880935    1.812367674134
O     0.044192320617   -0.587628373532   -1.968314167606
C     3.323195546021    0.366856635233   -0.098057733517
C    -0.666393325539    3.167483069142   -0.320334855224
C    -3.480875431909   -0.801851741865    0.101028688329
C     0.510048898743   -3.600623051910    0.168697640324
N    -1.757517691317    0.958193833867   -0.140550581528
```

N	1.104275799671	1.452817243595	-0.182684102614
N	1.616861837303	-1.396855872080	0.066133729099
N	-1.273588761312	-1.894665603917	0.105938189705
C	1.628752736801	-2.760124114784	0.117966227412
C	2.906114602025	-0.966245189179	-0.000173958558
C	2.474828488146	1.474028010129	-0.177865975983
C	0.666804971323	2.745746948779	-0.281705972149
C	-0.826535274084	-3.183928590327	0.165056822516
C	-2.636021557512	-1.918686791280	0.151233497123
C	-3.057438621138	0.521519162806	-0.057483842835
C	-1.781387714826	2.321780263626	-0.264243606913
C	2.992499507766	-3.209491841008	0.092233205913
C	3.778519411868	-2.106008188194	0.021144604098
C	2.913680073864	2.834788701503	-0.271194473322
C	1.805339308355	3.615013146255	-0.329520694573
C	-3.145802057913	2.756607227224	-0.292248737862
C	-3.927676642687	1.655011578923	-0.152837398863
C	-1.960499964396	-4.060349388728	0.235130304589
C	-3.073195179825	-3.282469880334	0.230698544376
H	3.302743624313	-4.242238315584	0.122550171615
H	4.855606208393	-2.060704825462	-0.019962972746
H	3.945442487937	3.149111667157	-0.287929053461
H	1.757288042012	4.690180788379	-0.401186454038
H	-3.463000226745	3.782333224840	-0.398189938452
H	-5.005088069331	1.608657746162	-0.122915292031
H	-1.906172456147	-5.136681615980	0.284544577810
H	-4.103399026637	-3.599371746300	0.275117208277
C	4.792111955511	0.622620510664	-0.140608653665
C	5.514419279263	0.749912933978	1.053430436763
C	5.447517953085	0.725471294740	-1.375258401989
C	6.884893724608	0.982322979746	0.990367398536
C	6.819726903994	0.956413011695	-1.392529403729
H	7.440868772624	1.078866025948	1.917424923870
H	7.324753167945	1.029260523932	-2.350385779609
C	7.558491552224	1.090891393181	-0.221677865390
C	-0.921836111699	4.634936097670	-0.404548768641
C	-0.753093241204	5.313418456176	-1.618548891702
C	-1.326253287129	5.339531699518	0.742198732241
C	-1.017793089404	6.680433803187	-1.672602985770
C	-1.571619616069	6.703794826934	0.644059796601
H	-0.895375066376	7.198059548668	-2.618576289879
H	-1.878720510232	7.243395172566	1.534465175018
C	-1.430689764435	7.395390435481	-0.556249445841
C	-4.944800356655	-1.046334420706	0.253752696098
C	-5.782141151854	-1.115241047092	-0.865588543739
C	-5.479177829067	-1.212077469769	1.542449921543
C	-7.142178915153	-1.355541858904	-0.679325270094
C	-6.841732987288	-1.444967832713	1.682745099844
H	-7.785574048208	-1.415233523807	-1.551180851009
H	-7.249515963305	-1.568687342236	2.681027914815
C	-7.693808230464	-1.523218179085	0.583980419867
C	0.767127798681	-5.069620082838	0.224987881142
C	0.989652052587	-5.695148068724	1.461014043471
C	0.793012409976	-5.819196818942	-0.957188258909
C	1.235322169965	-7.063091896452	1.488819516137
C	1.046148717378	-7.187056571058	-0.883945293584
H	1.406370652789	-7.542898805344	2.447342633173
H	1.071369299302	-7.763560183308	-1.803023641631
C	1.269552075882	-7.829946892585	0.327188489903
C	-4.601046061419	-1.140061912575	2.751810132758
H	-5.190007733929	-1.157018219760	3.666730279483
H	-3.902049237302	-1.978319895111	2.793655131149
H	-3.991829720000	-0.234366227435	2.757836921741
C	-5.245438812584	-0.913481103251	-2.247109044419
H	-5.082563653079	0.147164317250	-2.456142511960
H	-4.285579831331	-1.411918353031	-2.393287480622
H	-5.938976902080	-1.285363267572	-2.999379979807
C	-9.155401354967	-1.776086148433	0.767719204490
H	-9.334882813139	-2.703682438650	1.313425936714
H	-9.627109983553	-0.980247821353	1.346757845583
H	-9.675057194539	-1.844323700805	-0.186219316565
C	1.531213687100	-9.300301042836	0.389708569136
H	1.615899302277	-9.736106751013	-0.604109627553
H	2.451288694147	-9.520786072687	0.932647364044
H	0.729409785536	-9.824641956184	0.913029260872
C	0.966292620554	-4.906217149492	2.731718458708
H	1.097952402609	-5.550982600743	3.598379423386

```
H        1.757137923913    -4.153582086383     2.753708164796
H        0.026144716032    -4.365174293099     2.854411808608
C        0.543996059614    -5.170858291362    -2.282413113986
H        0.744967193111    -5.857965183245    -3.102104211902
H       -0.493562330942    -4.841780236069    -2.375750327876
H        1.163018767190    -4.282981326018    -2.424701509654
C        4.828632220898     0.635667302818     2.377905705213
H        4.014149625407     1.356038843643     2.474492891500
H        4.382208127969    -0.351020447697     2.517342164300
H        5.523880631131     0.804052251907     3.197915525955
C        4.689318257318     0.586261853256    -2.657662634147
H        5.355963493502     0.630074473536    -3.516689729812
H        4.141920987286    -0.357347129732    -2.703904312160
H        3.945051075203     1.377275390791    -2.773750667090
C        9.025954859707     1.371816972666    -0.264535752886
H        9.490768043767     0.954509031787    -1.156802966681
H        9.220551609478     2.446784976139    -0.278993099352
H        9.539147842487     0.969794548122     0.608021538037
C       -1.485628588164     4.645984813512     2.058690484851
H       -2.361734622405     3.994134314834     2.071022569483
H       -0.628558962558     4.011126810223     2.289205683606
H       -1.602037553371     5.364389813457     2.867906836932
C       -1.712016535517     8.861317994517    -0.631455298824
H       -1.082336159904     9.424431600689     0.059280865803
H       -1.540605927793     9.252433851860    -1.632616461645
H       -2.745232807196     9.083718642812    -0.359451814534
C       -0.274129939781     4.601989461528    -2.843637513544
H        0.809348532364     4.460005751052    -2.825087902895
H       -0.712222452499     3.607961705114    -2.934645452592
H       -0.509112343309     5.166045980347    -3.744510179837
C       -1.022764027147    -1.012716708105    -2.762884289385
C       -0.723567318725    -0.098700529539    -3.875073702189
C       -1.404131624938     1.087738256907    -4.205584459878
H        0.170051512568    -0.334246259859    -4.442962508365
H       -0.950960787511    -2.075165801852    -3.031420350454
H       -2.007131958769    -0.845768571495    -2.310445496428
H       -2.921424064109     0.960064444742    -2.670943348325
C       -2.573364973028     1.512577827951    -3.534188700928
C       -3.270604610242     2.619020007826    -3.967752347168
H       -4.165997599857     2.928159254827    -3.442466859908
H       -3.384363903234     4.203127912453    -5.415547391766
C       -2.829522749183     3.337584481016    -5.076617620676
C       -1.668258527728     2.950758959957    -5.741536909346
C       -0.957195182416     1.853473150403    -5.308843167541
H       -0.054967799390     1.544609323418    -5.822790242864
H       -1.321673378184     3.516245649899    -6.596882648550
```

131

^{3}TS(F,D) E_{tot} (UM06L-D3(Dichloromethane)/def2TZVP/W06) = -2940.23389323 $\langle S^2 \rangle$ = 2.0132

```
Ru       0.174029673304    -0.055975868405     0.379427049617
O        0.065799331092     0.040379402723     2.113488137652
C        3.620244166071    -0.165207226386     0.246086113938
C        0.281508127427     3.377146998601     0.025648888285
C       -3.251293340965     0.020304545424    -0.159592989527
C        0.075302404093    -3.511264054061     0.277987999249
N       -1.214870684095     1.414569401012    -0.020643570622
N        1.663865452255     1.336151099752     0.159938402677
N        1.578322715616    -1.549835550220     0.287124047117
N       -1.311516267889    -1.475148097011     0.137956572718
C        1.331254360784    -2.899664468496     0.295460562041
C        2.939884334807    -1.384313539742     0.272189279722
C        3.012306510556     1.090878855819     0.198211006019
C        1.498367414736     2.697444648119     0.112208828667
C       -1.144397224519    -2.835721377451     0.191438226210
C       -2.652590346050    -1.233362220905    -0.014309626191
C       -2.568750834489     1.239237219408    -0.174789176689
C       -0.968686328658     2.762399338924    -0.085142735837
C        2.578976037990    -3.601519936384     0.292203348291
C        3.566582067221    -2.671153839514     0.274609714567
C        3.715021342743     2.337594188470     0.174532349970
C        2.785155895359     3.324657886888     0.133260090323
C       -2.203125024701     3.453393900378    -0.300234201559
C       -3.184918423958     2.517630233845    -0.361947389609
C       -2.425221863030    -3.466660729390     0.090518090916
C       -3.351453176274    -2.482506371647    -0.038356668940
H        2.680596639544    -4.675411281844     0.291156112604
H        4.632321997630    -2.837106481548     0.258173481021
```

H	4.788822685116	2.439187332855	0.186158324585
H	2.951191156142	4.389976137079	0.102200524948
H	-2.300192693963	4.522997556835	-0.401722568023
H	-4.240150594275	2.674967126969	-0.521527034955
H	-2.589745219393	-4.532709646125	0.103674963284
H	-4.419060981624	-2.589011719551	-0.150075846340
C	5.111323396026	-0.207016950239	0.249051839369
C	5.807435055930	-0.248618226377	1.462948572841
C	5.811649361545	-0.203507521488	-0.966806109944
C	7.199711985664	-0.285646639254	1.440427232732
C	7.200957679403	-0.242241077024	-0.943204870682
H	7.736320870031	-0.316958910993	2.383115367611
H	7.739135692671	-0.241302526302	-1.885853909676
C	7.916858811236	-0.282723207044	0.250443029954
C	0.313747438311	4.867943604708	0.075387787713
C	0.602617106550	5.621678935610	-1.068539099450
C	0.050987693359	5.512280471790	1.295414293089
C	0.620462205909	7.011517990398	-0.973715746920
C	0.082034882250	6.900846880102	1.344793281799
H	0.840956045270	7.591705425261	-1.863866174216
H	-0.117521364498	7.393325658329	2.291297755351
C	0.363348265449	7.672462326209	0.220801618091
C	-4.731543475041	0.053930968926	-0.343606384068
C	-5.276981647560	-0.076447900825	-1.626582400693
C	-5.573336430123	0.208146837242	0.767794322712
C	-6.661215160365	-0.048657741814	-1.777893114671
C	-6.949398502847	0.229369203547	0.572046327391
H	-7.079580881482	-0.148200956981	-2.774168736779
H	-7.597212408311	0.347363522759	1.434885438656
C	-7.516385595122	0.102633712416	-0.693349316163
C	0.036295195817	-5.002808224603	0.309087899753
C	0.001206074878	-5.674349024652	1.540378283627
C	0.036138715070	-5.729168789161	-0.888049297920
C	-0.033699700846	-7.063999608737	1.549005268832
C	0.002164489361	-7.120520298415	-0.833439210690
H	-0.061950272561	-7.579025068395	2.504022274603
H	0.004052078869	-7.679611611773	-1.763524546944
C	-0.032917984720	-7.808958032045	0.372783304625
C	-5.003034204316	0.345395477603	2.143651404433
H	-5.790027160600	0.427306298945	2.890643848186
H	-4.377250672517	-0.509264766987	2.407755938763
H	-4.367015679584	1.228650402475	2.230447161944
C	-4.392952957605	-0.239717813610	-2.822483527431
H	-3.693436152225	0.593197046813	-2.919981503088
H	-3.785492200167	-1.145713217963	-2.758390329215
H	-4.974857099741	-0.294580856593	-3.740410122746
C	-9.000275448574	0.125733275022	-0.871078576494
H	-9.281016874306	0.047099752898	-1.919719007343
H	-9.478173074949	-0.697167988110	-0.336730095574
H	-9.435638591746	1.045099533430	-0.476521764934
C	-0.065174997613	-9.302726306419	0.413940920323
H	-0.934529499589	-9.668618309287	0.962355817347
H	-0.096869474727	-9.731475963514	-0.585999942769
H	0.813123909876	-9.706321638515	0.920718783933
C	0.000981304593	-4.910597928907	2.826504070228
H	-0.047918850448	-5.580207228970	3.682801822546
H	0.898815696676	-4.298364639359	2.931946762018
H	-0.845955271273	-4.224489465492	2.888399902343
C	0.067081377074	-5.031088121409	-2.210927414587
H	0.122370751075	-5.743911451326	-3.031312641257
H	-0.825413602770	-4.420036913600	-2.362999870349
H	0.922541158043	-4.357817967887	-2.293992081257
C	5.074117346588	-0.254014471865	2.766770809625
H	4.416779032685	0.612095800175	2.864193195836
H	4.436502176466	-1.134709055916	2.867106875200
H	5.764444018535	-0.246532029972	3.607879763133
C	5.079449777393	-0.164660008075	-2.271135588498
H	5.770671667691	-0.173572796409	-3.111531922849
H	4.407626564003	-1.018657488066	-2.380871364096
H	4.455697166772	0.727549448900	-2.360324869867
C	9.411253864243	-0.313295592766	0.244482011985
H	9.814661899196	-0.433497634795	1.248234119354
H	9.792778004250	-1.129297426181	-0.370746661492
H	9.825981870117	0.607506693645	-0.170009662007
C	-0.259072349032	4.724372801628	2.529320675033
H	-1.216692845449	4.205512329380	2.446723107907
H	0.490226551937	3.953610838961	2.718813784860

H	-0.308965857492	5.368904961790	3.404719811020
C	0.388630912419	9.164492828033	0.305923062363
H	-0.567402532755	9.560618516508	0.652053266879
H	1.141880036174	9.510462827511	1.015783478115
H	0.607787820332	9.618837530768	-0.658570429133
C	0.884622956712	4.957354878051	-2.378236458296
H	1.789777239723	4.347542278060	-2.337521428010
H	0.076296188460	4.285602141026	-2.674119334866
H	1.016525362237	5.691395669385	-3.170847838645
O	0.852734457448	-0.080415105040	-2.719030653879
H	-0.389655516541	-1.757109101918	-3.127789339869
C	0.088605053252	-0.896928794448	-3.590373401546
H	0.537625419601	-1.074915872190	-4.563753733082
C	-0.366943471426	0.447731614235	-3.249547747346
H	-1.171317392079	0.518964516076	-2.518875967164
C	-0.251917388758	1.601583524341	-4.165722976690
H	1.808227298747	1.258726049859	-4.643813951222
C	0.942559001353	1.885219553728	-4.824575146640
H	1.960703116464	3.183474106443	-6.187958734680
C	1.025913188986	2.967514540503	-5.685725577210
C	-0.081952112726	3.776528058604	-5.902935446315
H	-0.013888074582	4.621931433120	-6.575835072645
C	-1.272201901231	3.504480687901	-5.243735173324
H	-2.136631026315	4.137909944180	-5.398226450321
C	-1.354024771705	2.427285898966	-4.374210383175
H	-2.279315065005	2.223604011031	-3.846634489525

7.4.3 Species related to microsolvation path

121

$^{1}\mathbf{A}_{\mathbf{w2}}$ E_{tot}(RM06L-D3(Dichloromethane)/def2TZVP/W06) = -2783.39890412 $\langle S^2 \rangle$ = 0.0000

Ru	0.161746848233	-0.028511708226	0.481783608902
O	0.331166736560	0.143006874868	2.195968317743
O	0.258044657954	0.163744985670	-1.235784606592
C	3.586499242280	0.546443387063	0.407486578031
C	-0.362248077982	3.404435462089	0.502826693664
C	-3.233583881584	-0.545785413238	0.556445134285
C	0.732286991016	-3.415600906210	0.463233945942
N	-1.500622333006	1.202820044653	0.526091944649
N	1.405096170184	1.689372783742	0.462357555940
N	1.851909194568	-1.223182578316	0.440298155089
N	-1.020406261032	-1.662639584990	0.515519409624
C	1.860490460131	-2.584571814846	0.447864351007
C	3.147521500256	-0.781778046561	0.425544699585
C	2.763298459986	1.682017573660	0.410895782715
C	0.973148228143	2.977944099584	0.453588436126
C	-0.590417757496	-2.972011791602	0.496776290956
C	-2.398501892163	-1.663632168968	0.538729241013
C	-2.796199043728	0.785565035054	0.562277311247
C	-1.485746195312	2.571739177160	0.540258969276
C	3.220565182509	-3.032441049808	0.439730428987
C	4.008795888696	-1.927333756080	0.427706869694
C	3.221250774679	3.042639705991	0.356336031030
C	2.120803566464	3.839312061959	0.382020049934
C	-2.842082427927	3.031627544309	0.593825310720
C	-3.645492428624	1.937521656110	0.605290354903
C	-1.740604965037	-3.817803860018	0.516629586355
C	-2.841955034198	-3.020803224693	0.541912439769
H	3.527780626326	-4.066156439436	0.443340420089
H	5.086394654235	-1.883990388989	0.419522992022
H	4.255829907481	3.343898739316	0.308678548823
H	2.083355580643	4.917062448065	0.360241159877
H	-3.136844452434	4.068756443089	0.617819121311
H	-4.722901238424	1.906751043732	0.640975305823
H	-1.706325024353	-4.895754109411	0.508383182337
H	-3.876558180764	-3.324881104390	0.558401885271
C	5.059768855438	0.778977655818	0.389434371758
C	5.745027830656	0.976381692178	1.594232628354
C	5.750006014469	0.801098874562	-0.831494182849
C	7.119925985154	1.195186813587	1.556539018128
C	7.121566460914	1.026176441282	-0.821959990934
H	7.649935675911	1.345520572710	2.491374590205
H	7.653110404859	1.046218247217	-1.768054725366
C	7.827816895207	1.225737693288	0.361284824408
C	-0.602000622237	4.876635812982	0.518810262964
C	-0.833258113674	5.559624663925	-0.684246974370
C	-0.590625134715	5.569298483373	1.735591845571
C	-1.046817135105	6.932487215306	-0.645533724797
C	-0.812293097205	6.944246254192	1.727332383689
H	-1.223240317520	7.458874700567	-1.578180438761
H	-0.806502349853	7.479160056767	2.671364799461
C	-1.040814542179	7.646042649596	0.550067740877
C	-4.706510383133	-0.783149675545	0.553469694212
C	-5.403065406108	-0.792767446597	-0.665048938627
C	-5.389343225348	-0.986330974439	1.758480279822
C	-6.776346715506	-1.005536980037	-0.651782197249
C	-6.766384848905	-1.193098528549	1.724587423225
H	-7.311767185612	-1.013066109007	-1.595833091880
H	-7.293567260741	-1.345731086809	2.660625103560
C	-7.479941562909	-1.206785525232	0.532780714998
C	0.966874943651	-4.888424807277	0.430931521164
C	1.001137723480	-5.620523515290	1.623630785613
C	1.157879243217	-5.533274487643	-0.800973034075
C	1.233243847452	-6.992682266885	1.564335032056
C	1.382933183199	-6.904550323000	-0.813079957156
H	1.263637319314	-7.556821024558	2.490718352394
H	1.527649601636	-7.400259023470	-1.767684877889
C	1.427369648581	-7.655103399238	0.358874609249
C	-4.659729005444	-0.992870694591	3.063434223491
H	-5.351214102148	-1.044520529512	3.901929123819
H	-3.984507357869	-1.847770657616	3.140140127760
H	-4.040320848517	-0.103452361129	3.190413591375
C	-4.686044141718	-0.576675725907	-1.959960022009
H	-4.230900837349	0.414779355224	-2.009131869941
H	-3.875133478406	-1.294238833587	-2.098461178625
H	-5.364132756397	-0.671176333112	-2.805518858597

```
C     -8.957160716105    -1.432434563217     0.514026121464
H     -9.215027067240    -2.332404534996    -0.046846130598
H     -9.360123395802    -1.540950254428     1.519194216312
H     -9.480781560227    -0.606129548086     0.030874985620
C      1.682133135848    -9.126967330991     0.313053507726
H      0.964791403090    -9.636391973538    -0.331918679206
H      2.672063251606    -9.346277597957    -0.090968876024
H      1.621539357455    -9.576752943091     1.302250637644
C      0.777239731806    -4.952796403785     2.942783480731
H      1.003740796365    -5.624010196883     3.768682903827
H      1.387559596389    -4.055724577405     3.058177220140
H     -0.261913529416    -4.634897729063     3.056427064130
C      1.119547423138    -4.764179434200    -2.083383120240
H      1.213910317979    -5.426096957589    -2.941697767896
H      0.188885032696    -4.204159106926    -2.191862087575
H      1.927465487110    -4.031668078955    -2.139660780665
C      5.020720634000     0.952236338872     2.902898801034
H      4.280325364891     1.752196696661     2.969835691582
H      4.475876806442     0.017223824874     3.047019368971
H      5.709976493147     1.069048728679     3.736611162692
C      5.027044714877     0.578307833857    -2.121318307180
H      5.682845880912     0.739615433770    -2.974510389753
H      4.639487643381    -0.440850516702    -2.190900339212
H      4.163465037915     1.238511160744    -2.227849294268
C      9.302442750358     1.467995738174     0.338047523512
H      9.832864106480     0.653965401491    -0.158316168535
H      9.547387686531     2.378000559716    -0.212376146761
H      9.709369412754     1.568308339871     1.342491608531
C     -0.346790137168     4.851890154933     3.025408203551
H     -1.061558006243     4.041133552569     3.179216902923
H      0.645111672311     4.395717089555     3.052339726698
H     -0.422309077251     5.529312210511     3.873448922797
C     -1.264623821680     9.123690187888     0.558740379754
H     -1.333819877051     9.513787876106     1.572457482271
H     -0.451140623578     9.650928204092     0.056804935844
H     -2.180385422929     9.393051918970     0.031014360338
C     -0.856164160712     4.825692821727    -1.986897694422
H      0.046705319573     4.229882290387    -2.137431755892
H     -1.693269479731     4.125681881405    -2.037964549128
H     -0.949644526810     5.512674987090    -2.825500677041
O     -1.075941485178    -1.727468678078     3.827642814503
H     -1.405021903572    -2.361408891871     3.179775161981
H     -0.590893793590    -1.078309184987     3.292676845267
O      1.709797211658     2.164442810427    -2.731882835785
H      2.091213435707     2.741507780595    -2.058909559473
H      1.222868199050     1.496273685899    -2.219442361363
```

137

^{1}TS$_{w2}$(A) E_{tot}(RM06L-D3(Dichloromethane)/def2TZVP/W06) = -3093.11486643 $\langle S^2 \rangle$ = 0.0000

```
Ru    -0.094592215203    -0.012027457695     0.142634951366
O      0.054536556526    -0.015657059045     1.883036598955
O      0.122830991771     0.219401369657    -1.656886443134
C      3.327219283409     0.538585844694     0.158616003971
C     -0.611543731858     3.406449859383     0.117084081416
C     -3.479113447135    -0.548137293895     0.104512257031
C      0.480644556828    -3.416820959162    -0.092521112655
N     -1.737989818683     1.199750039617     0.115835793120
N      1.143290039527     1.686148530053     0.208000323670
N      1.599371023938    -1.229185540254     0.056207184895
N     -1.269835756353    -1.658610413368    -0.008228793561
C      1.610144368538    -2.589256748097    -0.003496848535
C      2.892271865262    -0.792107187913     0.110614049946
C      2.504775031600     1.673288911749     0.184129807795
C      0.723109118166     2.977091141291     0.156293973686
C     -0.841940909120    -2.966611891834    -0.095068796289
C     -2.645147032043    -1.665663114109     0.009575462690
C     -3.038726131960     0.779953901262     0.129525753840
C     -1.732083908808     2.571546888050     0.123243998410
C      2.970082423184    -3.040073714618     0.020543440567
C      3.757279025621    -1.935997824476     0.090864695176
C      2.971765320720     3.031945766546     0.133212462961
C      1.876056396665     3.834201449211     0.110904352061
C     -3.088803992313     3.027085363513     0.144687964078
C     -3.889156222908     1.930106005113     0.157962040355
C     -1.993197524379    -3.814051329339    -0.149649087680
C     -3.093840646233    -3.020130204009    -0.076250425191
H      3.278716352085    -4.072923687584    -0.013206385176
```

H	4.834436289136	-1.893916496841	0.127329116316
H	4.008850180076	3.327775821178	0.111211987480
H	1.844915272128	4.911655578677	0.069606600793
H	-3.388015189817	4.063212136291	0.153953810139
H	-4.966966505411	1.897889677554	0.178017597668
H	-1.958665335691	-4.889229512221	-0.228987300709
H	-4.128967965347	-3.323286626992	-0.086491919775
C	4.800571408739	0.769498922381	0.144978394133
C	5.485010995402	0.994321579667	1.345248188381
C	5.493843282631	0.757793663737	-1.075070961224
C	6.860954857361	1.206511491066	1.304799149727
C	6.866137420076	0.978977535845	-1.068837426219
H	7.390457868891	1.375804844050	2.236727271642
H	7.399548863384	0.974911016227	-2.014157416081
C	7.571037542499	1.205420898301	0.110432589897
C	-0.853881758832	4.874675340513	0.026914543468
C	-1.135522831054	5.450178399172	-1.222413707546
C	-0.796895551183	5.672849165025	1.175779856013
C	-1.344109560223	6.822260828177	-1.296915781025
C	-1.018679275625	7.042503807202	1.054642759022
H	-1.553783992466	7.265896339744	-2.265092719591
H	-0.978977877061	7.658452480495	1.947069118487
C	-1.290430424263	7.638846255265	-0.170476385935
C	-4.951156835498	-0.780140506582	0.199894629204
C	-5.743608066358	-0.793275503112	-0.957244503590
C	-5.540145301412	-0.967518249879	1.456773850919
C	-7.113904780144	-0.989278254397	-0.835500791172
C	-6.917934457284	-1.159145866662	1.533179748523
H	-7.721345901396	-0.998282431161	-1.734940762671
H	-7.370695674800	-1.299296525869	2.509314786319
C	-7.724362139532	-1.172516808243	0.402366050548
C	0.716848872370	-4.888862400491	-0.185611909841
C	0.671529112381	-5.684206831398	0.966113168465
C	0.997199914683	-5.473267963648	-1.431233458488
C	0.911243283271	-7.051906932560	0.852552402246
C	1.225661988155	-6.842134980821	-1.498998457232
H	0.880912493860	-7.661828147294	1.749455763274
H	1.439224446735	-7.287065951555	-2.465651904042
C	1.189931153627	-7.652868709834	-0.367773849440
C	-4.711716674472	-0.977992871572	2.701223990148
H	-5.337016440538	-0.995776994792	3.591645900403
H	-4.059296423085	-1.852856617598	2.739541645276
H	-4.054882264951	-0.108810804288	2.765566321213
C	-5.121983832747	-0.611765770015	-2.303433905753
H	-4.623692785679	0.355893094305	-2.398886810632
H	-4.356758633124	-1.367680249428	-2.489377920024
H	-5.862358082390	-0.684048210856	-3.097831931943
C	-9.201176204983	-1.378666249237	0.502975419079
H	-9.515406730187	-2.276211473670	-0.032485937121
H	-9.523312220409	-1.479551898997	1.537642719758
H	-9.751093755156	-0.546270369599	0.061137749207
C	1.447235666551	-9.121324322623	-0.471395437416
H	0.745282550168	-9.601836354856	-1.154695027895
H	2.446308133274	-9.323937166240	-0.860891775634
H	1.361482772686	-9.613645394610	0.495440807469
C	0.354557757046	-5.091999689375	2.301550120144
H	0.581167497088	-5.788685827838	3.106325545752
H	0.905096266464	-4.169158711485	2.485724926221
H	-0.706220690135	-4.841928470998	2.381247261320
C	1.050305637417	-4.643163667964	-2.673715879232
H	1.208203451511	-5.261639747929	-3.555049138647
H	0.127695807173	-4.078475880983	-2.821328393154
H	1.859370886182	-3.909981128980	-2.635350862441
C	4.758528065127	1.004688621271	2.652920304885
H	4.058554413070	1.840314466376	2.719087326122
H	4.167462146023	0.098073112365	2.795148559396
H	5.451333687689	1.088579857095	3.487783964557
C	4.774429015258	0.505014385297	-2.362467720292
H	5.419598071186	0.695193741746	-3.218106207708
H	4.434422102477	-0.531589772162	-2.433723100890
H	3.881637971106	1.126575026440	-2.464863008297
C	9.046449931947	1.443030558403	0.083936639538
H	9.574564117617	0.623083202690	-0.405018598971
H	9.293666762677	2.347354494940	-0.474939984524
H	9.454180388244	1.552136195605	1.087163342633
C	-0.507653494766	5.072966889870	2.515350040976
H	-1.167176164016	4.231064900853	2.732763063323

H	0.512305070293	4.687108284528	2.574480339684
H	-0.626623505677	5.807453848180	3.309402030950
C	-1.519394560458	9.111463051166	-0.283774895776
H	-1.453905506595	9.604243488158	0.684447797703
H	-0.787766049127	9.578323633360	-0.945328087276
H	-2.501830629299	9.331370095773	-0.704634003761
C	-1.220353043371	4.606947048804	-2.456145394168
H	-0.370135016722	3.927057924614	-2.550909070311
H	-2.114693483139	3.978777913451	-2.450281549925
H	-1.261841495570	5.224066097183	-3.351958580497
C	0.322110315280	-0.993331470169	-2.961601533729
C	-0.956513852021	-1.340211138618	-3.382714620838
C	-1.765803875867	-0.524943807317	-4.228099856634
H	-1.399833989601	-2.257546427157	-3.005965106407
H	0.915066381617	-0.344906195580	-3.595538897745
H	0.903804631350	-1.741351063983	-2.437853073191
H	-0.595999449475	1.276917716744	-3.989726146205
C	-1.461061652402	0.831507576751	-4.466432648158
C	-2.275293562041	1.605888180667	-5.268004336184
H	-2.034952524021	2.650221435010	-5.424305924428
H	-4.034642097754	1.664803703644	-6.501225526138
C	-3.402270276972	1.053960229786	-5.869858225481
C	-3.716248061900	-0.283544035220	-5.652423574874
C	-2.919272071625	-1.058400099008	-4.834721389749
H	-3.169498777654	-2.097394582140	-4.654183337562
H	-4.593649452973	-0.715992491999	-6.116801839851
O	-1.192528712373	-2.067605144115	3.308859014182
H	-1.510197348798	-2.664008533689	2.621290763557
H	-0.726428482364	-1.368546563450	2.811398242950
O	1.605473186285	2.257282178043	-2.956218577606
H	1.962825886522	2.894255296756	-2.324683240799
H	1.119073466254	1.621209222912	-2.398844190433

137

^{1,3}**MECP$_{w2}$(A) [singlet]** E_{tot}(RM06L-D3(Dichloromethane)/def2TZVP/W06) = -3093.11823783 $\langle S^2 \rangle$ = 0.0000

Ru	-0.102022490000	-0.004437540000	0.152468590000
O	0.038344260000	-0.022274160000	1.894234880000
O	0.132124060000	0.189894520000	-1.677698400000
C	3.317191110000	0.559952420000	0.190838090000
C	-0.630026840000	3.409480090000	0.097431660000
C	-3.480500640000	-0.560515120000	0.066172770000
C	0.491938140000	-3.407228300000	-0.118888730000
N	-1.743455250000	1.194771190000	0.076013640000
N	1.126063700000	1.694381030000	0.220915880000
N	1.597838560000	-1.214489410000	0.056666510000
N	-1.264402010000	-1.656719050000	-0.024553530000
C	1.616331130000	-2.574995350000	-0.006068390000
C	2.888992680000	-0.772562530000	0.132600900000
C	2.488514200000	1.689106130000	0.210029670000
C	0.704004270000	2.984736590000	0.156531540000
C	-0.831479150000	-2.961863040000	-0.128898140000
C	-2.640220830000	-1.672301370000	-0.033738810000
C	-3.045064370000	0.768909710000	0.091163720000
C	-1.745672410000	2.568397020000	0.088756250000
C	2.976090520000	-3.019507020000	0.039885450000
C	3.757931320000	-1.911489370000	0.125353330000
C	2.951591580000	3.049867450000	0.155606630000
C	1.854032960000	3.847353340000	0.117730510000
C	-3.102552550000	3.015767290000	0.110002940000
C	-3.898331670000	1.913715880000	0.123577220000
C	-1.978483610000	-3.814816930000	-0.214360660000
C	-3.082920930000	-3.027686900000	-0.145352710000
H	3.289896900000	-4.050888130000	0.010279430000
H	4.834044470000	-1.865303120000	0.182443660000
H	3.987864960000	3.348926560000	0.140075520000
H	1.818249440000	4.924301920000	0.067515610000
H	-3.407309160000	4.050246560000	0.123391040000
H	-4.975933530000	1.876898180000	0.147049450000
H	-1.936906900000	-4.888074980000	-0.313705730000
H	-4.116722080000	-3.333835980000	-0.178003010000
C	4.789449280000	0.797517910000	0.190136610000
C	5.460866300000	1.039233400000	1.394529710000
C	5.495062230000	0.776217080000	-1.022781540000
C	6.836103490000	1.258070900000	1.365256470000
C	6.865991230000	1.005048460000	-1.005565710000
H	7.355695920000	1.439818900000	2.300410480000
H	7.408622380000	0.994114260000	-1.945584690000

C	7.557995220000	1.248045090000	0.178022400000
C	-0.879995810000	4.876029110000	0.004139990000
C	-1.156157010000	5.448948650000	-1.247619850000
C	-0.837770340000	5.675269580000	1.152932940000
C	-1.373517820000	6.819490540000	-1.324626600000
C	-1.068289360000	7.043265600000	1.029309290000
H	-1.578521030000	7.261111720000	-2.294716260000
H	-1.040336480000	7.659922340000	1.921698750000
C	-1.334433190000	7.637044150000	-0.198293830000
C	-4.949993150000	-0.798521250000	0.184973280000
C	-5.773598890000	-0.771908310000	-0.949737760000
C	-5.505886540000	-1.027752480000	1.450405360000
C	-7.140639620000	-0.969986170000	-0.797762060000
C	-6.881548180000	-1.219546970000	1.557603090000
H	-7.771923420000	-0.948329040000	-1.680399880000
H	-7.308176790000	-1.390601360000	2.540541980000
C	-7.718218340000	-1.193562730000	0.449162010000
C	0.737390160000	-4.875958080000	-0.235048830000
C	0.654641150000	-5.698556860000	0.895128110000
C	1.064553280000	-5.430355080000	-1.483469090000
C	0.903588950000	-7.062555520000	0.758061110000
C	1.300343610000	-6.796431220000	-1.575202100000
H	0.844597920000	-7.693370350000	1.638919830000
H	1.549914490000	-7.217788240000	-2.543781030000
C	1.227151100000	-7.633871160000	-0.465233620000
C	-4.645523400000	-1.079147320000	2.671986480000
H	-5.247913560000	-1.116695020000	3.577467710000
H	-3.999605390000	-1.959738830000	2.669909460000
H	-3.979939490000	-0.217166770000	2.743014120000
C	-5.187714470000	-0.552305220000	-2.305757630000
H	-4.685550150000	0.414600430000	-2.387872220000
H	-4.432331100000	-1.306520310000	-2.532918810000
H	-5.949228540000	-0.598348460000	-3.082007220000
C	-9.192128060000	-1.402252420000	0.582022630000
H	-9.517115480000	-2.296308360000	0.046912700000
H	-9.490500570000	-1.512735780000	1.622816010000
H	-9.753255760000	-0.567522500000	0.159371350000
C	1.490657440000	-9.099191940000	-0.594434580000
H	0.797089790000	-9.568184890000	-1.294274980000
H	2.494155990000	-9.291601660000	-0.977356050000
H	1.395268340000	-9.610579890000	0.361566390000
C	0.288036630000	-5.140762540000	2.232711710000
H	0.521627130000	-5.842770040000	3.030951640000
H	0.799770620000	-4.201936970000	2.444720440000
H	-0.783689820000	-4.933456060000	2.292631260000
C	1.156069710000	-4.571917400000	-2.704454950000
H	1.350516160000	-5.169382650000	-3.592979440000
H	0.234209670000	-4.011397490000	-2.872500350000
H	1.956480450000	-3.832958390000	-2.621159960000
C	4.721415970000	1.061102920000	2.694819800000
H	4.027766350000	1.902816300000	2.750361810000
H	4.121351380000	0.160188450000	2.835182650000
H	5.406565220000	1.143522410000	3.536157720000
C	4.790403870000	0.506501730000	-2.315029530000
H	5.439387560000	0.703773480000	-3.166187940000
H	4.468311220000	-0.535883430000	-2.385087380000
H	3.888672140000	1.113256870000	-2.426906960000
C	9.032103720000	1.494609050000	0.163290220000
H	9.569406760000	0.677959470000	-0.321056210000
H	9.278077670000	2.400302880000	-0.394001740000
H	9.431028620000	1.607093270000	1.169687360000
C	-0.556442690000	5.077783160000	2.495222450000
H	-1.213918130000	4.233000210000	2.707755560000
H	0.464661120000	4.696484120000	2.562904110000
H	-0.685354520000	5.812244950000	3.287716620000
C	-1.573321070000	9.107876170000	-0.314268020000
H	-1.514277260000	9.602396220000	0.653489830000
H	-0.843166210000	9.579053270000	-0.974333220000
H	-2.556061690000	9.320369360000	-0.738233700000
C	-1.225599900000	4.604533380000	-2.481503420000
H	-0.368135570000	3.933154580000	-2.571097790000
H	-2.113591330000	3.967427070000	-2.480069600000
H	-1.268277660000	5.220755190000	-3.377892970000
C	0.353726980000	-0.894473520000	-2.856711780000
C	-0.912216830000	-1.325544340000	-3.301137200000
C	-1.737975480000	-0.558184580000	-4.152746270000
H	-1.316533290000	-2.254983600000	-2.913488700000

H	0.929922150000	-0.300222030000	-3.561382310000
H	0.967442190000	-1.661681720000	-2.393371730000
H	-0.587081650000	1.271240560000	-3.997180280000
C	-1.455470780000	0.798799760000	-4.440266560000
C	-2.296165290000	1.535911230000	-5.246896420000
H	-2.075679830000	2.578698850000	-5.438110120000
H	-4.072570060000	1.529833050000	-6.456670160000
C	-3.422764070000	0.948100230000	-5.815857180000
C	-3.712985290000	-0.388838450000	-5.556950060000
C	-2.896159220000	-1.126513370000	-4.728813070000
H	-3.126532060000	-2.163428120000	-4.513530290000
H	-4.589625540000	-0.846729140000	-5.997524060000
O	-1.114804760000	-2.129655880000	3.271681640000
H	-1.390845550000	-2.752800470000	2.590296260000
H	-0.689416310000	-1.405314640000	2.766982170000
O	1.605464190000	2.265700350000	-2.935408620000
H	1.969894750000	2.906964190000	-2.312089520000
H	1.126095010000	1.635072560000	-2.367582270000

137

[1,3]MECP$_{w2}$(A) [triplet] E_{tot}(UM06L-D3(Dichloromethane)/def2TZVP/W06) = -3093.11823821 $\langle S^2 \rangle$ = 2.0345

Ru	-0.102022490000	-0.004437540000	0.152468590000
O	0.038344260000	-0.022274160000	1.894234880000
O	0.132124060000	0.189894520000	-1.677698400000
C	3.317191110000	0.559952420000	0.190838090000
C	-0.630026840000	3.409480090000	0.097431660000
C	-3.480500640000	-0.560515120000	0.066172770000
C	0.491938140000	-3.407228300000	-0.118888730000
N	-1.743455250000	1.194771190000	0.076013640000
N	1.126063700000	1.694381030000	0.220915880000
N	1.597838560000	-1.214489410000	0.056666510000
N	-1.264402010000	-1.656719050000	-0.024553530000
C	1.616331130000	-2.574995350000	-0.006068390000
C	2.888992680000	-0.772562530000	0.132600900000
C	2.488514200000	1.689106130000	0.210029670000
C	0.704004270000	2.984736590000	0.156531540000
C	-0.831479150000	-2.961863040000	-0.128898140000
C	-2.640220830000	-1.672301370000	-0.033738810000
C	-3.045064370000	0.768909710000	0.091163720000
C	-1.745672410000	2.568397020000	0.088756250000
C	2.976090520000	-3.019507020000	0.039885450000
C	3.757931320000	-1.911489370000	0.125353330000
C	2.951591580000	3.049867450000	0.155606630000
C	1.854032960000	3.847353340000	0.117730510000
C	-3.102552550000	3.015767290000	0.110002940000
C	-3.898331670000	1.913715880000	0.123577220000
C	-1.978483610000	-3.814816930000	-0.214360660000
C	-3.082920930000	-3.027686900000	-0.145352710000
H	3.289896900000	-4.050888130000	0.010279430000
H	4.834044470000	-1.865303120000	0.182443660000
H	3.987864960000	3.348926560000	0.140075520000
H	1.818249440000	4.924301920000	0.067515610000
H	-3.407309160000	4.050246560000	0.123391040000
H	-4.975933530000	1.876898180000	0.147049450000
H	-1.936906900000	-4.888074980000	-0.313705730000
H	-4.116722080000	-3.333835980000	-0.178003010000
C	4.789449280000	0.797517910000	0.190136610000
C	5.460866300000	1.039233400000	1.394529710000
C	5.495062230000	0.776217080000	-1.022781540000
C	6.836103490000	1.258070900000	1.365256470000
C	6.865991230000	1.005048460000	-1.005565710000
H	7.355695920000	1.439818900000	2.300410480000
H	7.408622380000	0.994114260000	-1.945584690000
C	7.557995220000	1.248045090000	0.178022400000
C	-0.879995810000	4.876029110000	0.004139990000
C	-1.156157010000	5.448948650000	-1.247619850000
C	-0.837770340000	5.675269580000	1.152932940000
C	-1.373517820000	6.819490540000	-1.324626600000
C	-1.068289360000	7.043265600000	1.029309290000
H	-1.578521030000	7.261111720000	-2.294716260000
H	-1.040336480000	7.659922340000	1.921698750000
C	-1.334433190000	7.637044150000	-0.198293830000
C	-4.949993150000	-0.798521250000	0.184973280000
C	-5.773598890000	-0.771908310000	-0.949737760000
C	-5.505886540000	-1.027752480000	1.450405360000
C	-7.140639620000	-0.969986170000	-0.797762060000
C	-6.881548180000	-1.219546970000	1.557603090000

H	-7.771923420000	-0.948329040000	-1.680399880000
H	-7.308176790000	-1.390601360000	2.540541980000
C	-7.718218340000	-1.193562730000	0.449162010000
C	0.737390160000	-4.875958080000	-0.235048830000
C	0.654641150000	-5.698556860000	0.895128110000
C	1.064553280000	-5.430355080000	-1.483469090000
C	0.903588950000	-7.062555520000	0.758061110000
C	1.300343610000	-6.796431220000	-1.575202100000
H	0.844597920000	-7.693370350000	1.638919830000
H	1.549914490000	-7.217788240000	-2.543781030000
C	1.227151100000	-7.633871160000	-0.465233620000
C	-4.645523400000	-1.079147320000	2.671986480000
H	-5.247913560000	-1.116695020000	3.577467710000
H	-3.999605390000	-1.959738830000	2.669909460000
H	-3.979939490000	-0.217166770000	2.743014120000
C	-5.187714470000	-0.552305220000	-2.305757630000
H	-4.685550150000	0.414600430000	-2.387872220000
H	-4.432331100000	-1.306520310000	-2.532918810000
H	-5.949228540000	-0.598348460000	-3.082007220000
C	-9.192128060000	-1.402252420000	0.582022630000
H	-9.517115480000	-2.296308360000	0.046912700000
H	-9.490500570000	-1.512735780000	1.622816010000
H	-9.753255760000	-0.567522500000	0.159371350000
C	1.490657440000	-9.099191940000	-0.594434580000
H	0.797089790000	-9.568184890000	-1.294274980000
H	2.494155990000	-9.291601660000	-0.977356050000
H	1.395268340000	-9.610579890000	0.361566390000
C	0.288036630000	-5.140762540000	2.232711710000
H	0.521627130000	-5.842770040000	3.030951640000
H	0.799770620000	-4.201936970000	2.444720440000
H	-0.783689820000	-4.933456060000	2.292631260000
C	1.156069710000	-4.571917400000	-2.704454950000
H	1.350516160000	-5.169382650000	-3.592979440000
H	0.234209670000	-4.011397490000	-2.872500350000
H	1.956480450000	-3.832958390000	-2.621159960000
C	4.721415970000	1.061102920000	2.694819800000
H	4.027766350000	1.902816300000	2.750361810000
H	4.121351380000	0.160188450000	2.835182650000
H	5.406565220000	1.143522410000	3.536157720000
C	4.790403870000	0.506501730000	-2.315029530000
H	5.439387560000	0.703773480000	-3.166187940000
H	4.468311220000	-0.535883430000	-2.385087380000
H	3.888672140000	1.113256870000	-2.426906960000
C	9.032103720000	1.494609050000	0.163290220000
H	9.569406760000	0.677959470000	-0.321056210000
H	9.278077670000	2.400302880000	-0.394001740000
H	9.431028620000	1.607093270000	1.169687360000
C	-0.556442690000	5.077783160000	2.495222450000
H	-1.213918130000	4.233000210000	2.707755560000
H	0.464661120000	4.696484120000	2.562904110000
H	-0.685354520000	5.812244950000	3.287716620000
C	-1.573321070000	9.107876170000	-0.314268020000
H	-1.514277260000	9.602396220000	0.653489830000
H	-0.843166210000	9.579053270000	-0.974333220000
H	-2.556061690000	9.320369360000	-0.738233700000
C	-1.225599900000	4.604533380000	-2.481503420000
H	-0.368135570000	3.933154580000	-2.571097790000
H	-2.113591330000	3.967427070000	-2.480069600000
H	-1.268277660000	5.220755190000	-3.377892970000
C	0.353726980000	-0.894473520000	-2.856711780000
C	-0.912216830000	-1.325544340000	-3.301137200000
C	-1.737975480000	-0.558184580000	-4.152746270000
H	-1.316533290000	-2.254983600000	-2.913488700000
H	0.929922150000	-0.300222030000	-3.561382310000
H	0.967442190000	-1.661681720000	-2.393371730000
H	-0.587081650000	1.271240560000	-3.997180280000
C	-1.455470780000	0.798799760000	-4.440266560000
C	-2.296165290000	1.535911230000	-5.246896420000
H	-2.075679830000	2.578698850000	-5.438110120000
H	-4.072570060000	1.529833050000	-6.456670160000
C	-3.422764070000	0.948100230000	-5.815857180000
C	-3.712985290000	-0.388838450000	-5.556950060000
C	-2.896159220000	-1.126513370000	-4.728813070000
H	-3.126532060000	-2.163428120000	-4.513530290000
H	-4.589625540000	-0.846729140000	-5.997524060000
O	-1.114804760000	-2.129655880000	3.271681640000
H	-1.390845550000	-2.752800470000	2.590296260000

H	-0.689416310000	-1.405314640000	2.766982170000
O	1.605464190000	2.265700350000	-2.935408620000
H	1.969894750000	2.906964190000	-2.312089520000
H	1.126095010000	1.635072560000	-2.367582270000

137

^{1}**E$_{w2}$** E_{tot}(RM06L-D3(Dichloromethane)/def2TZVP/W06) = -3093.12505811 $\langle S^2 \rangle$ = 0.0000

Ru	0.143730140287	-0.003720686326	0.626701480004
O	0.312351996946	-0.073341435841	2.363036430725
O	0.331282485899	0.161607474653	-1.326539388243
C	3.558717936651	0.543519047966	0.576113463226
C	-0.377235865098	3.406463565009	0.500327116967
C	-3.238263005226	-0.555773780782	0.564023759669
C	0.714517116795	-3.413565820993	0.312358169667
N	-1.488544285859	1.191502929336	0.541941404909
N	1.368339100715	1.681858356944	0.630574754672
N	1.829745554452	-1.221139779991	0.461294774677
N	-1.032448581177	-1.661208302345	0.460408983336
C	1.842609477565	-2.583070170915	0.402065543236
C	3.124394837552	-0.787435823927	0.520414849531
C	2.733465603707	1.672703521129	0.601114691473
C	0.953824225653	2.975669789925	0.552956868928
C	-0.606867733995	-2.966245607464	0.335857786577
C	-2.404934529836	-1.673212611044	0.467674931599
C	-2.794958715020	0.769719835133	0.573130893037
C	-1.492403538708	2.567296126805	0.525740620926
C	3.199953472598	-3.033663513575	0.426465984462
C	3.988199932037	-1.928781164386	0.501275907586
C	3.200780353756	3.029810828474	0.526508967996
C	2.106360605658	3.831550120339	0.488886201639
C	-2.846920120286	3.016979073418	0.553194979160
C	-3.644127400929	1.916600280590	0.594725673976
C	-1.757670718651	-3.817019857332	0.259787025042
C	-2.857303971681	-3.026673166278	0.351672178607
H	3.508460392498	-4.066545758363	0.392953979589
H	5.065162801529	-1.887058051867	0.543892284109
H	4.238151574074	3.323695107196	0.494104197078
H	2.074404049231	4.907718270492	0.422603465994
H	-3.149843156753	4.052056541255	0.551484748221
H	-4.721514660840	1.882657440305	0.630806992355
H	-1.720638320256	-4.889454327762	0.150038511372
H	-3.892893402762	-3.327921473696	0.329974254860
C	5.031648610528	0.776589432612	0.569827674857
C	5.706270370800	1.012745077921	1.773701145470
C	5.735007658505	0.757001737230	-0.644306368384
C	7.082128398721	1.227111712786	1.742759553799
C	7.106766420605	0.981555451707	-0.628820103194
H	7.603841032177	1.404425804786	2.677598037915
H	7.647657206293	0.972093648110	-1.569871769677
C	7.801819047450	1.218430373057	0.554207091838
C	-0.623376390890	4.869925692480	0.363268354096
C	-0.923537613171	5.399331149193	-0.902508364791
C	-0.553135922948	5.709749368257	1.481254786226
C	-1.134986527058	6.767603561679	-1.024241514223
C	-0.778702193790	7.073893003585	1.313283431869
H	-1.358713073454	7.175389065503	-2.004951309594
H	-0.728761353470	7.721982018889	2.182132617634
C	-1.067084950317	7.624925225465	0.070852409775
C	-4.709435905353	-0.786582626189	0.669448900850
C	-5.519930098765	-0.745457430240	-0.474597752918
C	-5.279622833560	-1.032165482176	1.925061201842
C	-6.888289718721	-0.947295628078	-0.341480102418
C	-6.656369116281	-1.226051131989	2.013702447272
H	-7.509144775612	-0.917389905418	-1.231270443725
H	-7.094403606912	-1.411047436301	2.989094646278
C	-7.479960618246	-1.187213167874	0.895881463150
C	0.954452074330	-4.881024675430	0.174853710898
C	0.897504991294	-5.716573660750	1.296982789817
C	1.247240681745	-5.421524653880	-1.088089879317
C	1.140700493123	-7.079267089527	1.137953519752
C	1.477645093512	-6.786867580465	-1.201777086848
H	1.101899091664	-7.720448408838	2.012455060259
H	1.699847595177	-7.197715060616	-2.181465562819
C	1.432586487915	-7.636721723972	-0.099758749971
C	-4.432324073900	-1.100495389018	3.155029765345
H	-5.044074011309	-1.139659180330	4.054182415313
H	-3.794632410640	-1.987126559695	3.152435592608

H	-3.759684898024	-0.245227237464	3.238870842381
C	-4.918729016672	-0.502782214796	-1.820629607723
H	-4.458760882286	0.486024045606	-1.893512884290
H	-4.126774768232	-1.222893364110	-2.033335011294
H	-5.664304286131	-0.580123945704	-2.609856576520
C	-8.955852558325	-1.393112503869	1.009909340250
H	-9.291920725723	-2.225361116317	0.389484498354
H	-9.254712477408	-1.599711385877	2.035817416804
H	-9.507605565244	-0.513582652755	0.673629702354
C	1.695859614905	-9.099911306023	-0.251882543421
H	1.020454692657	-9.553557926254	-0.978757579048
H	2.709044134116	-9.286257808481	-0.612320294109
H	1.577051271846	-9.630014485832	0.691201900279
C	0.562951585990	-5.173218846647	2.648855226690
H	0.812872014172	-5.884728132137	3.433666353546
H	1.081335470229	-4.237647170221	2.859437604418
H	-0.506358355346	-4.963376131043	2.735256838883
C	1.308017784202	-4.549411242747	-2.301559089936
H	1.470113770946	-5.138034815546	-3.202413102346
H	0.386318168998	-3.978999415768	-2.434623219224
H	2.117024723575	-3.818240135780	-2.234069354930
C	4.969494028696	1.034464217927	3.075576612103
H	4.283339652668	1.881950699463	3.136354426282
H	4.361752491178	0.138225617770	3.212935711927
H	5.656940090347	1.107167103015	3.915951267190
C	5.027465261159	0.492980325722	-1.936092690824
H	5.672485103427	0.700737915747	-2.787824364446
H	4.712308094286	-0.551031368427	-2.012908314261
H	4.121103016093	1.094104318381	-2.040267986412
C	9.276784645919	1.459891505973	0.537757567043
H	9.810560840790	0.641500608810	0.052418163352
H	9.525365062894	2.365030500954	-0.019270935596
H	9.677313167742	1.570293971049	1.543754353984
C	-0.246974790213	5.159391397032	2.838209340329
H	-0.896361254365	4.319197511381	3.089827217170
H	0.777171438750	4.785532748398	2.901886967253
H	-0.366448216061	5.919846024478	3.607426514830
C	-1.298668733516	9.092380246305	-0.093203570332
H	-1.229320374220	9.619158824973	0.856687149565
H	-0.570480386160	9.536478815468	-0.774014792440
H	-2.283160437431	9.296298343096	-0.517238223520
C	-1.025197723515	4.511871321591	-2.103872185299
H	-0.176261250600	3.828780797801	-2.187040065539
H	-1.918683756302	3.883619674318	-2.062998765768
H	-1.080296192386	5.096119849950	-3.020891262292
C	0.588908901542	-0.824858497993	-2.341852880191
C	-0.713451982768	-1.220049411715	-2.879427011402
C	-1.452946263785	-0.429913831883	-3.768521521161
H	-1.173164467366	-2.131241693199	-2.508005606517
H	1.236310398217	-0.366900240121	-3.097927975698
H	1.123309378274	-1.677717110354	-1.917065166944
H	-0.135806200825	1.294765983988	-3.713069714276
C	-1.042096966861	0.878774174961	-4.135632652862
C	-1.804678998850	1.635828115793	-4.995874728697
H	-1.488359327292	2.639873384329	-5.249337328781
H	-3.568996894713	1.719706598626	-6.221729804931
C	-2.978923877853	1.119438743181	-5.541357642352
C	-3.393366150675	-0.169532382278	-5.210618676971
C	-2.654990958281	-0.927661602619	-4.332248286225
H	-2.976871388814	-1.926839982023	-4.063929044638
H	-4.303645831144	-0.570514611848	-5.637910131765
O	-0.883769484246	-2.168735647710	3.743034356897
H	-1.183383404119	-2.774130501293	3.055450980529
H	-0.437681072609	-1.454726925685	3.243795734245
O	1.829805172319	2.220283198040	-2.530176079858
H	2.188870125989	2.873574725033	-1.916640139748
H	1.318197624152	1.615784409223	-1.958830859779

137

^{BS}TS$_{w2}$(A) E_{tot}(UM06L-D3(Dichloromethane)/def2TZVP/W06) = -3093.11546905 $\langle S^2 \rangle$ = 0.2516

Ru	-0.094294999306	-0.011666966885	0.143117161378
O	0.071340575831	-0.030546476670	1.881583485022
O	0.124568115280	0.229068283103	-1.652340895229
C	3.329087951817	0.538616020096	0.147127751848
C	-0.611488109047	3.405235284994	0.134223220718
C	-3.478895845876	-0.549614876050	0.111330523881
C	0.482219790750	-3.416689511579	-0.089183618237

N	-1.737389679573	1.198108279708	0.133113799972
N	1.144826984304	1.685974593302	0.211351506269
N	1.602270762115	-1.229622557543	0.049705148486
N	-1.270124169535	-1.659626736610	-0.004995146865
C	1.612465496169	-2.589156928860	-0.008175386140
C	2.894606428919	-0.792376504667	0.098674476038
C	2.506310518675	1.672948772327	0.180318761917
C	0.723899872286	2.976638777090	0.165873586671
C	-0.840807143526	-2.967238500788	-0.089302187347
C	-2.644737806308	-1.667258342914	0.015803400158
C	-3.038310696338	0.778031812221	0.143293493947
C	-1.731653431412	2.570148345945	0.143290915715
C	2.972897816410	-3.040394652639	0.009230505270
C	3.760227246317	-1.936396125569	0.074783948443
C	2.972397792492	3.031891699454	0.132184741185
C	1.876277046910	3.834046367297	0.117982189623
C	-3.088618143736	3.025254947225	0.165991560691
C	-3.888757070625	1.928289368144	0.174695196334
C	-1.991494564564	-3.815574366224	-0.139861769295
C	-3.092755078970	-3.022199440374	-0.067499940868
H	3.281198526839	-4.073325600941	-0.025311721781
H	4.837553667172	-1.894013757752	0.105510596029
H	4.009261186202	3.328187266885	0.106423769722
H	1.844774614163	4.911645244398	0.081044872622
H	-3.388023295015	4.061293389415	0.178017112046
H	-4.966584353309	1.895911501964	0.193320894629
H	-1.956334333047	-4.890962480761	-0.215983327863
H	-4.127671571284	-3.326127605470	-0.075715577013
C	4.802267930940	0.770267625359	0.127642550578
C	5.491464926212	0.993535288809	1.325510520335
C	5.490669600881	0.760405451820	-1.095191363473
C	6.867133855791	1.206335733584	1.279870081622
C	6.862894099409	0.982161404831	-1.094065289360
H	7.400303249024	1.374462527557	2.209917696002
H	7.392530299749	0.979582979654	-2.041508938783
C	7.572424944249	1.207260713565	0.082664943386
C	-0.854764475259	4.873475654479	0.046767837053
C	-1.137592593244	5.450582909258	-1.201533610644
C	-0.797478640219	5.669857447192	1.196808105838
C	-1.347361156716	6.822625556268	-1.273734354393
C	-1.020373945460	7.039520453660	1.077952063641
H	-1.558063086211	7.267616927481	-2.241064708701
H	-0.980330508627	7.654145087633	1.971275985337
C	-1.293559583214	7.637496140486	-0.146056490374
C	-4.951664709314	-0.781749958658	0.194844358202
C	-5.732485226352	-0.796927795748	-0.970249173755
C	-5.553199182909	-0.967815232525	1.445883241199
C	-7.103767942751	-0.993822809832	-0.862046155966
C	-6.931567623159	-1.160457863229	1.508626936647
H	-7.702117211517	-1.004168025346	-1.767550856549
H	-7.394026838769	-1.299777986042	2.480323723368
C	-7.726555866160	-1.175980792160	0.369800875453
C	0.718120241371	-4.889351609250	-0.172904781958
C	0.685400478644	-5.673710594322	0.986820936595
C	0.986036880606	-5.485003829289	-1.415718041050
C	0.924443014836	-7.042305450436	0.883572521263
C	1.214476763956	-6.854424949463	-1.473039962262
H	0.903658117754	-7.643842691472	1.786398311516
H	1.418448304377	-7.308303840788	-2.437612146034
C	1.190700579593	-7.654527631735	-0.334007385486
C	-4.737290908284	-0.976373414873	2.698554400866
H	-5.371387701927	-0.995011697156	3.582706905103
H	-4.083989300224	-1.850260585353	2.743569515460
H	-4.082659261204	-0.106072374944	2.769136111919
C	-5.098227088821	-0.615791046740	-2.310660700031
H	-4.598064305893	0.351389090063	-2.401074893967
H	-4.332394096148	-1.372615966524	-2.490551711492
H	-5.831793410694	-0.686731305797	-3.111425791301
C	-9.204132805809	-1.383227734062	0.455879638490
H	-9.512206811832	-2.281930311179	-0.081214853695
H	-9.536525387318	-1.482681865670	1.487446084504
H	-9.750288479783	-0.552040775461	0.007160712260
C	1.447259646303	-9.123857968591	-0.426664310717
H	0.736599234192	-9.611204480130	-1.096005920422
H	2.441218364420	-9.330010141257	-0.827195658431
H	1.374236838418	-9.606783996599	0.545934460560
C	0.383539628900	-5.068064734926	2.319713351734

H	0.604784942669	-5.762265066639	3.128078805569
H	0.948322274285	-4.151819916440	2.494435541252
H	-0.672406862623	-4.800354436091	2.403310206223
C	1.027380981470	-4.665898089596	-2.665893056081
H	1.174859713143	-5.292244604122	-3.543458300585
H	0.104441150598	-4.100605145960	-2.808674683895
H	1.838385891156	-3.934224615260	-2.642150590869
C	4.770363205462	1.001442665302	2.636190743313
H	4.070857919636	1.837080306062	2.706971252938
H	4.179731267396	0.094638593829	2.779060515809
H	5.466631844458	1.083540295827	3.468350248016
C	4.766433646988	0.508750525109	-2.380095441981
H	5.408201108556	0.700641703228	-3.237922124246
H	4.427376753583	-0.528161922581	-2.451396918951
H	3.872572033566	1.129483086141	-2.478421970258
C	9.047641265145	1.445376401488	0.050593076732
H	9.574178739698	0.625510407564	-0.440210233578
H	9.292466866698	2.349673858502	-0.509365127619
H	9.459103363762	1.554732036889	1.052267289840
C	-0.506606772967	5.067982385658	2.535113089706
H	-1.167385608419	4.227176014589	2.752964377985
H	0.512703605735	4.680030250757	2.591623265957
H	-0.622531453513	5.801791836432	3.330233399607
C	-1.523968431744	9.110079480000	-0.256861100225
H	-1.456611575669	9.601607106747	0.711870648330
H	-0.794334459155	9.578429355843	-0.919547990045
H	-2.507610970930	9.329718280680	-0.675046814801
C	-1.222427371789	4.609157383595	-2.436520040121
H	-0.371966699911	3.929753151900	-2.532717290411
H	-2.116423437581	3.980413434908	-2.431712811332
H	-1.264382329100	5.227684684682	-3.331329413034
C	0.322922821125	-1.022712595404	-2.990160124053
C	-0.958124454789	-1.352173809789	-3.405657609083
C	-1.764540769690	-0.524062217945	-4.247320069775
H	-1.410025028555	-2.266728521288	-3.031328989619
H	0.918588180070	-0.359499043074	-3.604923812269
H	0.897840798482	-1.764430780817	-2.451329444359
H	-0.593401393783	1.271457858341	-3.980791314708
C	-1.456756258530	0.833489828871	-4.467496830014
C	-2.265920231971	1.618908813023	-5.264294026483
H	-2.022781157055	2.664487197417	-5.407541682853
H	-4.020151856492	1.695335995871	-6.503715834411
C	-3.391261587859	1.076136792543	-5.876942103342
C	-3.708506217795	-0.262889913043	-5.675708903920
C	-2.915473190350	-1.048662209677	-4.863655186422
H	-3.168747742585	-2.089040041677	-4.695472514602
H	-4.585019208837	-0.688546315628	-6.148013222852
O	-1.225373579749	-2.058786412325	3.311979608566
H	-1.575004630510	-2.632483727588	2.620486867409
H	-0.741623291587	-1.370405822090	2.818147825243
O	1.605321090380	2.261268149082	-2.955411868568
H	1.958500608798	2.897024545548	-2.320398997131
H	1.119650547687	1.620556727194	-2.402120138064

137

[BS,3]**MECP$_{w2}$(A) [singlet]** E_{tot}(UM06L-D3(Dichloromethane)/def2TZVP/W06) = -3093.13679297 $\langle S^2 \rangle$ = 0.9699

Ru	-0.096946390000	-0.002091060000	0.183944570000
O	0.085983940000	-0.090570170000	1.903850050000
O	0.133150510000	0.135195500000	-1.755207370000
C	3.324386320000	0.540002870000	0.128894320000
C	-0.617491890000	3.405413970000	0.089885830000
C	-3.477811640000	-0.559580560000	0.106597580000
C	0.482286590000	-3.418329800000	-0.060932600000
N	-1.725432510000	1.187143680000	0.106882780000
N	1.130809860000	1.680104760000	0.174999340000
N	1.600667890000	-1.227536840000	0.053112420000
N	-1.272226090000	-1.664599970000	0.026843450000
C	1.612034050000	-2.587921880000	0.016794810000
C	2.892645490000	-0.792228000000	0.099437040000
C	2.496762210000	1.668495510000	0.145149430000
C	0.715886590000	2.973812170000	0.122007940000
C	-0.840167940000	-2.971174970000	-0.060583180000
C	-2.642637910000	-1.678518110000	0.030262950000
C	-3.033114770000	0.763967630000	0.116832760000
C	-1.730082280000	2.565698000000	0.097835420000
C	2.970710450000	-3.039676070000	0.045778600000
C	3.758557110000	-1.934588570000	0.097054600000

C	2.962124840000	3.024970110000	0.085902530000
C	1.866892870000	3.828419130000	0.067116770000
C	-3.086020180000	3.012049450000	0.108483540000
C	-3.882290960000	1.911621560000	0.128292060000
C	-1.988092500000	-3.824456830000	-0.122102950000
C	-3.090808210000	-3.034394190000	-0.057458900000
H	3.278323970000	-4.073143400000	0.028242750000
H	4.835679660000	-1.891131910000	0.131963220000
H	3.999263560000	3.319391260000	0.054957770000
H	1.835381110000	4.905518710000	0.020247530000
H	-3.389774370000	4.046815350000	0.105139880000
H	-4.960033330000	1.876363840000	0.142667120000
H	-1.948174100000	-4.899331720000	-0.202141530000
H	-4.125548380000	-3.338261970000	-0.076962250000
C	4.796762370000	0.775455020000	0.113013360000
C	5.481345500000	1.001513500000	1.312922410000
C	5.488542970000	0.764229030000	-1.107711990000
C	6.856745930000	1.216412610000	1.271288730000
C	6.860351260000	0.988310180000	-1.102462640000
H	7.386657810000	1.387037440000	2.202719060000
H	7.393076220000	0.984666710000	-2.048136780000
C	7.565667530000	1.216598100000	0.076200230000
C	-0.860937250000	4.874094430000	0.015989350000
C	-1.145528960000	5.466316620000	-1.224490090000
C	-0.798826740000	5.656898040000	1.175324660000
C	-1.352545900000	6.839554040000	-1.280120980000
C	-1.019050590000	7.028255950000	1.073047370000
H	-1.565014500000	7.296157390000	-2.241616990000
H	-0.975396120000	7.632258330000	1.973419910000
C	-1.294059160000	7.641050600000	-0.143213990000
C	-4.950924240000	-0.788772460000	0.191378870000
C	-5.734549140000	-0.797559660000	-0.971837220000
C	-5.549379870000	-0.980523610000	1.443084120000
C	-7.105637830000	-0.994063900000	-0.861131970000
C	-6.927733610000	-1.172100870000	1.508351590000
H	-7.706047900000	-1.000481350000	-1.765266780000
H	-7.387836900000	-1.315576620000	2.480558800000
C	-7.725511890000	-1.181625320000	0.371385030000
C	0.722865700000	-4.889125660000	-0.155183000000
C	0.686409960000	-5.685338060000	0.996047350000
C	1.000054170000	-5.469755150000	-1.403249150000
C	0.930374060000	-7.051946570000	0.879364990000
C	1.232636210000	-6.837757160000	-1.473741090000
H	0.906871650000	-7.663220110000	1.775546430000
H	1.443216300000	-7.280735280000	-2.441915150000
C	1.204689690000	-7.650107870000	-0.343424030000
C	-4.730486400000	-0.994528050000	2.693683110000
H	-5.362259070000	-1.020848640000	3.579299800000
H	-4.073778370000	-1.866171580000	2.731727550000
H	-4.078805450000	-0.122229770000	2.768055590000
C	-5.102050500000	-0.609867010000	-2.311825910000
H	-4.621071290000	0.366828340000	-2.405085610000
H	-4.320527710000	-1.351394030000	-2.486692790000
H	-5.832378030000	-0.697992150000	-3.113832610000
C	-9.202915240000	-1.388957930000	0.459979590000
H	-9.511428350000	-2.288463330000	-0.075564210000
H	-9.533790750000	-1.487348000000	1.492139650000
H	-9.749806680000	-0.558515180000	0.010846280000
C	1.464728580000	-9.117759570000	-0.450982630000
H	0.753150070000	-9.600177360000	-1.122952740000
H	2.457862290000	-9.317498910000	-0.856634390000
H	1.395553020000	-9.610475060000	0.516963150000
C	0.377098060000	-5.093758600000	2.333540690000
H	0.594567740000	-5.796001160000	3.135941340000
H	0.940924420000	-4.179197000000	2.520398470000
H	-0.679221230000	-4.827003990000	2.414534920000
C	1.045088960000	-4.637243750000	-2.644625830000
H	1.205192330000	-5.253496840000	-3.527064560000
H	0.118117460000	-4.078713250000	-2.789289780000
H	1.848680490000	-3.898068500000	-2.606650080000
C	4.755695970000	1.010571290000	2.621082580000
H	4.051344850000	1.842590870000	2.685994930000
H	4.169874240000	0.100904540000	2.765879540000
H	5.448633440000	1.099814340000	3.455263850000
C	4.768191730000	0.507795240000	-2.393693790000
H	5.412441780000	0.695837360000	-3.250432360000
H	4.429038490000	-0.529281660000	-2.461392660000

H	3.874681000000	1.128188410000	-2.497324180000
C	9.040465910000	1.457456830000	0.048375250000
H	9.569847610000	0.638975370000	-0.441600020000
H	9.285018840000	2.362637150000	-0.510257570000
H	9.448958210000	1.566929280000	1.051231380000
C	-0.504847330000	5.039617090000	2.505950800000
H	-1.164382180000	4.195767850000	2.715609920000
H	0.515137080000	4.652460070000	2.556061780000
H	-0.619945240000	5.764176020000	3.309600070000
C	-1.521864980000	9.115223610000	-0.236300390000
H	-1.451200070000	9.595334060000	0.737893640000
H	-0.792980360000	9.589765240000	-0.895380510000
H	-2.506101180000	9.341433700000	-0.649533860000
C	-1.238429350000	4.638573940000	-2.467493190000
H	-0.393261230000	3.954080860000	-2.573613870000
H	-2.136804370000	4.016341130000	-2.464041140000
H	-1.280124450000	5.265896600000	-3.356044500000
C	0.284008370000	-0.893978340000	-2.730610160000
C	-1.013038780000	-1.312233530000	-3.312991910000
C	-1.776250950000	-0.488095050000	-4.168811310000
H	-1.388515210000	-2.306295970000	-3.090251160000
H	0.947083800000	-0.497095420000	-3.511479430000
H	0.791067140000	-1.758118490000	-2.291834020000
H	-0.586355280000	1.303335390000	-3.905237180000
C	-1.449065010000	0.870519620000	-4.397550840000
C	-2.224279860000	1.654274160000	-5.228238490000
H	-1.958784010000	2.693324350000	-5.381570440000
H	-3.939469410000	1.741386410000	-6.524218090000
C	-3.338543110000	1.121535640000	-5.871186830000
C	-3.671388750000	-0.216883740000	-5.672189210000
C	-2.911492370000	-1.005689440000	-4.836138050000
H	-3.176259650000	-2.044943490000	-4.677140770000
H	-4.534081190000	-0.639228440000	-6.172912160000
O	-1.264595510000	-2.100337750000	3.338329830000
H	-1.624551140000	-2.671054750000	2.649445440000
H	-0.753464170000	-1.435344220000	2.842796960000
O	1.582918330000	2.229275510000	-2.981721760000
H	1.923153080000	2.880255950000	-2.355587530000
H	1.105991720000	1.587372380000	-2.421179360000

137

[BS,3]MECP$_{w2}$(A) [triplet] E_{tot} (UM06L-D3(Dichloromethane)/def2TZVP/W06) = -3093.13679287 $\langle S^2 \rangle$ = 2.0469

Ru	-0.096946390000	-0.002091060000	0.183944570000
O	0.085983940000	-0.090570170000	1.903850050000
O	0.133150510000	0.135195500000	-1.755207370000
C	3.324386320000	0.540002870000	0.128894320000
C	-0.617491890000	3.405413970000	0.089885830000
C	-3.477811640000	-0.559580560000	0.106597580000
C	0.482286590000	-3.418329800000	-0.060932600000
N	-1.725432510000	1.187143680000	0.106882780000
N	1.130809860000	1.680104760000	0.174999340000
N	1.600667890000	-1.227536840000	0.053112420000
N	-1.272226090000	-1.664599970000	0.026843450000
C	1.612034050000	-2.587921880000	0.016794810000
C	2.892645490000	-0.792228000000	0.099437040000
C	2.496762210000	1.668495510000	0.145149430000
C	0.715886590000	2.973812170000	0.122007940000
C	-0.840167940000	-2.971174970000	-0.060583180000
C	-2.642637910000	-1.678518110000	0.030262950000
C	-3.033114770000	0.763967630000	0.116832760000
C	-1.730082280000	2.565698000000	0.097835420000
C	2.970710450000	-3.039676070000	0.045778600000
C	3.758557110000	-1.934588570000	0.097054600000
C	2.962124840000	3.024970110000	0.085902530000
C	1.866892870000	3.828419130000	0.067116770000
C	-3.086020180000	3.012049450000	0.108483540000
C	-3.882290960000	1.911621560000	0.128292060000
C	-1.988092500000	-3.824456830000	-0.122102950000
C	-3.090808210000	-3.034394190000	-0.057458900000
H	3.278323970000	-4.073143400000	0.028242750000
H	4.835679660000	-1.891131910000	0.131963220000
H	3.999263560000	3.319391260000	0.054957770000
H	1.835381110000	4.905518710000	0.020247530000
H	-3.389774370000	4.046815350000	0.105139880000
H	-4.960033330000	1.876363840000	0.142667120000
H	-1.948174100000	-4.899331720000	-0.202141530000
H	-4.125548380000	-3.338261970000	-0.076962250000

C	4.796762370000	0.775455020000	0.113013360000
C	5.481345500000	1.001513500000	1.312922410000
C	5.488542970000	0.764229030000	-1.107711990000
C	6.856745930000	1.216412610000	1.271288730000
C	6.860351260000	0.988310180000	-1.102462640000
H	7.386657810000	1.387037440000	2.202719060000
H	7.393076220000	0.984666710000	-2.048136780000
C	7.565667530000	1.216598100000	0.076200230000
C	-0.860937250000	4.874094430000	0.015989350000
C	-1.145528960000	5.466316620000	-1.224490090000
C	-0.798826740000	5.656898040000	1.175324660000
C	-1.352545900000	6.839554040000	-1.280120980000
C	-1.019050590000	7.028255950000	1.073047370000
H	-1.565014500000	7.296157390000	-2.241616990000
H	-0.975396120000	7.632258330000	1.973419910000
C	-1.294059160000	7.641050600000	-0.143213990000
C	-4.950924240000	-0.788772460000	0.191378870000
C	-5.734549140000	-0.797559660000	-0.971837220000
C	-5.549379870000	-0.980523610000	1.443084120000
C	-7.105637830000	-0.994063900000	-0.861131970000
C	-6.927733610000	-1.172100870000	1.508351590000
H	-7.706047900000	-1.000481350000	-1.765266780000
H	-7.387836900000	-1.315576620000	2.480558800000
C	-7.725511890000	-1.181625320000	0.371385030000
C	0.722865700000	-4.889125660000	-0.155183000000
C	0.686409960000	-5.685338060000	0.996047350000
C	1.000054170000	-5.469755150000	-1.403249150000
C	0.930374060000	-7.051946570000	0.879364990000
C	1.232636210000	-6.837757160000	-1.473741090000
H	0.906871650000	-7.663220110000	1.775546430000
H	1.443216300000	-7.280735280000	-2.441915150000
C	1.204689690000	-7.650107870000	-0.343424030000
C	-4.730486400000	-0.994528050000	2.693683110000
H	-5.362259070000	-1.020848640000	3.579299800000
H	-4.073778370000	-1.866171580000	2.731727550000
H	-4.078805450000	-0.122229770000	2.768055590000
C	-5.102050500000	-0.609867010000	-2.311825910000
H	-4.621071290000	0.366828340000	-2.405085610000
H	-4.320527710000	-1.351394030000	-2.486692790000
H	-5.832378030000	-0.697992150000	-3.113832610000
C	-9.202915240000	-1.388957930000	0.459979590000
H	-9.511428350000	-2.288463330000	-0.075564210000
H	-9.533790750000	-1.487348000000	1.492139650000
H	-9.749806680000	-0.558515180000	0.010846280000
C	1.464728580000	-9.117759570000	-0.450982630000
H	0.753150070000	-9.600177360000	-1.122952740000
H	2.457862290000	-9.317498910000	-0.856634390000
H	1.395553020000	-9.610475060000	0.516963150000
C	0.377098060000	-5.093758600000	2.333540690000
H	0.594567740000	-5.796001160000	3.135941340000
H	0.940924420000	-4.179197000000	2.520398470000
H	-0.679221230000	-4.827003990000	2.414534920000
C	1.045088960000	-4.637243750000	-2.644625830000
H	1.205192330000	-5.253496840000	-3.527064560000
H	0.118117460000	-4.078713250000	-2.789289780000
H	1.848680490000	-3.898068500000	-2.606650080000
C	4.755695970000	1.010571290000	2.621082580000
H	4.051344850000	1.842590870000	2.685994930000
H	4.169874240000	0.100904540000	2.765879540000
H	5.448633440000	1.099814340000	3.455263850000
C	4.768191730000	0.507795240000	-2.393693790000
H	5.412441780000	0.695837360000	-3.250432360000
H	4.429038490000	-0.529281660000	-2.461392660000
H	3.874681000000	1.128188410000	-2.497324180000
C	9.040465910000	1.457456830000	0.048375250000
H	9.569847610000	0.638975370000	-0.441600020000
H	9.285018840000	2.362637150000	-0.510257570000
H	9.448958210000	1.566929280000	1.051231380000
C	-0.504847330000	5.039617090000	2.505950800000
H	-1.164382180000	4.195767850000	2.715609920000
H	0.515137080000	4.652460070000	2.556061780000
H	-0.619945240000	5.764176020000	3.309600070000
C	-1.521864980000	9.115223610000	-0.236300390000
H	-1.451200070000	9.595334060000	0.737893640000
H	-0.792980360000	9.589765240000	-0.895380510000
H	-2.506101180000	9.341433700000	-0.649533860000
C	-1.238429350000	4.638573940000	-2.467493190000

H	-0.393261230000	3.954080860000	-2.573613870000
H	-2.136804370000	4.016341130000	-2.464041140000
H	-1.280124450000	5.265896600000	-3.356044500000
C	0.284008370000	-0.893978340000	-2.730610160000
C	-1.013038780000	-1.312233530000	-3.312991910000
C	-1.776250950000	-0.488095050000	-4.168811310000
H	-1.388515210000	-2.306295970000	-3.090251160000
H	0.947083800000	-0.497095420000	-3.511479430000
H	0.791067140000	-1.758118490000	-2.291834020000
H	-0.586355280000	1.303335390000	-3.905237180000
C	-1.449065010000	0.870519620000	-4.397550840000
C	-2.224279860000	1.654274160000	-5.228238490000
H	-1.958784010000	2.693324350000	-5.381570440000
H	-3.939469410000	1.741386410000	-6.524218090000
C	-3.338543110000	1.121535640000	-5.871186830000
C	-3.671388750000	-0.216883740000	-5.672189210000
C	-2.911492370000	-1.005689440000	-4.836138050000
H	-3.176259650000	-2.044943490000	-4.677140770000
H	-4.534081190000	-0.639228440000	-6.172912160000
O	-1.264595510000	-2.100337750000	3.338329830000
H	-1.624551140000	-2.671054750000	2.649445440000
H	-0.753464170000	-1.435344220000	2.842796960000
O	1.582918330000	2.229275510000	-2.981721760000
H	1.923153080000	2.880255950000	-2.355587530000
H	1.105991720000	1.587372380000	-2.421179360000

137

$^{BS}E_{w2}$ E_{tot}(UM06L-D3(Dichloromethane)/def2TZVP/W06) = -3093.13711795 $\langle S^2 \rangle$ = 0.9766

Ru	0.154134659921	-0.007057618661	0.580367132834
O	0.298853134697	-0.062778470337	2.308427234432
O	0.361224254730	0.139935234565	-1.381190050732
C	3.570616692742	0.540721232431	0.507859497603
C	-0.374708583765	3.403684351161	0.486968113604
C	-3.230980848065	-0.559393184851	0.496289645210
C	0.729711297320	-3.420357754249	0.359192672282
N	-1.483407950440	1.188454702263	0.497104788995
N	1.376723050511	1.678022629503	0.555937018764
N	1.844930557950	-1.226686914489	0.461060697793
N	-1.023783718989	-1.666134366267	0.424185356013
C	1.857649469161	-2.588322250341	0.427656660586
C	3.138167692643	-0.791013568951	0.490466893756
C	2.742405202794	1.669138160214	0.523916165739
C	0.958797957700	2.971998293546	0.512154454507
C	-0.593040636908	-2.974279690758	0.353830298155
C	-2.395440438889	-1.678326974207	0.430984820644
C	-2.788091953290	0.765676825353	0.502766921452
C	-1.488284912396	2.564483041002	0.490334469141
C	3.216957137239	-3.038771005691	0.444397977397
C	4.004453745019	-1.933160670491	0.483102936055
C	3.205339866330	3.025974116956	0.468151986864
C	2.108302246185	3.827943514540	0.458239832279
C	-2.845481796073	3.013248212834	0.496183347276
C	-3.640846872444	1.913231432134	0.510171012245
C	-1.740916804266	-3.826942776715	0.303206649222
C	-2.843365973248	-3.034755894978	0.358656332876
H	3.525033315077	-4.072120047017	0.426503826577
H	5.081908344530	-1.888945801053	0.505232220256
H	4.241890752843	3.322453329156	0.436060104572
H	2.075257612705	4.905278846760	0.417844619198
H	-3.148541978634	4.048230437763	0.492483528209
H	-4.718637450945	1.876546076306	0.518941921762
H	-1.702178553476	-4.902854958745	0.237538545586
H	-3.878159716010	-3.338843275742	0.344313118864
C	5.042714760452	0.777208051173	0.477520293573
C	5.738461356009	1.012602932160	1.669238302119
C	5.723201866148	0.759488476894	-0.749573223269
C	7.112897867184	1.230469200120	1.613104655643
C	7.094563505821	0.986447815431	-0.758851699198
H	7.651223381252	1.408722214444	2.538282581301
H	7.618273922872	0.977584031880	-1.709525804845
C	7.810548790458	1.224308022556	0.411423539594
C	-0.617016618501	4.873504664920	0.429743963849
C	-0.895906078487	5.482655175072	-0.803601091632
C	-0.559412677554	5.640859751842	1.599619395161
C	-1.102383664466	6.856632471075	-0.841916617579
C	-0.778559874832	7.013624836989	1.514826486357
H	-1.310692517848	7.326064281915	-1.798148126174

H	-0.738003371748	7.605488221233	2.423379336059
C	-1.048506618870	7.642836479488	0.305838277759
C	-4.704284652821	-0.790598366178	0.572287888818
C	-5.479568242434	-0.801624570604	-0.596678891227
C	-5.311633337680	-0.983316926684	1.819464404321
C	-6.850881195695	-1.001666709253	-0.496098879268
C	-6.690043618896	-1.178289311722	1.874637295395
H	-7.444662640854	-1.009687896120	-1.404624818507
H	-7.157086288450	-1.322216398638	2.843466491176
C	-7.479358454651	-1.190517777477	0.731878086588
C	0.971062149782	-4.891487708005	0.274664132436
C	0.956870500672	-5.676538410501	1.433968244798
C	1.226190125909	-5.483336344618	-0.972560131123
C	1.201741463446	-7.043635274226	1.326035345563
C	1.461250933916	-6.851472670101	-1.034316424834
H	1.195066158332	-7.646532047677	2.228151355261
H	1.655329372127	-7.303408847396	-2.001826564712
C	1.456117150106	-7.652757034451	0.104172307240
C	-4.502137633897	-0.997116462431	3.076285631192
H	-5.140720713708	-1.000867513551	3.957417921996
H	-3.864130909983	-1.881842692693	3.129837231870
H	-3.833778244953	-0.137243747225	3.145033497747
C	-4.838697507194	-0.611482023104	-1.932560363969
H	-4.360790425433	0.367112535489	-2.021897799556
H	-4.053642654159	-1.350394712315	-2.102868539444
H	-5.563702718337	-0.701452928532	-2.739200523692
C	-8.956804304412	-1.401839466788	0.809687843507
H	-9.259157791724	-2.301618357072	0.271107612291
H	-9.294729762445	-1.502153111347	1.839378804038
H	-9.502828269344	-0.572432217701	0.357584585153
C	1.719339822344	-9.120604030484	0.006556091124
H	1.002576593022	-9.611132836872	-0.653891480340
H	2.709036817442	-9.320917835844	-0.407259065635
H	1.661754878554	-9.604897090693	0.979512262813
C	0.671221253002	-5.070755775021	2.770249878817
H	0.870228556037	-5.776088675910	3.574664669863
H	1.264929732278	-4.173723437064	2.950657064292
H	-0.374842126127	-4.767672213763	2.854127882167
C	1.247096839782	-4.661358112462	-2.221713578605
H	1.386637961141	-5.285174658648	-3.102331020420
H	0.318939076455	-4.100950987697	-2.350705145227
H	2.053705027464	-3.924527299030	-2.206503908564
C	5.025421062014	1.029213948514	2.984228893708
H	4.320477364507	1.860638432475	3.050517344783
H	4.442424736442	0.119667605726	3.140454931818
H	5.726164467454	1.124944070418	3.811143992468
C	4.991682402574	0.492636002661	-2.027155520524
H	5.627070418487	0.678511737488	-2.891010173685
H	4.656502862230	-0.546289987979	-2.085657211052
H	4.094430223859	1.108514656174	-2.125880342097
C	9.284468177278	1.468495712600	0.368075543795
H	9.811187747434	0.648379212624	-0.122060143591
H	9.521526940863	2.370922098743	-0.198218715878
H	9.702203190137	1.584978423695	1.366342690635
C	-0.270697027036	5.005906561253	2.923074876240
H	-0.934273212178	4.162366927032	3.121093711006
H	0.747426081058	4.613531858453	2.970129489893
H	-0.384258419516	5.720912209731	3.735438027858
C	-1.276489815440	9.118082282738	0.231773801444
H	-1.200838732286	9.586362013018	1.211333778579
H	-0.551267445357	9.600649941806	-0.425457444432
H	-2.263032815272	9.349268737510	-0.173239884354
C	-0.984170816105	4.671184309995	-2.057465657619
H	-0.139573269425	3.986774418719	-2.168085176233
H	-1.883427565251	4.050267477443	-2.065511718717
H	-1.021037845102	5.309755582865	-2.938203153244
C	0.547073554500	-0.883206816171	-2.341627119381
C	-0.743615465656	-1.275865424169	-2.963437933872
C	-1.506594285155	-0.431090863876	-3.795503017186
H	-1.119810577966	-2.278721973900	-2.783465571755
H	1.238101175586	-0.502565733818	-3.108016352527
H	1.024203531072	-1.759128420072	-1.891520706063
H	-0.317528616701	1.357507011692	-3.508781533755
C	-1.177050078862	0.929646290660	-4.010059935279
C	-1.946342946285	1.718806360596	-4.839218653180
H	-1.679207240111	2.758326072083	-4.985312633185
H	-3.658373731949	1.818717015337	-6.138371343919

C	-3.060115721044	1.191969698137	-5.489486899009
C	-3.398002286797	-0.146269516469	-5.301382807796
C	-2.642347936813	-0.942095921145	-4.468454755146
H	-2.907924975663	-1.982318533648	-4.318258583101
H	-4.260585060470	-0.562426872258	-5.807091760535
O	-1.015230009288	-2.090547280567	3.748157597354
H	-1.393773162816	-2.640987754090	3.052761790776
H	-0.510985986358	-1.415736145953	3.259158614453
O	1.817186620681	2.233469597214	-2.589823639859
H	2.162102557461	2.881083734718	-1.962781015038
H	1.331647700182	1.595941928686	-2.030777584637

137

$^{3}\mathbf{E_{w2}}$ E_{tot}(UM06L-D3(Dichloromethane)/def2TZVP/W06) = -3093.14561066 $\langle S^2 \rangle$ = 2.0345

Ru	-0.044409284551	-0.038076511328	0.259150490394
O	-0.096006486731	-0.097362400081	2.016144621751
O	-0.044509459812	0.075486052694	-1.819155255536
C	3.349551426508	0.530835671213	0.055217180219
C	-0.618883116710	3.367347614960	0.153336439920
C	-3.442499109988	-0.604492616481	0.035719637187
C	0.528646078852	-3.444504596704	0.106594481385
N	-1.720168393736	1.161275442582	0.131534063263
N	1.144811346254	1.638696300306	0.175641506668
N	1.626430689242	-1.234428368391	0.124229601669
N	-1.234148245115	-1.712569631481	0.088549646912
C	1.644280933571	-2.603105146500	0.104805555286
C	2.922002284904	-0.800518772984	0.076465113721
C	2.510788979263	1.650071732603	0.096655788422
C	0.712016084622	2.936320145055	0.151755461207
C	-0.803550658125	-3.014113427300	0.099524926940
C	-2.602117558826	-1.723756676067	0.057604487441
C	-3.014165492856	0.726975120563	0.057352909367
C	-1.736518406137	2.527182917412	0.123579564750
C	3.005874715127	-3.047479302254	0.053681820809
C	3.790361947320	-1.940578770573	0.035595816141
C	2.956604968508	3.010925412485	0.026081636157
C	1.851803531649	3.801122175257	0.067073268634
C	-3.097631133860	2.973844186769	0.046491634840
C	-3.881998079101	1.867876135771	-0.000155936930
C	-1.945823454414	-3.877191164493	0.086754015753
C	-3.050554492825	-3.084646975188	0.061536131351
H	3.316010457490	-4.080408166638	0.032131486076
H	4.867314847462	-1.893311374529	-0.004098102210
H	3.987923718555	3.319168395714	-0.044378996107
H	1.807460904393	4.878389830215	0.033257034323
H	-3.406445682356	4.007191809839	0.021961425664
H	-4.957752106906	1.820648424010	-0.066374895405
H	-1.904860282540	-4.955091973501	0.091331735372
H	-4.084003402353	-3.393221160473	0.043438860600
C	4.816101702094	0.777110538851	-0.059131120771
C	5.572709668623	1.074801053294	1.081035047729
C	5.433853569610	0.706801301949	-1.317597849939
C	6.939554607605	1.303991108748	0.941576328549
C	6.799944331085	0.946538296928	-1.410932986560
H	7.523740018120	1.530637159373	1.827508064905
H	7.272745298423	0.896484323847	-2.386732179556
C	7.573518495085	1.248319945548	-0.293514541813
C	-0.860496652871	4.839643749913	0.162495165167
C	-1.017141794203	5.534547395996	-1.046013960398
C	-0.922939311129	5.524746516059	1.381998158076
C	-1.230698952467	6.907527667912	-1.010781482302
C	-1.141327676878	6.900602679608	1.371451199213
H	-1.349215184628	7.441358823596	-1.948469729119
H	-1.191034966175	7.428389020796	2.318307909289
C	-1.296119225206	7.612313995329	0.188384312614
C	-4.912413793927	-0.860214472587	0.005113831299
C	-5.556750597164	-1.139137676205	-1.210009616990
C	-5.643970558933	-0.836892833390	1.199476338756
C	-6.924440710241	-1.387258451960	-1.206899907221
C	-7.012400756068	-1.094599930000	1.156748615639
H	-7.417966625813	-1.600594228788	-2.149848725282
H	-7.574335952899	-1.082815370307	2.085016356453
C	-7.673584752829	-1.370759744818	-0.033412669249
C	0.773073300315	-4.915693364132	0.080264167049
C	0.936034920197	-5.618271990927	1.279953447770
C	0.835857529548	-5.591793333432	-1.147640598435
C	1.159705569422	-6.992320152103	1.230778523759

C	1.061656933206	-6.963208153528	-1.151031631096
H	1.284319184248	-7.534285450614	2.162586622177
H	1.110468141137	-7.482213124454	-2.103091446533
C	1.226493629529	-7.685006543782	0.028300124393
C	-4.975503036477	-0.539404108100	2.504047360252
H	-5.646923722692	-0.721628182811	3.340992600361
H	-4.075317240988	-1.139888843090	2.652603433104
H	-4.655925051401	0.503834364368	2.561124290003
C	-4.790203946453	-1.164222729112	-2.494242011074
H	-4.257893256277	-0.225551984985	-2.657822307368
H	-4.032931942658	-1.952100308975	-2.501244852555
H	-5.447894057790	-1.329394693772	-3.345825822501
C	-9.144918680652	-1.632253021969	-0.061848492051
H	-9.378906221332	-2.551617800990	-0.599936356939
H	-9.557721182262	-1.715564909428	0.941825615649
H	-9.680559957078	-0.829243193701	-0.572027535427
C	1.468899847677	-9.159477429014	-0.007001716271
H	0.656762722018	-9.685491507979	-0.511431110340
H	2.381286644946	-9.400368292155	-0.555147366259
H	1.563573541714	-9.575308611053	0.994337097665
C	0.870378026617	-4.910232541993	2.595321006105
H	0.983775405444	-5.605707786818	3.424484155133
H	1.652283370460	-4.153236029424	2.685625278252
H	-0.076950908178	-4.383192642552	2.726411091079
C	0.664290486644	-4.852936578316	-2.437703977969
H	0.757007975410	-5.522237069566	-3.290766409377
H	-0.313930389240	-4.369403834857	-2.498709592357
H	1.408801454817	-4.061656201624	-2.551225559976
C	4.932497050821	1.143597240836	2.431348867213
H	4.232657861802	1.978664439751	2.504905349869
H	4.358260936819	0.242359159028	2.653011125475
H	5.677544686340	1.269120926174	3.214571044256
C	4.644844328347	0.366640404294	-2.542789507991
H	5.225099643366	0.544473068036	-3.446399855572
H	4.350383926050	-0.686216148848	-2.546549456368
H	3.719558338700	0.943276836426	-2.613697897572
C	9.039205398285	1.509513375098	-0.426718729036
H	9.547163319501	0.690792487479	-0.938242228930
H	9.230622731931	2.408768661112	-1.015221637097
H	9.513365295608	1.643913226089	0.543767849795
C	-0.756445716913	4.798223705292	2.678906242405
H	-1.480082233198	3.987852768699	2.785054056687
H	0.230828893560	4.338738637963	2.759672797185
H	-0.879564650773	5.470237379116	3.525836634810
C	-1.522800056302	9.089745260163	0.194609943257
H	-1.591855129109	9.481538047070	1.207714838951
H	-0.713230857392	9.618782805270	-0.311332972994
H	-2.441653358549	9.355528291614	-0.330285430287
C	-0.965054230880	4.809762204465	-2.352662252665
H	-0.047778116546	4.227453118024	-2.465746539894
H	-1.786440858713	4.095683612917	-2.442350891646
H	-1.033317153028	5.499272925092	-3.191986561353
C	0.062600257608	-0.981533836636	-2.657091954430
C	-0.657120852834	-0.968261707171	-3.923987460814
C	-1.605085792354	-0.061748623553	-4.407282320184
H	-0.429516162414	-1.819205337076	-4.561069502347
H	1.125386457895	-1.193367984973	-2.918414072475
H	-0.220139682759	-1.951980023343	-2.187457646912
H	-1.518202342949	1.365410751373	-2.783923795781
C	-2.008037375846	1.112211207989	-3.713496916522
C	-3.014280104301	1.903110452838	-4.223425348339
H	-3.323819746299	2.789584439430	-3.684929641123
H	-4.422196574491	2.207323838953	-5.815304961633
C	-3.636382434612	1.573459996012	-5.424460633596
C	-3.247158873487	0.436156061856	-6.131444655800
C	-2.247346449067	-0.365248614542	-5.637791426026
H	-1.938779762595	-1.254503032695	-6.173768239355
H	-3.731928669931	0.187655420730	-7.066256636541
O	-1.654036699915	-2.100685077613	3.252428015115
H	-1.964555906067	-2.650408525513	2.523347539196
H	-1.106779101860	-1.418225788711	2.816588252002
O	1.444209136476	2.116642544921	-3.030643255752
H	1.895322482023	2.648748423025	-2.363689316575
H	0.929885812189	1.467935832253	-2.508686551345

137

$^{3}\mathbf{F}_{\mathbf{w2}}$ E_{tot}(UM06L-D3(Dichloromethane)/def2TZVP/W06) = -3093.16955723 $\langle S^2 \rangle$ = 2.0127

Ru	-0.100047488555	-0.152308707128	0.170079373443
O	-0.107444240995	-0.200029369670	1.925363717979
O	-0.048683753230	0.018299200085	-2.168454034197
C	3.294760345139	0.449387487222	-0.019293641457
C	-0.707759732418	3.236300867167	-0.077553722585
C	-3.500819869182	-0.758888810674	0.080659743241
C	0.503738029510	-3.547256177264	-0.040284977183
N	-1.785401244010	1.016380760763	-0.003588995635
N	1.073770895208	1.527855861158	0.009261040830
N	1.578038640687	-1.323922994710	-0.041063462330
N	-1.277382339180	-1.834341700712	-0.012829619200
C	1.609245379537	-2.693441387282	-0.077271578991
C	2.874703853618	-0.882718817747	-0.058779675761
C	2.443338763022	1.558813090457	-0.000018873434
C	0.627688624456	2.822461066713	-0.038337281670
C	-0.831069581123	-3.132206321269	0.002677389917
C	-2.647625700501	-1.867451848632	0.067840231684
C	-3.082770398348	0.574307917746	0.005365344793
C	-1.815915429059	2.384290171826	-0.074605457206
C	2.973822546875	-3.126499485337	-0.136296006765
C	3.750805113181	-2.014965379937	-0.119538661944
C	2.874890154466	2.924190967716	-0.044604480408
C	1.759041198466	3.700570091378	-0.071369238145
C	-3.180986561457	2.815954774921	-0.131471767184
C	-3.958803903137	1.705111676083	-0.079274957006
C	-1.958555149380	-4.008878254517	0.088830097385
C	-3.073796689490	-3.232254627321	0.135897664675
H	3.291332681092	-4.156419702919	-0.180489685068
H	4.827567615323	-1.958934488709	-0.150148341024
H	3.904643933398	3.245066322375	-0.061890430191
H	1.703079400863	4.776991367055	-0.113952871022
H	-3.498617385948	3.844304491791	-0.204142123000
H	-5.035957930974	1.648871323178	-0.099512275411
H	-1.901922964075	-5.085829598504	0.112151704259
H	-4.100724316371	-3.555030108924	0.203709197643
C	4.763395978269	0.709645603862	-0.010119558006
C	5.414827094557	0.964763627090	1.203289785737
C	5.488405479716	0.696447709697	-1.211506170465
C	6.786273651647	1.208067528637	1.193563167322
C	6.855155444721	0.948171573309	-1.174836137940
H	7.288658474708	1.401147404337	2.135835514594
H	7.411615164894	0.942104523013	-2.106818580016
C	7.525729336000	1.207579751724	0.017357858927
C	-0.966763090493	4.704857157233	-0.121064999863
C	-1.092787875141	5.359620511640	-1.355368674396
C	-1.078157484455	5.424876021090	1.074419746748
C	-1.326259558543	6.729952881050	-1.368213781357
C	-1.313637634677	6.796495932149	1.014885456638
H	-1.422522152047	7.233399459322	-2.325015646236
H	-1.400406673000	7.351980923905	1.943041040848
C	-1.439801194388	7.469579053217	-0.193989465278
C	-4.964127765050	-1.020011653350	0.199156638078
C	-5.706127843031	-1.441127653840	-0.915214076938
C	-5.593497486333	-0.851451555516	1.439952608572
C	-7.068025207461	-1.676671711395	-0.769074506703
C	-6.959391301031	-1.104685131270	1.541899269260
H	-7.638505729096	-1.994903196891	-1.635877439899
H	-7.441935461417	-0.979545387648	2.505730608019
C	-7.716759923800	-1.515065887705	0.452153357290
C	0.766962317315	-5.015783206068	-0.028524311966
C	1.001961303492	-5.668871787770	1.187655190872
C	0.776594794571	-5.738369490519	-1.230745538626
C	1.240267225556	-7.041366652481	1.179475134199
C	1.021011189883	-7.106087039330	-1.193359809103
H	1.417739578145	-7.545491554920	2.123868143643
H	1.028442677130	-7.660868566933	-2.126266319324
C	1.254985694631	-7.779617919024	0.002657546990
C	-4.821582953468	-0.409426968009	2.643072091761
H	-5.408772237141	-0.529940410400	3.551534081460
H	-3.891049317885	-0.968727042334	2.763078770451
H	-4.539068776664	0.643767807457	2.574954614934
C	-5.049860586470	-1.639967510948	-2.244668053824
H	-4.422887119039	-0.789154500921	-2.516348257973
H	-4.396434653870	-2.515886225472	-2.246564105814
H	-5.786856307210	-1.780433604644	-3.033061039788
C	-9.182818394937	-1.776507256556	0.578708666024
H	-9.433001995383	-2.797731203246	0.287004832574

H	-9.531105851665	-1.626486366829	1.598845765352
H	-9.762243096330	-1.118265441883	-0.070917636044
C	1.517894084281	-9.250953528295	0.011531534938
H	0.710498466498	-9.804270213349	-0.470379263724
H	2.430241735307	-9.495335492209	-0.535473390613
H	1.627112460625	-9.633593943784	1.024568636930
C	1.002233092254	-4.910512788417	2.476461151992
H	1.104142081477	-5.580093549740	3.328188413697
H	1.824914965305	-4.193065962616	2.519360281417
H	0.087445930440	-4.329265073337	2.610594151537
C	0.531417968213	-5.051881277053	-2.537189824375
H	0.603722029244	-5.749304915767	-3.369372446277
H	-0.460289249266	-4.595042967221	-2.572577050055
H	1.251099739879	-4.248930856517	-2.710815801926
C	4.658739457498	0.977978595378	2.494236544160
H	3.958379936785	1.814572660651	2.543262946054
H	4.063500290693	0.072569913068	2.625571394360
H	5.332999262956	1.064067806161	3.344002883070
C	4.810734023509	0.407211717840	-2.513367489606
H	5.475020327669	0.600198024334	-3.353779622016
H	4.498951276426	-0.638469431919	-2.577331937759
H	3.906716388698	1.004972903722	-2.649889975296
C	8.995161778812	1.481167241408	0.023351763842
H	9.553500392011	0.679420630080	-0.462000612223
H	9.231163270978	2.396887398445	-0.521875800803
H	9.379452736957	1.591439170551	1.035691434540
C	-0.946377649788	4.739688800620	2.397621958989
H	-1.680690126372	3.940260343361	2.514400054157
H	0.034078434773	4.273844873005	2.515351695780
H	-1.081938673110	5.440003188104	3.219330919972
C	-1.687892513164	8.942854374760	-0.240705585510
H	-1.781971118197	9.366565376443	0.757428557742
H	-0.877099733391	9.467306287213	-0.749322377584
H	-2.600784549249	9.177037665208	-0.790557615994
C	-0.989783434546	4.596765630513	-2.637229905116
H	-0.046626287631	4.050754734582	-2.719088502775
H	-1.779722899521	3.846683449144	-2.719368195569
H	-1.070479986748	5.256342216388	-3.499374909873
C	-0.075549773748	-1.115094125098	-3.047735990311
C	-1.246516265734	-0.254038810445	-2.970883699300
C	-1.617184063080	0.776581266707	-3.959682389807
H	-2.066858664978	-0.607673313934	-2.351616007556
H	0.611140476739	-1.037122055138	-3.884299455665
H	-0.094716046066	-2.076325498736	-2.544053332077
H	0.230413553709	0.739795015370	-5.063095719751
C	-0.751211692922	1.187363392851	-4.971472298514
C	-1.136794390785	2.175131113796	-5.861879131682
H	-0.455265082215	2.489197656318	-6.641979852830
H	-2.692660414732	3.526351530183	-6.461349278415
C	-2.393565402476	2.757752564691	-5.760034384407
C	-3.261741668117	2.356261238455	-4.755962938107
C	-2.871183488723	1.375521943675	-3.857446896318
H	-3.537254866432	1.072870195973	-3.056905378136
H	-4.239418361289	2.811969497161	-4.664680869650
O	-1.579232471943	-2.259393116797	3.191036789822
H	-1.965672018450	-2.779207832199	2.476152300284
H	-1.076914294017	-1.559332458357	2.730562725143
O	1.756228370992	2.175031311939	-3.148483480891
H	2.090289771053	2.718287268760	-2.422840530440
H	1.142479690289	1.562161914862	-2.714855217244

137

$^{3}\mathbf{TS_{w2}(F,G)}$ E_{tot}(UM06L-D3(Dichloromethane)/def2TZVP/W06) = -3093.15835267 $\langle S^2 \rangle$ = 2.0130

Ru	0.287587282706	0.005330562982	0.307495877257
O	0.194323111036	-0.049785907900	2.051976259634
C	3.725374694186	-0.070634345592	0.524947913334
C	0.399741749985	3.440539028608	-0.054523783423
C	-3.147977397020	0.090241985019	0.067912202858
C	0.242241374155	-3.411160593158	-0.190684292571
N	-1.108748771697	1.483450523993	0.042225017817
N	1.784919722542	1.425117473986	0.261786624713
N	1.716180527458	-1.454153778128	0.116341556409
N	-1.176592620745	-1.396508559639	-0.006674911663
C	1.490101449595	-2.795720269542	-0.059109767658
C	3.066384671430	-1.285632739280	0.305914380069
C	3.120330540695	1.188399044359	0.449344853260
C	1.614504717307	2.778933458002	0.147190718049

C	-0.989194908431	-2.750183190230	-0.138283431567
C	-2.529345775266	-1.163855465378	0.014636851913
C	-2.471103283414	1.313369787789	0.024099243156
C	-0.857388533650	2.826750844321	-0.085201648863
C	2.743858024049	-3.485899752896	-0.020805016986
C	3.708046205391	-2.562369969926	0.223472592440
C	3.812239574239	2.441601085710	0.482743345937
C	2.890169398925	3.416929376784	0.277265558464
C	-2.104281648312	3.518231550900	-0.215966634926
C	-3.094657108767	2.593340266581	-0.123523688047
C	-2.269239964929	-3.388501790184	-0.197386153662
C	-3.214505333257	-2.416102137155	-0.087820177392
H	2.865707885510	-4.549762845322	-0.149218738073
H	4.769407579409	-2.725059170280	0.326372258428
H	4.875941090188	2.554852964307	0.621566864264
H	3.055175150040	4.481425666836	0.221203132134
H	-2.205834300011	4.584121013388	-0.347047328860
H	-4.159803533626	2.757414341658	-0.171451550713
H	-2.423172690068	-4.450615181757	-0.307609061236
H	-4.286855075790	-2.532531068875	-0.097185611820
C	5.175762335920	-0.117998914686	0.872710503483
C	5.539451299802	-0.162413574442	2.228267434184
C	6.161779242252	-0.111566509419	-0.120099194123
C	6.888032232845	-0.200744982424	2.561780596979
C	7.501485146838	-0.150157767925	0.259161419312
H	7.164777750508	-0.235706984961	3.610686634881
H	8.262751571412	-0.143792102254	-0.513993989019
C	7.887755771491	-0.195011002628	1.592827326290
C	0.453986750572	4.915183688417	-0.270090266976
C	0.761975098435	5.408708123522	-1.547963508983
C	0.209413632649	5.799576374634	0.787238612676
C	0.804723266459	6.784061271964	-1.744697524061
C	0.267097825042	7.170078535758	0.545428111891
H	1.038850634316	7.161640109919	-2.734983770502
H	0.081646885480	7.852973396736	1.368052244897
C	0.558566648761	7.684602717006	-0.711992308061
C	-4.637754060514	0.127658853325	0.156448017975
C	-5.426834642592	0.174175384539	-0.998934297035
C	-5.243702323230	0.123004862336	1.422912266631
C	-6.813390371887	0.203315896258	-0.869777277821
C	-6.630866434840	0.153772109563	1.505436532945
H	-7.420169330124	0.237158743054	-1.768700839277
H	-7.093902913484	0.149429008440	2.487049529197
C	-7.437227194412	0.191262178234	0.371351564256
C	0.230817463218	-4.890279693354	-0.387029928996
C	0.181012064690	-5.749317887265	0.720936617942
C	0.289209068655	-5.415633616519	-1.683470306479
C	0.198465589129	-7.122608888760	0.508373509223
C	0.305092488579	-6.798427406957	-1.850778425779
H	0.164341728951	-7.783514082815	1.368672205725
H	0.355545052472	-7.201856696077	-2.856831005244
C	0.262474594227	-7.670413090090	-0.770055072121
C	-4.414705360902	0.093809352435	2.666812168052
H	-5.040489532602	0.080782145559	3.556982685439
H	-3.758472516111	-0.778494034280	2.704132073461
H	-3.759114822072	0.965130454893	2.733008136929
C	-4.800483459579	0.210574303696	-2.356155645646
H	-4.210292415929	1.119640315593	-2.496810454365
H	-4.119454449568	-0.628891009148	-2.512375289167
H	-5.553036944024	0.181562379349	-3.141661031556
C	-8.926615426987	0.216351057396	0.492897374690
H	-9.263480251852	1.057515167219	1.100541905552
H	-9.408254289482	0.292917197669	-0.480157435437
H	-9.301256883223	-0.686207035739	0.978885573251
C	0.285471399930	-9.152144903698	-0.964224305720
H	-0.605109217159	-9.622864140677	-0.544603418678
H	0.337823277618	-9.419344404308	-2.017961980027
H	1.141105526283	-9.606468676838	-0.461998359171
C	0.094809377881	-5.202240095261	2.109351767952
H	0.184876276569	-5.992258333006	2.852183442764
H	0.870227530448	-4.460332235084	2.307548037460
H	-0.858314270956	-4.696915543824	2.279662347831
C	0.311619707672	-4.513141322416	-2.876494772208
H	0.503669147106	-5.071139620913	-3.790867608199
H	-0.645574199136	-4.001235624904	-2.998780234379
H	1.070855806215	-3.732854633774	-2.790458252680
C	4.496701101750	-0.164860434362	3.301162268072

H	3.894430154058	0.745917026641	3.280960438349
H	3.796655925269	-0.994747907659	3.185706531989
H	4.947603232133	-0.242485224918	4.288353509777
C	5.790548812898	-0.064823726833	-1.567296529186
H	6.673267612304	-0.028090345204	-2.202516030976
H	5.210884144949	-0.942868538656	-1.861517365442
H	5.179475419293	0.810388785583	-1.798652605160
C	9.329762725628	-0.235393701713	1.983619567689
H	9.596528334279	0.616414848284	2.611343424223
H	9.560852687687	-1.130895128235	2.562789878944
H	9.982801177525	-0.224519767499	1.112961902815
C	-0.101337838886	5.289535750635	2.158661821401
H	-1.048605794530	4.746841557235	2.183690165921
H	0.660633600633	4.592540126581	2.512057015787
H	-0.169384591550	6.104948229637	2.876008124964
C	0.598291487203	9.158269267098	-0.958326532986
H	0.528595390071	9.723475213840	-0.030761021639
H	1.516298219823	9.455413188961	-1.466586181769
H	-0.227277966302	9.474940391243	-1.598862820540
C	1.046729175683	4.477535761455	-2.684469693459
H	1.974500867447	3.921051621068	-2.529806185420
H	0.259755310370	3.730840546701	-2.806573939402
H	1.142465533720	5.019588519673	-3.623307761691
O	-0.072295817725	-0.292818837022	-2.613488839686
H	-1.628506701801	-1.741149064605	-2.656520602658
C	-1.058929486390	-1.064224734520	-3.287588240836
H	-0.739151341696	-1.467669845768	-4.243917414177
C	-1.232458897826	0.377260145492	-3.141904039315
H	-1.911818248716	0.709420438896	-2.362468915384
C	-1.023661225397	1.351031527701	-4.231707828817
H	-2.661214158211	2.569423399318	-3.580890429656
C	-1.873456978232	2.450840167587	-4.316963645909
H	-2.381177513036	4.239242170341	-5.381067800305
C	-1.713683533831	3.388689540460	-5.325575036406
C	-0.691816268177	3.241852854595	-6.251672097246
H	-0.558284484523	3.976757364035	-7.035266718760
C	0.165671327717	2.151892173447	-6.167195317089
H	0.968094192227	2.036766322264	-6.884751027601
C	-0.000265307064	1.208555992868	-5.166594427971
H	0.673009756167	0.361636400070	-5.104132316477
O	2.736979387541	-0.117283799837	-3.038463155489
H	1.774327145794	-0.143256702048	-2.924148938423
H	3.077744900625	-0.134534271141	-2.136007971509
O	-1.498043920733	-2.044737745131	3.146872932310
H	-1.882762697890	-2.499000710874	2.387985718645
H	-0.927008535913	-1.362437868735	2.746391537661

137

$^{3}\mathbf{G_{w2}}$ E_{tot}(UM06L-D3(Dichloromethane)/def2TZVP/W06) = -3093.15579949 $\langle S^2 \rangle$ = 2.0129

Ru	0.187761274932	-0.112364122705	0.926823766775
O	0.188037667182	-0.048548157130	2.672926275426
O	-1.554765088141	0.096519616335	-2.545571232317
C	3.561933053120	0.535358102964	0.686018010346
C	-0.486110941308	3.239172379957	0.416564645447
C	-3.201980908833	-0.772254417921	0.926997028747
C	0.831371666892	-3.494561084302	0.569932843752
N	-1.522599226513	1.021533560024	0.719228504982
N	1.322275531466	1.571885981778	0.634755655503
N	1.876438981202	-1.265694433275	0.752434656259
N	-0.963433870373	-1.812760557404	0.782563468757
C	1.926102752593	-2.629600791767	0.637340642382
C	3.164815173377	-0.804429580764	0.717120990168
C	2.693346676513	1.628433428823	0.622335699719
C	0.855133582322	2.851367795049	0.471725021274
C	-0.506346759882	-3.101851514396	0.664039226962
C	-2.333544242810	-1.866286714681	0.859151695449
C	-2.809326964487	0.564467521351	0.821557017617
C	-1.578774371472	2.379760380670	0.556343662171
C	3.296127077034	-3.040967118679	0.556899686339
C	4.057997755064	-1.920548936813	0.618011960722
C	3.098953604616	2.991349020504	0.459978260089
C	1.970862989916	3.740027236624	0.354698881552
C	-2.950739318029	2.791486236674	0.554239699414
C	-3.705721977667	1.678907593451	0.737851298159
C	-1.626444664713	-3.992094527687	0.662218179161
C	-2.746714083132	-3.234588079040	0.800608468491
H	3.627229542995	-4.063084453568	0.461516902006

H	5.133435977100	-1.847413484142	0.578728623377
H	4.122918806044	3.327855954045	0.416581487415
H	1.896014783851	4.806873059826	0.213718138769
H	-3.288780452946	3.809136696522	0.437169662677
H	-4.780743311849	1.610644397393	0.794180623547
H	-1.560572478608	-5.064948839288	0.570913968692
H	-3.771066009609	-3.570502905889	0.840357902825
C	5.026240669991	0.817478876635	0.644403313965
C	5.742038122264	0.956214999358	1.839077682560
C	5.682284747975	0.938402843342	-0.590016465290
C	7.109244409876	1.216527867514	1.778887498590
C	7.047687096399	1.197451234192	-0.604154779330
H	7.661283929656	1.324516106922	2.706851148577
H	7.551888359259	1.288410115680	-1.561062382557
C	7.782437859387	1.340551191213	0.569995058576
C	-0.773874249076	4.675992884777	0.139928298404
C	-0.938889028750	5.093614676116	-1.190001477771
C	-0.877877205090	5.598033925257	1.186991182313
C	-1.205771916858	6.433189094757	-1.446179482460
C	-1.147557850986	6.930991143926	0.885178604783
H	-1.331859087752	6.753096151740	-2.475698000142
H	-1.228395904140	7.644242491557	1.698987294274
C	-1.315016381918	7.370658286094	-0.422162987086
C	-4.657772977008	-1.054478634627	1.094862649748
C	-5.515164538146	-1.072239077359	-0.015896476565
C	-5.165459500469	-1.300854024090	2.376999252945
C	-6.866441830971	-1.332320679593	0.178244896041
C	-6.527368333822	-1.553233751615	2.526516068441
H	-7.523862908644	-1.348123518722	-0.685286118346
H	-6.916561156252	-1.737972316099	3.522453444655
C	-7.396277213817	-1.573427842709	1.443205100603
C	1.107381221928	-4.937520538178	0.309610953521
C	1.207304278085	-5.854923382105	1.361103265527
C	1.263175964430	-5.366358736472	-1.018659011238
C	1.468356513603	-7.191554857740	1.066997086071
C	1.518872488674	-6.709531780827	-1.266718054375
H	1.549112761207	-7.899309834673	1.885603165098
H	1.636957612183	-7.037067320742	-2.294811062864
C	1.627870802056	-7.641165899948	-0.237563269457
C	-4.266442009299	-1.312334635821	3.572158955953
H	-4.839805925304	-1.321923194188	4.497120663279
H	-3.621513571181	-2.193938345474	3.577650232365
H	-3.598682393792	-0.449475962163	3.594203612999
C	-4.987855081411	-0.818768146512	-1.392276767283
H	-4.602884847455	0.197875969406	-1.494238425999
H	-4.159335705467	-1.487175422000	-1.632177474446
H	-5.762326553144	-0.957446680562	-2.144449357224
C	-8.856217538386	-1.838738509123	1.622432714115
H	-9.190769046744	-2.670723394702	1.001125520839
H	-9.097758369547	-2.075425413088	2.656945920706
H	-9.456111685579	-0.974286362831	1.332032692018
C	1.908920254396	-9.078543365048	-0.535823445643
H	1.147505085462	-9.506667735115	-1.189769821468
H	2.863132083284	-9.199528828058	-1.051423031379
H	1.943876399327	-9.678070454316	0.371869083120
C	1.020456034575	-5.419698507934	2.779121306007
H	1.284597756791	-6.213963931142	3.474592275692
H	1.620810729919	-4.541848238398	3.021354363580
H	-0.018734269888	-5.145482959298	2.976100849961
C	1.162535828744	-4.396483515946	-2.154137302163
H	1.203637136591	-4.908412522274	-3.113576268865
H	0.235135283571	-3.820922621548	-2.117222273441
H	1.973753059055	-3.664996686742	-2.133227124848
C	5.056058339799	0.826194160581	3.161853838025
H	4.239145604226	1.542834341996	3.265819980575
H	4.612918634476	-0.163297599071	3.290514337623
H	5.750146123210	0.988794568904	3.983921983925
C	4.928885562557	0.791882539007	-1.874487756377
H	5.594537890792	0.863451977953	-2.732308762881
H	4.409593054767	-0.166948961450	-1.932831823675
H	4.165915764813	1.566449265652	-1.984027587165
C	9.250710039159	1.616289688059	0.522300372631
H	9.789919490293	0.815706715242	0.013054629194
H	9.467060494018	2.533620678100	-0.027572470891
H	9.673225051867	1.718553982237	1.520095975800
C	-0.701541826862	5.165122016359	2.607624103998
H	-1.402812860545	4.373714650466	2.878427308821

H	0.297777554944	4.762280403362	2.784399797375
H	-0.851507051077	5.995117156612	3.295111206787
C	-1.606534972635	8.804469074644	-0.727685424247
H	-1.629977150591	9.410982920594	0.175745755960
H	-0.857516704272	9.230526084034	-1.397000213746
H	-2.569467351528	8.917433265131	-1.228788002685
C	-0.830718333660	4.114351410666	-2.316918094971
H	0.138422413385	3.609389065307	-2.322930346002
H	-1.585094985058	3.327272096301	-2.242827508508
H	-0.956546875545	4.602438177304	-3.281341068079
C	-1.715412101117	-0.995439563559	-3.440967444619
C	-2.284611055353	0.308851224240	-3.763748084099
C	-1.692796304008	1.227165248140	-4.759734390930
H	-3.351446678033	0.437032733951	-3.600581784892
H	-0.808122244246	-1.313682257796	-3.948547151352
H	-2.355408972403	-1.798251143563	-3.085460764079
H	0.350199296742	0.693363120665	-4.353919442020
C	-0.314028168093	1.312028217769	-4.947690534790
C	0.212993551172	2.185201819750	-5.884348731476
H	1.285639625480	2.244310532770	-6.019455103988
H	-0.215528107662	3.663678915712	-7.381560176737
C	-0.629604345232	2.981940769006	-6.649668095957
C	-2.002200590645	2.904057218264	-6.467870232381
C	-2.529596903085	2.035217092823	-5.523832464796
H	-3.601411201438	1.980678756891	-5.371596374766
H	-2.664682594814	3.526078962164	-7.056392199323
O	-0.665840206336	-2.365365574021	4.024039994455
H	-1.000560101599	-2.942959117744	3.328542654056
H	-0.382704866610	-1.564895718400	3.542799532828
O	1.171656762869	-0.199883893559	-1.972104092186
H	1.713463987395	0.577226888028	-1.785635721133
H	0.298663676845	0.160692617237	-2.208815241040

137

^{3}TS$_{w2}$(G,H) E_{tot}(UM06L-D3(Dichloromethane)/def2TZVP/W06) = -3093.15564235 $\langle S^2 \rangle$ = 2.0130

Ru	-0.040158869999	-0.133126239952	0.608963350941
O	-0.058168229630	-0.076953097092	2.355550505725
O	-1.929131900881	0.052295030077	-2.913146480980
C	3.334944272359	0.519975128062	0.367683251807
C	-0.716827710955	3.218206491497	0.107049800836
C	-3.430072023745	-0.798354113546	0.594437463353
C	0.606876098904	-3.511280473472	0.215872336920
N	-1.751948958237	0.997637978952	0.390784302586
N	1.094177024061	1.553034303317	0.326010949813
N	1.649956192040	-1.282465314727	0.422760558020
N	-1.190370137506	-1.833935719038	0.441108714177
C	1.700512896553	-2.645654174891	0.291877465869
C	2.938635373231	-0.820123447738	0.389043292495
C	2.465023213274	1.612292391006	0.313631761991
C	0.624712954772	2.832525204299	0.165974445467
C	-0.731297691463	-3.121224235406	0.313910703852
C	-2.560196138945	-1.890528544939	0.519386831874
C	-3.038636484538	0.538897951875	0.491290361671
C	-1.809244155141	2.356738989980	0.236365794505
C	3.070538590120	-3.054798804399	0.204796731401
C	3.831918430178	-1.934638164802	0.276953122141
C	2.868286620241	2.976451714518	0.157184745001
C	1.738976440620	3.723514850738	0.053873553097
C	-3.181480709085	2.767028457581	0.234492155342
C	-3.935640326397	1.652735670733	0.411694124282
C	-1.849720063835	-4.013655166227	0.308381261296
C	-2.971105880292	-3.259169164766	0.453735702064
H	3.402136207139	-4.075553252299	0.097366850193
H	4.907220456124	-1.860365507023	0.235870474026
H	3.891662968921	3.314860449495	0.115078221743
H	1.662309011402	4.790702139206	-0.083519813526
H	-3.520562515583	3.784824458205	0.122021117625
H	-5.010660835397	1.583159590562	0.466403040105
H	-1.781838459164	-5.085744981191	0.209830340032
H	-3.994880000727	-3.596943519201	0.492885169072
C	4.798232659183	0.804596111542	0.314878763438
C	5.524187222251	0.949354098640	1.502502825662
C	5.442251826979	0.925151193429	-0.926007486030
C	6.889601405743	1.215653596481	1.429009583777
C	6.806057820489	1.191151711507	-0.953419455632
H	7.449707499955	1.328453155932	2.351543825300
H	7.300858872610	1.283160522853	-1.915115763043

C	7.550867727797	1.340578615166	0.213653547618
C	-1.007817937074	4.655130263339	-0.164637805354
C	-1.190372156037	5.072158425984	-1.492407249094
C	-1.101650876983	5.576510769985	0.883859851273
C	-1.464253625850	6.410918538268	-1.745274725889
C	-1.378653603620	6.908726912240	0.585382842312
H	-1.604729457701	6.730471622018	-2.773040433496
H	-1.452579522706	7.621547113126	1.400218906914
C	-1.563525210975	7.347945594815	-0.719748642555
C	-4.884517439111	-1.082671977123	0.771172560666
C	-5.748644790486	-1.108623459122	-0.334132784404
C	-5.383776293207	-1.323234716401	2.057714358881
C	-7.098312865639	-1.369441155873	-0.130198148954
C	-6.744252587444	-1.577593595578	2.216964879329
H	-7.760988949635	-1.390571866300	-0.989592704331
H	-7.126823459211	-1.758080375072	3.216235972328
C	-7.619875863611	-1.604851810497	1.139274626251
C	0.883854496499	-4.952378516811	-0.055047604902
C	1.004525457448	-5.872621162088	0.991809688177
C	1.018670441695	-5.377099360730	-1.386934153487
C	1.267061778021	-7.207198398423	0.689761165627
C	1.276736217864	-6.718335952993	-1.642886150027
H	1.364051692142	-7.916897541156	1.504916074905
H	1.379438326253	-7.042315455340	-2.673749631235
C	1.407655687050	-7.652321121059	-0.618485286736
C	-4.477198549670	-1.327159879832	3.247171812885
H	-5.044568261983	-1.340206554106	4.175765529244
H	-3.825662285397	-2.203867341581	3.248979381304
H	-3.815849938222	-0.459222462760	3.264460685241
C	-5.228175806861	-0.868900543574	-1.715404192885
H	-4.792458679571	0.126846414775	-1.815372578356
H	-4.437675524803	-1.577058350884	-1.969916389319
H	-6.018055694939	-0.964940030935	-2.458135456001
C	-9.078155894177	-1.871896902777	1.329101102048
H	-9.414901293903	-2.708106857881	0.714676500765
H	-9.312749194222	-2.103162643401	2.366440073839
H	-9.681581467188	-1.010317715692	1.037504912376
C	1.692879756837	-9.086991040495	-0.925594451889
H	0.931289298333	-9.514360177699	-1.579730612631
H	2.646113401854	-9.201375031859	-1.444625014454
H	1.733292332911	-9.691344387646	-0.021343085433
C	0.835454379602	-5.442798497685	2.413614590637
H	1.119478082361	-6.235478043777	3.103024509854
H	1.428895286551	-4.558329042047	2.648520783405
H	-0.203821271870	-5.181407083365	2.627121130230
C	0.894750794167	-4.405672217554	-2.518689096057
H	0.935974563898	-4.914767490852	-3.479644043898
H	-0.040711139378	-3.843585242696	-2.474412246324
H	1.696238049885	-3.663681505084	-2.501760615691
C	4.850412552394	0.820955332363	2.831666178598
H	4.041113788866	1.544690025609	2.945815523955
H	4.399743303024	-0.164865323326	2.961768856715
H	5.553904830589	0.975367097644	3.647292999318
C	4.677988759650	0.768147989854	-2.202930270289
H	5.328288222704	0.881023802239	-3.068038854880
H	4.200834985245	-0.212109771299	-2.270464625876
H	3.879816624837	1.509248878291	-2.289493611819
C	9.017100023533	1.624229773064	0.151798974762
H	9.556108363529	0.825201281974	-0.360090796717
H	9.223322810404	2.541186030282	-0.402567308438
H	9.448068444920	1.731669624188	1.145426169108
C	-0.908368488063	5.143646699590	2.302311978243
H	-1.596976092706	4.342668374051	2.577322314558
H	0.097559971025	4.753736498305	2.470562030276
H	-1.063392535527	5.970588989319	2.992355615304
C	-1.863069648794	8.780938127110	-1.021322784644
H	-1.886536443515	9.385417824432	-0.116527485297
H	-1.118005138056	9.212090708743	-1.691844206341
H	-2.827931821903	8.890307534666	-1.519418877243
C	-1.091190215508	4.092729996920	-2.620068147597
H	-0.109305091859	3.613699527970	-2.654153419823
H	-1.821145844837	3.285716047034	-2.521393440326
H	-1.258276378748	4.575002774531	-3.581093846278
C	-2.060484272540	-1.029728834014	-3.825724791206
C	-2.633422267408	0.273739508600	-4.144837250099
C	-2.028193247571	1.210540126459	-5.115207749462
H	-3.704604367083	0.390638252475	-4.003218528341

H	-1.139050860108	-1.334779168049	-4.315756603040
H	-2.701591193876	-1.841669048059	-3.494571081656
H	0.009311972163	0.670565881010	-4.690719529291
C	-0.646970598856	1.299862045857	-5.282114862904
C	-0.107362298671	2.189227947432	-6.196056461771
H	0.967008355548	2.251034509665	-6.315258891702
H	-0.516077012257	3.693024772073	-7.673461948225
C	-0.939800506146	2.998719628565	-6.959128922655
C	-2.314689841790	2.917331590833	-6.797598061733
C	-2.854684304700	2.031650452929	-5.876559836858
H	-3.928504301144	1.973542469216	-5.740797633375
H	-2.969354000900	3.549237284248	-7.384325384747
O	-0.884513126747	-2.418996557212	3.681752325773
H	-1.216696873650	-2.990682101111	2.980152521861
H	-0.611533500938	-1.609514559023	3.209897181790
O	0.772938413252	-0.243466561557	-2.282550259459
H	1.329044349508	0.535347350594	-2.150546703869
H	-0.094472592614	0.114988134766	-2.544466375290

137

^{3}**H$_{w2}$** E_{tot}(UM06L-D3(Dichloromethane)/def2TZVP/W06) = -3093.16812078 $\langle S^2 \rangle$ = 2.0127

Ru	0.076386973712	-0.092791812443	0.793746772023
O	0.166261586728	-0.034365589903	2.549379588206
O	-2.236507193703	0.493425944445	-2.901833768744
C	3.421083836539	0.651602397900	0.339626704402
C	-0.697059834415	3.255246580379	0.462892899537
C	-3.291751389335	-0.843596059396	0.930082832341
C	0.809993014442	-3.456798055435	0.555549326051
N	-1.668040963156	0.997496837208	0.688700286608
N	1.161455692165	1.630914669166	0.490451847719
N	1.798731878725	-1.195355460080	0.550425626686
N	-1.032037941154	-1.824481539616	0.758703095322
C	1.880477171571	-2.560527354154	0.492885718619
C	3.066134575143	-0.699270243618	0.407777117620
C	2.523593384781	1.722488068973	0.378319371667
C	0.654988207294	2.901705570267	0.424475786548
C	-0.534547755705	-3.099824739290	0.698896335947
C	-2.393089755835	-1.914826742980	0.885999877939
C	-2.939328002417	0.502704156505	0.808039111057
C	-1.763242430707	2.358777900941	0.582327781915
C	3.253718490773	-2.937283823778	0.329424975886
C	3.982471169251	-1.794065578787	0.279268213889
C	2.887351993150	3.102443370830	0.253900157046
C	1.739637373819	3.826554316606	0.280704623719
C	-3.146498098415	2.732691139268	0.622888612703
C	-3.868259941855	1.593224791588	0.769412157101
C	-1.622539378851	-4.025793548262	0.802923644617
C	-2.763244821934	-3.297390557313	0.929398689264
H	3.609789056048	-3.952973654809	0.257037311486
H	5.049639275397	-1.693905391417	0.157524613128
H	3.897528830354	3.466681469906	0.150828735942
H	1.631026434770	4.897177685928	0.205406615026
H	-3.513574537050	3.744570007018	0.551625517368
H	-4.940063609950	1.491760732942	0.838248113681
H	-1.522642393688	-5.099743949103	0.780990797301
H	-3.773502899023	-3.662792030485	1.026970455433
C	4.862834338606	0.971667961471	0.131258985590
C	5.727512979409	1.089507844935	1.225160453314
C	5.347327586330	1.149883620649	-1.173805867568
C	7.069369891478	1.384554411643	0.994186405349
C	6.693346227788	1.441333999049	-1.359567306264
H	7.737138621142	1.476963405853	1.844545128914
H	7.064526008865	1.575739638873	-2.370694554914
C	7.574195685026	1.563314809438	-0.287791821306
C	-1.028779446422	4.705333248106	0.349491383757
C	-1.211154378805	5.280726011584	-0.916818229155
C	-1.157460075613	5.488162821328	1.502864915122
C	-1.521477191921	6.632616482951	-1.005326538973
C	-1.469646328606	6.839173828033	1.368931343354
H	-1.661780990537	7.073147891747	-1.987431194860
H	-1.568871056595	7.443027766463	2.265054177169
C	-1.656004084161	7.432476209626	0.126494916766
C	-4.736868394006	-1.168969831331	1.109984317081
C	-5.560202916255	-1.378634912264	-0.005943125869
C	-5.265214651514	-1.269945860655	2.403053018744
C	-6.899180138139	-1.694234092171	0.192998627947
C	-6.613087609693	-1.587023261109	2.556969359025

H	-7.531432057581	-1.858326780863	-0.673956642481
H	-7.018916913702	-1.665949228183	3.560307391547
C	-7.447681640566	-1.805267328473	1.468004233643
C	1.121514081495	-4.907862857948	0.402992224749
C	1.315693996356	-5.718276853517	1.526978895972
C	1.218415341305	-5.455082477469	-0.886329338332
C	1.610983990959	-7.067474739491	1.342693517640
C	1.512069369977	-6.806367485758	-1.024523219075
H	1.764945504822	-7.691884361641	2.216728449786
H	1.585433502195	-7.225316508984	-2.023211871332
C	1.714368068594	-7.632655555619	0.078037880733
C	-4.402671843519	-1.037947357475	3.602244632978
H	-4.962838559743	-1.179361899356	4.524538238247
H	-3.540489577901	-1.708131239750	3.624995857159
H	-3.993824610721	-0.025031046185	3.612591712266
C	-5.011588006115	-1.250116358963	-1.391265810587
H	-4.682961391030	-0.228666443739	-1.593497132742
H	-4.139042763206	-1.888633040907	-1.544329778216
H	-5.757382570799	-1.514765055261	-2.138738032262
C	-8.891253494638	-2.147228476388	1.651268499524
H	-9.135421392673	-3.107018752157	1.193004165631
H	-9.160316732375	-2.203570443653	2.704365739181
H	-9.539693012065	-1.405861561208	1.181282687825
C	2.029260305473	-9.082722787523	-0.102125698278
H	1.224930696818	-9.603044876297	-0.625021752032
H	2.930365171118	-9.224545296338	-0.700875253366
H	2.181717981213	-9.582626698867	0.852592808404
C	1.189927800297	-5.156707500040	2.906930335922
H	1.531705599337	-5.867648817225	3.656690101340
H	1.759026058580	-4.233881634362	3.028252112527
H	0.152046464341	-4.907931191948	3.140835710464
C	1.013859597047	-4.601009995289	-2.098429341810
H	1.038373324587	-5.197004790443	-3.008654787367
H	0.058460992932	-4.073092519530	-2.066349713997
H	1.785032834433	-3.831848112800	-2.184989359470
C	5.224863151100	0.902090209837	2.621243101996
H	4.398330615497	1.578360594990	2.846871361931
H	4.844716304244	-0.109029423837	2.780191445333
H	6.011997361301	1.079122471037	3.351439957572
C	4.429168079012	1.032021674042	-2.350068117881
H	4.973012442488	1.119543436800	-3.289009145078
H	3.900472789009	0.076131225380	-2.355837369129
H	3.661776146539	1.810226442845	-2.338409111361
C	9.018049604729	1.873974295392	-0.518172708808
H	9.502648309601	1.097282472493	-1.112341753812
H	9.143120677510	2.807919169646	-1.068328729002
H	9.564434836890	1.963551630622	0.418861980472
C	-0.964788459758	4.889414885552	2.859904111084
H	-1.679712375299	4.086957365044	3.052125202382
H	0.027384392272	4.447277910390	2.968683474121
H	-1.084926920412	5.636728125378	3.641709328112
C	-1.984469639232	8.885247162987	0.000604673655
H	-2.105270301698	9.355778684758	0.974591116402
H	-1.200880639267	9.425641803868	-0.533554626544
H	-2.906187846458	9.039709051665	-0.562341190098
C	-1.077786347160	4.454329746547	-2.156996022321
H	-0.093870141509	3.984457853129	-2.227227198154
H	-1.808926463394	3.642636219470	-2.178946464080
H	-1.222785122679	5.058635268103	-3.050382891897
C	-2.629272685152	-0.306317832940	-4.011261726722
C	-2.070714790770	1.018323630710	-4.240320932706
C	-0.689169072218	1.266813358849	-4.707031933257
H	-2.771045107389	1.820986773832	-4.453071100830
H	-2.017301218230	-1.186610578618	-4.185916245000
H	-3.699074696567	-0.460146221461	-4.109302257284
H	0.087524393909	-0.677388529266	-4.202613087273
C	0.314466649299	0.302307432397	-4.606855389001
C	1.605354208134	0.586742154340	-5.020548477742
H	2.375600598848	-0.169486782908	-4.934245932909
H	2.921096411975	2.054568468736	-5.870964294020
C	1.910884962838	1.834595123833	-5.549713846359
C	0.916995804332	2.794195016468	-5.667238027592
C	-0.374059607319	2.510966489546	-5.246870804060
H	-1.151193813994	3.261866041401	-5.330283688332
H	1.146872410749	3.767323826240	-6.082065108078
O	-0.944478834095	-2.210369958393	3.950793588134
H	-1.196332827320	-2.839603012168	3.264826388435

H	-0.570692397507	-1.458164024765	3.450608468113
O	0.024213523910	-0.230314097442	-1.503745178622
H	0.725523692271	0.313899240188	-1.889977611036
H	-0.798355523543	0.057553099202	-1.957979322017

120

$^{3}\mathbf{D_{w2}}$ E_{tot}(UM06L-D3(Dichloromethane)/def2TZVP/W06) = -2708.19989638 $\langle S^2 \rangle$ = 2.0126

Ru	0.245830666017	-0.113802883428	0.770899336490
O	0.351033798293	-0.051424767871	2.522413006060
C	3.609918654487	0.544696829572	0.357707544898
C	-0.436175096336	3.260740964620	0.519694627724
C	-3.147325698549	-0.782404743548	0.784279988212
C	0.900736491888	-3.499523648962	0.602912675096
N	-1.469331807799	1.023710697201	0.665318055581
N	1.372648487553	1.580923042839	0.468262173044
N	1.936487725969	-1.260188693931	0.530377388479
N	-0.905233623224	-1.818219435616	0.719779149244
C	1.990656451099	-2.628083920857	0.523066931545
C	3.218844733002	-0.796098112038	0.414905382163
C	2.738843989356	1.637712408306	0.377917930449
C	0.903848554331	2.868754437039	0.449603396503
C	-0.438279525843	-3.107321538938	0.694199393938
C	-2.274473261446	-1.874949844184	0.777720479586
C	-2.756302738674	0.558681552980	0.731472255060
C	-1.527046414505	2.390837762266	0.610221788158
C	3.358773042367	-3.038553941810	0.404351440399
C	4.113234579772	-1.913240057982	0.338178019888
C	3.141795392380	3.009236480604	0.294639190724
C	2.015019040916	3.765147373805	0.337303428754
C	-2.899668095010	2.799871097258	0.646368664311
C	-3.654725072157	1.674705367520	0.719705568685
C	-1.552623702752	-4.004654295960	0.753007518542
C	-2.680214707522	-3.247752752923	0.804037233271
H	3.693737620996	-4.063309656447	0.370136354755
H	5.184922835590	-1.838731397706	0.240255636888
H	4.162810634033	3.347183189870	0.210711317488
H	1.937519372210	4.840350216700	0.296451114635
H	-3.238456249222	3.823578021970	0.616363396037
H	-4.730109941977	1.600357270197	0.760380990122
H	-1.478171977265	-5.080837760506	0.747223548898
H	-3.703281724398	-3.586989921782	0.848482708235
C	5.069882079146	0.831661461501	0.251875201196
C	5.843985469096	0.939491184607	1.413530358251
C	5.663211306581	0.993836000413	-1.008890830765
C	7.204896366400	1.211046795170	1.293159245030
C	7.024836375501	1.262610239323	-1.083350058648
H	7.801769785394	1.296297568219	2.195363686685
H	7.480068341063	1.385817674250	-2.060998088410
C	7.816400602124	1.376004803091	0.056567628097
C	-0.726642479161	4.723665326958	0.483835144613
C	-0.938914016924	5.366226781224	-0.744817053450
C	-0.788924545866	5.450962410144	1.678538578839
C	-1.211476347690	6.729219268296	-0.754858767819
C	-1.064352084552	6.815302071284	1.622895392916
H	-1.376262006641	7.221877495648	-1.707924471994
H	-1.113081425881	7.376003889616	2.550658265289
C	-1.278787622452	7.474951377107	0.419070314246
C	-4.611155899948	-1.070302061507	0.807126486558
C	-5.322213521200	-1.141441551597	-0.400761943501
C	-5.272761484883	-1.266810653218	2.024855124129
C	-6.686272138261	-1.405232447832	-0.365995537233
C	-6.641575301438	-1.526253420161	2.014058154917
H	-7.231611574660	-1.460296185301	-1.302891789364
H	-7.151843883877	-1.673781725350	2.960313918458
C	-7.368210156974	-1.599551756485	0.832450556621
C	1.186891541217	-4.963229246113	0.553744582865
C	1.320857996939	-5.693538696359	1.740219258587
C	1.322269997447	-5.603910178041	-0.687693940476
C	1.596649879652	-7.057090232039	1.665403195219
C	1.594618861906	-6.966513041980	-0.716330175072
H	1.704705568271	-7.618807325387	2.587569355099
H	1.697826362003	-7.457940379093	-1.678652561528
C	1.738079164473	-7.714021284966	0.449733155799
C	-4.526165562018	-1.214905070392	3.319054250360
H	-5.204493382394	-1.256415901414	4.168951631825
H	-3.829108647613	-2.050581515737	3.411806510683
H	-3.924360468663	-0.308938719504	3.408642003586

C	-4.628622712031	-0.937820230107	-1.711143627089
H	-4.217107498986	0.070497310923	-1.797765931080
H	-3.791048016645	-1.627518213651	-1.836020485163
H	-5.310667183078	-1.087690446846	-2.545659604796
C	-8.836296020998	-1.880125192448	0.838644539735
H	-9.069222516954	-2.796465620673	0.293604595922
H	-9.220291368692	-1.989465900156	1.851150988060
H	-9.397666180952	-1.080172302875	0.353267672610
C	2.031938559804	-9.178188839933	0.386717820878
H	1.238410980936	-9.721878651973	-0.128854851708
H	2.952307093158	-9.377555717050	-0.164428567092
H	2.138330355773	-9.609859890941	1.380173282850
C	1.153941541745	-5.031569509765	3.070413415200
H	1.444825057371	-5.695371675363	3.882218505999
H	1.743342196078	-4.116889836572	3.150936521368
H	0.115274662201	-4.739789470286	3.241315131852
C	1.177026769805	-4.837453760731	-1.964477505282
H	1.245051495417	-5.495191488684	-2.828727494132
H	0.220067662621	-4.314073628494	-2.015118809744
H	1.950491757573	-4.073253008949	-2.067407864815
C	5.225702418677	0.766961420450	2.764702808059
H	4.404958403683	1.468348421448	2.926724954679
H	4.803103118521	-0.231985983521	2.890159480004
H	5.957227016612	0.919429126627	3.555713314748
C	4.846560578008	0.882066365533	-2.257601218233
H	5.468934910740	0.971401323440	-3.145741161767
H	4.319498356204	-0.072654733962	-2.311934535387
H	4.082240532833	1.660781469141	-2.309097281668
C	9.279648809089	1.660009600376	-0.054795316805
H	9.802177502275	0.856435429804	-0.576904016927
H	9.468091033146	2.572226854234	-0.622778082432
H	9.742426371178	1.773985057955	0.923716090343
C	-0.564947103333	4.780028646853	2.996511135005
H	-1.283929221841	3.976627437903	3.168335702297
H	0.424497033978	4.321945654683	3.053222609823
H	-0.652350225403	5.487127260908	3.818930319542
C	-1.566323609477	8.941129572995	0.377378077098
H	-1.659876842433	9.360762407673	1.377268428049
H	-0.773809876500	9.487169319151	-0.137682740384
H	-2.490018386850	9.152760015732	-0.163056657137
C	-0.874052869275	4.600090316454	-2.028440083846
H	0.107515599938	4.144154777531	-2.175765495665
H	-1.600787156567	3.785081048793	-2.049242666103
H	-1.072180139778	5.243758641351	-2.883127041019
O	-0.935905295426	-2.079323064390	3.971089317891
H	-1.288323508020	-2.667495192413	3.293350957356
H	-0.490231667961	-1.376955141700	3.458367608300
O	0.108393400524	-0.228512729152	-1.576996321148
H	0.517420880193	0.546465439135	-1.988489153139
H	-0.814767276575	-0.205257981093	-1.867500180575

8 References

[1] A. Saib, A. Bara-Estaún, O. J. Harper, D. B. G. Berry, I. A. Thomlinson, R. Broomfield-Tagg, J. P. Lowe, C. L. Lyall, U. Hintermair, "Engineering Aspects of FlowNMR Spectroscopy Setups for Online Analysis of Solution-Phase Processes", *React. Chem. Eng.* **2021**, *6* (9), 1548–1573.

[2] A. M. R. Hall, J. C. Chouler, A. Codina, P. T. Gierth, J. P. Lowe, U. Hintermair, "Practical Aspects of Real-Time Reaction Monitoring Using Multi-Nuclear High Resolution FlowNMR Spectroscopy", *Catal. Sci. Technol.* **2016**, *6* (24), 8406–8417.

[3] I. R. Paeng, K. Nakamoto, "Resonance Raman Spectra of Reaction Intermediates in Oxidation Process of Ruthenium(II) and Iron(II) Porphyrins", *J. Am. Chem. Soc.* **1990**, *112* (9), 3289–3297.

[4] N. Jin, M. Ibrahim, T. G. Spiro, J. T. Groves, "Trans-Dioxo Manganese(V) Porphyrins", *J. Am. Chem. Soc.* **2007**, *129* (41), 12 416–12 417.

[5] (a) Seo, M. S.; In, J.-H.; Kim, S. O.; Oh, N. Y.; Hong, J.; Kim, J.; Que Jr., L.; Nam, W., Direct Evidence for Oxygen-Atom Exchange between Nonheme Oxoiron(IV) Complexes and Isotopically Labeled Water. *Angew. Chem. Int. Ed.* **2004**, *43* (18), 2417–2420. (b) Lee, K. A.; Nam, W. Determination of Reactive Intermediates in Iron Porphyrin Complex-Catalyzed Oxygenations of Hydrocarbons Using Isotopically Labeled Water: Mechanistic Insights. *J. Am. Chem. Soc.* **1997**, *119* (8), 1916–1922.

[6] G. Jiang, J. Chen, H.-Y. Thu, J.-S. Huang, N. Zhu, C.-M. Che, "Ruthenium Porphyrin-Catalyzed Aerobic Oxidation of Terminal Aryl Alkenes to Aldehydes by a Tandem Epoxidation–Isomerization Pathway", *Angew. Chem. Int. Ed.* **2008**, *47* (35), 6638–6642.

[7] J. S. Lindsey, R. W. Wagner, "Investigation of the Synthesis of Ortho-Substituted Tetraphenylporphyrins", *J. Org. Chem.* **1989**, *54* (4), 828–836.

[8] Q. Su, T. D. Hamilton, "Extending Mechanochemical Porphyrin Synthesis to Bulkier Aromatics: Tetramesitylporphyrin", *Beilstein J. Org. Chem.* **2019**, *15*, 1149–1153.

[9] S. Farhadi, A. Zabardasti, M. H. Rahmati, "Manganese(III) Porphyrin Covalently Bound to sol-gel Derived Silica (Mn(III) Porphyrinosilica): A Reusable and Green Heterogeneous Photocatalyst for Oxidative Decarboxylation of α-Arylacetic Acids with H_2O_2", *Journal of Chemical Research* **2011**, *35* (3), 157–160.

[10] M. K. Peters, F. Röhricht, C. Näther, R. Herges, "One-Pot Approach to Chlorins, Isobacteriochlorins, Bacteriochlorins, and Pyrrocorphins", *Org. Lett.* **2018**, *20* (24), 7879–7883.

[11] A. Tohara, Y. Sugawara, M. Sato, "Preparation and Characterization of Meso-Tetrakis(2,6-diaryloxyphenyl)porphyrins", *J. Porphyrins Phthalocyanines* **2006**, *10* (02), 104–116.

[12] N. Bag, S.-S. Chern, S.-M. Peng, C. K. Chang, "Bis-pocket Porphyrins without Meso-Substituents: Tetramethyltetra(2,4,6-triisopropylphenyl)porphyrin I and Tetramethyltetraterphenylporphyrin I", *Tetrahedron Lett.* **1995**, *36* (36), 6409–6412.

[13] N. Ono, H. Kawamura, M. Bougauchi, K. Maruyama, "Porphyrin Synthesis from Nitrocompounds", *Tetrahedron* **1990**, *46* (21), 7483–7496.

[14] C. Granchi, I. Caligiuri, E. Bertelli, G. Poli, F. Rizzolio, M. Macchia, A. Martinelli, F. Minutolo, T. Tuccinardi, "Development of Terphenyl-2-Methyloxazol-5(4H)-one Derivatives as Selective Reversible MAGL Inhibitors", *J. Enzyme Inhibit. Med. Chem.* **2017**, *32* (1), 1240–1252.

[15] S. K. Kristensen, S. L. R. Laursen, E. Taarning, T. Skrydstrup, "Ex Situ Formation of Methanethiol: Application in the Gold(I)-Promoted Anti-Markovnikov Hydrothiolation of Olefins", *Angew. Chem. Int. Ed.* **2018**, *57* (42), 13 887–13 891.

[16] L. A. Paquette, G. D. Maynard, "Ring and C–O Bond Fragmentation as Tools for Fingerprinting the Extent of Homolysis During Base-Catalyzed Carbon–Carbon Bond Cleavages of the Haller-Bauer, Cram, and Gilday types", *J. Org. Chem.* **1989**, *54* (21), 5054–5063.

[17] Q.-F. Liang, J.-J. Liu, J. Chen, "Sandwich Structure of a Ruthenium Porphyrin and an Amino Acid Hydrazide for Probing Molecular Chirality by Circular Dichroism", *Tetrahedron Lett.* **2011**, *52* (31), 3987–3991.

[18] J. T. Groves, M. Bonchio, T. Carofiglio, K. Shalyaev, "Rapid Catalytic Oxygenation of Hydrocarbons by Ruthenium Pentafluorophenylporphyrin Complexes: Evidence for the Involvement of a Ru(III) Intermediate", *J. Am. Chem. Soc.* **1996**, *118* (37), 8961–8962.

[19] J. T. Groves, R. Quinn, "Models of Oxidized Heme Proteins. Preparation and Characterization of a Trans-Dioxoruthenium(VI) Porphyrin Complex", *Inorg. Chem.* **1984**, *23* (24), 3844–3846.

[20] C.-J. Liu, W.-Y. Yu, C.-M. Che, C.-H. Yeung, "A Mechanistic Investigation of Alkene Epoxidation by Sterically Encumbered trans-Dioxoruthenium(VI) Porphyrins", *J. Org. Chem.* **1999**, *64* (20), 7365–7374.

[21] C. Ai, F. Zhu, Y. Wang, Z. Yan, S. Lin, "SO_2F_2-Mediated Epoxidation of Olefins with Hydrogen Peroxide", *J. Org. Chem.* **2019**, *84* (18), 11 928–11 934.

[22] O. Cussó, I. Garcia-Bosch, D. Font, X. Ribas, J. Lloret-Fillol, M. Costas, "Highly Stereoselective Epoxidation with H_2O_2 Catalyzed by Electron-Rich Aminopyridine Manganese Catalysts", *Org. Lett.* **2013**, *15* (24), 6158–6161.

[23] "Gaussian 16, Revision C.01", M. J. Frisch, G. W. Trucks, H. B. Schlegel, G. E. Scuseria, M. A. Robb, J. R. Cheeseman, G. Scalmani, V. Barone, G. A. Petersson, H. Nakatsuji, X. Li, M. Caricato, A. V. Marenich, J. Bloino, B. G. Janesko, R. Gomperts, B. Mennucci, H. P. Hratchian, J. V. Ortiz, A. F. Izmaylov, J. L. Sonnenberg, D. Williams-Young, F. Ding, F. Lipparini, F. Egidi, J. Goings, B. Peng, A. Petrone, T. Henderson, D. Ranasinghe, V. G. Zakrzewski, J. Gao, N. Rega, G. Zheng, W. Liang, M. Hada, M. Ehara, K. Toyota, R. Fukuda, J. Hasegawa, M. Ishida, T. Nakajima, Y. Honda, O. Kitao, H. Nakai, T. Vreven, K. Throssell, J. A. Montgomery, Jr., J. E. Peralta, F. Ogliaro, M. J. Bearpark, J. J. Heyd, E. N. Brothers, K. N. Kudin, V. N. Staroverov, T. A. Keith, R. Kobayashi, J. Normand, K. Raghavachari, A. P. Rendell, J. C. Burant, S. S. Iyengar, J. Tomasi, M. Cossi, J. M. Millam, M. Klene, C. Adamo, R. Cammi, J. W. Ochterski, R. L. Martin, K. Morokuma, Ö. Farkas, J. B. Foresman, D. J. Fox (Gaussian, Inc., Wallingford, CT), **2019**, see `http://www.gaussian.com`.

[24] F. Neese, “Software update: The ORCA program system – Version 5.0”, *WIREs Comput. Mol. Sci.* **2022**, *12* (5), e1606.

[25] “ORCA version 5.0.3, an *ab initio*, DFT and semiempirical SCF-MO package”, F. Neese, *Technical Directorship* F. Wennmohs, *with contributions from* D. Aravena, M. Atanasov, A. A. Auer, U. Becker, G. Bistoni, D. Bykov, V. G. Chilkuri, D. Datta, A. K. Dutta, S. Ehlert, D. Ganyushin, M. Garcia, Y. Guo, A. Hansen, B. Helmich-Paris, L. Huntington, R. Izsák, M. Kettner, C. Kollmar, S. Kossmann, M. Krupička, L. Lang, M. Lechner, D. Lenk, D. G. Liakos, D. Manganas, D. A. Pantazis, A. Papadopoulos, T. Petrenko, P. Pinski, P. Pracht, C. Reimann, M. Retegan, C. Riplinger, T. Risthaus, M. Roemelt, M. Saitow, B. Sandhöfer, I. Schapiro, A. Sen, K. Sivalingam, B. de Souza, G. Stoychev, W. Van den Heuvel, B. Wezisla, *and with contributions from collaborators* M. Kállay, S. Grimme, E. Valeev, G. Chan, J. Pittner, M. Brehm, L. Goerigk, V. Åsgeirsson, L. Ungur (Max-Planck-Institut für Kohlenforschung, Mülheim a. d. Ruhr, Germany), **2022**, see `https://orcaforum.kofo.mpg.de/`.

[26] Y. Zhao, D. G. Truhlar, “A new local density functional for main-group thermochemistry, transition metal bonding, thermochemical kinetics, and noncovalent interactions”, *J. Chem. Phys.* **2006**, *125* (19), 194 101.

[27] S. Grimme, J. Antony, S. Ehrlich, H. Krieg, “A consistent and accurate *ab initio* parametrization of density functional dispersion correction (DFT-D) for the 94 elements H–Pu”, *J. Chem. Phys.* **2010**, *132* (15), 154 104.

[28] A. V. Marenich, C. J. Cramer, D. G. Truhlar, “Universal Solvation Model Based on Solute Electron Density and on a Continuum Model of the Solvent Defined by the Bulk Dielectric Constant and Atomic Surface Tensions”, *J. Phys. Chem. B* **2009**, *113* (18), 6378–6396.

[29] F. Weigend, R. Ahlrichs, “Balanced basis sets of split valence, triple zeta valence and quadruple zeta valence quality for H to Rn: Design and assessment of accuracy”, *Phys. Chem. Chem. Phys.* **2005**, *7* (18), 3297–3305.

[30] F. Weigend, “Accurate Coulomb-fitting basis sets for H to Rn”, *Phys. Chem. Chem. Phys.* **2006**, *8* (9), 1057–1065.

[31] J. N. Harvey, M. Aschi, H. Schwarz, W. Koch, “The singlet and triplet states of phenyl cation. A hybrid approach for locating minimum energy crossing points between non-interacting potential energy surfaces”, *Theor. Chem. Acc.* **1998**, *99* (2), 95–99.

[32] “EasyMECP”, J. Rodríguez-Guerra, I. Funes-Ardoiz, F. Maseras (Zenodo), **2018**, see `https://github.com/jaimergp/easymecp`.

[33] T. Soda, Y. Kitagawa, T. Onishi, Y. Takano, Y. Shigeta, H. Nagao, Y. Yoshioka, K. Yamaguchi, “*Ab initio* computations of effective exchange integrals for H–H, H–He–H and Mn_2O_2 complex: comparison of broken-symmetry approaches”, *Chem. Phys. Lett.* **2000**, *319* (3-4), 223–230.

[34] F. Neese, “Prediction of molecular properties and molecular spectroscopy with density functional theory: From fundamental theory to exchange-coupling”, *Coord. Chem. Rev.* **2009**, *253* (5), 526–563.

[35] J. P. Malrieu, R. Caballol, C. J. Calzado, C. de Graaf, N. Guihéry, “Magnetic Interactions in Molecules and Highly Correlated Materials: Physical Content, Analytical Derivation, and Rigorous Extraction of Magnetic Hamiltonians”, *Chem. Rev.* **2014**, *114* (1), 429–492.

[36] C. Riplinger, P. Pinski, U. Becker, E. F. Valeev, F. Neese, “Sparse maps — A systematic infrastructure for reduced-scaling electronic structure methods. II. Linear scaling domain based pair natural orbital coupled cluster theory”, *J. Chem. Phys.* **2016**, *144* (2), 024 109.

[37] Y. Guo, C. Riplinger, U. Becker, D. G. Liakos, Y. Minenkov, L. Cavallo, F. Neese, “Communication: An improved linear scaling perturbative triples correction for the domain based local pair-natural orbital based singles and doubles coupled cluster method [DLPNO-CCSD(T)]”, *J. Chem. Phys.* **2018**, *148* (1), 011 101.

[38] A. Hellweg, C. Hättig, S. Höfener, W. Klopper, “Optimized accurate auxiliary basis sets for RI-MP2 and RI-CC2 calculations for the atoms Rb to Rn”, *Theor. Chem. Acc.* **2007**, *117* (4), 587–597.

[39] F. Neese, F. Wennmohs, A. Hansen, U. Becker, “Efficient, approximate and parallel Hartree–Fock and hybrid DFT calculations. A ’chain-of-spheres’ algorithm for the Hartree–Fock exchange”, *Chem. Phys.* **2009**, *356* (1–3), 98–109.

[40] F. Neese, E. F. Valeev, “Revisiting the Atomic Natural Orbital Approach for Basis Sets: Robust Systematic Basis Sets for Explicitly Correlated and Conventional Correlated *ab initio* Methods?”, *J. Chem. Theory Comput.* **2011**, *7* (1), 33–43.

[41] A. Altun, F. Neese, G. Bistoni, “Extrapolation to the Limit of a Complete Pair Natural Orbital Space in Local Coupled-Cluster Calculations”, *J. Chem. Theory Comput.* **2020**, *16* (10), 6142–6149.

[42] L.-L. Zhang, X.-Y. Wang, K.-Y. Jiang, B.-Y. Zhao, H.-M. Yan, X.-Y. Zhang, Z.-X. Zhang, Z. Guo, C.-M. Che, “A theoretical study on the oxidation of alkenes to aldehydes catalyzed by ruthenium porphyrins using O_2 as the sole oxidant”, *Dalton Trans.* **2018**, *47* (15), 5286–5297.

[43] B. Popp, S. Stahl, “Mechanism of $Pd(OAc)_2$/Pyridine Catalyst Reoxidation by O_2: Influence of Labile Monodentate Ligands and Identification of a Biomimetic Mechanism for O_2 Activation”, *Chem. Eur. J.* **2009**, *15* (12), 2915–2922.

[44] J. N. Harvey, “Spin-forbidden reactions: computational insight into mechanisms and kinetics”, *WIREs Comput. Mol. Sci.* **2014**, *4* (1), 1–14.
